# Transcriptomic changes in the microsporidia proliferation and host responses in congenitally infected embryos and larvae

**DOI:** 10.1186/s12864-024-10236-y

**Published:** 2024-04-01

**Authors:** Zigang Shen, Zhuojun Ke, Qiong Yang, Samson Teweldeberhan Ghebremichael, Tangxin Li, Tian Li, Jie Chen, Xianzhi Meng, Heng Xiang, Chunfeng Li, Zeyang Zhou, Guoqing Pan, Ping Chen

**Affiliations:** 1 College of Sericulture, Textile and Biomass Sciences, Southwest University, Tiansheng Street, Chongqing, 400716 China; 2 State Key Laboratory of Resource Insects, Southwest University, Tiansheng Street, Chongqing, 400716 China; 3 Chongqing Key Laboratory of Microsporidia Infection and Control, Southwest University, Tiansheng Street, Chongqing, 400716 China; 4 Sericulture and Agri-food Research Institute, Guangdong Academy of Agricultural Sciences, Guangzhou, China; 5 College of Animal Science and Technology, Southwest University, Chongqing, China; 6 College of Life Sciences, Chongqing Normal University, Chongqing, China

**Keywords:** *N. Bombycis*, *B. mori*, Congenital infection, Proliferation, Immune response, Metabolism, Epigenetic regulation

## Abstract

Congenital infection caused by vertical transmission of microsporidia *N. bombycis* can result in severe economic losses in the silkworm-rearing industry. Whole-transcriptome analyses have revealed non-coding RNAs and their regulatory networks in *N. bombycis* infected embryos and larvae. However, transcriptomic changes in the microsporidia proliferation and host responses in congenitally infected embryos and larvae remains unclear. Here, we simultaneously compared the transcriptomes of *N. bombycis* and its host *B. mori* embryos of 5-day and larvae of 1-, 5- and 10-day during congenital infection. For the transcriptome of *N. bombycis*, a comparison of parasite expression patterns between congenital-infected embryos and larva showed most genes related to parasite central carbon metabolism were down-regulated in larvae during infection, whereas the majority of genes involved in parasite proliferation and growth were up-regulated. Interestingly, a large number of distinct or shared differentially expressed genes (DEGs) were revealed by the Venn diagram and heat map, many of them were connected to infection related factors such as Ricin B lectin, spore wall protein, polar tube protein, and polysaccharide deacetylase. For the transcriptome of *B. mori* infected with *N. bombycis*, beyond numerous DEGs related to DNA replication and repair, mRNA surveillance pathway, RNA transport, protein biosynthesis, and proteolysis, with the progression of infection, a large number of DEGs related to immune and infection pathways, including phagocytosis, apoptosis, TNF, Toll-like receptor, NF-kappa B, Fc epsilon RI, and some diseases, were successively identified. In contrast, most genes associated with the insulin signaling pathway, 2-oxacarboxylic acid metabolism, amino acid biosynthesis, and lipid metabolisms were up-regulated in larvae compared to those in embryos. Furthermore, dozens of distinct and three shared DEGs that were involved in the epigenetic regulations, such as polycomb, histone-lysine-specific demethylases, and histone-lysine-N-methyltransferases, were identified via the Venn diagram and heat maps. Notably, many DEGs of host and parasite associated with lipid-related metabolisms were verified by RT-qPCR. Taken together, simultaneous transcriptomic analyses of both host and parasite genes lead to a better understanding of changes in the microsporidia proliferation and host responses in embryos and larvae in *N. bombycis* congenital infection.

**Supplementary Information:**

The online version contains supplementary material available at 10.1186/s12864-024-10236-y.

## Introduction

The microsporidia are a group of unicellular intracellular pathogens closely related to fungi [1]. They have a wide range of hosts, including invertebrates and all classes of vertebrates. To date, more than 1500 species belonging to over 200 genera have been described [2]. *Nosema bombycis* (*N. bombycis*), the first reported microsporidia, is a parasite that causes silkworm pébrine disease, resulting in a significant economic losses to the sericulture industry. This microsporidian can infect and transmit horizontally in host silkworm larvae, as well as vertically in host silkworm embryos [3]. Ma et al., have reported that *N. bombycis* induced a broad immune response in the larvae of silkworms [4]. Song et al., have confirmed by transcriptomic analysis that, they replicate at all stages of embryonic development [5]. The characteristics of microsporidia proliferation and host responses in congenitally infected embryos and larvae remains misunderstood till now, despite the fact that we have accumulated preliminary knowledge regarding the biology of microsporidia and host responses upon infection.

Host-pathogen interactions are highly complex and are regulated by a large number of host and pathogen genes. Pathogen infection can induce the activation of host immune genes, which facilitate the clearance of pathogens. On the other hand, the proteins produced by the pathogens have the capacity to modulate host cellular processes and the immune system of the host, enabling their survival and replication within the host. Recently, transcriptomic analysis with a focus on gene expressions of both host and pathogens has proven to be a valuable tool in enhancing our understanding of the intricate dynamics underlying host-pathogen interactions [6–9]. Several intracellular parasites, including *Leishmania*, *E. falciformis*, and *Toxoplasma gondii*, as well as their respective hosts, have been studied by dual RNA-seq analyses, which have successfully identified important coding and noncoding genes expressed by both the host and pathogen during infection [10–14]. We also used high-throughput RNA sequencing to investigate the non-coding RNAs of both the host and pathogen during *N. bombycis* congenital infection in silkworm embryos and larvae [15]. In our current study, we aim to further elucidate pathogen proliferation and host responses during congenital infection in silkworm embryos and larvae using a dual RNA-seq approach.

Zebrafish is a frequently used model organism in studying the pathogenesis of infectious diseases in vertebrates, mostly due to its well-developed immune system [16, 17]. In addition, zebrafish embryos and larvae are used to elucidate the innate host factors involved in disease progression due to the temporal distinction between the innate and adaptive immune responses [18]. *Mycobacterium marinum* leads to granuloma development in zebrafish embryos and larvae, and three main phases (early-, middle- and late-phase) in the host responses have been discovered [19]. In invertebrates, *B. mori* has been widely used to study disease pathogenicity [20]. *N. bombycis*, an important pathogen of silkworms, can infect silkworm embryos and induce immunosuppression in the host, and during the stages of larvae development, *N. bombycis* proliferates and activates host immune systems [15]. A similar phenomenon has also been reported in the zebrafish embryos and larvae infected with *M. marinum* [19]. Nevertheless, the mechanisms by which *N. bombycis* modulates the hosts for its survival and persistence within silkworms are still not fully understood. Further investigation of the interactions between *N. bombycis* and its host is essential for the advancement of utilizing *N. bombycis* as a model system for studying congenital infections in invertebrates.

In this study, we performed transcriptomic analyses to assess gene expression of the pathogen and host during *N. bombycis* congenital infection. By comparing the transcriptomes of *N. bombycis*, we found that, most of its genes related to central carbon, amino acid, and lipid metabolisms were down-regulated, whereas the majority of genes involved in cell proliferation and growth were up-regulated in larvae compared to those in embryos during infection. Several genes, such as Ricin B lectin, spore wall protein, polar tube protein, and polysaccharide deacetylase, may have a vital role in the microsporidia infection. Meanwhile, *N. bombycis* induced host cellular and humoral immune responses in turn, and these immunity responses decreased in the late stages of congenital infection in larvae. Most genes related to glucose, amino acid and lipid metabolisms were down-regulated in larvae compared to those in embryos infected with *N. bombycis*. Additionally, many host DEGs, such as polycomb protein, lysine-specific histone demethylase, and histone deacetylase, were involved in the epigenetic regulations in the host. According to the recent study conducted in parasites, such as *Toxoplasma gondii*, *Leishmania amazonensis*, and *Plasmodium falciparum*, epigenetic modification changes the host cell transcription there by promote infection [21–23]. The findings presented in this study lay the foundation for a comprehensive understanding of the molecular pathways involved in the *N. bombycis* congenital infection in silkworms.

## Materials and methods

### Data collection

All RNA-seqs are available under the Sequence Read Archive (SRA) with the BioProject accession number (PRJNA953616) as previously described [15]. The data includes 12 *N. bombycis*-infected silkworm samples and 12 normal control silkworm samples. Briefly, *N. bombycis*-infected eggs were prepared (placed in an incubator at 26 °C), and samples were collected from individuals at 5 days in the embryos (I-E5), 1 day in the larvae (I-L1), 5 days in the larvae (I-L5), and 10 days in the larvae (I-L10). Concurrently, normal control silkworm eggs were prepared at the same condition (placed in another incubator at 26 °C). Samples were collected from individuals at 5 days in the embryos (NI-E5), 1 day in the larvae (NI-L1), 5 days in the larvae (NI-L5), and 10 days in the larvae (NI-L10). RNA extraction, library construction, and sequencing were conducted as previously described [15].

### Differential expression analysis of the pathogen and the host

RNA-seq analysis of the pathogen and host was conducted by Gene Denovo Biotechnology Co. (Guangzhou, China) as previously described [15]. Expression values were calculated as fragment per kilobase per million fragments mapped (FPKM) and normalized by totals per million read. Expression values obtained at 5 days in the embryos were used as the baseline for gene expression comparison. Absolute fold change ≥2 and a *P* value < 0.05 were calculated based on FPKM, and DEGs were identified. Considering that silkworms undergo a complete metamorphosis during their life cycles, the genes involved in host development were excluded from the list of identified DEGs during parasite infection.

### Functional enrichment analysis of pathogen and host

The DEGs for both *N. bombycis* and *B. mori* were mapped to the Gene Ontology (GO) terms (http://www.geneontology.org/), and the enrichment analysis was performed using the genomes of *N. bombycis* and *B. mori* as references. Distribution of the GO categories were assigned into three categories: biological process, cellular components, and molecular functions. Then, a scatterplot visualization was constructed using REVIGO [24]. Meanwhile, the DEGs for both *N. bombycis* and *B. mori* were mapped to a Kyoto Encyclopedia of Genes and Genomes (KEGG) pathway [25–27]. Finally, the enrichment results were visualized by the ggplot2 package in R software (version 4.2.2).

### The identification of specific and shared DEGs in both the pathogen and host

To study gene functions in the different stages during *N. bombycis* infection, the specific and shared DEGs of the pathogen and host were analyzed using TBtools (version 1.098), and the heat map was also constructed using the same software [28].

### RT-qPCR validations

To validate RNA-seq results, 12 lipid metabolism genes, including 6 genes from the *N. bombycis* and 6 genes from the silkworms, were selected for relative expression analyses using RT-qPCR assays. The RT-qPCR primers are shown in Supplementary Table 1. For RT-qPCR analysis, qPCR reactions (95 °C for 5 min, followed by 40 cycles of 95 °C for 20 s, 60 °C for 30 s, and 72 °C for 20 s) were performed using SYBR Green qPCR kit (Yeasen, Shanghai, China). Tubulin and β-actin were used as the endogenous control of *N. bombycis* and *B. mori*, respectively. All qPCR experiments were conducted in triplicate.

## Statistical analyses

A student’s *t*-test was used to assess statistical differences in gene expressions between 10 days, 5 days, and 1 day in the larvae regarding 5 days in the embryos during infection of *N. bombycis*. Data are expressed as the mean of three independent experiments. *p* < 0.05 was considered statistically significant.

## Results

### Differential expression analysis of *N. Bombycis* and host silkworm genes in congenitally infected silkworm embryos and larvae

To investigate the differences in the gene profile of *N. bombycis* in embryos and larvae, expression values (FPKM) obtained at 5 days in the embryos were used as the baseline for gene expression comparison. As previously described, there were 223 (104 up-regulated and 119 down-regulated), 109 (79 up-regulated and 30 down-regulated), and 251 (200 up-regulated and 51 down-regulated) DEGs in *N. bombycis* at I-L1, I-L5 and I-L10, respectively [14]. These different DEGs in *N. bombycis* were listed in Supplementary Table 2.

**Fig. 1:**
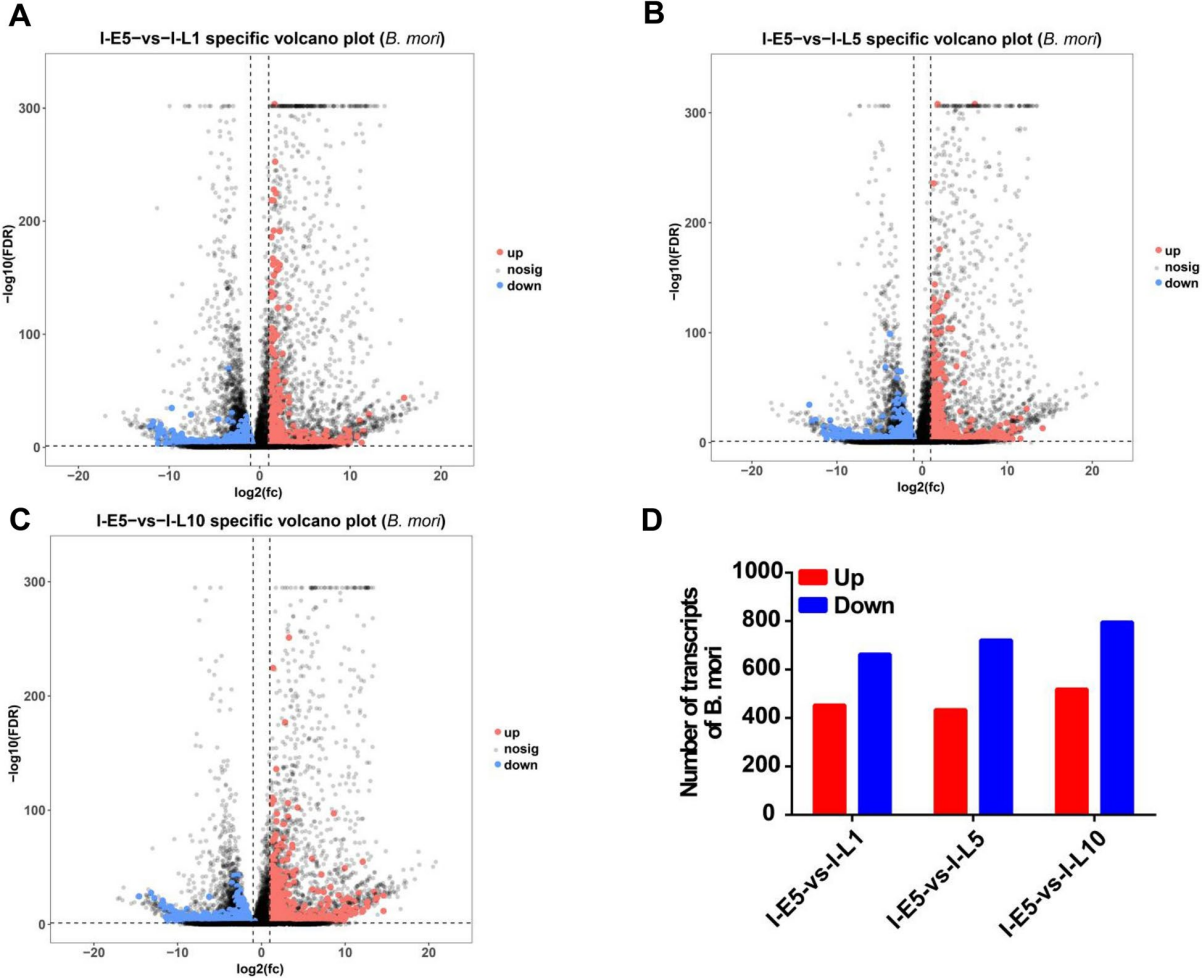
Differential gene expression of silkworm in congenitally infected embryos and larvae. **A**: Differential gene expression of silkworm between 5-day embryos and 1-day larvae during *N. bombycis* infection are shown in a volcano plot; **B**: Differential gene expression of silkworm between 5-day embryos and 5-day larvae during *N. bombycis* infection are shown in a volcano plot; **C**: Differential gene expression of silkworm between 5-day embryos and 10-day larvae during *N. bombycis* infection are shown in a volcano plot; **D**: The number of DEGs of silkworm at 1-, 5-, and 10-day larvae using as control the expression values at 5-day embryos

For silkworms, as shown in sFig. 1A-C, there were 5660, 5377, and 5574 DEGs at I-L1, I-L5 and I-L10, respectively, compared to those at I-E5 during *N. bombycis* infection. In normal silkworms, there were 5373, 5337, and 5257 DEGs at I-L1, I-L5 and I-L10, respectively, compared to those at I-E5, among which 4544, 4222, and 4260, respectively, were shared with the infected silkworms with the remaining (1116, 1155, and 1314 genes, respectively) being considered as specific DEGs (Fig. 1A-C). As shown in Fig. 1D, there were 453 up-regulated and 663 down-regulated, 434 up-regulated and 721 down-regulated, and 519 up-regulated and 795 down-regulated genes at I-L1, I-L5, and I-L10 during *N. bombycis* infection, respectively. These different DEGs in silkworms were listed in Supplementary Table 3.

### Functional enrichment analysis of *N. Bombycis* genes in congenitally infected silkworm embryos and larvae

**Fig. 2:**
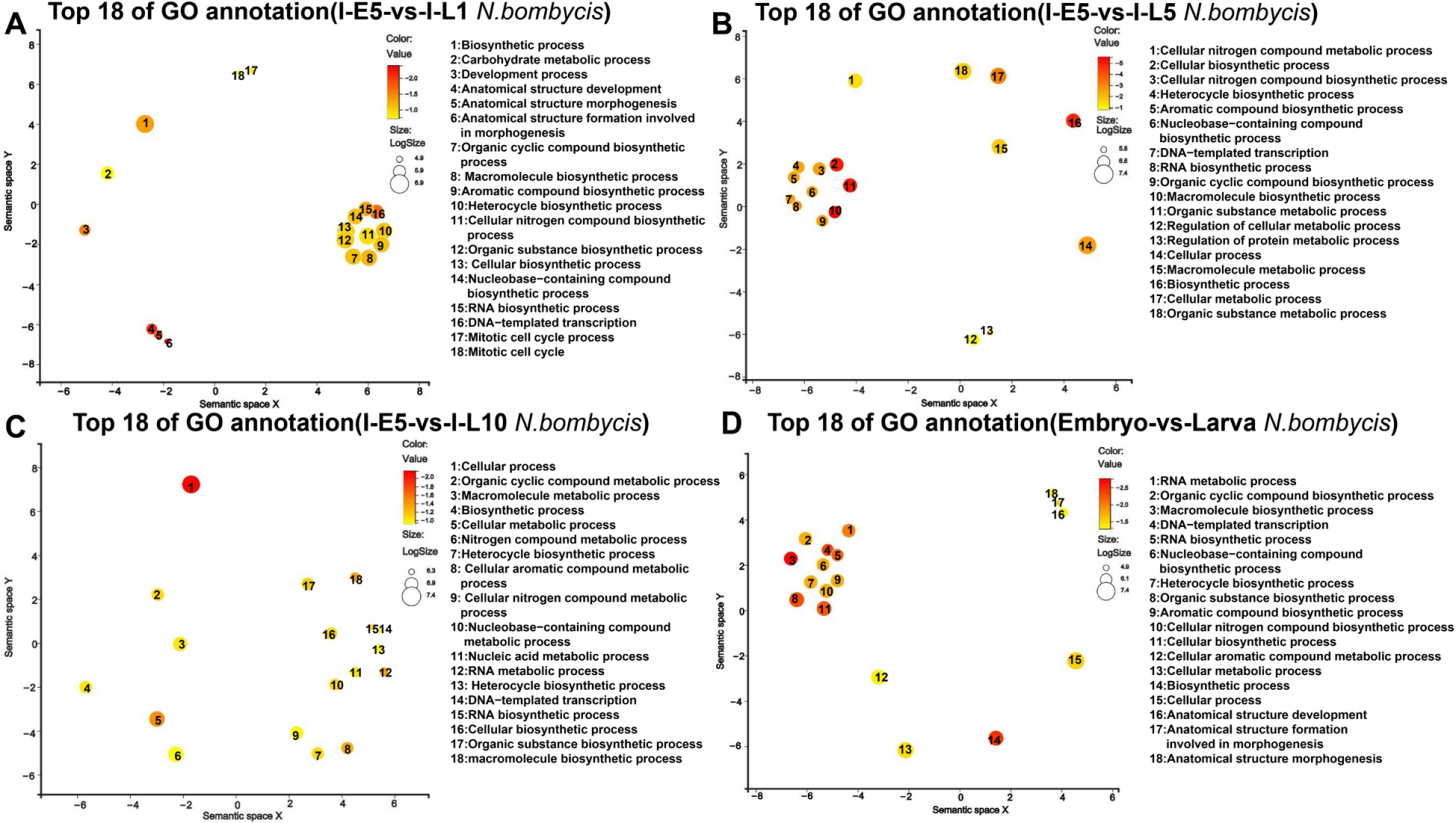
GO enrichment analysis of significant differential genes of *N. bombycis* in congenitally infected silkworm embryos and larvae. **A**: GO enrichment analysis of significant differential genes of *N. bombycis* between 5-day embryos and 1-day larvae during *N. bombycis* infection; **B**: GO enrichment analysis of significant differential genes of *N. bombycis* between 5-day embryos and 5-day larvae during *N. bombycis* infection; **C**: GO enrichment analysis of significant differential genes of *N. bombycis* between 5-day embryos and 10-day larvae during *N. bombycis* infection; **D**: GO enrichment analysis of significant differential genes in *N. bombycis* between embryo and larva during infection. Bubble color indicates *p*-value, and size indicates the frequency of the GO term in the underlying GOA database. The axes in the plot have no intrinsic meaning. The guiding principle is that semantically similar GO terms should remain close together in the plot

To identify key molecular pathways involved in the congenitally infected silkworm embryos and larvae, GO enrichment analysis of the different DEGs for *N. bombycis* was performed. The results showed that many DEGs in *N. bombycis* were involved in RNA biosynthetic process, DNA-templated transcription, biosynthetic process, and carbon metabolic process at 1 day in the larvae during *N. bombycis* infection (Fig. 2A). As shown in Fig. 2B, in addition to genes related to RNA biosynthetic process, DNA-templated transcription and biosynthetic process, several genes were involved in regulation of cellular protein metabolic process. GO enrichment analysis revealed that many DEGs in *N. bombycis* belonged to RNA biosynthetic process, DNA-templated transcription, biosynthetic process, cellular metabolic process, cellular nitrogen compound metabolic process, and nitrogen compound metabolic process (Fig. 2C). As shown in Fig. 2D, all DEGs of *N. bombycis* were mapped to GO terms. Many DEGs were involved in DNA-templated transcription, RNA biosynthetic process and cellular metabolic process.

**Fig. 3:**
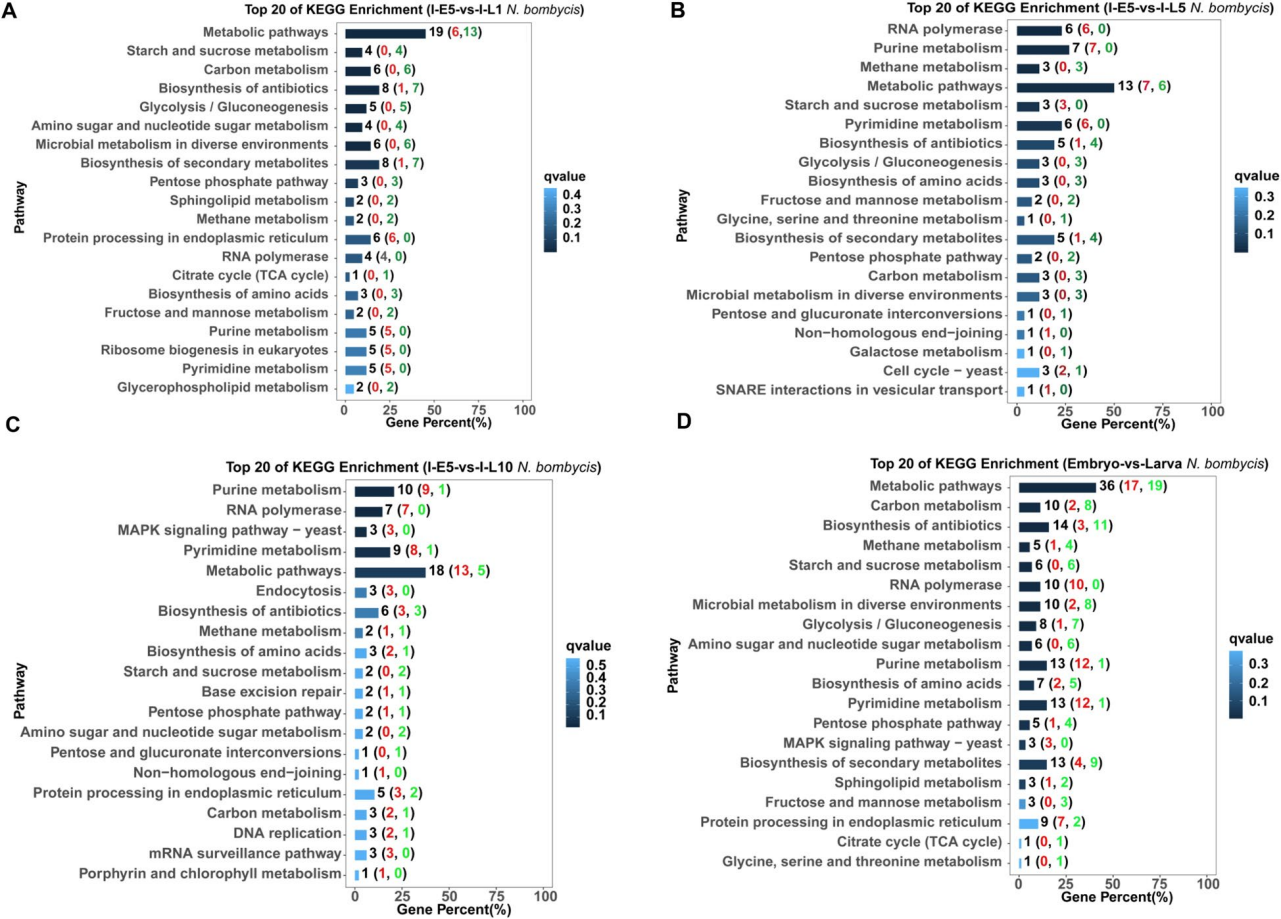
KEGG enrichment analysis of significant differential genes of *N. bombycis* in congenitally infected silkworm embryos and larvae. **A**: KEGG enrichment analysis of significant differential genes of *N. bombycis* between 5-day embryos and 1-day larvae during *N. bombycis* infection; **B**: KEGG enrichment analysis of significant differential genes of *N. bombycis* between 5-day embryos and 5-day larvae during *N. bombycis* infection; **C**: KEGG enrichment analysis of significant differential genes of *N. bombycis* between 5-day embryos and 10-day larvae during *N. bombycis* infection; **D**: KEGG enrichment analysis of significant differential genes in *N. bombycis* between embryo and larva during infection. A red font represents the number of up-regulated genes, and a green font represents the number of down-regulated genes

KEGG enrichment analysis of the DEGs for *N. bombycis* was performed to identify key pathways altered during infection. KEGG enrichment analysis revealed that 10 to 20 DEGs in *N. bombycis* belonged to metabolic pathways, and several DEGs in *N. bombycis* belonged to RNA polymerase, protein processing in endoplasmic reticulum, biosynthesis of amino acids, and ribosome biogenesis at 1 day in the larvae, respectively (Fig. 3A). We found that except for genes related to purine and pyrimidine metabolism, other genes related to metabolisms were down-regulated at 1 day, 5 days, and 10 days in the larvae compared to those at 5 days in the embryos (Fig. 3B-C). Additionally, genes related to RNA polymerase, ribosome biogenesis, and protein processing in the endoplasmic reticulum were up-regulated at 1 day in the larvae. Meanwhile, several up-regulated DEGs were related to cell cycle, non-homologous end-joining, and SNARE interactions in vesicular transport at 5 days in the larvae.

Several DEGs were involved in the mitogen-activated protein kinase (MAPK) signaling pathway, endocytosis, DNA replication, and mRNA surveillance pathway, and most of these genes are up-regulated at 10 days in the larvae compared to those at 5 days in the embryos. Subsequently, in order to understand the difference in microsporidia proliferation in embryos and larvae during infection, all the DEGs were mapped to KEGG enrichment analysis in larvae, compared to those in embryos. KEGG enrichment analysis showed that some DEGs in *N. bombycis* were involved in metabolic pathway, RNA polymerase, MAPK signaling pathway, and protein processing in endoplasmic reticulum in larvae (Fig. 3D). Notably, most down-regulated genes of *N. bombycis* in larvae were related to central carbon metabolism (sFig. 2A-F). Furthermore, in *N. bombycis*, some genes related to RNA polymerase, purine metabolism, pyrimidine metabolism, MAPK signaling pathway, and protein processing in the endoplasmic reticulum were up-regulated in larvae during infection. Overall, the data suggest that the spore proliferation activity was weaker in embryos than in larvae, and the spores may inhibit their basic metabolic activity and obtain nutrients from the host in larvae.

### Functional enrichment analysis of silkworm genes in congenitally infected silkworm embryos and larvae

**Fig. 4:**
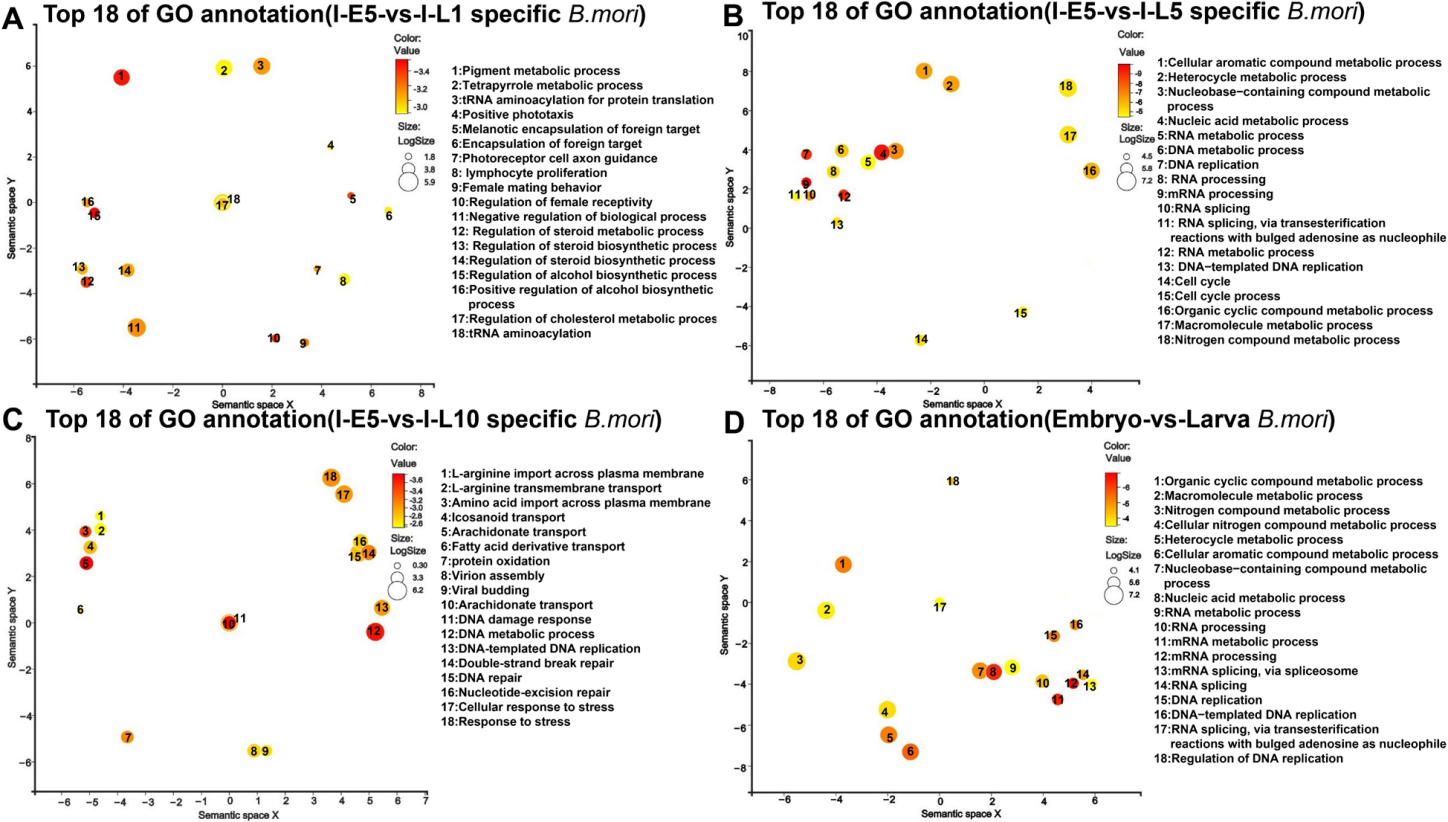
GO enrichment analysis of significant differential silkworm genes in congenitally infected silkworm embryos and larvae. **A**: GO enrichment analysis of significant differential genes of silkworm between 5-day embryos and 1-day larvae during *N. bombycis* infection; **B**: GO enrichment analysis of significant differential genes of silkworm between 5-day embryos and 5-day larvae during *N. bombycis* infection; **C**: GO enrichment analysis of significant differential genes of silkworm between 5-day embryos and 10-day larvae during *N. bombycis* infection; **D**: GO enrichment analysis of significant differential genes in silkworm between embryo and larva during infection. Bubble color indicates *p*-value, and size indicates the frequency of the GO term in the underlying GOA database. The axes in the plot have no intrinsic meaning. The guiding principle is that semantically similar GO terms should remain close together in the plot

In the same way, GO enrichment analysis of the different DEGs for silkworms was performed. As shown in Fig. 4A, many DEGs were involved in tRNA aminoacylation for protein translation, tRNA aminoacylation, regulation of cholesterol process, regulation of cellular protein metabolic process, regulation of steroid metabolic process, and lymphocyte proliferation in 1-day larvae compared to those in 5-day embryos during infection. GO enrichment analysis revealed that many DEGs were related to DNA replication, RNA processing, RNA-splicing, DNA-templated DNA replication, cell cycle, cell cycle process, nitrogen compound metabolic process, and macromolecule metabolic process in 5-day larvae compared to those in 5-day embryos during infection (Fig. 4B). As shown in Fig. 4C, many DEGs belonged to DNA repair double-strand break repair, nucleotide-excision repair, cellular response to stress, response to stress, virion assembly, viral budding, protein oxidation, and amino acid across plasma membrane in 10-day larvae. Lastly, all DEGs of silkworms were mapped to GO terms, and a large number of DEGs were involved in the regulation of DNA replication, DNA replication, RNA processing, RNA metabolic process, DNA strand elongation, DNA strand elongation involved in replication, and nitrogen compound metabolic process (Fig. 4D).

**Fig. 5:**
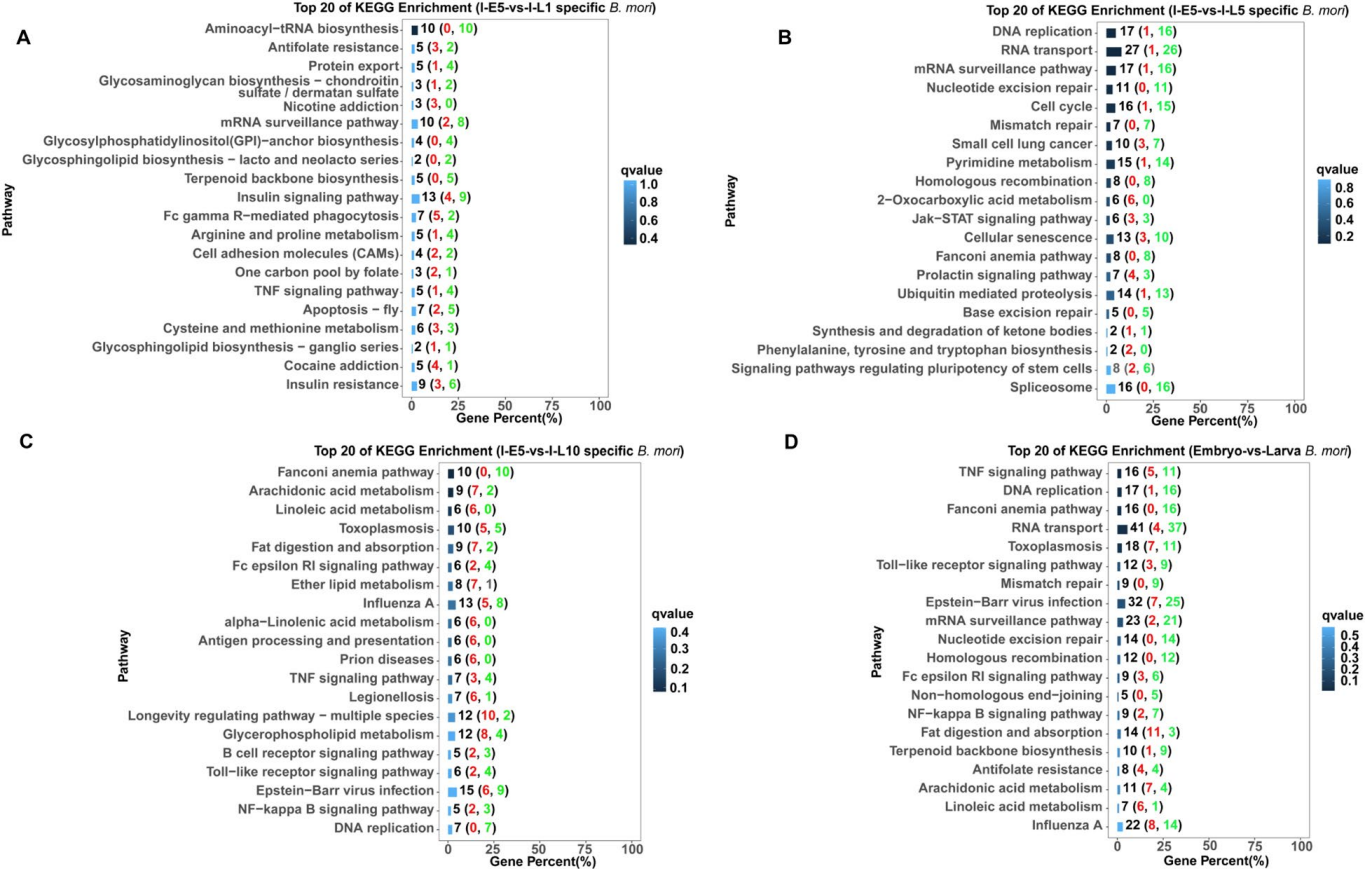
KEGG enrichment analysis of significant differential silkworm genes in congenitally infected silkworm embryos and larvae. **A**: KEGG enrichment analysis of significant differential silkworm genes between 5-day embryos and 1-day larvae during *N. bombycis* infection; **B**: KEGG enrichment analysis of significant differential silkworm genes between 5-day embryos and 5-day larvae during *N. bombycis* infection; **C**: KEGG enrichment analysis of significant differential silkworm genes between 5-day embryos and 10-day larvae during *N. bombycis* infection; **D**: KEGG enrichment analysis of significant differential silkworm genes between embryos and larvae during *N. bombycis* infection. A red font represents the number of up-regulated genes, and a green font represents the number of down-regulated genes

For silkworms, KEGG enrichment analysis revealed that 10, 5 and 10 DEGs were related to aminoacyl-tRNA biosynthesis, protein export, and mRNA surveillance pathway during infection at 1 day in the larvae, respectively. Almost all of these genes were down-regulated compared to those at 5 days in the embryos (Fig. 5A), indicating that *N. bombycis* inhibits host protein biosynthesis and transport in 1-day larvae. Meanwhile, some genes related to the biosynthesis pathway, including glycosylphosphatidylinostitol (GPI)-anchor, glycosphingolipid, and terpenoid backbone biosynthesis, were down-regulated in 1-day larvae. Additionally, some genes related to insulin signaling pathways, insulin resistance, phagocytosis, and apoptosis were expressed differently in 5-day embryos and 1-day larvae. As shown in Fig. 5B, at 5 days in the larvae, KEGG enrichment analysis evidenced that a large number of down-regulated DEGs in silkworms were involved in DNA replication, RNA transport, mRNA surveillance pathway, nucleotide excision repair, cell cycle, and mismatch repair, homologous recombination, cellular senescence, fanconi anemia pathway, ubiquitin-mediated proteolysis, base excision repair, and spliceosome, suggesting that *N. bombycis* causes irreversible damage to the silkworms. In contrast, several genes related to 2-oxocarboxylic acid metabolism and phenylalanine, tyrosine, and tryptophan biosynthesis were up-regulated in 5-day larvae compared to those in 5-day embryos during infection. At 10 days in the larvae, we only found that a few genes related to the fanconi anemia pathway and DNA replication were down-regulated compared to those at 5 days in the embryos (Fig. 5C). Additionally, some genes related to Fc epsilon RI signaling pathway, TNF signaling pathway, Toll-like receptor signaling pathway, and human diseases were expressed differently in 10-day larvae and 5-day embryos.

Interestingly, some genes related to arachidonic acid metabolism, linoleic acid metabolism, fat digestion and absorption, ether lipid metabolism, alpha-linoleic acid metabolism, and glycerophospholipid metabolism were up-regulated in 10-day larvae compared to those in 5-day embryos during infection, suggesting that those lipid-related metabolisms play an important role in the *N. bombycis* infection in larvae. Lastly, all the DEGs of silkworms were mapped to KEGG enrichment analysis. As shown in Fig. 5D, the results showed that some DEGs related to DNA replication, Fanconi anemia pathway, RNA transport, mismatch repair, mRNA surveillance pathway, nucleotide excision repair, homologous recombination, and non-homologous end-joining were down-regulated in larvae compared to those in embryos. KEGG enrichment analysis revealed that some genes related to the Toll-like receptor signaling pathway, NF-kappa B signaling pathway, TNF signaling pathway, and some diseases were expressed differently in larvae and embryos. Notably, several genes related to fat digestion and absorption, arachidonic acid metabolism, and linoleic acid metabolism were up-regulated in larvae compared to those in larvae during infection (sFig. 3A-C), suggesting that *N. bombycis* may utilize host lipids to facilitate its own replication in larvae.

### The specific and shared DEGs analysis of the pathogen and host in congenitally infected silkworm embryos and larvae

**Fig. 6:**
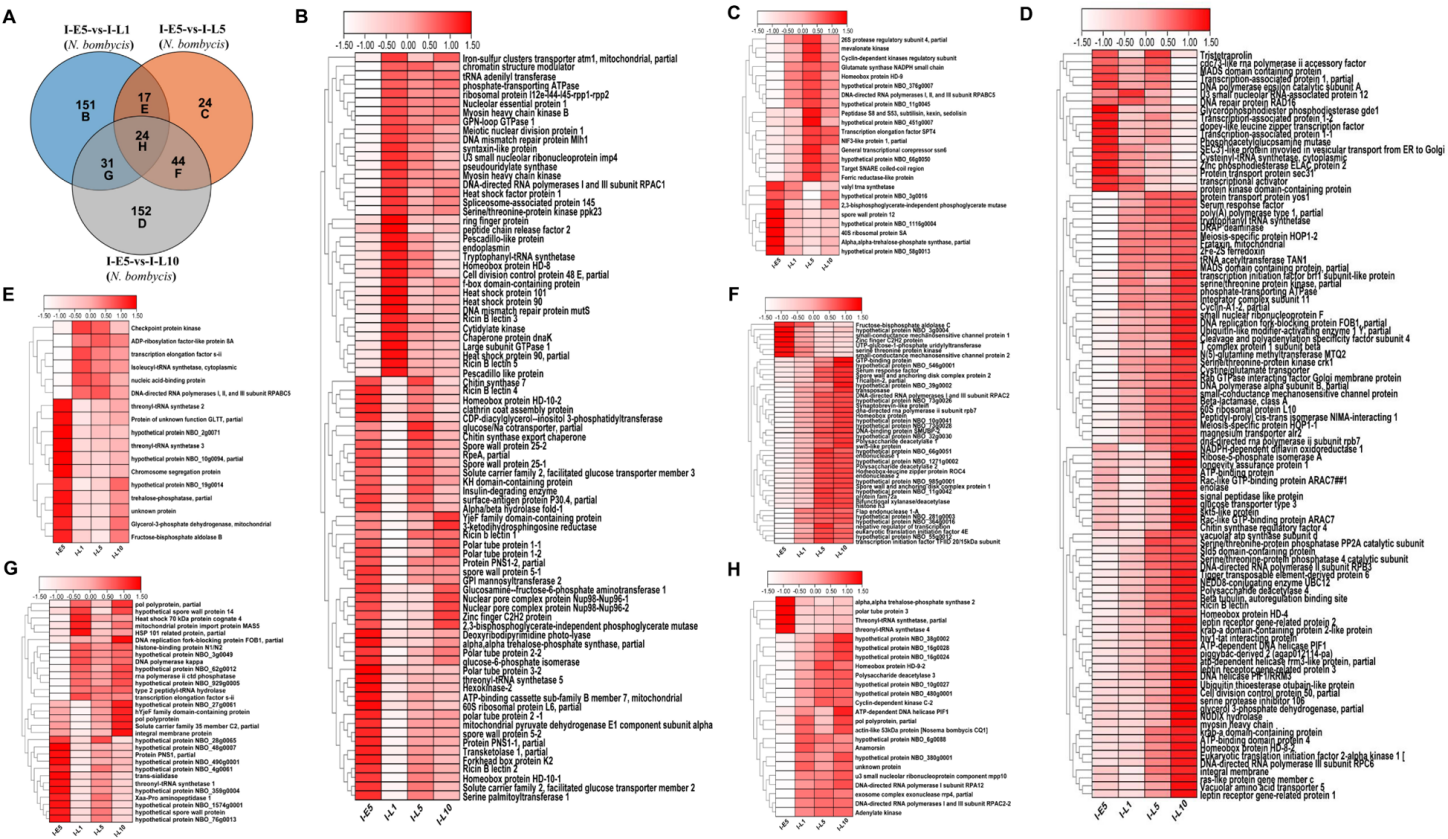
The specific and shared DEGs analysis of *N. bombycis* during the congenital infection. **A**: Venn diagram showing the number of specific and shared genes in *N. bombycis* in congenitally infected silkworm embryos and larvae; **B**: The heat maps show a subset of specific genes expressed by *N. bombycis* in 1-day larvae; **C**: The heat maps show a subset of specific genes expressed by *N. bombycis* in 5-day larvae; **D**: The heat maps show a subset of specific genes expressed by *N. bombycis* in 10-day larvae; **E**: The heat maps show a subset of shared genes expressed by *N. bombycis* in 1-day and 5-day larvae; **F**: The heat maps show a subset of shared genes expressed by *N. bombycis* in 5-day and 10-day larvae; **G**: The heat maps show a subset of shared genes expressed by *N. bombycis* in 1-day and 10-day larvae; **H**: The heat maps show a subset of shared genes expressed by *N. bombycis* in 1-, 5-, and 10-day larvae

To further investigate gene functions, those DEGs of the pathogen and host were divided into the specific and shared DEGs by the Venn diagram. As shown in Fig. 6A, for the pathogen, 151, 24, and 152 genes were the specific DEGs in larvae at 1, 5, and 10 days, respectively, and 17, 44, 31, and 24 genes were shared in DEGs, among which 24 were shared over all the larvae stages, compared to those in embryos. In the larvae at 1 day, the specific DEGs of the pathogen included heat shock protein, Ricin B lectin, spore wall protein, and polar tube protein that involved in infection and stress response (Fig. 6B). Additionally, hexokinase-2, glucose-6-phosphate isomerase, and serine palmitoyl transferase 1 related to metabolism pathways were specific DEGs in 1-day larvae compared to those in 5-day embryos. For larvae at 5 days, 24 specific DEGs of the pathogen included subtilisin, spore wall protein 12, mevalonate kinase, and valyl-tRNA synthetase (Fig. 6C). The specific DEGs of the pathogen included DNA replication fork-blocking protein FOB1, meiosis-specific protein HOP1, serine protease inhibitor 106, leptin receptor gene-related protein, longevity assurance protein 1, serum response factor 1, and 2Fe-2S ferredoxin at the larvae for 10 days, compared to those at the embryos for 5 days (Fig. 6D). Additionally, Ricin B lectin 6 and polysaccharide deacetylase 3 related to infection process were up-regulated in 10-day larvae compared to those in 5-day embryos. As shown in Fig. 6E, at 1-day and 5-day larvae, 17 common DEGs included isoleucyl-tRNA synthetase and threonyl-tRNA synthetase, that were involved in amino acid biosynthesis. Meanwhile, trehalose-phosphatase, glycerol-3-phosphatase dehydrogenase, and fructose-bisphosphate aldolase related to metabolisms were down-regulated in 1-day and 5-day larvae compared to those in 5-day embryos. At 5-day and 10-day larvae, 44 common DEGs encoding polysaccharide deacetylase 1, polysaccharide deacetylase 2, serum response factor, endonuclease, and spore wall and anchoring disk complex protein 1, and spore wall and anchoring disk complex protein 2 were up-regulated, compared to those in 5-day embryos (Fig. 6F). At 1-day and 10-day larvae, 31 common DEGs encode trans-sialidase, mitochondrial protein import protein MAS5, and heat shock protein (Fig. 6G). Notably, at 1, 5, 10- day larvae, 24 common DEGs included the down-regulated genes, such as trehalose-phosphate synthase, polar tube protein 3, and threonyl-tRNA-synthase, and the up-regulated genes, such as polysaccharide deacetylase 3, anamorsin, exosome complex exonuclease RRP4, and adenylate kinase (Fig. 6H).

**Fig. 7:**
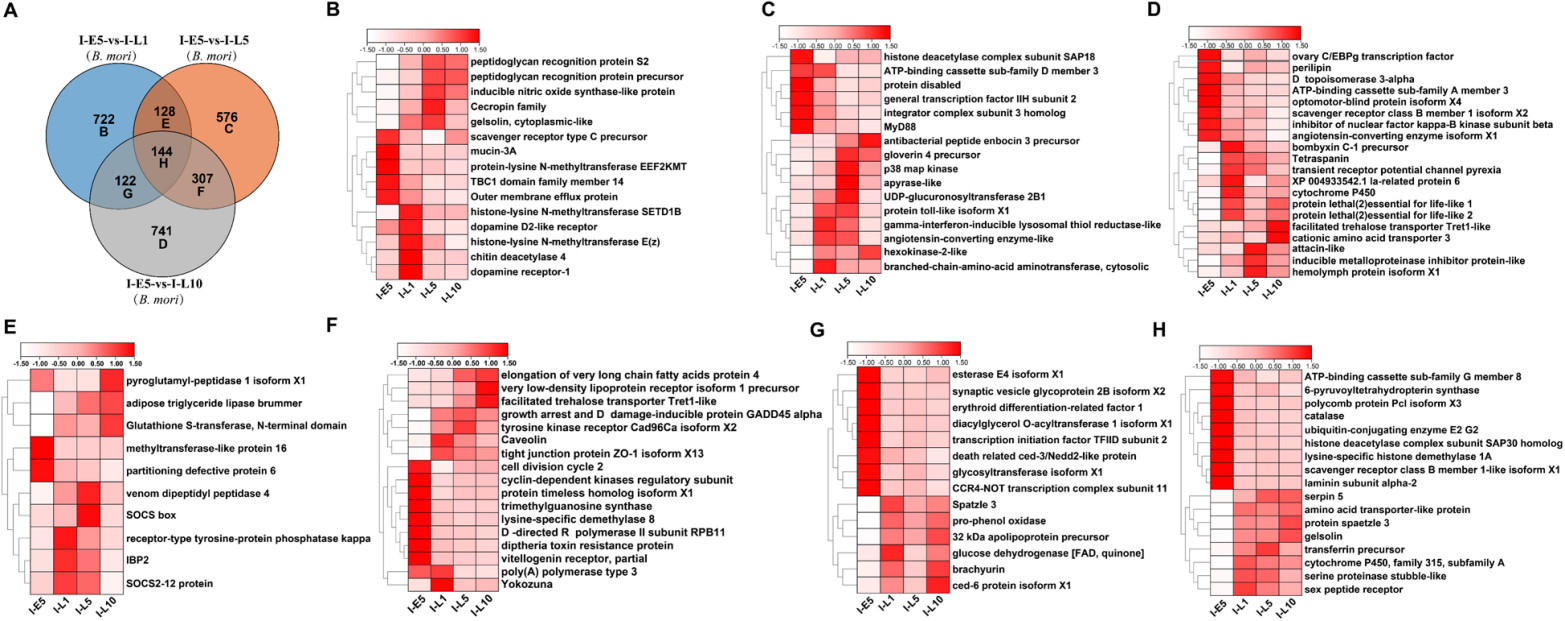
The specific and shared DEGs analysis of silkworm during the congenital *N. bombycis* infection. **A**: Venn diagram showing the number of exclusive and shared genes in silkworm in congenitally infected silkworm embryos and larvae; **B**: The heat maps show a subset of specific genes expressed by silkworm in 1-day larvae; **C**: The heat maps show a subset of specific genes expressed by silkworm in 5-day larvae; **D**: The heat maps show a subset of specific genes expressed by silkworm in 10-day larvae; **E**: The heat maps show a subset of shared genes expressed by silkworm in 1-day and 5-day larvae; **F**: The heat maps show a subset of shared genes expressed by silkworm in 5-day and 10-day larvae; **G**: The heat maps show a subset of shared genes expressed by silkworm in 1-day and 10-day larvae; **H**: The heat maps show a subset of shared genes expressed by silkworm in 1-, 5-, and 10-day larvae

For host, 722, 576, and 741 DEGs were unique in larvae at 1, 5, and 10 days, respectively, and 128, 307, 122, and 144 DEGs were shared among all stages of larvae (Fig. 7A). As shown in Fig. 7B, the specific DEGs in larvae at 1 day included immune-related genes, such as peptidoglycan recognition protein, scavenger receptor, and cecropin, and dopamine receptor. In larvae at 5 days, the specific DEGs included angiotensin-converting enzyme, hexokinase-2, branched-chain amino acid aminotransferase, and immune-related genes (MyD88, enbocin, and gloverin) (Fig. 7C). As shown in Fig. 7D, the unique DEGs included perilipin, cytochrome P450, protein lethal (2) essential for life 1, protein lethal (2) essential for life 2, and cationic amino acid transporter in larvae at 10 days. At 1-day and 5-day larvae, 128 common DEGs included suppressor of cytokine signaling (*SOCS*), insulin-related peptide binding protein 2 (*IBP2*), venom dipeptidyl peptidase 4, and partitioning defective protein 6, compared to those in embryos at 5 days (Fig. 7E). As shown in Fig. 7F, compared to those at embryos, for 5-day and 10-day larvae, these common DEGs consisted of Caveolin, lysine-specific demethylase 8, vitellogenin receptor, and Yokozuna. For 1-day and 10-day larvae, 122 common DEGs included diacylglycerol O-acyltransferase 1, Spätzle 3, pro-phenol oxidase, and brachyuran (Fig. 7G). Importantly, those 144 common DEGs included polycomb, histone deacetylase, and lysine-specific histone demethylase, which were down-regulated at all the larvae stages compared to those at embryos. Additionally, serpin 5, amino acid transport, protein spätzle 3, transferrin, and gelsolin were up-regulated at each stage of the larvae compared to those in embryos (Fig. 7H).

### Validation of differently expressed host and pathogen genes by quantitative reverse transcription polymerase chain reaction (RT-qPCR)

**Fig. 8:**
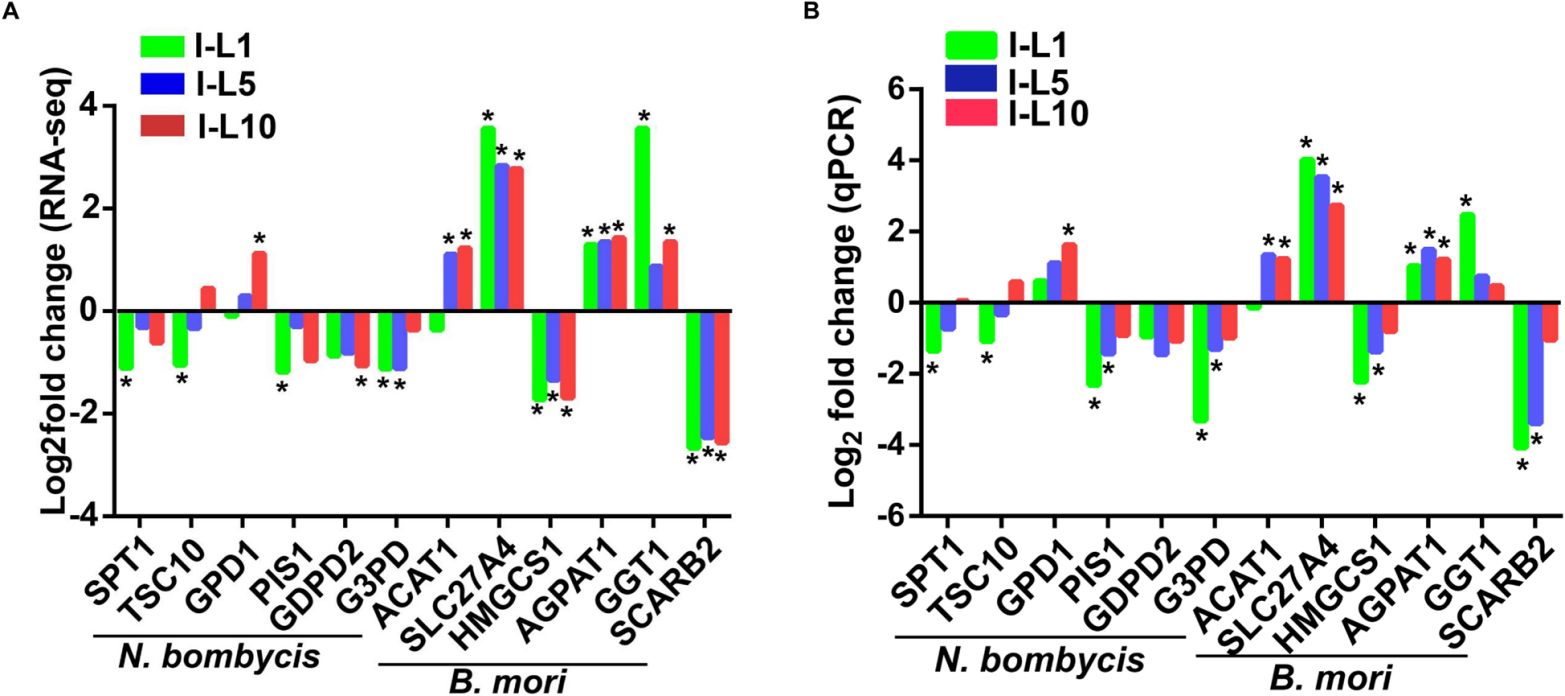
The individual fold changes obtain through RNA-seq and RT-qPCR for lipid-related genes from *N. bombycis* and silkworm differentially expressed during infection. **A**: The individual fold changes obtain through RNA-seq for lipid-related genes from *N. bombycis* and silkworm differentially expressed during infection; **B**: The individual fold changes obtain through RT-qPCR for lipid-related genes from *N. bombycis* and silkworm differentially expressed during infection. Fold change were estimated using reference expression values obtained at 5-day larvae for the same genes. *Indicates significant differences regarding at 5-day larvae in RNA-seq and RT-qPCR. *p* < 0.05 is considered statistically significant

Transcriptomic analyses showed that *N. bombycis* lipid-related genes, such as serine palmitoyltransferase 1 (*SPT1*), 3-ketodihydrosphingosine reductase (Temperature-sensitve csg2Δ suppressor 10, *TSC10*), CDP-diacylglycerol-inositol 3-phosphatidyltransferase (Phospatidylinositol synthase, *PIS1*), glycerophosphodiester phosphodiesterase 2 (*GDPD2*), and glyceraldehyde-3-phosphate dehydrogenase (*G3PD*), were down-regulated in larvae at 1, 5, 10 days, compared to their expressions in embryos at 5 days (Fig. 8A). Additionally, glycerol3-phosphate dehydrogenase 1 (*GPD1*) in *N. bombycis* was up-regulated in larvae at 1, 5, 10 days, compared to it expression in embryos at 5 days. For silkworms, lipid-related genes, such as acetoacetyl-CoA thiolase 1 (*ACAT1*), fatty acid transport protein (Solute carrier family 27 member 4, *SLC27A4*), 1-acylglycerol-3-phosphate O-acyltransferase 1 (*AGPAT1*), and gamma-glutamyltranspeptidase 1 (*GGT1*), were up-regulated, and 3-hydroxy-3-methylglutaryl-CoA synthase 1 (*HMGCS1*) and scavenger receptor class B member 2 (*SCARB2*) genes were down-regulated in larvae at 1, 5, 10 days, compared to their expressions in embryos at 5 days (Fig. 8A). To validate our findings revealed by transcriptomic analysis, a subset of those aforementioned genes was selected, and RT-qPCR quantified their relative expressions. As shown in Fig. 8B, the variations in gene expression quantified by RT-qPCR were largely consistent with what we have observed in transcriptomic analysis. In addition to confirming that *N. bombycis* utilizes host lipids to facilitate its replication, our RT-qPCR data validated that RNA-seq is a powerful tool for the comprehensive evaluation of genetic alterations both in the host and parasites.

## Discussion

*Bombyx mori* is a well-known lepidopteran insect, and its complete life cycle includes four different stages: embryo (egg), larva, pupa, and moth (adult). Given that its genome has been fully sequenced, *B. mori* has now become an important insect, not only for the sericulture industry but also for molecular biology studies. *N. bombycis* can transovarially transmit to silkworm embryos, leading to congenital infection [5],  as well as it can also infect silkworm larvae through horizontal transmission. However, the proliferation of *N. bombycis* and host responses in silkworm embryos and larvae are still not well understood. In this study, using dual RNA-seq, we were able to evaluate the systemic gene expressions of *N. bombycis* and its host silkworm at the embrynic and larval stages. We identified novel pathways involved in the interaction of the pathogen and host. Due to its capacity to survive in the host and cause congenital infection, *N. bombycis* is not only a significant pathogen for sericulture but also an important model for studying immune evasion strategies, pathogen survival mechanisms, and immune responses in invertebrates. Our study on the congenital infection of *N. bombycis* at the transcriptomics level can help researchers to better utilize this pathogen as a model organism.

The comparative transcriptomics analysis in our study identified the genes of the pathogen that were differently expressed in *N. bombycis*-infected embryos and larvae. We identified that many parasitic genes, including RNA polymerase, purine, and pyrimidine metabolism, were up-regulated in larvae compared to those in embryos, suggesting that the replication of microsporidia was more efficient in larvae. Additionally, many genes involved in protein processing in the endoplasmic reticulum were up-regulated in larvae, compared to those in embryos, indicating that protein processing capacity in larvae was greater than that in embryos. Furthermore, three genes related to the MAPK signaling pathway were up-regulated in larvae. The MAPK signaling pathway is closely related to cell proliferation and differentiation [29]. It has been reported that the microsporidian parasite *Encephalitozoon cuniculi* exhibited reduced intracellular replication in the parasite-infected mice treated with a MAPK inhibitor [30], suggesting that the MAPK signaling pathway plays an important role in the parasite growth.

Moreover, many genes in metabolic pathways, such as carbon metabolism, starch and sucrose metabolism, glycolysis, pentose phosphate pathway, and sphingolipid metabolism, were down-regulated in larvae, suggesting that the larvae’s survival is more dependent on the nutrients from the host compared to parasite growth during the embryonic stage. He et al., found that the majority of the genes involved in trehalose synthesis metabolism, glycolysis, and the pentose phosphate pathway were down-regulated in the sporoplasm, compared to those in the mature spores, suggesting that the sporoplasm may inhibit its basic metabolic activity and obtain the nutrients from host silkworm [31]. Some studies have reported that two kinases are involved in nutrient signaling: the target of rapamycin (TOR) kinase and AMP-activated kinase (AMPK), which are essential for metabolism change during the lytic cycle of intracellular parasites, such as *Trypanosoma brucei* and *Toxoplasma gondii* [32, 33]. However, the mechanism of metabolic change in microsporidia is unclear. Taken together, the evidence may indicate that the gene expressions of pathogens are characterized by down-regulated genes related to central carbon metabolism and up-regulated genes related to cell proliferation and growth.

Notably, several parasite genes, such as polysaccharide deacetylase, spore wall protein, polar tube protein, and Ricin B lectin, were differentially expressed, as revealed by our comparative transcriptomic analysis of embryos and larvae. We found that three polysaccharide deacetylase genes were up-regulated in larvae. Polysaccharide deacetylases are conserved and found in many bacteria, fungi, and insects [34]. They are mainly responsible for metal-dependent deacetylation of O- or N- acetylated polysaccharides, including peptidoglycan, chitin, and acetylxylan, which is crucial for cell shape, neutral polysaccharide synthesis, and *Bacillus anthracis* pathogenicity [35]. Xu et al., reported that a polysaccharide deacetylase from *Puccinia striiformis* f. sp. Tritici (Pst), Pst_13661, may modify the fungal cell wall to prevent being recognized by host plants [36]. Spore wall protein 12 and polar tube protein 3 have been described as the putative virulence genes that are involved in establishing host-pathogen interactions [37, 38]. Ricin B lectins were identified in microsporidian genomes, and those proteins enhance spore adhesion to host cells [39, 40]. In *N. bombycis*, some Ricin B lectin proteins were verified, and NbRBL28 is involved in controlling the cell cycle progression by regulating the expression of host cell genes [41]. Our findings, combined with previous evidence, suggest that those infection-related genes, such as polysaccharide deacetylase, spore wall protein, polar tube protein, and Ricin B lectin, are vital during microsporidia infection.

The comparative transcriptomics analysis revealed the DEGs of silkworms between the embryos and larvae during infection. The large number of genes associated with DNA replication and repair, including DNA replication, fanconi anemia pathway, mismatch repair, mRNA surveillance pathway, nucleotide excision repair, homologous recombination, and non-homologous end-joining, were down-regulated in larvae during *N. bombycis* congenital infection, suggesting that *N. bombycis* infection may cause major damage to the larvae and even death, but only minor damage to the embryos. At least two different mechanisms can explain such differences in virulence between embryos and larvae. First, the number of *N. bombycis* (parasite load) in embryos is less than that in larvae, and the virulence was weaker than that in larvae [42]. Second, *N. bombycis* proliferated mainly around yolk granules and the newly formed intestinal lumen in the embryonic stage [5], whereas *N. bombycis* proliferated in all types of tissues in the larvae stage. Microsporidia may use these strategies to develop vertical transmission in embryos.

We have recently reported that *N. bombycis* infection leads to host embryo immunosuppression [15]. In the early stage of congenital infection in larvae, beyond DNA replication and repair, mRNA surveillance pathway, RNA transport, protein biosynthesis, and protein export genes, the expression of genes related to phagocytosis, apoptosis, and TNF signaling pathway were different from those in embryos, suggesting that cellular immunity plays an important role in the fight against microsporidia infection; in the middle and late stages of congenital infection in larvae, some genes related to infection and immune pathways, including Toll-like receptor signaling pathway, NF-kappa B signaling pathway and human diseases, were also differentially expressed in embryos and larvae during infection of *N. bombycis*, indicating that humoral immunity plays a major role during infection. We also found that the majority of those genes were down-regulated in larvae, especially 10-day larvae, indicating that *N. bombycis* was able to inhibit host immune responses. The inhibition was stronger with increasing parasite loads in the later stages of infection. Interestingly, the RhoGAP protein, histamine-releasing factor, and elongation factor 1 alpha, which have been described as immune suppressive factors in other parasite species [43, 44], have not yet been identified in microsporidian.

Previous study has shown that microsporidia can hijack the host’s basic metabolisms for their growth and reproduction [45]. For example, Tang et al., have reported that most proteins involved in basic metabolism and lipid droplet protein pelilipin were up-regulated in ovaries following infection [46]. Hu et al., found that most silkworm metabolism-related genes, especially those involved in fatty acid consumption and ATP synthesis pathways, were up-regulated [47]. We also found that *N. bombycis* infection leads to metabolism dysregulation in embryos. In 1-day larvae, several DEGs were associated with insulin signaling pathways and insulin resistance, suggesting that glucose and lipid metabolisms were involved in the process of *N. bombycis* infection. In 5-day larvae, several DEGs were involved in 2-oxocarboxylic acid metabolism, and phenylalanine, tyrosine, and tryptophan biosynthesis were up-regulated compared to those in embryos during infection, suggesting that amino acid metabolisms play an important role in the microsporidia infection. Importantly, many genes involved in arachidonic acid metabolism, linoleic acid metabolism, fat digestion and absorption, ether lipid metabolism, alpha-linoleic acid metabolism, and glycerophospholipid metabolism were found to be up-regulated mostly in 10-day larvae. In contrast, most lipid-related genes in *N. bombycis*, such as *SPT1*, *TSC10*, *PIS1*, *GDPD2*, and *G3PD*, were down-regulated in larvae during *N. bombycis* infection. These findings suggest that microsporidia can hijack host basic metabolism, especially host amino acid and lipid metabolisms, to facilitate their proliferation and development.

Lastly, we compared host-specific and common DEGs in embryos and larvae during *N. bombycis* infection, and found that several genes that are involved in the epigenetic regulation mechanism, such as histone-lysine-N-methyltransferase EEF2KMT, histone-lysine-N-methyltransferase SETD1B, histone-lysine-N-methyltransferase E(z), polycomb protein Pcl, lysine-specific histone demethylase 1A, histone deacetylase complex subunit SAP30, and deacetylase complex subunit SAP18, were most significantly down-regulated in larvae. DNA methylation and post-translational histone modifications are two major types of epigenetic modifications that regulate gene expression by modifying chromatin accessibility to transcriptional regulators [48]. Epigenetic modification plays an important role in the survival of intracellular parasites, including *Plasmodium falciparum*, *leishmania*, and *Toxoplasma gondii* [49–52]. Meanwhile, intracellular parasites also modulate host epigenome by histone acetylation, histone deacetylation, histone methylation, and DNA methylation to evade host immunity and maintain long-term persistence in the host [53–56]. Our findings provide additional evidence that the interactions between microsporidia and their hosts in embryos and larvae depend heavily on epigenetic modifications.

**Fig. 9:**
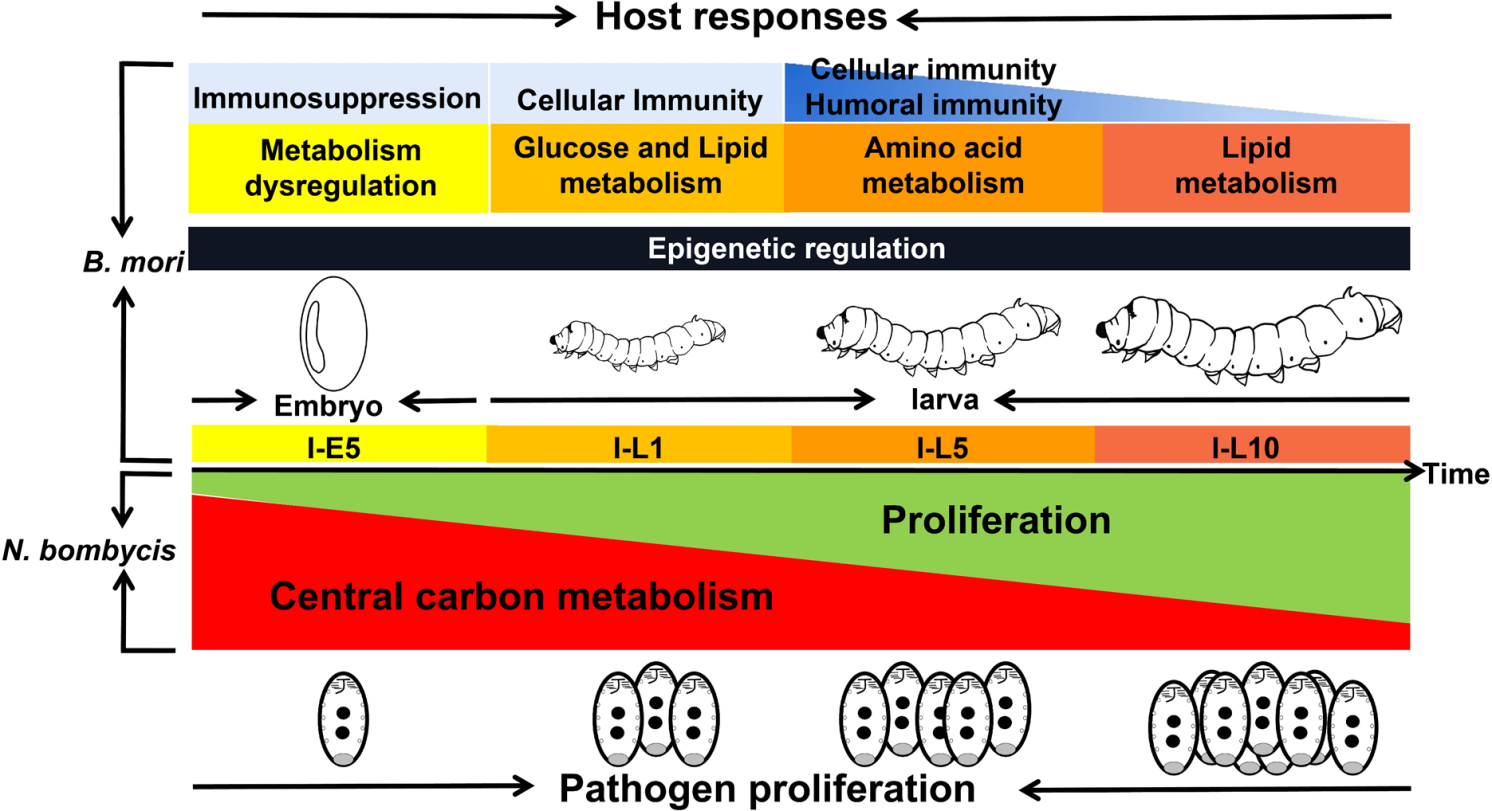
Schematic diagram showing the changes in the pathogen proliferation and host responses in congenitally infected embryos and larvae. In the larvae infected with *N. bombycis*, parasite genes related to parasite proliferation and growth were up-regulated, whereas genes related to central carbon metabolism were down-regulated compared to those expressions in embryos. As the infection progresses in embryos and larvae, host responses including immunity and metabolisms varied. Epigenetic modification was likely to participate in the pathogen-host interactions

Overall, our study can help researchers understand how the pathogen and host genes are expressed differently in *N. bombycis* congenitally infected embryos and larvae (Fig. 9). The results of this study may also shed light on how *N. bombycis* and silkworms interact. However, more research is needed to confirm the significance of carbon, amino acid, and lipid metabolisms on microsporidia proliferation and development. Notably, our work has significant implications for our understanding of how epigenetic regulators play a role in the interactions between microsporidia and hosts.

### Supplementary Information

**Supplementary Material 1.**

**Supplementary Material 2.**

## Data Availability

The raw data generated was submitted to the National Center for Biotechnology Information (NCBI) Sequence Read Archive (SRA, http://www.ncbi.nlm.nih.gov/sra) with the BioProject accession number (PRJNA953616).

## Abbreviations

DEGs: Differentially expressed genes
FPKM: Fragments per kilobase of transcript per million fragments mapped
KEGG: Kyoto encyclopedia of genes and genomes
NCBI: National center for biotechnology information
SRA: Sequence read archive
GO: Gene ontology
MAPK: Mitogen-activated protein kinase
RT-qPCR: Quantitative reverse transcription polymerase chain reaction
SPT1: Serine palmitoyltransferase 1
TSC10: Temperature-sensitive csg2Δ suppressor 10
PIS1: Phosphatidylinositol synthase 1
GDPD2: Glycerophosphodiester phosphodiesterase 2
G3PD: Glyceraldehyde-3-phosphate dehydrogenase
GPD1: Glycerol 3-phosphate dehydrogenase 1
ACAT1: Acetoacetyl-CoA thiolase 1
SLC27A4: Solute carrier family 27 member 4
AGPAT1: 1-acylglycerol-3-phosphate O-acyltransferase 1
GGT1: Gamma-glutamyltranspeptidase 1
HMGCS1: 3-hydroxy-3-methylglutaryl-CoA synthase 1
SCARB2: Scavenger receptor class B member 2
SOCS: Suppressor of cytokine signaling
IBP2: Insulin-related peptide binding protein 2

## References

[1] James TY, Pelin A, Bonen L, Ahrendt S, Sain D, Corradi N, Stajich JE (2013). Shared signatures of parasitism and phylogenomics unite Cryptomycota and microsporidia. Curr Biol.

[2] Vavra J, Lukes J (2013). Microsporidia and 'the art of living together'. Adv Parasitol.

[3] Fu Z, He X, Cai S, Liu H, He X, Li M, Lu X (2016). Quantitative PCR for detection of Nosema bombycis in single silkworm eggs and newly hatched larvae. J Microbiol Methods.

[4] Ma Z, Li C, Pan G, Li Z, Han B, Xu J, Lan X, Chen J, Yang D, Chen Q, Sang Q, Ji X, Li T, Long M, Zhou Z (2013). Genome-wide transcriptional response of silkworm (Bombyx mori) to infection by the microsporidian Nosema bombycis. PLoS One.

[5] Song Y, Tang Y, Yang Q, Li T, He Z, Wu Y, He Q, Li T, Li C, Long M, Chen J, Wei J, Bao J, Shen Z, Meng X, Pan G, Zhou Z (2020). Proliferation characteristics of the intracellular microsporidian pathogen Nosema bombycis in congenitally infected embryos. J Invertebr Pathol.

[6] Naidoo S, Visser EA, Zwart L, Toit YD, Bhadauria V, Shuey LS (2018). Dual RNA-sequencing to elucidate the plant-pathogen duel. Curr Issues Mol Biol.

[7] Westermann AJ, Barquist L, Vogel J (2017). Resolving host-pathogen interactions by dual RNA-seq. PLoS Pathog.

[8] Fronicke L, Bronner DN, Byndloss MX, McLaughlin B, Baumler AJ, Westermann AJ (2018). Toward cell type-specific in vivo dual RNA-Seq. Methods Enzymol.

[9] Burgess DJ (2017). Gene expression: host-pathogen duels revealed by dual RNA-seq in vivo. Nat Rev Genet.

[10] Aoki JI, Muxel SM, Laranjeira-Silva MF, Zampieri RA, Muller KE, Nerland AH, et al. Dual transcriptome analysis reveals differential gene expression modulation influenced by Leishmania arginase and host genetic background. Microb Genom. 2020;6(9). 10.1099/mgen.0.000427 PMC7643972 32886592

[11] Fernandes MC, Dillon LA, Belew AT, Bravo HC, Mosser DM, El-Sayed NM. Dual transcriptome profiling of Leishmania-infected human macrophages reveals distinct reprogramming signatures. mBio. 2016;7(3). 10.1128/mBio.00027-16 PMC4959658 27165796

[12] Pittman KJ, Aliota MT, Knoll LJ (2014). Dual transcriptional profiling of mice and toxoplasma gondii during acute and chronic infection. BMC Genomics.

[13] Ehret T, Spork S, Dieterich C, Lucius R, Heitlinger E (2017). Dual RNA-seq reveals no plastic transcriptional response of the coccidian parasite Eimeria falciformis to host immune defenses. BMC Genom.

[14] Westermann AJ, Forstner KU, Amman F, Barquist L, Chao Y, Schulte LN, Muller L, Reinhardt R, Stadler PF, Vogel J (2016). Dual RNA-seq unveils noncoding RNA functions in host-pathogen interactions. Nature..

[15] Shen Z, Yang Q, Luo L, Li T, Ke Z, Li T, Chen J, Meng X, Xiang H, Li C, Zhou Z, Chen P, Pan G (2023). Non-coding RNAs identification and regulatory networks in pathogen-host interaction in the microsporidia congenital infection. BMC Genom.

[16] Miller JD, Neely MN (2004). Zebrafish as a model host for streptococcal pathogenesis. Acta Trop.

[17] Saralahti A, Piippo H, Parikka M, Henriques-Normark B, Ramet M, Rounioja S (2014). Adult zebrafish model for pneumococcal pathogenesis. Dev Comp Immunol.

[18] Meijer AH, Spaink HP (2011). Host-pathogen interactions made transparent with the zebrafish model. Curr Drug Targets.

[19] Benard EL, Rougeot J, Racz PI, Spaink HP, Meijer AH (2016). Transcriptomic approaches in the zebrafish model for tuberculosis-insights into host- and pathogen-specific determinants of the innate immune response. Adv Genet.

[20] Tabunoki H, Bono H, Ito K, Yokoyama T (2016). Can the silkworm (Bombyx mori) be used as a human disease model?. Drug Discov Ther..

[21] Rosenberg A, Sibley LD (2022). Epigenetic modifiers Alter host cell transcription to promote toxoplasma infection. ACS Infect Dis..

[22] Lecoeur H, Prina E, Rosazza T, Kokou K, N'Diaye P, Aulner N, Varet H, Bussotti G, Xing Y, Milon G, Weil R, Meng G, Spath G (2020). Targeting macrophage histone H3 modification as a Leishmania strategy to dampen the NF-kappaB/NLRP3-mediated inflammatory response. Cell Rep.

[23] Serrano-Duran R, Lopez-Farfan D, Gomez-Diaz E. Epigenetic and Epitranscriptomic gene regulation in plasmodium falciparum and how we can use it against malaria. Genes. 2022;13(10). 10.3390/genes13101734 PMC9601349 36292619

[24] Supek F, Bosnjak M, Skunca N, Smuc T (2011). REVIGO summarizes and visualizes long lists of gene ontology terms. PLoS One.

[25] Kanehisa M, Goto S (2000). KEGG: Kyoto encyclopedia of genes and genomes. Nucleic Acids Res.

[26] Kanehisa M (2019). Toward understanding the origin and evolution of cellular organisms. Protein Sci : Publ Protein Soc.

[27] Kanehisa M, Furumichi M, Sato Y, Kawashima M, Ishiguro-Watanabe M (2023). KEGG for taxonomy-based analysis of pathways and genomes. Nucleic Acids Res.

[28] Chen C, Chen H, Zhang Y, Thomas HR, Frank MH, He Y, Xia R (2020). TBtools: an integrative toolkit developed for interactive analyses of big biological data. Mol Plant.

[29] Sun Y, Liu WZ, Liu T, Feng X, Yang N, Zhou HF (2015). Signaling pathway of MAPK/ERK in cell proliferation, differentiation, migration, senescence and apoptosis. J Recept Signal Transduct Res.

[30] Wei S, Daniel BJ, Brumlik MJ, Burow ME, Zou W, Khan IA, Wadsworth S, Siekierka J, Curiel TJ (2007). Drugs designed to inhibit human p38 mitogen-activated protein kinase activation treat toxoplasma gondii and Encephalitozoon cuniculi infection. Antimicrob Agents Chemother.

[31] He Q, Luo J, Xu JZ, Wang CX, Meng XZ, Pan GQ, et al. Morphology and transcriptome analysis of nosema bombycis sporoplasm and insights into the initial infection of microsporidia. mSphere. 2020;5(1). 10.1128/mSphere.00958-19 PMC7021473 32051240

[32] Li Y, Niu Z, Yang J, Yang X, Chen Y, Li Y, Liang X, Zhang J, Fan F, Wu P, Peng C, Shen B (2023). Rapid metabolic reprogramming mediated by the AMP-activated protein kinase during the lytic cycle of toxoplasma gondii. Nat Commun.

[33] Smith TK, Bringaud F, Nolan DP, Figueiredo LM. Metabolic reprogramming during the Trypanosoma brucei life cycle. F1000Research. 2017;6. 10.12688/f1000research.10342.2 PMC5461901 28620452

[34] Zhao Y, Park RD, Muzzarelli RA (2010). Chitin deacetylases: properties and applications. Mar Drugs.

[35] Balomenou S, Fouet A, Tzanodaskalaki M, Couture-Tosi E, Bouriotis V, Boneca IG (2013). Distinct functions of polysaccharide deacetylases in cell shape, neutral polysaccharide synthesis and virulence of bacillus anthracis. Mol Microbiol.

[36] Xu Q, Wang J, Zhao J, Xu J, Sun S, Zhang H, Wu J, Tang C, Kang Z, Wang X (2020). A polysaccharide deacetylase from Puccinia striiformis f. sp. tritici is an important pathogenicity gene that suppresses plant immunity. Plant Biotechnol J.

[37] Jagadish A, Dubey H, Kamatchi I, Pradeep AR, Subrahmanyam G, Mishra RK, Ponnuvel KM (2021). Transcriptome analysis of Nosema assamensis infecting muga silkworms (Antheraea assamensis) reveals insights into candidate pathogenicity related genes and molecular pathways required for pathogenesis. Ann Parasitol.

[38] Li Z, Pan G, Li T, Huang W, Chen J, Geng L, Yang D, Wang L, Zhou Z (2012). SWP5, a spore wall protein, interacts with polar tube proteins in the parasitic microsporidian Nosema bombycis. Eukaryot Cell.

[39] Liu H, Li MQ, Cai SF, He XY, Shao YQ, Lu XM (2016). Ricin-B-lectin enhances microsporidia Nosema bombycis infection in BmN cells from silkworm Bombyx mori. Acta Biochim Biophys Sin.

[40] Prybylski N, Fayet M, Dubuffet A, Delbac F, Kocer A, Gardarin C, et al. Ricin B lectin-like proteins of the microsporidian Encephalitozoon cuniculi and Anncaliia algerae are involved in host-cell invasion. Parasitol Int. 2022;87. 10.1016/j.parint.2021.102518 34808329

[41] Xu JZ, Luo J, Chen JJ, Vossbrinck CR, Li T, Zhou ZY. Characterization of the largest secretory protein family, ricin B lectin-like protein, in Nosema bombycis: insights into microsporidian adaptation to host. J Fungi. 2022;8(6). 10.3390/jof8060551 PMC9224602 35736035

[42] Terry RS, Smith JE, Sharpe RG, Rigaud T, Littlewood DT, Ironside JE, Rollinson D, Bouchon D, MacNeil C, Dick JT, Dunn AM (2004). Widespread vertical transmission and associated host sex-ratio distortion within the eukaryotic phylum Microspora. Proc Biol Sci.

[43] Demarta-Gatsi C, Rivkin A, Di Bartolo V, Peronet R, Ding S, Commere PH, Guillonneau F, Bellalou J, Brule S, Abou Karam P, Cohen SR, Lagache T, Janse C, Regev-Rudzki N, Mecheri S (2019). Histamine releasing factor and elongation factor 1 alpha secreted via malaria parasites extracellular vesicles promote immune evasion by inhibiting specific T cell responses. Cell Microbiol.

[44] Labrosse C, Stasiak K, Lesobre J, Grangeia A, Huguet E, Drezen JM, Poirie M (2005). A RhoGAP protein as a main immune suppressive factor in the Leptopilina boulardi (Hymenoptera, Figitidae)-Drosophila melanogaster interaction. Insect Biochem Mol Biol.

[45] Pan G, Bao J, Ma Z, Song Y, Han B, Ran M, Li C, Zhou Z (2018). Invertebrate host responses to microsporidia infections. Dev Comp Immunol.

[46] Tang X, Zhang Y, Zhou Y, Liu R, Shen Z (2020). Quantitative proteomic analysis of ovaries from Nosema bombycis-infected silkworm (Bombyx mori). J Invertebr Pathol.

[47] Hu N, Dong ZQ, Long JQ, Zheng N, Hu CW, Wu Q, Chen P, Lu C, Pan MH (2021). Transcriptome analysis reveals changes in silkworm energy metabolism during Nosema bombycis infection. Pestic Biochem Physiol.

[48] Klemm SL, Shipony Z, Greenleaf WJ (2019). Chromatin accessibility and the regulatory epigenome. Nat Rev Genet.

[49] Duffy MF, Selvarajah SA, Josling GA, Petter M (2014). Epigenetic regulation of the plasmodium falciparum genome. Brief Funct Genom.

[50] McDonald JR, Jensen BC, Sur A, Wong ILK, Beverley SM, Myler PJ. Localization of epigenetic markers in Leishmania chromatin. Pathogens. 2022;11(8). 10.3390/pathogens11080930 PMC9413968 36015053

[51] Dixon SE, Stilger KL, Elias EV, Naguleswaran A, Sullivan WJ (2010). A decade of epigenetic research in toxoplasma gondii. Mol Biochem Parasitol.

[52] Ponts N, Fu L, Harris EY, Zhang J, Chung DW, Cervantes MC, Prudhomme J, Atanasova-Penichon V, Zehraoui E, Bunnik EM, Rodrigues EM, Lonardi S, Hicks GR, Wang Y, Le Roch KG (2013). Genome-wide mapping of DNA methylation in the human malaria parasite plasmodium falciparum. Cell Host Microbe.

[53] Hakimi MA (2022). Epigenetic reprogramming in host-parasite coevolution: the toxoplasma paradigm. Ann Rev Microbiol.

[54] Afrin F, Khan I, Hemeg HA (2019). Leishmania-host interactions-an epigenetic paradigm. Front Immunol.

[55] Marr AK, MacIsaac JL, Jiang R, Airo AM, Kobor MS, McMaster WR (2014). Leishmania donovani infection causes distinct epigenetic DNA methylation changes in host macrophages. PLoS Pathog.

[56] Parmar N, Chandrakar P, Kar S (2020). Leishmania donovani subverts host immune response by epigenetic reprogramming of macrophage M(lipopolysaccharides + IFN-gamma)/M(IL-10) polarization. J Immunol.

