# Supplementary material for: Transcriptomic changes in the microsporidia proliferation and host responses in congenitally infected embryos and larvae
Source: BMC Genomics. 2024 Apr 1;25:321. doi: 10.1186/s12864-024-10236-y (PMC10983672; doi:10.1186/s12864-024-10236-y)

sFig. 1 Venn diagrams showing the number of a subset of exclusive and shared genes expressed by silkworm between non-infected or infected embryos and larvae. A: Venn diagrams showing the number of a subset of exclusive and shared genes expressed by silkworm between non-infected or infected 5-day embryos and 1-day larvae; B: Venn diagrams showing the number of a subset of exclusive and shared genes expressed by silkworm between non-infected or infected 5-day embryos and 5-day larvae; C: Venn diagrams showing the number of a subset of exclusive and shared genes expressed by silkworm between non-infected or infected 5-day embryos and 10-day larvae.


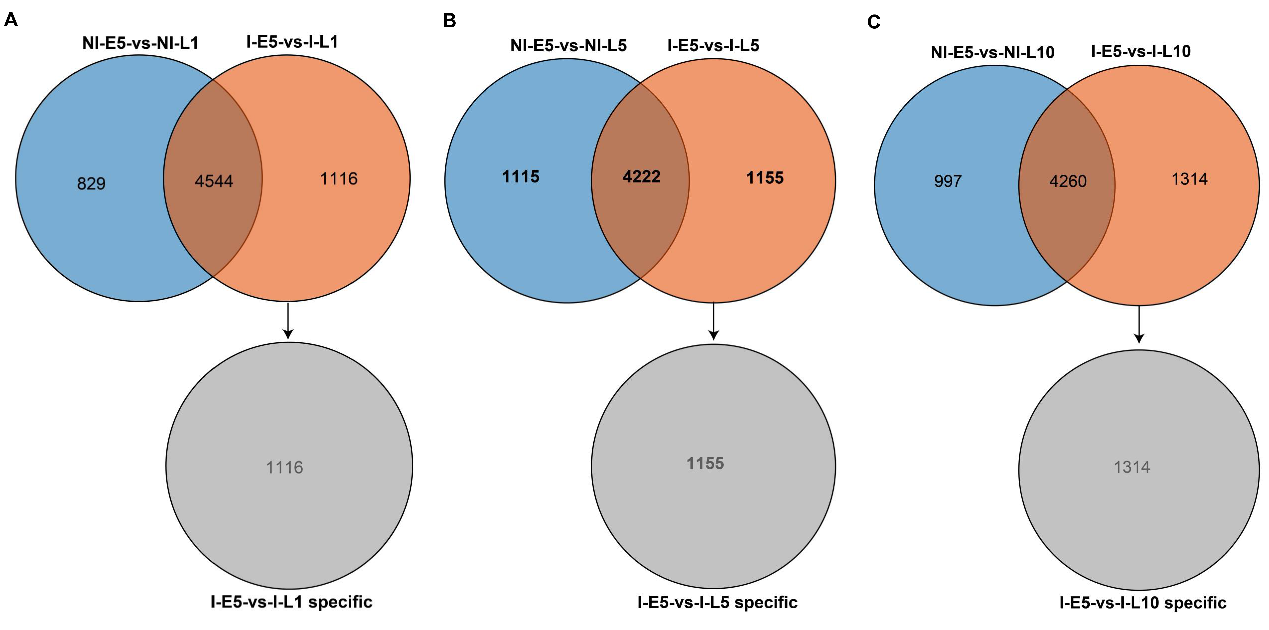


sFig. 2 Subset of the carbon metabolism, biosynthesis of amino acids, and sphingolipid metabolism pathway in *N. bombycis* between embryos and larvae. A: Pentose phosphate pathway; B: Fructose and mannose metabolism; C: Starch and sucrose metabolism; D: Sphingolipid metabolism; E: Glycolysis/gluconeogenesis; F: Biosynthesis of amino acids. A red font represents the genes that were up-regulated, and a green font represents the genes that were down-regulated in larvae compared to those in embryos during *N. bombycis* infection.


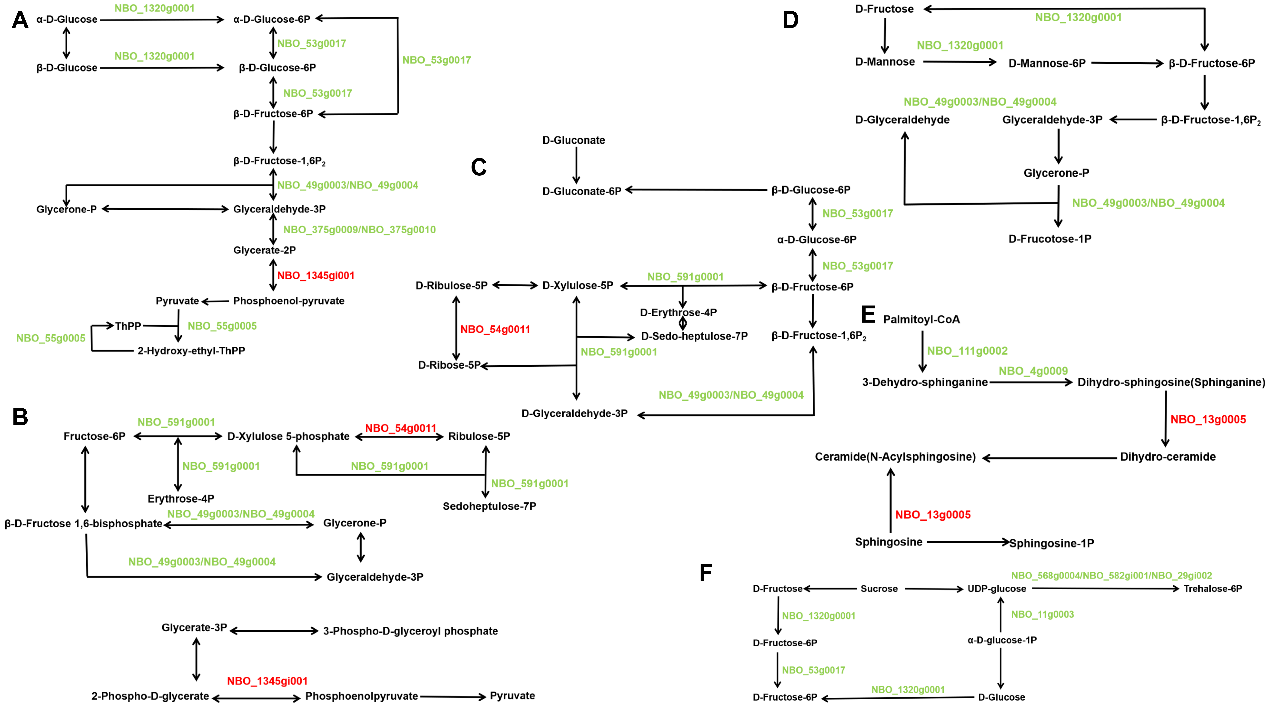


sFig. 3 Subset of the arachidonic acid metabolism, linoleic acid metabolism, and fat digestion and absorption in silkworm between embryos and larvae during *N. bombycis* infection. A: Arachidonic acid metabolism; B: Linoleic acid metabolism; C: Fat digestion and absorption. A red font represents the genes that were up-regulated, and a green font represents the genes that were down-regulated in larvae compared to those in embryos during *N. bombycis* infection.


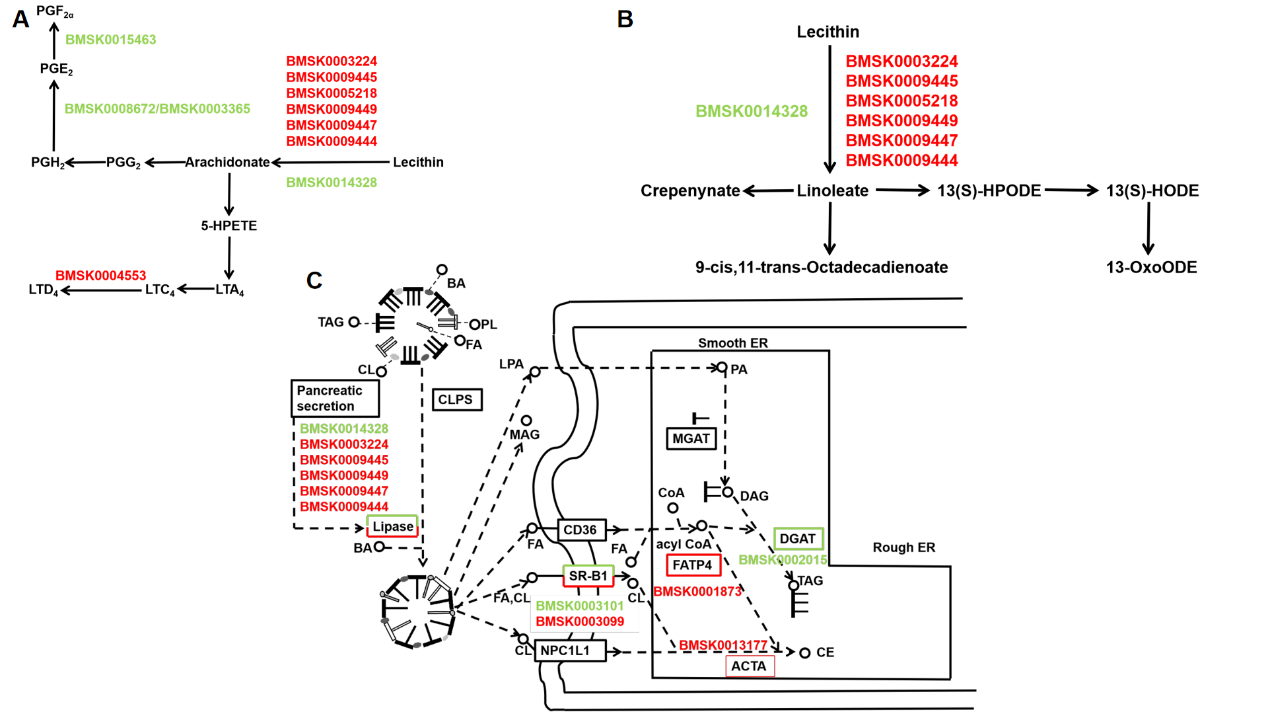

Supplement: Supplementary file 1 — Supplementary Material 1. [file 12864_2024_10236_MOESM1_ESM.docx]
