# Supplementary material for: Transcriptomic changes in the microsporidia proliferation and host responses in congenitally infected embryos and larvae
Source: BMC Genomics. 2024 Apr 1;25:321. doi: 10.1186/s12864-024-10236-y (PMC10983672; doi:10.1186/s12864-024-10236-y)
Supplement: Supplementary file 2 — Supplementary Material 2. [file 12864_2024_10236_MOESM2_ESM.docx]

# Supplementary Table 1. The list of primer's names and their sequences

| Primers | Sequence |
| --- | --- |
| SPT1  TSC10  GPD1  PIS1  GDPD2  G3PD  ACAT1 | 5’- TGACCCAATTGAATCTGCCA-3’ and 5’-TAGGACGGTAGGAGGAAGGG-3’  5’- TCGGTGGAAGTAGTGGATTAGG-3’ and 5’-CGTCACACACATCCAGAACC-3’  5’- GGAACAGGAAATTGGGGAACA-3’ and 5’-TCAGGGAGTCGGATACCACT-3’  5’- TGGTGGTTGCCTGGATATGA-3’ and 5’-CACTCATTGTCATTATCGCGGA-3’  5’- GGTAAGGGAGCTGAATGGGT-3’ and 5’-TCGTCTTCAGGTCGATCGAT-3’  5’- GCACAAAGTTCGGGTACACA-3’ and 5’-CGGCTTGATTGGGTTCTACG-3’  5’- ATGGCAGCATTCTCGACCAA-3’ and 5’-TTCGATTGCCGCGTTAACAG-3 |
| SLC27A4 | 5’-TTCGGATGGAGTGTCTTGGG-3’ and 5’-AGATGATAAGACTGGTGAGCCA-3’ |
| HMGCS1  AGPAT1  GGT1 | 5’- TTCCCGTCGCAATACGTTGA-3’ and 5’-TGTGCAAGGCTGTGAGACAA-3’  5’- TCATGAGCAGACCCAGTGTT-3’ and 5’- GCCGCCATTTTCGAATACCT-3’  5’- GGAATCAGCAGAGCATGGGT-3’ and 5’-CAACGGCTAGCGGACCTTTA-3’ |
| SCARB2 | 5’- AGATCTGGACGAAGAAGCCG-3’ and 5’-CAGATTGGGCACAGTGACGA-3’ |

**Supplementary Table 2 Differentially expressed genes of *N. bombycis* between embryos and larvae during *N. bombycis* infection**

| **Differentially expressed genes of *N. bombycis* between 5-day embryos and 1-day larvae** **during *N. bombycis* infection** | | | | |
| --- | --- | --- | --- | --- |
| ID | I-E5_mean | I-L1_mean | log2(fc) | Description |
| NBO_1078g0004 | 477.0333333 | 237.8366667 | -1.004118931 | Zinc finger C2H2 protein [Nosema bombycis CQ1] |
| NBO_401g0005 | 90.40333333 | 27.92 | -1.695077027 | YjeF family domain-containing protein [Anncaliia algerae PRA339] |
| NBO_64g0032 | 29.79333333 | 12.96666667 | -1.20018189 | YjeF family domain-containing protein [Anncaliia algerae PRA339] |
| NBO_11g0046 | 151.8933333 | 32.27 | -2.234793068 | Xaa-Pro aminopeptidase 1 [Nosema bombycis CQ1] |
| NBO_19g0026 | 82.78 | 171.5433333 | 1.051218906 | U3 small nucleolar ribonucleoprotein imp4 [Nosema bombycis CQ1] |
| NBO_28g0074 | 71.84333333 | 161.1633333 | 1.165597356 | u3 small nucleolar ribonucleoprotein component mpp10 [Nosema ceranae] |
| NBO_76g0012 | 35.91666667 | 76.66 | 1.093820535 | type 2 peptidyl-tRNA hydrolase [Encephalitozoon romaleae SJ-2008] |
| NBO_696g0002 | 40.86 | 90 | 1.139235797 | Tryptophanyl-tRNA synthetase [Nosema bombycis CQ1] |
| NBO_64gi001 | 30.84666667 | 70.17333333 | 1.185808296 | tRNA adenilyl transferase [Nosema bombycis CQ1] |
| NBO_29g0027 | 947.33 | 448.4 | -1.079080794 | trehalose-phosphatase, partial [Nosema bombycis CQ1] |
| MSTRG.8023 | 122.4066667 | 53.63333333 | -1.190480309 | trans-sialidase [Cordyceps brongniartii RCEF 3172] |
| NBO_591g0001 | 1609.136667 | 713.2466667 | -1.173813857 | Transketolase 1, partial [Nosema bombycis CQ1] |
| NBO_401g0008 | 82.34 | 201.42 | 1.290541589 | transcription elongation factor s-ii [Nosema ceranae] |
| NBO_64g0035 | 47.17666667 | 117.6166667 | 1.317947118 | transcription elongation factor s-ii [Nosema ceranae] |
| NBO_365g0001 | 167.01 | 70.61333333 | -1.241921963 | Threonyl-tRNA synthetase, partial [Nosema bombycis CQ1] |
| MSTRG.2169 | 141.0033333 | 42.37333333 | -1.734500739 | threonyl-tRNA synthetase [Nosema bombycis CQ1] |
| NBO_32gi001 | 1041.966667 | 450.0666667 | -1.211098502 | threonyl-tRNA synthetase [Nosema bombycis CQ1] |
| NBO_1332gi002 | 94.37666667 | 24.14333333 | -1.966805343 | threonyl-tRNA synthetase [Nosema bombycis CQ1] |
| NBO_76gi004 | 162.7433333 | 69.16 | -1.234588672 | threonyl-tRNA synthetase [Nosema bombycis CQ1] |
| MSTRG.831 | 93.93666667 | 40.79666667 | -1.20323712 | threonyl-tRNA synthetase [Nosema bombycis CQ1] |
| NBO_1157g0003 | 48.59666667 | 101.5666667 | 1.063497734 | syntaxin-like protein [Nosema ceranae] |
| NBO_552g0005 | 20652.47 | 8376.813333 | -1.301840905 | surface-antigen protein P30.4, partial [Nosema bombycis] |
| NBO_552g0002 | 5271.623333 | 2189.13 | -1.26788966 | spore wall protein 5 [Nosema bombycis] |
| NBO_27g0035 | 5161.3 | 2108.026667 | -1.291841372 | spore wall protein 5 [Nosema bombycis] |
| NBO_10g0028 | 37.77666667 | 76.16666667 | 1.011664352 | Spliceosome-associated protein 145 [Nosema bombycis CQ1] |
| NBO_417g0011 | 34.39666667 | 11.31 | -1.604669833 | Solute carrier family 35 member C2, partial [Nosema bombycis CQ1] |
| NBO_29g0031 | 848.5766667 | 418.15 | -1.021024446 | Solute carrier family 2, facilitated glucose transporter member 3 [Nosema bombycis CQ1] |
| NBO_85g0011 | 21.04 | 5.313333333 | -1.985445576 | Solute carrier family 2, facilitated glucose transporter member 2 [Nosema bombycis CQ1] |
| NBO_63g0025 | 12.87 | 36.61 | 1.50822572 | Serine/threonine-protein kinase ppk23 [Nosema bombycis CQ1] |
| NBO_111g0002 | 66.74333333 | 31.11666667 | -1.100936218 | Serine palmitoyltransferase 1 [Nosema bombycis CQ1] |
| NBO_2g0058 | 6.366666667 | 1.23 | -2.371879917 | RpeA, partial [Nosema bombycis CQ1] |
| NBO_42g0007 | 52.76 | 110.5866667 | 1.067660982 | rna polymerase ii ctd phosphatase [Nosema ceranae] |
| NBO_169g0003 | 68.69 | 141.14 | 1.038954926 | ring finger protein [Nosema bombycis CQ1] |
| NBO_1135g0001 | 1325.543333 | 600.93 | -1.141314982 | ricin b lectin [Nosema ceranae] |
| NBO_1133g0001 | 148.5533333 | 64.18666667 | -1.210635431 | Ricin B lectin [Nosema bombycis CQ1] |
| NBO_6g0048 | 21.12333333 | 73.46333333 | 1.798186844 | Ricin B lectin [Nosema bombycis CQ1] |
| NBO_6g0062 | 11.30666667 | 1.92 | -2.557995453 | Ricin B lectin [Nosema bombycis CQ1] |
| NBO_6g0049 | 2.603333333 | 19.18666667 | 2.881672139 | Ricin B lectin [Nosema bombycis CQ1] |
| NBO_740g0001 | 20.40666667 | 53.20333333 | 1.382476093 | ribosomal protein l12e-l44-l45-rpp1-rpp2 [Nosema apis BRL 01] |
| MSTRG.466 | 20275.41667 | 9467.443333 | -1.098684777 | RecName: Full=Spore wall protein 25; Flags: Precursor |
| NBO_940g0001 | 6761.953333 | 3204.433333 | -1.077370804 | RecName: Full=Spore wall protein 25; Flags: Precursor |
| NBO_12g0010 | 44.51666667 | 94.24333333 | 1.082044997 | pseudouridylate synthase [Nosema ceranae] |
| NBO_1000gi001 | 73.97 | 22.76333333 | -1.700228443 | Protein PNS1, partial [Nosema bombycis CQ1] |
| NBO_33gi001 | 59.04666667 | 20.26333333 | -1.5429841 | Protein PNS1, partial [Nosema bombycis CQ1] |
| NBO_350g0001 | 7.58 | 1.443333333 | -2.392793324 | Protein PNS1, partial [Nosema bombycis CQ1] |
| NBO_447g0003 | 384.6233333 | 134.8566667 | -1.512019444 | Protein of unknown function GLTT, partial [Nosema bombycis CQ1] |
| NBO_974g0001 | 3.43 | 15.22333333 | 2.150003807 | Polysaccharide deacetylase [Nosema bombycis CQ1] |
| NBO_4g0029 | 4325.663333 | 1946.84 | -1.151787063 | polar tube protein 3 [Nosema bombycis] |
| NBO_10g0052 | 3559.52 | 1645.906667 | -1.112800179 | Polar tube protein 3 [Nosema bombycis CQ1] |
| NBO_7g0015 | 5144.466667 | 2076.15 | -1.309110838 | polar tube protein 2 [Nosema bombycis] |
| NBO_943g0003 | 3005.176667 | 1277.51 | -1.234115222 | Polar tube protein 2 [Nosema bombycis CQ1] |
| NBO_7g0016 | 5794.833333 | 2505.716667 | -1.209543877 | Polar tube protein 1 [Nosema bombycis CQ1] |
| NBO_943g0004 | 8215.633333 | 3711.356667 | -1.146425142 | Polar tube protein 1 [Nosema bombycis CQ1] |
| MSTRG.5652 | 13.15666667 | 89.50333333 | 2.766147395 | pol polyprotein, partial [Nosema apis BRL 01] |
| MSTRG.5960 | 5.983333333 | 29.59666667 | 2.306413358 | pol polyprotein, partial [Nosema apis BRL 01] |
| MSTRG.3956 | 164.9366667 | 74.26 | -1.151254936 | pol polyprotein [Nosema apis BRL 01] |
| NBO_198gi002 | 11.74333333 | 31.31666667 | 1.415088687 | phosphate-transporting ATPase [Trachipleistophora hominis] |
| NBO_1010g0001 | 23.02333333 | 49.31 | 1.09878353 | Pescadillo-like protein [Nosema bombycis CQ1] |
| NBO_20g0002 | 25.75666667 | 55.51333333 | 1.107888426 | Pescadillo like protein [Nosema bombycis CQ1] |
| NBO_20g0008 | 10.91666667 | 26.40333333 | 1.274187671 | peptide chain release factor 2 [Nosema ceranae] |
| NBO_28g0052 | 19.09333333 | 49.81666667 | 1.383559499 | Nucleolar essential protein 1 [Nosema bombycis CQ1] |
| NBO_443g0001 | 39.45666667 | 88.08333333 | 1.158599985 | nucleic acid-binding protein [Nosema ceranae] |
| NBO_1053g0002 | 1241.056667 | 588.8533333 | -1.075588741 | Nuclear pore complex protein Nup98-Nup96 [Nosema bombycis CQ1] |
| NBO_29g0030 | 1241.526667 | 567.6033333 | -1.129160282 | Nuclear pore complex protein Nup98-Nup96 [Nosema bombycis CQ1] |
| NBO_925g0001 | 12.39666667 | 38.43666667 | 1.63253098 | Myosin heavy chain kinase B [Nosema bombycis CQ1] |
| NBO_920g0001 | 8.103333333 | 22.58666667 | 1.478883981 | Myosin heavy chain kinase B [Nosema bombycis CQ1] |
| NBO_55g0005 | 183.0933333 | 88.19666667 | -1.053783225 | mitochondrial pyruvate dehydrogenase E1 component subunit alpha [Nosema bombycis] |
| NBO_34g0010 | 1563.37 | 5024.006667 | 1.68417912 | mitochondrial protein import protein MAS5 [Nosema pernyi] |
| NBO_6g0037 | 55.37666667 | 123.2466667 | 1.154198508 | Meiotic nuclear division protein 1 [Nosema bombycis CQ1] |
| NBO_59g0003 | 40.51666667 | 86.16 | 1.088502762 | Large subunit GTPase 1 [Nosema bombycis CQ1] |
| NBO_37g0007 | 32.32666667 | 15.50666667 | -1.059836157 | KH domain-containing protein [Nosema bombycis CQ1] |
| NBO_1608g0001 | 28.19666667 | 70.77333333 | 1.327681249 | Isoleucyl-tRNA synthetase, cytoplasmic [Nosema bombycis CQ1] |
| NBO_423g0006 | 9.333333333 | 32.51 | 1.80041923 | Iron-sulfur clusters transporter atm1, mitochondrial, partial [Nosema bombycis CQ1] |
| NBO_418gi001 | 48.31666667 | 22.19333333 | -1.122394558 | integral membrane protein [Nosema bombycis CQ1] |
| NBO_4g0050 | 74.25333333 | 36.30666667 | -1.03222131 | Insulin-degrading enzyme [Nosema bombycis CQ1] |
| MSTRG.63 | 72.43333333 | 163.4366667 | 1.174006012 | hypothetical spore wall protein 14 [Nosema bombycis] |
| NBO_26g0013 | 13029.31 | 4997.016667 | -1.382621749 | hypothetical spore wall protein [Nosema bombycis] |
| NBO_975g0001 | 368.69 | 182.2 | -1.016885328 | hypothetical protein NBO_975g0001 [Nosema bombycis CQ1] |
| NBO_929g0005 | 57.6 | 130.7233333 | 1.18237596 | hypothetical protein NBO_929g0005 [Nosema bombycis CQ1] |
| NBO_928g0001 | 2586.58 | 774.17 | -1.740323505 | hypothetical protein NBO_928g0001 [Nosema bombycis CQ1] |
| MSTRG.3032 | 164.05 | 58.25333333 | -1.493723084 | hypothetical protein NBO_8g0009 [Nosema bombycis CQ1] |
| NBO_7g0058 | 6.526666667 | 29.94666667 | 2.197977163 | hypothetical protein NBO_7g0058 [Nosema bombycis CQ1] |
| NBO_7g0006 | 15.22666667 | 2.633333333 | -2.531638092 | hypothetical protein NBO_7g0006 [Nosema bombycis CQ1] |
| NBO_76g0027 | 13.03666667 | 52.08333333 | 1.998246749 | hypothetical protein NBO_76g0027 [Nosema bombycis CQ1] |
| NBO_76g0022 | 776.8066667 | 269.3533333 | -1.528055663 | hypothetical protein NBO_76g0022 [Nosema bombycis CQ1] |
| NBO_76g0013 | 198.0966667 | 94.92666667 | -1.061319275 | hypothetical protein NBO_76g0013 [Nosema bombycis CQ1] |
| NBO_742g0003 | 23.42666667 | 7.476666667 | -1.647684571 | hypothetical protein NBO_742g0003 [Nosema bombycis CQ1] |
| NBO_73g0016 | 30.80333333 | 65.15 | 1.0806787 | hypothetical protein NBO_73g0016 [Nosema bombycis CQ1] |
| NBO_71g0003 | 16.56666667 | 35.99666667 | 1.119579966 | hypothetical protein NBO_71g0003, partial [Nosema bombycis CQ1] |
| NBO_70g0007 | 2616.106667 | 1036.223333 | -1.33608639 | hypothetical protein NBO_70g0007 [Nosema bombycis CQ1] |
| NBO_6gi003 | 3419.533333 | 1451.2 | -1.236553092 | hypothetical protein NBO_6gi003 [Nosema bombycis CQ1] |
| NBO_6g0118 | 7.25 | 0.653333333 | -3.472089842 | hypothetical protein NBO_6g0118 [Nosema bombycis CQ1] |
| NBO_6g0088 | 31.38333333 | 77.4 | 1.302334972 | hypothetical protein NBO_6g0088 [Nosema bombycis CQ1] |
| NBO_6g0086 | 11.77666667 | 27.88 | 1.243299313 | hypothetical protein NBO_6g0086 [Nosema bombycis CQ1] |
| NBO_6g0004 | 33.97 | 82.82333333 | 1.285776049 | hypothetical protein NBO_6g0004 [Nosema bombycis CQ1] |
| MSTRG.11026 | 3.26 | 21.81333333 | 2.742266378 | hypothetical protein NBO_67g0011 [Nosema bombycis CQ1] |
| NBO_66g0005 | 10.56333333 | 83.2 | 2.977518369 | hypothetical protein NBO_66g0005 [Nosema bombycis CQ1] |
| NBO_63g0015 | 9.526666667 | 19.59333333 | 1.040319443 | hypothetical protein NBO_63g0015, partial [Nosema bombycis CQ1] |
| NBO_62g0012 | 23.39 | 59.65333333 | 1.350710894 | hypothetical protein NBO_62g0012 [Nosema bombycis CQ1] |
| NBO_606g0001 | 113.33 | 27.03666667 | -2.067540614 | hypothetical protein NBO_606g0001 [Nosema bombycis CQ1] |
| NBO_55g0009 | 475.7333333 | 216.8466667 | -1.133477848 | hypothetical protein NBO_55g0009 [Nosema bombycis CQ1] |
| NBO_54g0012 | 12.01666667 | 30.39666667 | 1.338876366 | hypothetical protein NBO_54g0012 [Nosema bombycis CQ1] |
| NBO_547g0003 | 13.51666667 | 30.64 | 1.180676884 | hypothetical protein NBO_547g0003 [Nosema bombycis CQ1] |
| NBO_507g0012 | 60.11 | 122.7366667 | 1.029889383 | hypothetical protein NBO_507g0012 [Nosema bombycis CQ1] |
| NBO_4g0061 | 39.24 | 7.493333333 | -2.388645507 | hypothetical protein NBO_4g0061 [Nosema bombycis CQ1] |
| NBO_4g0007 | 241.6433333 | 104.02 | -1.21601825 | hypothetical protein NBO_4g0007 [Nosema bombycis CQ1] |
| NBO_49g0001 | 75.59666667 | 29.12 | -1.376312267 | hypothetical protein NBO_49g0001 [Nosema bombycis CQ1] |
| NBO_490g0001 | 59.95 | 20.53666667 | -1.545557719 | hypothetical protein NBO_490g0001 [Nosema bombycis CQ1] |
| NBO_48g0007 | 2010.91 | 824.4633333 | -1.286321275 | hypothetical protein NBO_48g0007 [Nosema bombycis CQ1] |
| NBO_480g0001 | 1.173333333 | 10.67 | 3.184875343 | hypothetical protein NBO_480g0001 [Nosema bombycis CQ1] |
| NBO_47g0001 | 0.88 | 12.89 | 3.87260493 | hypothetical protein NBO_47g0001 [Nosema bombycis CQ1] |
| NBO_468g0001 | 18.82333333 | 45.16 | 1.262523356 | hypothetical protein NBO_468g0001 [Nosema bombycis CQ1] |
| NBO_447g0004 | 2028.19 | 929.3033333 | -1.125971322 | hypothetical protein NBO_447g0004 [Nosema bombycis CQ1] |
| NBO_429g0001 | 38.12333333 | 17.47333333 | -1.125519415 | hypothetical protein NBO_429g0001 [Nosema bombycis CQ1] |
| NBO_424g0002 | 12.19 | 1.66 | -2.876442979 | hypothetical protein NBO_424g0002 [Nosema bombycis CQ1] |
| NBO_419g0005 | 8451.5 | 3891.513333 | -1.118876118 | hypothetical protein NBO_419g0005 [Nosema bombycis CQ1] |
| NBO_404g0002 | 51.61666667 | 23.87333333 | -1.112436959 | hypothetical protein NBO_404g0002 [Nosema bombycis CQ1] |
| NBO_3g0049 | 95.33666667 | 224.9433333 | 1.238458521 | hypothetical protein NBO_3g0049 [Nosema bombycis CQ1] |
| NBO_3g0033 | 26.71 | 69.52333333 | 1.380117279 | hypothetical protein NBO_3g0033 [Nosema bombycis CQ1] |
| NBO_38g0017 | 1622.243333 | 518.6166667 | -1.645249762 | hypothetical protein NBO_38g0017 [Nosema bombycis CQ1] |
| NBO_38g0014 | 568.5 | 248.7 | -1.192753842 | hypothetical protein NBO_38g0014 [Nosema bombycis CQ1] |
| NBO_38g0002 | 5.653333333 | 19.78333333 | 1.80711186 | hypothetical protein NBO_38g0002 [Nosema bombycis CQ1] |
| NBO_384g0005 | 45.06 | 95.31 | 1.080780277 | hypothetical protein NBO_384g0005 [Nosema bombycis CQ1] |
| NBO_380g0001 | 57.26333333 | 173.0266667 | 1.595310843 | hypothetical protein NBO_380g0001 [Nosema bombycis CQ1] |
| NBO_378g0002 | 1531.41 | 501.43 | -1.610740364 | hypothetical protein NBO_378g0002 [Nosema bombycis CQ1] |
| NBO_376g0001 | 374.61 | 171.73 | -1.125247322 | hypothetical protein NBO_376g0001 [Nosema bombycis CQ1] |
| NBO_367g0001 | 48.89333333 | 17.93 | -1.447262277 | hypothetical protein NBO_367g0001 [Nosema bombycis CQ1] |
| NBO_362g0010 | 5.943333333 | 19.12333333 | 1.685989815 | hypothetical protein NBO_362g0010 [Nosema bombycis CQ1] |
| NBO_359g0004 | 13.26333333 | 0.84 | -3.98091026 | hypothetical protein NBO_359g0004 [Nosema bombycis CQ1] |
| NBO_355g0002 | 0.001 | 16.92333333 | 14.04672614 | hypothetical protein NBO_355g0002 [Nosema bombycis CQ1] |
| NBO_30g0002 | 135.1333333 | 54.72 | -1.304243453 | hypothetical protein NBO_30g0002 [Nosema bombycis CQ1] |
| NBO_2g0075 | 61.50666667 | 29.1 | -1.079723639 | hypothetical protein NBO_2g0075 [Nosema bombycis CQ1] |
| NBO_2g0071 | 8.143333333 | 2.75 | -1.566187839 | hypothetical protein NBO_2g0071 [Nosema bombycis CQ1] |
| NBO_29g0016 | 37.54666667 | 75.92 | 1.015795159 | hypothetical protein NBO_29g0016, partial [Nosema bombycis CQ1] |
| NBO_29g0013 | 5569.413333 | 1834.17 | -1.602398005 | hypothetical protein NBO_29g0013 [Nosema bombycis CQ1] |
| NBO_28g0065 | 16.07 | 2.71 | -2.568005172 | hypothetical protein NBO_28g0065 [Nosema bombycis CQ1] |
| NBO_28g0056 | 558.28 | 186.0533333 | -1.585272635 | hypothetical protein NBO_28g0056 [Nosema bombycis CQ1] |
| NBO_28g0050 | 638.7666667 | 222.8366667 | -1.519302389 | hypothetical protein NBO_28g0050 [Nosema bombycis CQ1] |
| NBO_28g0046 | 43.66333333 | 108.3966667 | 1.311826215 | hypothetical protein NBO_28g0046 [Nosema bombycis CQ1] |
| NBO_28g0026 | 39.72333333 | 12.31666667 | -1.689374826 | hypothetical protein NBO_28g0026 [Nosema bombycis CQ1] |
| NBO_28g0021 | 3464.54 | 1245.273333 | -1.476201369 | hypothetical protein NBO_28g0021 [Nosema bombycis CQ1] |
| NBO_27g0061 | 230.19 | 95.02 | -1.276522049 | hypothetical protein NBO_27g0061 [Nosema bombycis CQ1] |
| NBO_27g0020 | 2127.92 | 943.0433333 | -1.174047943 | hypothetical protein NBO_27g0020 [Nosema bombycis CQ1] |
| NBO_27g0019 | 402.6333333 | 198.8133333 | -1.018052102 | hypothetical protein NBO_27g0019, partial [Nosema bombycis CQ1] |
| NBO_26g0007 | 6.21 | 65.44333333 | 3.39758106 | hypothetical protein NBO_26g0007 [Nosema bombycis CQ1] |
| NBO_204g0002 | 11.52333333 | 33.16 | 1.524885904 | hypothetical protein NBO_204g0002 [Nosema bombycis CQ1] |
| NBO_19g0014 | 402.33 | 156.8633333 | -1.358871154 | hypothetical protein NBO_19g0014 [Nosema bombycis CQ1] |
| NBO_18g0007 | 2.513333333 | 12.66333333 | 2.332983283 | hypothetical protein NBO_18g0007 [Nosema bombycis CQ1] |
| NBO_16g0051 | 12.5 | 72.69666667 | 2.539961119 | hypothetical protein NBO_16g0051 [Nosema bombycis CQ1] |
| NBO_16g0040 | 260.38 | 111.8333333 | -1.219268372 | hypothetical protein NBO_16g0040 [Nosema bombycis CQ1] |
| NBO_16g0028 | 31.17333333 | 76.83333333 | 1.301419916 | hypothetical protein NBO_16g0028 [Nosema bombycis CQ1] |
| NBO_16g0024 | 9.01 | 40.81333333 | 2.179441533 | hypothetical protein NBO_16g0024 [Nosema bombycis CQ1] |
| NBO_16g0003 | 403.39 | 116.86 | -1.787394126 | hypothetical protein NBO_16g0003 [Nosema bombycis CQ1] |
| NBO_1574g0001 | 621.95 | 296.4733333 | -1.068896258 | hypothetical protein NBO_1574g0001 [Nosema bombycis CQ1] |
| NBO_13g0046 | 100.4133333 | 201.8866667 | 1.007594783 | hypothetical protein NBO_13g0046 [Nosema bombycis CQ1] |
| NBO_137g0001 | 40.55666667 | 11.09666667 | -1.869812713 | hypothetical protein NBO_137g0001 [Nosema bombycis CQ1] |
| NBO_1302g0001 | 44.75666667 | 20.01 | -1.161381426 | hypothetical protein NBO_1302g0001 [Nosema bombycis CQ1] |
| NBO_12g0019 | 12296.51667 | 5690.293333 | -1.111674759 | hypothetical protein NBO_12g0019 [Nosema bombycis CQ1] |
| NBO_12g0008 | 48.41333333 | 8.69 | -2.477976347 | hypothetical protein NBO_12g0008 [Nosema bombycis CQ1] |
| NBO_1203g0001 | 9.496666667 | 37.20666667 | 1.970068025 | hypothetical protein NBO_1203g0001 [Nosema bombycis CQ1] |
| NBO_1116g0003 | 456.81 | 180.4633333 | -1.339888494 | hypothetical protein NBO_1116g0003 [Nosema bombycis CQ1] |
| NBO_10g0094 | 10.5 | 3.023333333 | -1.796177373 | hypothetical protein NBO_10g0094, partial [Nosema bombycis CQ1] |
| NBO_10g0037 | 15.88333333 | 46.44666667 | 1.548061349 | hypothetical protein NBO_10g0037 [Nosema bombycis CQ1] |
| NBO_10g0027 | 51.29 | 111.2066667 | 1.116493802 | hypothetical protein NBO_10g0027 [Nosema bombycis CQ1] |
| NBO_1086g0001 | 14.98666667 | 3.96 | -1.920107199 | hypothetical protein NBO_1086g0001 [Nosema bombycis CQ1] |
| NBO_1078g0002 | 39.59333333 | 100.9866667 | 1.350835389 | hypothetical protein NBO_1078g0002 [Nosema bombycis CQ1] |
| NBO_1170gi001 | 2097.113333 | 9618.863333 | 2.19746159 | HSP 101 related protein, partial [Nosema bombycis CQ1] |
| NBO_64g0030 | 63.63 | 149.3166667 | 1.230596181 | Homeobox protein HD-9 [Nosema bombycis CQ1] |
| NBO_36g0002 | 10.55666667 | 43.82666667 | 2.053654589 | Homeobox protein HD-8 [Nosema bombycis CQ1] |
| NBO_85g0005 | 367.5566667 | 145.54 | -1.336550973 | Homeobox protein HD-10 [Nosema bombycis CQ1] |
| NBO_11g0012 | 472.3333333 | 217.6533333 | -1.117773237 | Homeobox protein HD-10 [Nosema bombycis CQ1] |
| NBO_60g0027 | 262.91 | 546.7633333 | 1.05634748 | histone-binding protein N1/N2 [Nosema bombycis CQ1] |
| NBO_1320g0001 | 1047.446667 | 506.5133333 | -1.048204636 | Hexokinase-2 [Nosema bombycis CQ1] |
| NBO_108g0004 | 2627.693333 | 5434.56 | 1.04836632 | Heat shock protein 90, partial [Nosema bombycis CQ1] |
| NBO_468g0005 | 588.2433333 | 1323.766667 | 1.170163878 | Heat shock protein 90 [Nosema bombycis CQ1] |
| NBO_4g0017 | 2725.366667 | 11123.49 | 2.029087259 | Heat shock protein 101 [Nosema bombycis CQ1] |
| NBO_616g0003 | 11.97333333 | 32.9 | 1.458262735 | Heat shock factor protein 1 [Nosema bombycis CQ1] |
| NBO_16g0027 | 1551.196667 | 5621.106667 | 1.857472584 | Heat shock 70 kDa protein cognate 4 [Nosema bombycis CQ1] |
| NBO_58g0026 | 1.683333333 | 23.45333333 | 3.80040019 | GPN-loop GTPase 1 [Nosema bombycis CQ1] |
| NBO_15g0003 | 60.78666667 | 21.96333333 | -1.468657882 | GPI mannosyltransferase 2 [Nosema bombycis CQ1] |
| NBO_84g0004 | 484.3766667 | 223.4933333 | -1.115897572 | Glycerol-3-phosphate dehydrogenase, mitochondrial [Nosema bombycis CQ1] |
| NBO_53g0017 | 1395.286667 | 635.9633333 | -1.133546065 | glucose-6-phosphate isomerase [Nosema bombycis CQ1] |
| NBO_1118gi001 | 27.25666667 | 12.03666667 | -1.17917322 | glucose/Na cotransporter, partial [Nosema bombycis CQ1] |
| NBO_374g0007 | 1054.283333 | 524.4466667 | -1.007394665 | Glucosamine--fructose-6-phosphate aminotransferase 1 [Nosema bombycis CQ1] |
| NBO_49g0003 | 530.02 | 231.1733333 | -1.197071813 | Fructose-bisphosphate aldolase B [Nosema bombycis CQ1] |
| NBO_28g0053 | 218.08 | 74.25333333 | -1.55432977 | Forkhead box protein K2 [Nosema bombycis CQ1] |
| NBO_13g0067 | 0.313333333 | 9.436666667 | 4.912507182 | f-box domain-containing protein [Nosema ceranae] |
| NBO_369g0001 | 32.79666667 | 77.82666667 | 1.246715375 | exosome complex exonuclease rrp4, partial [Nosema bombycis CQ1] |
| NBO_108g0002 | 524.4466667 | 1244.286667 | 1.24645093 | endoplasmin [Nosema bombycis CQ1] |
| NBO_452g0005 | 19.45 | 74.01 | 1.927950061 | DNA-directed RNA polymerases I, II, and III subunit RPABC5 [Nosema bombycis CQ1] |
| NBO_2gi003 | 38.90666667 | 95.62 | 1.297295023 | DNA-directed RNA polymerases I and III subunit RPAC2 [Nosema bombycis CQ1] |
| NBO_28g0019 | 26.38666667 | 58.06333333 | 1.137818286 | DNA-directed RNA polymerases I and III subunit RPAC1 [Nosema bombycis CQ1] |
| NBO_32g0025 | 53.51 | 132.5766667 | 1.308946451 | DNA-directed RNA polymerase I subunit RPA12 [Nosema bombycis CQ1] |
| MSTRG.635 | 4.736666667 | 11.54333333 | 1.285115833 | DNA replication fork-blocking protein FOB1, partial [Nosema bombycis CQ1] |
| NBO_32g0034 | 87.13333333 | 204.9533333 | 1.233998814 | DNA polymerase kappa [Nosema bombycis CQ1] |
| NBO_633g0001 | 3.43 | 16.03333333 | 2.224793912 | DNA mismatch repair protein mutS [Nosema bombycis CQ1] |
| NBO_10g0053 | 7.436666667 | 20.45 | 1.45937283 | DNA mismatch repair protein Mlh1 [Nosema bombycis CQ1] |
| NBO_1291g0001 | 265.24 | 123.8133333 | -1.099131675 | Deoxyribodipyrimidine photo-lyase [Nosema bombycis CQ1] |
| NBO_554g0002 | 30.31333333 | 75.13333333 | 1.309500606 | Cytidylate kinase [Nosema bombycis CQ1] |
| NBO_707g0002 | 21.93333333 | 64.25333333 | 1.550646217 | Cyclin-dependent kinase C-2 [Nosema bombycis CQ1] |
| NBO_4g0049 | 255.6533333 | 123.9866667 | -1.044003853 | clathrin coat assembly protein [Nosema bombycis CQ1] |
| NBO_19g0018 | 202.84 | 94.41 | -1.103330595 | Chromosome segregation protein [Nosema bombycis CQ1] |
| NBO_1209gi001 | 62.07 | 139.0466667 | 1.163601109 | chromatin structure modulator [Nosema bombycis CQ1] |
| NBO_16g0047 | 43.93333333 | 20.94666667 | -1.068595285 | Chitin synthase export chaperone [Nosema bombycis CQ1] |
| NBO_58g0010 | 346.8366667 | 173.38 | -1.000318936 | Chitin synthase 7 [Nosema bombycis CQ1] |
| NBO_54g0020 | 31.00333333 | 87.68 | 1.499824463 | Checkpoint protein kinase [Nosema bombycis CQ1] |
| NBO_16g0023 | 706.2733333 | 2496.866667 | 1.821820252 | Chaperone protein dnaK [Nosema bombycis CQ1] |
| NBO_915g0002 | 62.26 | 146.6566667 | 1.236065172 | Cell division control protein 48 E, partial [Nosema bombycis CQ1] |
| NBO_362g0011 | 65.58666667 | 28.96666667 | -1.179008878 | CDP-diacylglycerol--inositol 3-phosphatidyltransferase [Nosema bombycis CQ1] |
| MSTRG.1990 | 80.85 | 292.1533333 | 1.853406071 | ATP-dependent DNA helicase PIF1 [Hypsizygus marmoreus] |
| NBO_78g0012 | 7.84 | 2.07 | -1.921222887 | ATP-binding cassette sub-family B member 7, mitochondrial [Nosema bombycis CQ1] |
| NBO_929g0004 | 26.47666667 | 110.0066667 | 2.054797549 | Anamorsin [Nosema bombycis CQ1] |
| NBO_552g0008 | 76.68 | 26.39333333 | -1.53867677 | Alpha/beta hydrolase fold-1 [Nosema bombycis CQ1] |
| NBO_582gi001 | 306.1433333 | 145.3 | -1.075172564 | alpha,alpha trehalose-phosphate synthase, partial [Nosema bombycis CQ1] |
| NBO_29gi002 | 536.1466667 | 259.45 | -1.047171178 | alpha,alpha trehalose-phosphate synthase [Nosema bombycis CQ1] |
| NBO_455g0003 | 17.32666667 | 68.64666667 | 1.986195535 | ADP-ribosylation factor-like protein 8A [Nosema bombycis CQ1] |
| NBO_38g0031 | 7.313333333 | 67.21666667 | 3.200217975 | Adenylate kinase [Nosema bombycis CQ1] |
| NBO_11gi001 | 50.96666667 | 273.7433333 | 2.425197924 | actin-like 53kDa protein [Nosema bombycis CQ1] |
| NBO_3g0006 | 4916.676667 | 2108.993333 | -1.221128948 | 60S ribosomal protein L6, partial [Nosema bombycis] |
| NBO_4g0009 | 115.4566667 | 56.12333333 | -1.040678877 | 3-ketodihydrosphingosine reductase [Nosema bombycis CQ1] |
| NBO_375g0009 | 183.7266667 | 88.66 | -1.051205771 | 2,3-bisphosphoglycerate-independent phosphoglycerate mutase [Nosema bombycis CQ1] |
| MSTRG.14183 | 81.97666667 | 37.03666667 | -1.146259068 | - |
| MSTRG.6740 | 9.176666667 | 35.59 | 1.955429824 | - |
| **Differentially expressed genes of *N. bombycis* between 5-day embryos and 1-day larvae during *N. bombycis* infection** | | | | |
| ID | I-E5_mean | I-L5_mean | log2(fc) | Description |
| NBO_4g0029 | 4325.663333 | 2096 | -1.045282668 | polar tube protein 3 [Nosema bombycis] |
| NBO_1116g0004 | 7190.743333 | 3247.01 | -1.147029087 | hypothetical protein NBO_1116g0004 [Nosema bombycis CQ1] |
| NBO_16g0041 | 6252.186667 | 2771.976667 | -1.173445739 | 40S ribosomal protein SA [Phytophthora nicotianae] |
| NBO_3g0004 | 434.01 | 169.7466667 | -1.35434504 | hypothetical protein NBO_3g0004 [Nosema bombycis CQ1] |
| NBO_29gi002 | 536.1466667 | 203.1733333 | -1.399916654 | alpha,alpha trehalose-phosphate synthase [Nosema bombycis CQ1] |
| NBO_84g0004 | 484.3766667 | 224.51 | -1.109349663 | Glycerol-3-phosphate dehydrogenase, mitochondrial [Nosema bombycis CQ1] |
| NBO_29g0027 | 947.33 | 441.31 | -1.102074634 | trehalose-phosphatase, partial [Nosema bombycis CQ1] |
| NBO_568g0004 | 309.6033333 | 135.6266667 | -1.190780138 | Alpha,alpha-trehalose-phosphate synthase, partial [Nosema bombycis CQ1] |
| MSTRG.2169 | 141.0033333 | 57.34 | -1.298115459 | threonyl-tRNA synthetase [Nosema bombycis CQ1] |
| NBO_49g0003 | 530.02 | 189.4166667 | -1.484483522 | Fructose-bisphosphate aldolase B [Nosema bombycis CQ1] |
| NBO_11g0003 | 1797.203333 | 853.48 | -1.074324391 | UTP-glucose-1-phosphate uridylyltransferase [Nosema bombycis CQ1] |
| NBO_19g0014 | 402.33 | 192.3266667 | -1.064820507 | hypothetical protein NBO_19g0014 [Nosema bombycis CQ1] |
| NBO_261g0001 | 64.75666667 | 268.9433333 | 2.054201597 | endonuclease [Nosema bombycis CQ1] |
| NBO_64g0031 | 184.51 | 753.15 | 2.029238217 | Homeobox-leucine zipper protein ROC4 [Nosema bombycis CQ1] |
| NBO_38g0002 | 5.653333333 | 61.52333333 | 3.443960001 | hypothetical protein NBO_38g0002 [Nosema bombycis CQ1] |
| NBO_1332gi002 | 94.37666667 | 27.00666667 | -1.805114633 | threonyl-tRNA synthetase [Nosema bombycis CQ1] |
| NBO_16g0028 | 31.17333333 | 139.9766667 | 2.166802024 | hypothetical protein NBO_16g0028 [Nosema bombycis CQ1] |
| NBO_76gi004 | 162.7433333 | 76.44666667 | -1.090072944 | threonyl-tRNA synthetase [Nosema bombycis CQ1] |
| NBO_69g0015 | 177.16 | 80.10333333 | -1.145118719 | serine threonine protein kinase [Nosema ceranae] |
| NBO_11g0041 | 185.08 | 84.40666667 | -1.132720148 | small-conductance mechanosensitive channel protein [Nosema ceranae] |
| NBO_365g0001 | 167.01 | 74.48666667 | -1.164880382 | Threonyl-tRNA synthetase, partial [Nosema bombycis CQ1] |
| NBO_11g0032 | 2024.953333 | 4962.896667 | 1.293293756 | Peptidase S8 and S53, subtilisin, kexin, sedolisin [Nosema bombycis CQ1] |
| NBO_447g0003 | 384.6233333 | 174.37 | -1.141294438 | Protein of unknown function GLTT, partial [Nosema bombycis CQ1] |
| NBO_73g0026 | 299.3933333 | 608.23 | 1.02257488 | hypothetical protein NBO_73g0026 [Nosema bombycis CQ1] |
| NBO_707g0002 | 21.93333333 | 107.0366667 | 2.286908105 | Cyclin-dependent kinase C-2 [Nosema bombycis CQ1] |
| NBO_985g0001 | 33.76333333 | 132.0133333 | 1.967154401 | hypothetical protein NBO_985g0001 [Nosema bombycis CQ1] |
| NBO_31gi002 | 95.73 | 47.35666667 | -1.015403573 | small-conductance mechanosensitive channel protein [Nosema ceranae] |
| NBO_1218g0002 | 116.99 | 50.12 | -1.222926897 | Zinc finger C2H2 protein [Nosema bombycis CQ1] |
| NBO_16g0024 | 9.01 | 65.75333333 | 2.867465022 | hypothetical protein NBO_16g0024 [Nosema bombycis CQ1] |
| NBO_29g0021 | 34.10666667 | 123.5666667 | 1.857163946 | Spore wall and anchoring disk complex protein [Nosema bombycis CQ1] |
| NBO_364g0016 | 164.84 | 366.29 | 1.151919946 | hypothetical protein NBO_364g0016 [Nosema bombycis CQ1] |
| NBO_38g0031 | 7.313333333 | 90.47 | 3.628838446 | Adenylate kinase [Nosema bombycis CQ1] |
| NBO_32g0024 | 130.8833333 | 281.2933333 | 1.103793965 | General transcriptional corepressor ssn6 [Nosema bombycis CQ1] |
| NBO_64g0030 | 63.63 | 276.1366667 | 2.117603441 | Homeobox protein HD-9 [Nosema bombycis CQ1] |
| MSTRG.14183 | 81.97666667 | 36.13666667 | -1.181749894 | - |
| NBO_73g0017 | 8.04 | 76.23666667 | 3.245217634 | 26S protease regulatory subunit 4, partial [Nosema bombycis CQ1] |
| NBO_19g0018 | 202.84 | 88.05333333 | -1.203892655 | Chromosome segregation protein [Nosema bombycis CQ1] |
| NBO_49g0004 | 28.52666667 | 0.09 | -8.308170461 | Fructose-bisphosphate aldolase C [Nosema bombycis CQ1] |
| NBO_1057g0002 | 2163.236667 | 4813.01 | 1.15374791 | histone h3 [Nosema apis BRL 01] |
| NBO_32g0030 | 3.736666667 | 24.01333333 | 2.684011903 | hypothetical protein NBO_32g0030 [Nosema bombycis CQ1] |
| MSTRG.7278 | 6.056666667 | 38.97666667 | 2.686014796 | Tricalbin-2, partial [Nosema bombycis CQ1] |
| NBO_1271g0002 | 70.07666667 | 152.8566667 | 1.125173418 | hypothetical protein NBO_1271g0002 [Nosema bombycis CQ1] |
| NBO_66g0051 | 38.39333333 | 140.9366667 | 1.876119272 | hypothetical protein NBO_66g0051 [Nosema bombycis CQ1] |
| NBO_81g0019 | 0.863333333 | 49.29 | 5.835233479 | protein fam72a [Nosema apis BRL 01] |
| NBO_480g0001 | 1.173333333 | 18.85 | 4.00587969 | hypothetical protein NBO_480g0001 [Nosema bombycis CQ1] |
| NBO_55g0012 | 6.666666667 | 43.17 | 2.694991594 | hypothetical protein NBO_55g0012 [Nosema bombycis CQ1] |
| NBO_1082g0001 | 6.81 | 55.24 | 3.019986616 | endonuclease [Nosema bombycis CQ1] |
| NBO_360g0001 | 62.09 | 165.9266667 | 1.418112929 | transcription initiation factor TFIID 20/15kDa subunit [Nosema bombycis CQ1] |
| NBO_15g0005 | 58.51 | 151.2266667 | 1.369957437 | negative regulator of transcription [Encephalitozoon cuniculi GB-M1] |
| NBO_20g0004 | 2.546666667 | 27.95333333 | 3.456338287 | Bifunctional xylanase/deacetylase [Nosema bombycis CQ1] |
| NBO_378g0013 | 110.37 | 50.51333333 | -1.12761193 | spore wall protein 12 [Endoreticulatus sp. ZJ] |
| NBO_375g0010 | 74.32 | 34.09666667 | -1.124119795 | 2,3-bisphosphoglycerate-independent phosphoglycerate mutase [Nosema bombycis CQ1] |
| NBO_1230g0003 | 179.1433333 | 411.79 | 1.20079444 | swi5-like protein [Nosema ceranae] |
| NBO_974g0001 | 3.43 | 24.97 | 2.86391534 | Polysaccharide deacetylase [Nosema bombycis CQ1] |
| NBO_281g0003 | 152.7266667 | 330.02 | 1.111601474 | hypothetical protein NBO_281g0003 [Nosema bombycis CQ1] |
| NBO_11g0052 | 123.6 | 264.8733333 | 1.099623861 | Flap endonuclease 1-A [Nosema bombycis CQ1] |
| NBO_13g0031 | 182.98 | 395.99 | 1.11377803 | eukaryotic translation initiation factor 4E [Encephalitozoon romaleae SJ-2008] |
| NBO_1140g0001 | 88.68666667 | 192.42 | 1.117469631 | Synaptobrevin-like protein [Nosema bombycis CQ1] |
| NBO_560g0001 | 6.233333333 | 20.48666667 | 1.716609497 | DNA-binding protein SMUBP-2 [Nosema bombycis CQ1] |
| NBO_32g0025 | 53.51 | 150.3533333 | 1.490476419 | DNA-directed RNA polymerase I subunit RPA12 [Nosema bombycis CQ1] |
| NBO_369g0001 | 32.79666667 | 81.38 | 1.311125088 | exosome complex exonuclease rrp4, partial [Nosema bombycis CQ1] |
| NBO_11gi001 | 50.96666667 | 137.38 | 1.430546084 | actin-like 53kDa protein [Nosema bombycis CQ1] |
| NBO_28g0074 | 71.84333333 | 159.7466667 | 1.152859634 | u3 small nucleolar ribonucleoprotein component mpp10 [Nosema ceranae] |
| MSTRG.5652 | 13.15666667 | 30.63333333 | 1.219308342 | pol polyprotein, partial [Nosema apis BRL 01] |
| NBO_53g0005 | 6.866666667 | 28.95 | 2.075881512 | Polysaccharide deacetylase [Nosema bombycis CQ1] |
| NBO_10g0027 | 51.29 | 133.5133333 | 1.380234348 | hypothetical protein NBO_10g0027 [Nosema bombycis CQ1] |
| NBO_73g0028 | 86.78 | 193.17 | 1.154436565 | hypothetical protein NBO_73g0028 [Nosema bombycis CQ1] |
| NBO_401g0003 | 66.64 | 170.2233333 | 1.352968502 | Homeobox protein HD-9 [Nosema bombycis CQ1] |
| NBO_568g0002 | 12.69 | 35.82666667 | 1.497341751 | Spore wall and anchoring disk complex protein [Nosema bombycis CQ1] |
| NBO_54g0020 | 31.00333333 | 77.89 | 1.329014782 | Checkpoint protein kinase [Nosema bombycis CQ1] |
| NBO_10g0001 | 47.42 | 120.3566667 | 1.343748488 | dna-directed rna polymerase ii subunit rpb7 [Nosema ceranae] |
| NBO_451g0007 | 606.3033333 | 1339.286667 | 1.143353135 | hypothetical protein NBO_451g0007 [Nosema bombycis CQ1] |
| NBO_586gi001 | 41.41666667 | 14.05333333 | -1.55929908 | valyl trna synthetase [Nosema bombycis CQ1] |
| MSTRG.1990 | 80.85 | 162.1666667 | 1.004157625 | ATP-dependent DNA helicase PIF1 [Hypsizygus marmoreus] |
| NBO_10g0094 | 10.5 | 2.926666667 | -1.843058984 | hypothetical protein NBO_10g0094, partial [Nosema bombycis CQ1] |
| NBO_48g0004 | 11.62333333 | 34.95666667 | 1.588543758 | mevalonate kinase [Nosema bombycis CQ1] |
| MSTRG.6740 | 9.176666667 | 25.22 | 1.458526166 | - |
| NBO_452g0005 | 19.45 | 82.26333333 | 2.080479376 | DNA-directed RNA polymerases I, II, and III subunit RPABC5 [Nosema bombycis CQ1] |
| NBO_66g0050 | 68.74666667 | 155.49 | 1.177460131 | hypothetical protein NBO_66g0050 [Nosema bombycis CQ1] |
| NBO_166g0001 | 5.046666667 | 20.99333333 | 2.056528552 | Polysaccharide deacetylase [Nosema bombycis CQ1] |
| NBO_3g0016 | 37.36 | 12.38333333 | -1.593094745 | hypothetical protein NBO_3g0016 [Nosema bombycis CQ1] |
| NBO_6g0088 | 31.38333333 | 70.83333333 | 1.174429841 | hypothetical protein NBO_6g0088 [Nosema bombycis CQ1] |
| NBO_30g0008 | 153.0933333 | 359.9833333 | 1.233518654 | Target SNARE coiled-coil region [Nosema bombycis CQ1] |
| NBO_10g0041 | 54.02 | 131.12 | 1.279322215 | hypothetical protein NBO_10g0041 [Nosema bombycis CQ1] |
| NBO_10g0110 | 27.30666667 | 94.77333333 | 1.795227966 | DNA-directed RNA polymerases I and III subunit RPAC2 [Nosema bombycis CQ1] |
| NBO_929g0004 | 26.47666667 | 79.57333333 | 1.587563532 | Anamorsin [Nosema bombycis CQ1] |
| NBO_404g0004 | 25.44 | 64.05 | 1.3320999 | Serum response factor [Nosema bombycis CQ1] |
| NBO_28g0012 | 127.5266667 | 258.47 | 1.019197884 | Transcription elongation factor SPT4 [Nosema bombycis CQ1] |
| NBO_570g0004 | 69.33666667 | 138.7833333 | 1.001143937 | NIF3-like protein 1, partial [Nosema bombycis CQ1] |
| NBO_18g0012 | 16.91333333 | 68.63333333 | 2.020748405 | Cyclin-dependent kinases regulatory subunit [Nosema bombycis CQ1] |
| NBO_401g0008 | 82.34 | 168.8733333 | 1.036276177 | transcription elongation factor s-ii [Nosema ceranae] |
| NBO_84g0003 | 41.22 | 131.0166667 | 1.668333939 | Ferric reductase-like protein [Pseudoloma neurophilia] |
| NBO_63g0020 | 26.87 | 54.85 | 1.029495299 | Glutamate synthase NADPH small chain [Nosema bombycis CQ1] |
| NBO_2g0071 | 8.143333333 | 3.433333333 | -1.246009526 | hypothetical protein NBO_2g0071 [Nosema bombycis CQ1] |
| MSTRG.14040 | 8.026666667 | 19.26 | 1.262734812 | hypothetical protein NBO_11g0042 [Nosema bombycis CQ1] |
| NBO_411gi001 | 28.52 | 58.41333333 | 1.034323732 | GTP-binding protein [Nosema bombycis CQ1] |
| NBO_455g0003 | 17.32666667 | 83.94333333 | 2.27642162 | ADP-ribosylation factor-like protein 8A [Nosema bombycis CQ1] |
| NBO_39g0002 | 13.86333333 | 42.09333333 | 1.602317576 | hypothetical protein NBO_39g0002 [Nosema bombycis CQ1] |
| NBO_508g0029 | 22.81666667 | 88.03333333 | 1.947961855 | DNA-directed RNA polymerases I, II, and III subunit RPABC5 [Nosema bombycis CQ1] |
| NBO_401g0004 | 5.553333333 | 46.08 | 3.052714817 | Homeobox protein [Nosema bombycis CQ1] |
| MSTRG.7238 | 2.95 | 10.14666667 | 1.782218999 | transposase [Nosema bombycis] |
| NBO_2gi003 | 38.90666667 | 104.6233333 | 1.427115353 | DNA-directed RNA polymerases I and III subunit RPAC2 [Nosema bombycis CQ1] |
| NBO_11g0045 | 42.19 | 85.64333333 | 1.021439861 | hypothetical protein NBO_11g0045 [Nosema bombycis CQ1] |
| NBO_443g0001 | 39.45666667 | 82.17 | 1.058342686 | nucleic acid-binding protein [Nosema ceranae] |
| NBO_380g0001 | 57.26333333 | 142.1566667 | 1.3117982 | hypothetical protein NBO_380g0001 [Nosema bombycis CQ1] |
| NBO_376g0007 | 70.86 | 146.84 | 1.051201649 | hypothetical protein NBO_376g0007 [Nosema bombycis CQ1] |
| NBO_546g0001 | 1.53 | 9.13 | 2.577083207 | hypothetical protein NBO_546g0001 [Nosema bombycis CQ1] |
| NBO_58g0013 | 24.91333333 | 7.21 | -1.788846898 | hypothetical protein NBO_58g0013 [Nosema bombycis CQ1] |
| NBO_1608g0001 | 28.19666667 | 58.30666667 | 1.048136226 | Isoleucyl-tRNA synthetase, cytoplasmic [Nosema bombycis CQ1] |
| **Differentially expressed genes of *N. bombycis* between 5-day embryos and 10-day larvae during *N. bombycis* infection** | | | | |
| ID | I-E5_mean | I-L10_mean | log2(fc) | Description |
| NBO_38g0002 | 5.653333333 | 172.1466667 | 4.928392672 | hypothetical protein NBO_38g0002 [Nosema bombycis CQ1] |
| NBO_4g0029 | 4325.663333 | 2027.24 | -1.093404489 | polar tube protein 3 [Nosema bombycis] |
| NBO_16g0028 | 31.17333333 | 213.6066667 | 2.77657234 | hypothetical protein NBO_16g0028 [Nosema bombycis CQ1] |
| NBO_32gi001 | 1041.966667 | 495.8833333 | -1.071236483 | threonyl-tRNA synthetase [Nosema bombycis CQ1] |
| MSTRG.5652 | 13.15666667 | 114.6633333 | 3.123538201 | pol polyprotein, partial [Nosema apis BRL 01] |
| NBO_60g0017 | 230.4366667 | 109.95 | -1.06752269 | Transcription-associated protein 1 [Nosema bombycis CQ1] |
| NBO_64g0031 | 184.51 | 777.7566667 | 2.075619847 | Homeobox-leucine zipper protein ROC4 [Nosema bombycis CQ1] |
| NBO_26g0013 | 13029.31 | 5071.586667 | -1.361251608 | hypothetical spore wall protein [Nosema bombycis] |
| NBO_11gi001 | 50.96666667 | 332.61 | 2.706205638 | actin-like 53kDa protein [Nosema bombycis CQ1] |
| NBO_11g0003 | 1797.203333 | 778.9366667 | -1.206175706 | UTP-glucose-1-phosphate uridylyltransferase [Nosema bombycis CQ1] |
| NBO_166g0002 | 139.69 | 65.56666667 | -1.091194289 | Transcription-associated protein 1, partial [Nosema bombycis CQ1] |
| NBO_16g0024 | 9.01 | 113.4666667 | 3.65459762 | hypothetical protein NBO_16g0024 [Nosema bombycis CQ1] |
| NBO_76g0014 | 66.98666667 | 6.803333333 | -3.299560282 | hypothetical protein NBO_76g0014 [Nosema bombycis CQ1] |
| NBO_261g0001 | 64.75666667 | 254.1066667 | 1.972333596 | endonuclease [Nosema bombycis CQ1] |
| NBO_48g0007 | 2010.91 | 941.9833333 | -1.094075075 | hypothetical protein NBO_48g0007 [Nosema bombycis CQ1] |
| NBO_974g0002 | 218.6366667 | 108.2333333 | -1.014390486 | Transcription-associated protein 1 [Nosema bombycis CQ1] |
| MSTRG.5960 | 5.983333333 | 42.09333333 | 2.814570417 | pol polyprotein, partial [Nosema apis BRL 01] |
| NBO_985g0001 | 33.76333333 | 136.0033333 | 2.010112763 | hypothetical protein NBO_985g0001 [Nosema bombycis CQ1] |
| NBO_1020g0001 | 661.04 | 1368.756667 | 1.050056514 | Eukaryotic translation initiation factor 2-alpha kinase 1 [Nosema bombycis CQ1] |
| NBO_376g0002 | 242.96 | 805.1633333 | 1.728562661 | hypothetical protein NBO_376g0002 [Nosema bombycis CQ1] |
| NBO_3g0004 | 434.01 | 209.44 | -1.051191282 | hypothetical protein NBO_3g0004 [Nosema bombycis CQ1] |
| NBO_29gi002 | 536.1466667 | 238.4466667 | -1.168961099 | alpha,alpha trehalose-phosphate synthase [Nosema bombycis CQ1] |
| NBO_29g0021 | 34.10666667 | 128.3266667 | 1.911695329 | Spore wall and anchoring disk complex protein [Nosema bombycis CQ1] |
| NBO_76g0021 | 689.6166667 | 1863.416667 | 1.434083755 | hypothetical protein NBO_76g0021 [Nosema bombycis CQ1] |
| NBO_707g0002 | 21.93333333 | 102.15 | 2.219492216 | Cyclin-dependent kinase C-2 [Nosema bombycis CQ1] |
| NBO_11g0046 | 151.8933333 | 41.02666667 | -1.888424703 | Xaa-Pro aminopeptidase 1 [Nosema bombycis CQ1] |
| MSTRG.75 | 7.193333333 | 31.79333333 | 2.143991918 | hypothetical protein H312_01097 [Anncaliia algerae PRA339] |
| NBO_69g0015 | 177.16 | 75.19 | -1.236440196 | serine threonine protein kinase [Nosema ceranae] |
| MSTRG.4355 | 61.71666667 | 212.6966667 | 1.785065375 | atp-dependent helicase rrm3-like protein, partial [Nosema ceranae] |
| NBO_364g0016 | 164.84 | 378.6666667 | 1.19986206 | hypothetical protein NBO_364g0016 [Nosema bombycis CQ1] |
| NBO_671gi001 | 63.80333333 | 285.0666667 | 2.159595649 | hiv1-tat interacting protein [Nosema bombycis CQ1] |
| MSTRG.8023 | 122.4066667 | 54.93 | -1.156015937 | trans-sialidase [Cordyceps brongniartii RCEF 3172] |
| NBO_1057g0002 | 2163.236667 | 4784.186667 | 1.14508217 | histone h3 [Nosema apis BRL 01] |
| NBO_63g0022 | 1448.39 | 718.8133333 | -1.010761046 | hypothetical protein NBO_63g0022 [Nosema bombycis CQ1] |
| NBO_73g0026 | 299.3933333 | 652.93 | 1.124886233 | hypothetical protein NBO_73g0026 [Nosema bombycis CQ1] |
| NBO_444g0008 | 194.67 | 90.17333333 | -1.110257814 | Cysteinyl-tRNA synthetase, cytoplasmic [Nosema bombycis CQ1] |
| NBO_27g0025 | 688.76 | 1792.54 | 1.379932047 | skt5-like protein [Nosema apis BRL 01] |
| MSTRG.1911 | 34.24 | 133.9166667 | 1.967580916 | - |
| NBO_34g0010 | 1563.37 | 3448.436667 | 1.141283212 | mitochondrial protein import protein MAS5 [Nosema pernyi] |
| NBO_10g0039 | 174.26 | 450.3033333 | 1.369655709 | NUDIX hydrolase [Nosema bombycis CQ1] |
| MSTRG.7278 | 6.056666667 | 61.02 | 3.332686261 | Tricalbin-2, partial [Nosema bombycis CQ1] |
| NBO_39g0002 | 13.86333333 | 93.79333333 | 2.758211198 | hypothetical protein NBO_39g0002 [Nosema bombycis CQ1] |
| NBO_27g0024 | 554.9133333 | 1319.373333 | 1.249518478 | Chitin synthase regulatory factor 4 [Nosema bombycis CQ1] |
| MSTRG.63 | 72.43333333 | 157.5933333 | 1.121480832 | hypothetical spore wall protein 14 [Nosema bombycis] |
| NBO_555g0001 | 210.1033333 | 101.2233333 | -1.053557162 | hypothetical protein NBO_555g0001 [Nosema bombycis CQ1] |
| NBO_411gi001 | 28.52 | 110.7866667 | 1.957738375 | GTP-binding protein [Nosema bombycis CQ1] |
| NBO_27g0061 | 230.19 | 693.7 | 1.591486724 | hypothetical protein NBO_27g0061 [Nosema bombycis CQ1] |
| NBO_549g0007 | 53.10666667 | 24.52333333 | -1.114737888 | DNA polymerase epsilon catalytic subunit A [Nosema bombycis CQ1] |
| NBO_67g0015 | 90.92 | 41.52 | -1.13079124 | hypothetical protein NBO_67g0015 [Nosema bombycis CQ1] |
| NBO_800gi001 | 98.8 | 36.00333333 | -1.456380559 | dopey-like leucine zipper transcription factor [Nosema ceranae] |
| NBO_11g0041 | 185.08 | 83.19666667 | -1.153551372 | small-conductance mechanosensitive channel protein [Nosema ceranae] |
| MSTRG.1990 | 80.85 | 684.5766667 | 3.081892441 | ATP-dependent DNA helicase PIF1 [Hypsizygus marmoreus] |
| MSTRG.3488 | 13.39333333 | 52.38333333 | 1.967592803 | pol polyprotein [Nosema bombycis] |
| NBO_48g0008 | 391.4633333 | 188.1433333 | -1.057045013 | Glycerophosphodiester phosphodiesterase gde1 [Nosema bombycis CQ1] |
| NBO_1332gi002 | 94.37666667 | 37.68666667 | -1.324376021 | threonyl-tRNA synthetase [Nosema bombycis CQ1] |
| NBO_365g0001 | 167.01 | 82.62 | -1.015371524 | Threonyl-tRNA synthetase, partial [Nosema bombycis CQ1] |
| NBO_76g0003 | 98.85666667 | 213.0666667 | 1.107894741 | hypothetical protein NBO_76g0003 [Nosema bombycis CQ1] |
| NBO_1574g0001 | 621.95 | 303.0733333 | -1.037131685 | hypothetical protein NBO_1574g0001 [Nosema bombycis CQ1] |
| NBO_3g0047 | 514.85 | 1047.676667 | 1.024969468 | ras-like protein gene member c [Nosema apis BRL 01] |
| NBO_38g0031 | 7.313333333 | 92.09333333 | 3.654495698 | Adenylate kinase [Nosema bombycis CQ1] |
| MSTRG.635 | 4.736666667 | 38.04666667 | 3.005826008 | DNA replication fork-blocking protein FOB1, partial [Nosema bombycis CQ1] |
| NBO_87g0001 | 97.84 | 301.3266667 | 1.622832045 | Homeobox protein HD-8 [Nosema bombycis CQ1] |
| NBO_537g0001 | 182.69 | 595.3133333 | 1.70425154 | DNA replication fork-blocking protein FOB1, partial [Nosema bombycis CQ1] |
| NBO_28g0022 | 139.2166667 | 393.6566667 | 1.499605973 | leptin receptor gene-related protein [Nosema bombycis CQ1] |
| NBO_508g0021 | 102.64 | 281.6333333 | 1.456225022 | Rac-like GTP-binding protein ARAC7 [Nosema bombycis CQ1] |
| NBO_1230g0003 | 179.1433333 | 510.19 | 1.509920266 | swi5-like protein [Nosema ceranae] |
| NBO_80g0017 | 125.93 | 264.9766667 | 1.073243311 | Integrator complex subunit 11 [Nosema bombycis CQ1] |
| NBO_53g0011 | 212.5033333 | 473.6666667 | 1.156386677 | hypothetical protein NBO_53g0011 [Nosema bombycis CQ1] |
| NBO_1059gi002 | 160.77 | 348.6133333 | 1.116629527 | glycerol 3-phosphate dehydrogenase, partial [Nosema bombycis CQ1] |
| NBO_10g0055 | 470.1366667 | 219.5466667 | -1.098552572 | hypothetical protein NBO_10g0055, partial [Nosema bombycis CQ1] |
| NBO_1066g0001 | 202.2533333 | 522.9266667 | 1.370445162 | hypothetical protein NBO_1066g0001 [Nosema bombycis CQ1] |
| NBO_1230gi001 | 25.28666667 | 113.7133333 | 2.16895265 | hypothetical protein NBO_1230gi001 [Nosema bombycis CQ1] |
| NBO_16g0027 | 1551.196667 | 3220.13 | 1.053737324 | Heat shock 70 kDa protein cognate 4 [Nosema bombycis CQ1] |
| NBO_437g0002 | 56.43666667 | 164.9266667 | 1.547119999 | hypothetical protein NBO_437g0002 [Nosema bombycis CQ1] |
| MSTRG.1368 | 52.03 | 121.8 | 1.227098521 | pol polyprotein [Nosema bombycis] |
| NBO_60g0027 | 262.91 | 544.5666667 | 1.05053966 | histone-binding protein N1/N2 [Nosema bombycis CQ1] |
| NBO_1218g0002 | 116.99 | 50.63 | -1.208320828 | Zinc finger C2H2 protein [Nosema bombycis CQ1] |
| NBO_546g0001 | 1.53 | 26.26 | 4.101263358 | hypothetical protein NBO_546g0001 [Nosema bombycis CQ1] |
| MSTRG.3956 | 164.9366667 | 347.8833333 | 1.076691406 | pol polyprotein [Nosema apis BRL 01] |
| NBO_8g0045 | 267.6533333 | 536.58 | 1.003427662 | signal peptidase like protein [Nosema bombycis CQ1] |
| NBO_6g0076 | 222.83 | 548.5933333 | 1.299793612 | dna-directed rna polymerase ii subunit rpb7 [Nosema ceranae] |
| MSTRG.4585 | 15.76 | 66.69 | 2.081202914 | Tigger transposable element-derived protein 6 [Rhizoctonia solani AG-1 IB] |
| NBO_29g0012 | 88.33333333 | 269.1433333 | 1.607344831 | leptin receptor gene-related protein [Nosema bombycis CQ1] |
| NBO_38g0012 | 77.89666667 | 190.0233333 | 1.286543081 | hypothetical protein NBO_38g0012 [Nosema bombycis CQ1] |
| NBO_264g0001 | 68.58666667 | 202.45 | 1.561565596 | hypothetical protein NBO_264g0001, partial [Nosema bombycis CQ1] |
| NBO_38g0009 | 80.77 | 230.2733333 | 1.511455906 | hypothetical protein NBO_38g0009 [Nosema bombycis CQ1] |
| NBO_452g0003 | 158.7333333 | 377.69 | 1.250597467 | Peptidyl-prolyl cis-trans isomerase NIMA-interacting 1 [Nosema bombycis CQ1] |
| NBO_490g0001 | 59.95 | 29.37666667 | -1.029089049 | hypothetical protein NBO_490g0001 [Nosema bombycis CQ1] |
| NBO_32g0030 | 3.736666667 | 26.65333333 | 2.834492194 | hypothetical protein NBO_32g0030 [Nosema bombycis CQ1] |
| NBO_64g0030 | 63.63 | 215.2633333 | 1.758323574 | Homeobox protein HD-9 [Nosema bombycis CQ1] |
| NBO_437g0004 | 92.36333333 | 250.6566667 | 1.440320461 | hypothetical protein NBO_437g0004 [Nosema bombycis CQ1] |
| NBO_1517g0004 | 138.1866667 | 315.3033333 | 1.190122002 | Rac-like GTP-binding protein ARAC7 [Nosema bombycis CQ1] |
| NBO_31gi002 | 95.73 | 45.04 | -1.087764282 | small-conductance mechanosensitive channel protein [Nosema ceranae] |
| NBO_417g0013 | 255.2833333 | 533.26 | 1.062739763 | ATP-binding protein [Ordospora colligata OC4] |
| NBO_24g0004 | 86.38 | 220.12 | 1.349521012 | myosin heavy chain [Nosema ceranae] |
| NBO_28g0074 | 71.84333333 | 190.13 | 1.404059994 | u3 small nucleolar ribonucleoprotein component mpp10 [Nosema ceranae] |
| NBO_1110g0001 | 85.41333333 | 41.67666667 | -1.035221403 | SEC31-like protein invovled in vesicular transport from ER to Golgi [Nosema bombycis CQ1] |
| NBO_41g0014 | 69.57333333 | 147.2366667 | 1.081530645 | Vacuolar amino acid transporter 5 [Nosema bombycis CQ1] |
| NBO_380g0001 | 57.26333333 | 212.83 | 1.894017966 | hypothetical protein NBO_380g0001 [Nosema bombycis CQ1] |
| NBO_49g0004 | 28.52666667 | 0.001 | -14.80002356 | Fructose-bisphosphate aldolase C [Nosema bombycis CQ1] |
| NBO_32g0025 | 53.51 | 186.8 | 1.803614021 | DNA-directed RNA polymerase I subunit RPA12 [Nosema bombycis CQ1] |
| NBO_53g0005 | 6.866666667 | 38.11 | 2.472487771 | Polysaccharide deacetylase [Nosema bombycis CQ1] |
| NBO_58g0009 | 59.28333333 | 27.37666667 | -1.114679771 | DNA repair protein RAD16 [Nosema bombycis CQ1] |
| NBO_1271g0002 | 70.07666667 | 149.62 | 1.094296979 | hypothetical protein NBO_1271g0002 [Nosema bombycis CQ1] |
| NBO_568g0002 | 12.69 | 44.92666667 | 1.823879956 | Spore wall and anchoring disk complex protein [Nosema bombycis CQ1] |
| NBO_73g0028 | 86.78 | 226.1966667 | 1.382143178 | hypothetical protein NBO_73g0028 [Nosema bombycis CQ1] |
| NBO_72g0013 | 151.56 | 308.77 | 1.026643543 | Rab GTPase interacting factor Golgi membrane protein [Encephalitozoon romaleae SJ-2008] |
| NBO_753g0001 | 34.78 | 136.6233333 | 1.973874059 | leptin receptor gene-related protein [Nosema bombycis CQ1] |
| NBO_73g0023 | 37.24666667 | 115.22 | 1.629207938 | hypothetical protein NBO_73g0023 [Nosema bombycis CQ1] |
| NBO_27g0006 | 109.0833333 | 241.3933333 | 1.145955142 | integral membrane [Nosema apis BRL 01] |
| NBO_3g0028 | 80.93666667 | 182.53 | 1.173268261 | vacuolar atp synthase subunit d [Nosema ceranae] |
| NBO_62g0012 | 23.39 | 65.29333333 | 1.481043834 | hypothetical protein NBO_62g0012 [Nosema bombycis CQ1] |
| NBO_3g0041 | 8.96 | 52.74666667 | 2.557509288 | DNA-directed RNA polymerase III subunit RPC6 [Nosema bombycis CQ1] |
| MSTRG.7238 | 2.95 | 15.42666667 | 2.386639504 | transposase [Nosema bombycis] |
| NBO_979g0001 | 37.26666667 | 112.7633333 | 1.597340343 | transcription initiation factor brf1 subunit-like protein [Nosema ceranae] |
| NBO_353g0005 | 131.3333333 | 339.2333333 | 1.369044808 | hypothetical protein NBO_353g0005 [Nosema bombycis CQ1] |
| NBO_974g0001 | 3.43 | 27.62666667 | 3.009781021 | Polysaccharide deacetylase [Nosema bombycis CQ1] |
| NBO_1344g0003 | 50.15333333 | 135.53 | 1.434194737 | magnesium transporter alr2 [Nosema ceranae] |
| NBO_560g0001 | 6.233333333 | 22.96333333 | 1.881256308 | DNA-binding protein SMUBP-2 [Nosema bombycis CQ1] |
| MSTRG.7314 | 2.616666667 | 15.92 | 2.605038277 | transposable element [Pseudoloma neurophilia] |
| NBO_33gi001 | 59.04666667 | 27.3 | -1.112954667 | Protein PNS1, partial [Nosema bombycis CQ1] |
| MSTRG.6740 | 9.176666667 | 34.89 | 1.926771488 | - |
| NBO_32g0034 | 87.13333333 | 187.0466667 | 1.102101616 | DNA polymerase kappa [Nosema bombycis CQ1] |
| NBO_1140g0001 | 88.68666667 | 206.09 | 1.216485375 | Synaptobrevin-like protein [Nosema bombycis CQ1] |
| NBO_44g0011 | 22.72666667 | 71.56 | 1.654767289 | Cell division control protein 50, partial [Nosema bombycis CQ1] |
| NBO_281g0003 | 152.7266667 | 341.13 | 1.159369651 | hypothetical protein NBO_281g0003 [Nosema bombycis CQ1] |
| MSTRG.5484 | 7.143333333 | 20.43333333 | 1.516255224 | krab-a domain-containing protein [Nosema apis BRL 01] |
| MSTRG.2352 | 2.58 | 12.28666667 | 2.251650599 | pol polyprotein [Nosema bombycis] |
| NBO_1054gi001 | 6.393333333 | 58.64666667 | 3.197408892 | T complex protein 1 subunit beta [Nosema bombycis CQ1] |
| MSTRG.9337 | 31.38 | 83.17666667 | 1.406333517 | piggybac-derived 2 (agap012114-pa) [Nosema apis BRL 01] |
| NBO_76g0013 | 198.0966667 | 98.90666667 | -1.002064932 | hypothetical protein NBO_76g0013 [Nosema bombycis CQ1] |
| NBO_29g0015 | 120.2266667 | 52.82666667 | -1.186418641 | Phosphoacetylglucosamine mutase [Nosema bombycis CQ1] |
| NBO_1345gi001 | 94.46666667 | 195.15 | 1.046706205 | enolase [Nosema bombycis CQ1] |
| NBO_1082g0001 | 6.81 | 54.57666667 | 3.00255758 | endonuclease [Nosema bombycis CQ1] |
| NBO_480g0001 | 1.173333333 | 17.5 | 3.898670089 | hypothetical protein NBO_480g0001 [Nosema bombycis CQ1] |
| NBO_20g0004 | 2.546666667 | 26.34 | 3.370573303 | Bifunctional xylanase/deacetylase [Nosema bombycis CQ1] |
| MSTRG.1871 | 2.85 | 12.13666667 | 2.090338416 | DNA helicase PIF1/RRM3 [Trachipleistophora hominis] |
| NBO_10g0001 | 47.42 | 141.5 | 1.577234485 | dna-directed rna polymerase ii subunit rpb7 [Nosema ceranae] |
| NBO_81g0019 | 0.863333333 | 40.11666667 | 5.538140234 | protein fam72a [Nosema apis BRL 01] |
| NBO_1110g0002 | 39.96666667 | 12.07666667 | -1.726574948 | Protein transport protein sec31 [Nosema bombycis CQ1] |
| NBO_1024g0001 | 276.84 | 559.45 | 1.014956788 | DNA-directed RNA polymerase II subunit RPB3 [Nosema bombycis CQ1] |
| NBO_6g0033 | 110.51 | 278.2066667 | 1.331980067 | NEDD8-conjugating enzyme UBC12 [Nosema bombycis CQ1] |
| NBO_460g0011 | 101.1966667 | 218.54 | 1.110735595 | hypothetical protein NBO_460g0011 [Nosema bombycis CQ1] |
| NBO_13g0031 | 182.98 | 367.2033333 | 1.004893187 | eukaryotic translation initiation factor 4E [Encephalitozoon romaleae SJ-2008] |
| MSTRG.4557 | 1.906666667 | 10.50666667 | 2.462180483 | krab-a domain-containing protein 2-like protein [Nosema ceranae] |
| NBO_418gi001 | 48.31666667 | 106.94 | 1.146208749 | integral membrane protein [Nosema bombycis CQ1] |
| NBO_33g0017 | 73.96 | 33.84333333 | -1.127873554 | hypothetical protein NBO_33g0017 [Nosema bombycis CQ1] |
| NBO_404g0004 | 25.44 | 74.15666667 | 1.543477726 | Serum response factor [Nosema bombycis CQ1] |
| MSTRG.8648 | 26.72666667 | 54.51 | 1.028241004 | glucose transporter type 3 [Nosema bombycis CQ1] |
| NBO_6g0060 | 10.81 | 45.21 | 2.064275395 | hypothetical protein NBO_6g0060 [Nosema bombycis CQ1] |
| NBO_60g0018 | 8.553333333 | 31.95666667 | 1.901558262 | Polysaccharide deacetylase [Nosema bombycis CQ1] |
| NBO_10g0034 | 71.25333333 | 171.6233333 | 1.268216298 | hypothetical protein NBO_10g0034 [Nosema bombycis CQ1] |
| NBO_462g0003 | 106.4966667 | 213.01 | 1.000112886 | hypothetical protein NBO_462g0003 [Nosema bombycis CQ1] |
| NBO_74g0004 | 44.66333333 | 90.59333333 | 1.020313961 | small-conductance mechanosensitive channel protein [Nosema ceranae] |
| NBO_417g0011 | 34.39666667 | 78.12333333 | 1.183484745 | Solute carrier family 35 member C2, partial [Nosema bombycis CQ1] |
| NBO_10g0027 | 51.29 | 143.3733333 | 1.483027239 | hypothetical protein NBO_10g0027 [Nosema bombycis CQ1] |
| NBO_10g0041 | 54.02 | 155.76 | 1.527759244 | hypothetical protein NBO_10g0041 [Nosema bombycis CQ1] |
| NBO_401g0004 | 5.553333333 | 68.61 | 3.626992966 | Homeobox protein [Nosema bombycis CQ1] |
| NBO_28g0061 | 100.7866667 | 207.7233333 | 1.043358488 | Cleavage and polyadenylation specificity factor subunit 4 [Nosema bombycis CQ1] |
| MSTRG.1578 | 6.106666667 | 29.69666667 | 2.281844001 | hypothetical protein NAPIS_ORF01137 [Nosema apis BRL 01] |
| NBO_1230g0001 | 51.85666667 | 152.0566667 | 1.552007692 | hypothetical protein NBO_1230g0001 [Nosema bombycis CQ1] |
| NBO_81g0012 | 0.001 | 3.19 | 11.63934071 | MADS domain containing protein, partial [Nosema bombycis CQ1] |
| MSTRG.4001 | 22.74666667 | 71.78333333 | 1.657993772 | ATP-dependent DNA helicase PIF1 [Hypsizygus marmoreus] |
| NBO_463g0002 | 77.48666667 | 160.7833333 | 1.053097877 | Ricin B lectin [Nosema bombycis CQ1] |
| NBO_568g0001 | 71.37 | 33.84666667 | -1.076304013 | Tristetraproline [Nosema bombycis CQ1] |
| NBO_11g0052 | 123.6 | 254.22 | 1.040398791 | Flap endonuclease 1-A [Nosema bombycis CQ1] |
| NBO_20g0024 | 40.63333333 | 17.18666667 | -1.241373957 | cdc73-like rna polymerase ii accessory factor [Nosema ceranae] |
| NBO_66g0051 | 38.39333333 | 114.7666667 | 1.579775954 | hypothetical protein NBO_66g0051 [Nosema bombycis CQ1] |
| NBO_10g0110 | 27.30666667 | 97.94333333 | 1.842694082 | DNA-directed RNA polymerases I and III subunit RPAC2 [Nosema bombycis CQ1] |
| NBO_46g0002 | 52.72666667 | 122.3666667 | 1.214605915 | hypothetical protein NBO_46g0002 [Nosema bombycis CQ1] |
| NBO_6g0028 | 67.9 | 141.99 | 1.064305848 | hypothetical protein NBO_6g0028 [Nosema bombycis CQ1] |
| NBO_845g0001 | 31.33666667 | 66.88666667 | 1.093866923 | hypothetical protein NBO_845g0001 [Nosema bombycis CQ1] |
| NBO_42g0007 | 52.76 | 123.88 | 1.231426818 | rna polymerase ii ctd phosphatase [Nosema ceranae] |
| NBO_55g0012 | 6.666666667 | 36.35666667 | 2.447182434 | hypothetical protein NBO_55g0012 [Nosema bombycis CQ1] |
| NBO_114g0004 | 6.25 | 0.23 | -4.764150423 | Zinc phosphodiesterase ELAC protein 2 [Nosema bombycis CQ1] |
| NBO_929g0005 | 57.6 | 142.5966667 | 1.307799541 | hypothetical protein NBO_929g0005 [Nosema bombycis CQ1] |
| MSTRG.4521 | 1.366666667 | 6.6 | 2.271804615 | pol polyprotein [Nosema bombycis] |
| NBO_55g0006 | 31.97666667 | 79.13666667 | 1.307326742 | Cyclin-A1-2, partial [Nosema bombycis CQ1] |
| NBO_675g0001 | 63.07 | 142.4366667 | 1.175294741 | hypothetical protein NBO_675g0001 [Nosema bombycis CQ1] |
| MSTRG.8131 | 3.986666667 | 11.89666667 | 1.577302512 | pol polyprotein [Nosema bombycis] |
| NBO_81g0002 | 74.85333333 | 27.83666667 | -1.427080099 | MADS domain containing protein [Nosema pernyi] |
| NBO_360g0001 | 62.09 | 142.9633333 | 1.203212341 | transcription initiation factor TFIID 20/15kDa subunit [Nosema bombycis CQ1] |
| NBO_58g0022 | 6.536666667 | 0.38 | -4.104483806 | hypothetical protein NBO_58g0022 [Nosema bombycis CQ1] |
| NBO_53gi001 | 48.83 | 24.29 | -1.007405288 | transcriptional activator [Nosema bombycis CQ1] |
| NBO_1530gi001 | 19.77 | 63.36666667 | 1.680411255 | tryptophanyl tRNA synthetase [Nosema bombycis CQ1] |
| MSTRG.1369 | 9.57 | 22.91 | 1.259386629 | pol polyprotein [Nosema bombycis] |
| NBO_1552gi001 | 83.16333333 | 169.5433333 | 1.027634567 | poly(A) polymerase type 1, partial [Nosema bombycis CQ1] |
| MSTRG.1400 | 3.656666667 | 16.96666667 | 2.214102131 | pol polyprotein [Nosema bombycis] |
| NBO_929g0004 | 26.47666667 | 84.44666667 | 1.673318975 | Anamorsin [Nosema bombycis CQ1] |
| MSTRG.2750 | 5.07 | 14.38333333 | 1.504340406 | pol polyprotein [Nosema bombycis] |
| MSTRG.5613 | 6.816666667 | 15.67666667 | 1.201480489 | serine protease inhibitor 106 [Nosema bombycis] |
| NBO_38g0034 | 150.8866667 | 74.91333333 | -1.010170903 | protein kinase domain-containing protein [Nosema pernyi] |
| NBO_32g0021 | 44.61666667 | 103.26 | 1.210626865 | hypothetical protein NBO_32g0021 [Nosema bombycis CQ1] |
| NBO_389g0001 | 19.2 | 9.106666667 | -1.076111328 | U3 small nucleolar RNA-associated protein 12 [Nosema bombycis CQ1] |
| NBO_366gi001 | 27.20333333 | 60.55333333 | 1.154422938 | phosphate-transporting ATPase [Trachipleistophora hominis] |
| NBO_1232g0001 | 28.29 | 64.85333333 | 1.196888551 | hypothetical protein NBO_1232g0001 [Nosema bombycis CQ1] |
| NBO_76g0012 | 35.91666667 | 81.68333333 | 1.185388277 | type 2 peptidyl-tRNA hydrolase [Encephalitozoon romaleae SJ-2008] |
| NBO_369g0001 | 32.79666667 | 76.42333333 | 1.220463993 | exosome complex exonuclease rrp4, partial [Nosema bombycis CQ1] |
| NBO_37g0005 | 20.81666667 | 54.58333333 | 1.39072143 | Cystine/glutamate transporter [Nosema bombycis CQ1] |
| NBO_81g0015 | 11.74333333 | 36.54666667 | 1.637897853 | Serum response factor [Nosema bombycis CQ1] |
| NBO_359g0004 | 13.26333333 | 1.466666667 | -3.17683047 | hypothetical protein NBO_359g0004 [Nosema bombycis CQ1] |
| NBO_9g0005 | 16.66333333 | 35.38666667 | 1.086528844 | 60S ribosomal protein L10 [Nosema bombycis] |
| NBO_69g0004 | 24.62666667 | 52.35 | 1.087968172 | DNA polymerase alpha subunit B, partial [Nosema bombycis CQ1] |
| NBO_648gi001 | 4.306666667 | 25.82 | 2.583845431 | serine/threonine protein kinase, partial [Nosema bombycis CQ1] |
| NBO_2gi003 | 38.90666667 | 105.11 | 1.433810644 | DNA-directed RNA polymerases I and III subunit RPAC2 [Nosema bombycis CQ1] |
| NBO_4g0061 | 39.24 | 14.14 | -1.472542921 | hypothetical protein NBO_4g0061 [Nosema bombycis CQ1] |
| NBO_503g0002 | 39.15 | 9.84 | -1.992282087 | hypothetical protein NBO_503g0002 [Nosema bombycis CQ1] |
| NBO_10g0023 | 58.94666667 | 133.4466667 | 1.178781131 | hypothetical protein NBO_10g0023 [Nosema bombycis CQ1] |
| NBO_1024g0002 | 35.24333333 | 83.23666667 | 1.23986881 | N(5)-glutamine methyltransferase MTQ2 [Nosema bombycis CQ1] |
| NBO_174g0001 | 35.01333333 | 13.9 | -1.332819533 | hypothetical protein NBO_174g0001 [Nosema bombycis CQ1] |
| NBO_25g0006 | 10.94333333 | 63.69666667 | 2.541165627 | small nuclear ribonucleoprotein F [Nosema bombycis CQ1] |
| NBO_3g0049 | 95.33666667 | 211.41 | 1.148940531 | hypothetical protein NBO_3g0049 [Nosema bombycis CQ1] |
| NBO_92g0005 | 0.48 | 8.083333333 | 4.073844031 | Beta-lactamase, class A [Nosema bombycis CQ1] |
| NBO_54g0011 | 40.41 | 101.2466667 | 1.325090154 | Ribose-5-phosphate isomerase A [Nosema bombycis CQ1] |
| NBO_318g0001 | 21.27666667 | 58.47333333 | 1.458506689 | hypothetical protein NBO_318g0001 [Nosema bombycis CQ1] |
| NBO_46g0007 | 14.3 | 60.28333333 | 2.075744045 | hypothetical protein NBO_380g0001 [Nosema bombycis CQ1] |
| NBO_28g0065 | 16.07 | 2.283333333 | -2.815156537 | hypothetical protein NBO_28g0065 [Nosema bombycis CQ1] |
| NBO_552g0003 | 5.306666667 | 36.21666667 | 2.770775934 | Homeobox protein HD-4 [Nosema bombycis CQ1] |
| NBO_447g0002 | 9.79 | 25.16333333 | 1.361942281 | Meiosis-specific protein HOP1 [Nosema bombycis CQ1] |
| NBO_1239g0006 | 30.66666667 | 66.51666667 | 1.117044513 | ATP-binding domain protein 4 [Nosema bombycis CQ1] |
| NBO_34g0043 | 19.8 | 47.08 | 1.24961389 | Serine/threonine-protein phosphatase 4 catalytic subunit [Nosema bombycis CQ1] |
| NBO_11g0061 | 26.08333333 | 70.02 | 1.42463881 | Serine/threonine-protein kinase crk1 [Nosema bombycis CQ1] |
| NBO_166g0001 | 5.046666667 | 19.48333333 | 1.948837819 | Polysaccharide deacetylase [Nosema bombycis CQ1] |
| MSTRG.7603 | 0.876666667 | 5.523333333 | 2.655438898 | - |
| NBO_338g0001 | 19.64 | 42.94333333 | 1.12863925 | hypothetical protein NBO_338g0001 [Nosema bombycis CQ1] |
| NBO_53g0022 | 15.58333333 | 38.21 | 1.293946393 | DRAP deaminase [Nosema bombycis CQ1] |
| MSTRG.1618 | 2.446666667 | 8.95 | 1.87107012 | transposase [Nosema bombycis] |
| NBO_841g0001 | 19.94 | 5.81 | -1.779055341 | hypothetical protein NBO_841g0001 [Nosema bombycis CQ1] |
| NBO_13g0005 | 14.95666667 | 48.01666667 | 1.682746571 | longevity assurance protein 1 [Nosema bombycis CQ1] |
| NBO_1246g0001 | 64.12666667 | 129.8566667 | 1.01792376 | Serine/threonine-protein phosphatase PP2A catalytic subunit [Nosema bombycis CQ1] |
| NBO_423g0001 | 15.03 | 34.49666667 | 1.198611955 | NADPH-dependent diflavin oxidoreductase 1 [Nosema bombycis CQ1] |
| NBO_4g0016 | 67.48666667 | 140.4866667 | 1.057758811 | hypothetical protein NBO_4g0016 [Nosema bombycis CQ1] |
| NBO_770g0002 | 30.81333333 | 66.2 | 1.103276458 | Ubiquitin thioesterase otubain-like protein [Nosema bombycis CQ1] |
| NBO_1170gi001 | 2097.113333 | 4389.616667 | 1.065690128 | HSP 101 related protein, partial [Nosema bombycis CQ1] |
| NBO_85g0001 | 11.19 | 37.40666667 | 1.741085376 | Ubiquitin-like modifier-activating enzyme 1 Y, partial [Nosema bombycis CQ1] |
| NBO_452g0002 | 32.37666667 | 75.86 | 1.228384911 | Sld5 domain-containing protein [Rhizoctonia solani AG-1 IA] |
| NBO_6g0088 | 31.38333333 | 64.50333333 | 1.039375122 | hypothetical protein NBO_6g0088 [Nosema bombycis CQ1] |
| NBO_41g0030 | 26.57666667 | 54.23666667 | 1.029108346 | hypothetical protein NBO_41g0030 [Nosema bombycis CQ1] |
| NBO_64g0035 | 47.17666667 | 95.14 | 1.011978539 | transcription elongation factor s-ii [Nosema ceranae] |
| NBO_15g0005 | 58.51 | 118.33 | 1.016060761 | negative regulator of transcription [Encephalitozoon cuniculi GB-M1] |
| NBO_27gi003 | 20.3 | 44.05 | 1.117662292 | hypothetical protein NBO_27gi003 [Nosema bombycis CQ1] |
| NBO_459g0003 | 7.233333333 | 21.43666667 | 1.567348047 | Meiosis-specific protein HOP1 [Nosema bombycis CQ1] |
| NBO_16g0010 | 21.38333333 | 61.36333333 | 1.520890089 | 2Fe-2S ferredoxin [Nosema bombycis CQ1] |
| NBO_1276g0001 | 2.053333333 | 16.03 | 2.96473467 | tRNA acetyltransferase TAN1 [Nematocida displodere] |
| NBO_757g0002 | 5.12 | 26.87666667 | 2.392138506 | Beta tubulin, autoregulation binding site [Nosema bombycis CQ1] |
| NBO_665g0002 | 5.243333333 | 28.69666667 | 2.452326997 | hypothetical protein NBO_665g0002 [Nosema bombycis CQ1] |
| NBO_483g0001 | 7.193333333 | 19.79666667 | 1.460525168 | hypothetical protein NBO_483g0001 [Nosema bombycis CQ1] |
| NBO_64g0032 | 29.79333333 | 60.37333333 | 1.018921913 | YjeF family domain-containing protein [Anncaliia algerae PRA339] |
| NBO_503g0004 | 8.17 | 44.34666667 | 2.440417684 | protein transport protein yos1 [Nosema ceranae] |
| NBO_61g0013 | 35.97 | 91.32 | 1.3441367 | Frataxin, mitochondrial [Nosema bombycis CQ1] |
| MSTRG.14040 | 8.026666667 | 17.71333333 | 1.141962836 | hypothetical protein NBO_11g0042 [Nosema bombycis CQ1] |

**Supplementary Table 3 Differentially expressed genes of silkworm between embryos and larvae during *N. bombycis* infection**

| **Differentially expressed genes of silkworm between 5-day embryos and 1-day larvae during *N. bombycis* infection** | | | | | | |
| --- | --- | --- | --- | --- | --- | --- |
| ID | | I-E5_mean | | I-L1_mean | log2(fc) | Description |
| bmsk0006458 | | 0.001 | | 54.77666667 | 15.74127386 | phosphoglycolate phosphatase-like [Plutella xylostella] |
| bmsk0000346 | | 0.001 | | 3.686666667 | 11.84810126 | abnormal cell migration protein 10 isoform X2 |
| bmsk0005976 | | 0.001 | | 2.36 | 11.20457114 | gelsolin |
| bmsk0002006 | | 0.001 | | 2.136666667 | 11.06114614 | tensin isoform X4 |
| bmsk0004519 | | 0.001 | | 1.823333333 | 10.83236262 | cytochrome P450, family 315, subfamily a, polypeptide 1 isoform X1 |
| bmsk0013246 | | 0.001 | | 1.483333333 | 10.53462712 | - |
| bmsk0003933 | | 0.001 | | 1.476666667 | 10.52812848 | - |
| bmsk0013590 | | 0.001 | | 0.836666667 | 9.708509148 | esterase FE4-like isoform X1 |
| bmsk0015307 | | 0.001 | | 0.833333333 | 9.702749879 | - |
| bmsk0000436 | | 0.001 | | 0.8 | 9.64385619 | uncharacterized protein LOC101737027 |
| bmsk0013647 | | 0.001 | | 0.78 | 9.607330314 | nitrilase homolog 1 isoform X2 |
| bmsk0012576 | | 0.001 | | 0.736666667 | 9.524868154 | XP 012550723.1 band 7 protein AGAP004871 isoform X1 |
| bmsk0008377 | | 0.001 | | 0.726666667 | 9.505149919 | - |
| bmsk0012405 | | 0.001 | | 0.67 | 9.388017285 | - |
| bmsk0005711 | | 0.001 | | 0.583333333 | 9.188176706 | inducible nitric oxide synthase-like protein (iNOS-LP), partial |
| bmsk0007506 | | 0.001 | | 0.516666667 | 9.013089999 | - |
| bmsk0010954 | | 0.001 | | 0.483333333 | 8.916874684 | AEL88625.1 amino acid transporter-like protein |
| bmsk0002836 | | 0.001 | | 0.45 | 8.813781191 | hypothetical protein RR46 11993 [Papilio xuthus] |
| bmsk0005927 | | 0.001 | | 0.44 | 8.781359714 | gelsolin, cytoplasmic-like [Papilio xuthus] |
| bmsk0008572 | | 0.001 | | 0.433333333 | 8.759333407 | uncharacterized protein LOC106719573 [Papilio machaon] |
| bmsk0011625 | | 0.001 | | 0.41 | 8.6794801 | mR transport regulator 3 isoform X3 |
| bmsk0001600 | | 0.001 | | 0.403333333 | 8.655828831 | serpin-17 |
| bmsk0007982 | | 0.001 | | 0.386666667 | 8.594946589 | prothoracico static peptide, partial |
| bmsk0015328 | | 0.001 | | 0.366666667 | 8.518325308 | - |
| bmsk0015119 | | 0.001 | | 0.34 | 8.409390936 | facilitated trehalose transporter Tret1-like |
| bmsk0008719 | | 0.001 | | 0.34 | 8.409390936 | probable multidrug resistance-associated protein lethal(2)03659 |
| bmsk0014244 | | 0.001 | | 0.316666667 | 8.306821202 | XP 004923181.2 lysosome-associated membrane glycoprotein 1 |
| bmsk0000353 | | 0.001 | | 0.306666667 | 8.26052755 | uncharacterized protein LOC101742649 |
| bmsk0000455 | | 0.001 | | 0.303333333 | 8.244760234 | hypodermin-A |
| bmsk0005779 | | 0.001 | | 0.3 | 8.22881869 | RhoGEF domain |
| bmsk0000665 | | 0.001 | | 0.293333333 | 8.196397213 | Endonuclease/Exonuclease/phosphatase family |
| bmsk0007679 | | 0.001 | | 0.29 | 8.17990909 | - |
| bmsk0004766 | | 0.001 | | 0.283333333 | 8.14635653 | - |
| bmsk0001289 | | 0.001 | | 0.266666667 | 8.058893689 | uncharacterized protein LOC101743136 |
| bmsk0000435 | | 0.001 | | 0.263333333 | 8.040746342 | uncharacterized protein LOC101737027 |
| bmsk0012932 | | 0.001 | | 0.253333333 | 7.984893108 | signal recognition particle subunit SRP72 [Amyelois transitella] |
| bmsk0010641 | | 0.001 | | 0.25 | 7.965784285 | uncharacterized protein LOC106133664 [Amyelois transitella] |
| bmsk0012735 | | 0.001 | | 0.25 | 7.965784285 | cuticular protein RR-1 motif 40 precursor |
| bmsk0006922 | | 0.001 | | 0.243333333 | 7.926790153 | nose resistant to fluoxetine protein 6-like |
| bmsk0015926 | | 0.001 | | 0.24 | 7.906890596 | uncharacterized protein LOC106110594 [Papilio polytes] |
| bmsk0005456 | | 0.001 | | 0.223333333 | 7.803054785 | uncharacterized protein LOC105842218 |
| bmsk0013333 | | 0.001 | | 0.193333333 | 7.594946589 | XP 021207875.1 prominin-1 |
| bmsk0013912 | | 0.001 | | 0.186666667 | 7.544320516 | Cytochrome P450 |
| bmsk0011970 | | 0.001 | | 0.18 | 7.491853096 | XP 004925452.2 uncharacterized protein LOC101737101 isoform X1 |
| bmsk0006096 | | 0.001 | | 0.176666667 | 7.464886049 | uncharacterized protein LOC105841967 |
| bmsk0012317 | | 0.001 | | 0.156666667 | 7.291554446 | XP 021202909.1 cytochrome P450 6B1 |
| bmsk0004156 | | 0.001 | | 0.156666667 | 7.291554446 | uncharacterized protein LOC105841511 |
| bmsk0003853 | | 0.006666667 | | 0.993333333 | 7.21916852 | cuticular protein RR-2 motif 70 precursor |
| bmsk0007366 | | 0.001 | | 0.146666667 | 7.196397213 | facilitated trehalose transporter Tret1-like isoform X2 |
| bmsk0009351 | | 0.001 | | 0.143333333 | 7.163230349 | small G protein signaling modulator 2-like isoform X4 [Papilio polytes] |
| bmsk0013759 | | 0.001 | | 0.14 | 7.129283017 | - |
| bmsk0000936 | | 0.001 | | 0.133333333 | 7.058893689 | ced-6 protein isoform X1 |
| bmsk0010009 | | 0.001 | | 0.126666667 | 6.984893108 | uncharacterized protein LOC106119184 [Papilio xuthus] |
| bmsk0013887 | | 0.001 | | 0.12 | 6.906890596 | Ryanodine Receptor TM 4-6 |
| bmsk0014497 | | 0.001 | | 0.12 | 6.906890596 | juvenile hormone esterase 1 precursor |
| bmsk0004403 | | 0.001 | | 0.11 | 6.781359714 | uncharacterized protein LOC101744340 |
| bmsk0002789 | | 0.001 | | 0.11 | 6.781359714 | uncharacterized protein LOC101739696 |
| bmsk0013692 | | 0.001 | | 0.103333333 | 6.691161905 | XP 021203517.1 uncharacterized protein LOC101746620 |
| bmsk0006019 | | 0.006666667 | | 0.663333333 | 6.636624621 | uncharacterized protein LOC101741752 isoform X5 |
| bmsk0014312 | | 0.001 | | 0.096666667 | 6.594946589 | XP 012546088.1 uncharacterized protein LOC101743782 |
| bmsk0007271 | | 0.001 | | 0.086666667 | 6.437405312 | ATP-binding cassette sub-family G member 1-like isoform X1 |
| bmsk0006983 | | 0.001 | | 0.083333333 | 6.380821784 | ATP-binding cassette sub-family G member 1 |
| bmsk0014910 | | 0.001 | | 0.08 | 6.321928095 | protein phosphatase 1 regulatory subunit 21 |
| bmsk0011488 | | 0.001 | | 0.08 | 6.321928095 | synaptic vesicle glycoprotein 2C-like |
| bmsk0013429 | | 0.001 | | 0.076666667 | 6.26052755 | uncharacterized protein LOC106136069 isoform X2 [Amyelois transitella] |
| bmsk0003473 | | 0.001 | | 0.076666667 | 6.26052755 | angiotensin-converting enzyme |
| bmsk0009623 | | 0.001 | | 0.066666667 | 6.058893689 | cis, cis-muconate transporter protein |
| bmsk0014859 | | 0.013333333 | | 0.803333333 | 5.912889336 | XP 012544115.1 cuticular protein RR-1 motif 49 isoform X1 |
| bmsk0010782 | | 0.001 | | 0.06 | 5.906890596 | uncharacterized protein LOC101744177 isoform X1 |
| bmsk0011669 | | 0.016666667 | | 0.956666667 | 5.842978832 | ACN81326.1 Bm122 |
| bmsk0010660 | | 0.026666667 | | 1.506666667 | 5.820178962 | XP 004928296.1 brachyurin |
| bmsk0004176 | | 0.02 | | 1.006666667 | 5.653442239 | metabotropic glutamate receptor 2-like [Papilio xuthus] |
| bmsk0012666 | | 0.001 | | 0.05 | 5.64385619 | XP 021207520.1 sodium channel protein Nach-like |
| bmsk0007615 | | 0.001 | | 0.05 | 5.64385619 | sterol regulatory element-binding protein 2 [Amyelois transitella] |
| bmsk0007489 | | 0.001 | | 0.05 | 5.64385619 | phosphatidylinositol 4-phosphate 3-kinase C2 domain-containing subunit alpha isoform X3 |
| bmsk0012717 | | 0.001 | | 0.046666667 | 5.544320516 | XP 021207484.1 PR domain zinc finger protein 1 isoform X2 |
| bmsk0004184 | | 0.016666667 | | 0.593333333 | 5.153805336 | cationic amino acid transporter 2 [Papilio machaon] |
| bmsk0013569 | | 0.001 | | 0.033333333 | 5.058893689 | XP 021206639.1 uncharacterized protein LOC101739142 |
| bmsk0013250 | | 0.126666667 | | 3.676666667 | 4.859289562 | - |
| bmsk0008176 | | 0.01 | | 0.273333333 | 4.772589504 | uncharacterized protein LOC101745119 |
| bmsk0000067 | | 0.003333333 | | 0.09 | 4.754887502 | titin homolog |
| bmsk0013474 | | 0.001 | | 0.026666667 | 4.736965594 | vacuolar protein sorting-associated protein 13A-like |
| bmsk0007259 | | 0.001 | | 0.026666667 | 4.736965594 | retinol dehydrogenase 12-like [Anolis carolinensis] |
| bmsk0003069 | | 0.003333333 | | 0.08 | 4.584962501 | uncharacterized protein LOC101737288 |
| bmsk0006521 | | 0.083333333 | | 1.683333333 | 4.336283388 | - |
| bmsk0013248 | | 0.32 | | 6.396666667 | 4.321176495 | - |
| bmsk0002162 | | 0.026666667 | | 0.51 | 4.257387843 | whirlin |
| bmsk0007046 | | 0.02 | | 0.376666667 | 4.235216462 | - |
| bmsk0013066 | | 0.033333333 | | 0.616666667 | 4.209453366 | ecdysteroid regulated protein [Danaus plexippus] |
| bmsk0015600 | | 0.016666667 | | 0.306666667 | 4.201633861 | KWMTBOMO16015 |
| bmsk0003132 | | 0.133333333 | | 2.406666667 | 4.173926932 | - |
| bmsk0008289 | | 0.02 | | 0.36 | 4.169925001 | hypothetical protein KGM 07463 [Danaus plexippus] |
| bmsk0007396 | | 0.01 | | 0.166666667 | 4.058893689 | organic cation transporter protein-like isoform X3 |
| bmsk0006043 | | 0.006666667 | | 0.106666667 | 4 | sericin 1-like isoform X3 |
| bmsk0007415 | | 0.006666667 | | 0.106666667 | 4 | neuropeptide receptor A9 |
| bmsk0010002 | | 0.083333333 | | 1.243333333 | 3.89917563 | inactive rhomboid protein 1, partial |
| bmsk0001757 | | 0.023333333 | | 0.34 | 3.86507042 | - |
| bmsk0013201 | | 0.016666667 | | 0.24 | 3.847996907 | XP 004921779.2 solute carrier family 22 member 16-like |
| mstrg.6241 | | 0.096666667 | | 1.316666667 | 3.767727848 | PREDICTED: hemicentin-1-like [Bombyx mori] |
| bmsk0012136 | | 0.006666667 | | 0.09 | 3.754887502 | - |
| bmsk0009191 | | 0.003333333 | | 0.043333333 | 3.700439718 | serine proteinase stubble-like [Amyelois transitella] |
| bmsk0005202 | | 0.063333333 | | 0.753333333 | 3.572251449 | putative cuticle protein |
| bmsk0000572 | | 0.033333333 | | 0.393333333 | 3.560714954 | C2 domain |
| bmsk0003511 | | 0.57 | | 6.54 | 3.520256811 | Cecropin family |
| bmsk0013300 | | 0.09 | | 1.023333333 | 3.507207343 | Uncharacterized protein OBRU01 01183 [Operophtera brumata] |
| bmsk0010808 | | 0.07 | | 0.79 | 3.496425826 | Rh-like protein |
| bmsk0015269 | | 0.04 | | 0.443333333 | 3.470319935 | dopamine receptor-1 |
| bmsk0001826 | | 0.113333333 | | 1.253333333 | 3.46712601 | keratin, type I cytoskeletal 9 |
| bmsk0007504 | | 0.01 | | 0.106666667 | 3.415037499 | lipopolysaccharide-induced tumor necrosis factor-alpha factor homolog |
| bmsk0004776 | | 0.04 | | 0.423333333 | 3.403722186 | - |
| bmsk0004641 | | 0.053333333 | | 0.55 | 3.366322214 | - |
| bmsk0013332 | | 0.016666667 | | 0.166666667 | 3.321928095 | XP 021207885.1 uncharacterized protein LOC110386324 |
| bmsk0003618 | | 0.033333333 | | 0.326666667 | 3.292781749 | neuropeptide FF receptor 2-like |
| bmsk0009914 | | 0.01 | | 0.096666667 | 3.273018494 | SOCS box |
| bmsk0006122 | | 0.036666667 | | 0.343333333 | 3.227068909 | - |
| bmsk0004995 | | 0.03 | | 0.28 | 3.222392421 | pH-sensitive chloride channel 1 isoform 48 |
| bmsk0004480 | | 0.3 | | 2.723333333 | 3.182339172 | - |
| bmsk0009497 | | 0.026666667 | | 0.236666667 | 3.14974712 | - |
| bmsk0014651 | | 0.123333333 | | 1.056666667 | 3.098885665 | uncharacterized protein LOC101741141 isoform X1 |
| bmsk0014247 | | 0.116666667 | | 0.966666667 | 3.050626073 | ABW86946.1 sex peptide receptor |
| bmsk0000273 | | 0.58 | | 4.733333333 | 3.028731719 | putative fatty acyl-CoA reductase CG5065 |
| bmsk0008353 | | 0.9 | | 7.34 | 3.027783157 | transcription factor E74 |
| bmsk0012786 | | 4.52 | | 36.26666667 | 3.004249473 | uncharacterized protein LOC101740638 isoform X1 |
| bmsk0015095 | | 0.15 | | 1.203333333 | 3.004001931 | PIF1-like helicase |
| bmsk0009197 | | 0.066666667 | | 0.53 | 2.99095486 | uncharacterized protein LOC110386580 |
| bmsk0004777 | | 0.066666667 | | 0.526666667 | 2.981852653 | katanin p60 ATPase-containing subunit A-like 1 |
| bmsk0004848 | | 0.776666667 | | 6.1 | 2.973441789 | peptidoglycan recognition protein S2 |
| bmsk0015356 | | 0.056666667 | | 0.426666667 | 2.912537159 | probable sodium/potassium/calcium exchanger CG1090 |
| bmsk0005905 | | 0.136666667 | | 1.02 | 2.899835838 | tubulin beta chain isoform X2 |
| bmsk0006288 | | 0.026666667 | | 0.19 | 2.832890014 | dyslexia susceptibility 1 candidate gene 1 protein |
| bmsk0000679 | | 0.073333333 | | 0.52 | 2.8259706 | - |
| bmsk0003491 | | 0.133333333 | | 0.926666667 | 2.797012978 | - |
| bmsk0007653 | | 0.116666667 | | 0.8 | 2.777607579 | uncharacterized protein LOC101740144 isoform X2 |
| bmsk0008173 | | 0.11 | | 0.753333333 | 2.775784843 | soluble guanylyl cyclae alpha-1 subunit isoform X1 |
| bmsk0001950 | | 0.053333333 | | 0.363333333 | 2.768184325 | uncharacterized protein LOC106116522 [Papilio xuthus] |
| bmsk0011925 | | 0.033333333 | | 0.223333333 | 2.744161096 | uncharacterized protein LOC106102902 [Papilio polytes] |
| bmsk0004692 | | 0.173333333 | | 1.086666667 | 2.648288436 | uncharacterized protein LOC106132144 [Amyelois transitella] |
| bmsk0009262 | | 0.05 | | 0.3 | 2.584962501 | aldo-keto reductase AKR2E4 isoform X1 |
| bmsk0005238 | | 0.1 | | 0.6 | 2.584962501 | - |
| bmsk0005821 | | 0.006666667 | | 0.04 | 2.584962501 | matrix metalloproteinase-25-like [Papilio machaon] |
| bmsk0001932 | | 0.103333333 | | 0.616666667 | 2.57718515 | adenylate cyclase type 3 |
| bmsk0012881 | | 0.156666667 | | 0.923333333 | 2.559153314 | uncharacterized protein LOC105841761 |
| bmsk0000066 | | 0.62 | | 3.65 | 2.557556343 | Immunoglobulin I-set domain |
| bmsk0009146 | | 0.056666667 | | 0.33 | 2.541893779 | Transmembrane protease serine 9 [Papilio xuthus] |
| bmsk0009621 | | 0.853333333 | | 4.943333333 | 2.534302882 | mitochondrial import inner membrane translocase subunit Tim22 [Papilio machaon] |
| bmsk0008318 | | 0.286666667 | | 1.653333333 | 2.527931556 | Kinesin motor domain |
| bmsk0006884 | | 0.013333333 | | 0.076666667 | 2.523561956 | glucose dehydrogenase [FAD, quinone]-like |
| bmsk0008756 | | 0.08 | | 0.443333333 | 2.470319935 | ras-like GTP-binding protein RhoL [Papilio xuthus] |
| bmsk0000446 | | 0.216666667 | | 1.193333333 | 2.461447964 | - |
| bmsk0012807 | | 0.056666667 | | 0.3 | 2.404390255 | carbohydrate sulfotransferase 11 [Papilio xuthus] |
| bmsk0002122 | | 0.986666667 | | 5.166666667 | 2.388599135 | hemocyte protein-glutamine gamma-glutamyltransferase-like [Amyelois transitella] |
| bmsk0006659 | | 0.076666667 | | 0.4 | 2.38332864 | GTP-binding protein Rhes |
| bmsk0015536 | | 0.033333333 | | 0.173333333 | 2.378511623 | XP 012553331.1 uncharacterized protein LOC101742438 |
| bmsk0014377 | | 0.91 | | 4.716666667 | 2.373829197 | Insect cuticle protein |
| bmsk0013481 | | 0.163333333 | | 0.836666667 | 2.35683371 | GRAM domain-containing protein 1B-like isoform X1 |
| bmsk0003461 | | 0.086666667 | | 0.443333333 | 2.354842717 | suppressor of lurcher protein 1 |
| bmsk0006537 | | 0.103333333 | | 0.526666667 | 2.349584438 | cuticular protein hypothetical 12 precursor |
| bmsk0012495 | | 0.36 | | 1.83 | 2.345774837 | uncharacterized protein LOC106709922 [Papilio machaon] |
| mstrg.14416 | | 0.19 | | 0.963333333 | 2.342035668 | PREDICTED: cuticular protein RR-1 motif 33 isoform X1 [Bombyx mori] |
| bmsk0000652 | | 0.203333333 | | 1.02 | 2.326650505 | Domain of unknown function (DUF4780) |
| bmsk0000654 | | 0.203333333 | | 1.02 | 2.326650505 | Domain of unknown function (DUF4780) |
| bmsk0013778 | | 0.016666667 | | 0.083333333 | 2.321928095 | dynamin-like 120 kDa protein, mitochondrial |
| bmsk0006474 | | 2.026666667 | | 10.12333333 | 2.32050368 | glutamate receptor ionotropic, NMDA 2B-like |
| bmsk0001880 | | 0.65 | | 3.233333333 | 2.314510623 | protein muscleblind isoform X6 |
| bmsk0006982 | | 0.126666667 | | 0.616666667 | 2.283453947 | neural cell adhesion molecule 1-like isoform X2 [Papilio xuthus] |
| bmsk0007535 | | 0.143333333 | | 0.696666667 | 2.281094377 | zinc transporter 1 [Papilio polytes] |
| bmsk0010491 | | 0.97 | | 4.606666667 | 2.247666557 | XP 021205119.1 xanthine dehydrogenase |
| bmsk0013146 | | 0.226666667 | | 1.07 | 2.238966646 | tyramine receptor |
| bmsk0012159 | | 0.253333333 | | 1.193333333 | 2.235888264 | XP 021205921.1 small conductance calcium-activated potassium channel protein-like |
| bmsk0002269 | | 0.14 | | 0.65 | 2.215012891 | cuticular protein RR-2 motif 60 precursor |
| bmsk0010762 | | 0.026666667 | | 0.123333333 | 2.209453366 | XP 004928354.1 uncharacterized protein LOC101736409 |
| bmsk0008609 | | 0.396666667 | | 1.833333333 | 2.208470045 | Amino acid permease |
| mstrg.13277 | | 0.193333333 | | 0.893333333 | 2.208108195 | PREDICTED: uncharacterized protein LOC101743025 [Bombyx mori] |
| bmsk0002671 | | 0.186666667 | | 0.85 | 2.186998515 | alpha-catulin isoform X4 |
| bmsk0005337 | | 0.036666667 | | 0.166666667 | 2.184424571 | armadillo repeat-containing protein gudu |
| bmsk0010507 | | 2.513333333 | | 11.35333333 | 2.175442006 | helix-loop-helix protein delilah-like [Amyelois transitella] |
| bmsk0012368 | | 0.116666667 | | 0.526666667 | 2.174497731 | ras-specific guanine nucleotide-releasing factor 1-like |
| bmsk0009892 | | 0.19 | | 0.85 | 2.161463423 | Baculovirus FP protein |
| bmsk0003554 | | 0.296666667 | | 1.326666667 | 2.16089119 | cubilin isoform X1 |
| mstrg.21039 | | 0.173333333 | | 0.773333333 | 2.157541277 | PREDICTED: NACHT domain- and WD repeat-containing protein 1 [Papilio xuthus] |
| bmsk0002065 | | 0.036666667 | | 0.163333333 | 2.155278225 | uncharacterized protein LOC106720006 isoform X2 [Papilio machaon] |
| bmsk0004788 | | 0.106666667 | | 0.473333333 | 2.14974712 | probable muscarinic acetylcholine receptor gar-2 isoform X1 |
| mstrg.19078 | | 0.123333333 | | 0.53 | 2.10342959 | PREDICTED: uncharacterized protein LOC101744165 [Bombyx mori] |
| bmsk0000180 | | 0.146666667 | | 0.626666667 | 2.095157233 | miniparamyosin, partial |
| bmsk0015912 | | 0.93 | | 3.96 | 2.090197809 | XP 004923477.1 ELKS/Rab6-interacting/CAST family member 1 isoform X1 |
| bmsk0002473 | | 0.04 | | 0.17 | 2.087462841 | uncharacterized protein LOC101746734 isoform X1 |
| bmsk0013249 | | 0.58 | | 2.446666667 | 2.076692757 | AEM66422.1 moricin B3 |
| bmsk0011195 | | 0.07 | | 0.293333333 | 2.067114196 | XP 004922398.2 uncharacterized protein LOC101735305 |
| bmsk0000013 | | 0.703333333 | | 2.946666667 | 2.066803371 | SET and MYND domain-containing protein 4 |
| bmsk0008094 | | 0.073333333 | | 0.306666667 | 2.064130337 | glucose dehydrogenase [FAD, quinone]-like [Papilio xuthus] |
| bmsk0012166 | | 0.223333333 | | 0.933333333 | 2.063193826 | uncharacterized protein LOC101741259 |
| bmsk0011439 | | 0.436666667 | | 1.823333333 | 2.061974021 | XP 021204583.1 kelch-like protein 3 |
| bmsk0012715 | | 0.056666667 | | 0.233333333 | 2.041820176 | protein still life, isoform SIF type 1 |
| bmsk0015292 | | 2.26 | | 9.273333333 | 2.036765241 | actin-binding LIM protein 3 isoform X3 [Plutella xylostella] |
| bmsk0007743 | | 0.33 | | 1.346666667 | 2.028854863 | yellow-y isoform X1 |
| bmsk0004842 | | 0.133333333 | | 0.543333333 | 2.026800059 | hypothetical protein KGM 17444 [Danaus plexippus] |
| bmsk0006825 | | 0.136666667 | | 0.556666667 | 2.026152288 | cuticular protein hypothetical 28 precursor |
| bmsk0002302 | | 0.136666667 | | 0.553333333 | 2.017487427 | sex-determining region Y protein-like [Amyelois transitella] |
| bmsk0012472 | | 1.43 | | 5.74 | 2.00503559 | ABL75549.1 eukaryotic initiation factor 4E-1 |
| bmsk0011430 | | 0.026666667 | | 0.106666667 | 2 | putative Trypsin alpha [Danaus plexippus] |
| bmsk0000752 | | 0.016666667 | | 0.066666667 | 2 | androgen-dependent TFPI-regulating protein-like isoform X2 |
| mstrg.4060 | | 15.01 | | 60.03 | 1.999759691 | chitin deacetylase 4 [Cnaphalocrocis medinalis] |
| bmsk0011897 | | 61.72 | | 246.2433333 | 1.996274699 | beta-tubulin |
| bmsk0011440 | | 0.233333333 | | 0.92 | 1.97924144 | XP 021204456.1 ionotropic GABA-aminobutyric acid receptor RDL3 isoform X7 |
| bmsk0004596 | | 0.09 | | 0.353333333 | 1.973032952 | uncharacterized protein LOC101745337 |
| mstrg.8028 | | 0.676666667 | | 2.65 | 1.969475133 | PREDICTED: uncharacterized protein LOC101736812 [Bombyx mori] |
| bmsk0011966 | | 0.156666667 | | 0.61 | 1.961110987 | - |
| bmsk0001322 | | 1.883333333 | | 7.313333333 | 1.957240753 | uncharacterized protein LOC101741404 |
| bmsk0000230 | | 0.54 | | 2.093333333 | 1.954770746 | cyclic nucleotide-gated cation channel subunit A |
| bmsk0001086 | | 0.613333333 | | 2.356666667 | 1.942004449 | Ig-like and fibronectin type-III domain-containing protein 1 isoform X1 |
| bmsk0001286 | | 1.556666667 | | 5.98 | 1.941685435 | protein spaetzle 3 |
| bmsk0010807 | | 0.22 | | 0.843333333 | 1.938599455 | XP 021203283.1 connectin |
| bmsk0001622 | | 0.193333333 | | 0.74 | 1.936434871 | uncharacterized protein LOC105842097 |
| bmsk0013452 | | 0.036666667 | | 0.14 | 1.932885804 | - |
| bmsk0007761 | | 0.953333333 | | 3.633333333 | 1.930241083 | uncharacterized protein LOC106131303 isoform X1 [Amyelois transitella] |
| mstrg.22612 | | 0.543333333 | | 2.06 | 1.922734874 | endonuclease-reverse transcriptase [Bombyx mori] |
| bmsk0009286 | | 0.13 | | 0.49 | 1.914270126 | glucose dehydrogenase [FAD, quinone] |
| bmsk0015783 | | 0.27 | | 1.006666667 | 1.898554736 | XP 021208504.1 vacuolar ATPase subunit C isoform X1 |
| bmsk0007033 | | 0.103333333 | | 0.383333333 | 1.891293741 | - |
| bmsk0011575 | | 0.64 | | 2.366666667 | 1.886712714 | facilitated trehalose transporter Tret1-like [Amyelois transitella] |
| bmsk0009206 | | 0.253333333 | | 0.933333333 | 1.881355504 | limulus clotting factor C isoform X1 |
| bmsk0002030 | | 0.793333333 | | 2.916666667 | 1.878321443 | Bm2-cadherin, partial |
| bmsk0011020 | | 0.09 | | 0.33 | 1.874469118 | XP 012552026.1 receptor-type tyrosine-protein phosphatase kappa isoform X1 |
| bmsk0003985 | | 0.723333333 | | 2.63 | 1.862330258 | Glutathione S-transferase, N-terminal domain |
| bmsk0003568 | | 0.236666667 | | 0.86 | 1.861480136 | - |
| bmsk0009698 | | 0.126666667 | | 0.46 | 1.860596943 | hypothetical protein KGM 18550 [Danaus plexippus] |
| bmsk0002062 | | 0.106666667 | | 0.386666667 | 1.857980995 | MORN repeat |
| mstrg.16214 | | 0.053333333 | | 0.193333333 | 1.857980995 | PREDICTED: pro-resilin [Bombyx mori] |
| bmsk0005069 | | 45.15666667 | | 160.6933333 | 1.831299178 | bifunctional purine biosynthesis protein PURH [Papilio xuthus] |
| bmsk0008398 | | 0.266666667 | | 0.94 | 1.817623258 | protein vav isoform X1 |
| bmsk0015845 | | 2.06 | | 7.226666667 | 1.810686013 | XP 004933560.1 adenine phosphoribosyltransferase isoform X1 |
| bmsk0009941 | | 17.24333333 | | 60.45333333 | 1.8097832 | SOCS2-12 protein |
| bmsk0013781 | | 0.173333333 | | 0.603333333 | 1.799406169 | XP 004923771.2 protein stum |
| bmsk0014719 | | 0.436666667 | | 1.513333333 | 1.793125486 | uncharacterized protein LOC101738037 isoform X1 |
| bmsk0002054 | | 1.613333333 | | 5.523333333 | 1.77549465 | probable nuclear hormone receptor HR38 |
| bmsk0007697 | | 1.073333333 | | 3.663333333 | 1.771058793 | eye-specific diacylglycerol kinase isoform X2 |
| bmsk0010648 | | 0.186666667 | | 0.636666667 | 1.770073906 | UDP-glycosyltransferase UGT43B1 precursor |
| bmsk0012869 | | 1.873333333 | | 6.37 | 1.765685743 | XP 012547082.1 uncharacterized protein LOC101741916 isoform X1 |
| bmsk0010849 | | 0.033333333 | | 0.113333333 | 1.765534746 | XP 012546508.1 nose resistant to fluoxetine protein 6 |
| bmsk0010033 | | 0.326666667 | | 1.106666667 | 1.760329587 | chaoptin |
| bmsk0015391 | | 0.156666667 | | 0.53 | 1.758294104 | BAL70382.1 cecropin D |
| bmsk0003468 | | 0.25 | | 0.833333333 | 1.736965594 | suppressor of lurcher protein 1-like [Amyelois transitella] |
| bmsk0005397 | | 2.323333333 | | 7.72 | 1.732404692 | leucine-rich repeat-containing protein 70 [Papilio machaon] |
| bmsk0012638 | | 0.64 | | 2.123333333 | 1.730187062 | XP 004931952.1 elongation of very long chain fatty acids protein AAEL008004 isoform X2 |
| bmsk0007348 | | 0.366666667 | | 1.213333333 | 1.726434927 | guanine nucleotide binding protein isoform X1 |
| bmsk0015012 | | 0.066666667 | | 0.22 | 1.722466024 | XP 021202293.1 neurexin-1 isoform X4 |
| bmsk0007536 | | 0.383333333 | | 1.263333333 | 1.720563987 | spondin-1-like [Papilio xuthus] |
| bmsk0010133 | | 0.403333333 | | 1.323333333 | 1.71413196 | uncharacterized protein LOC105842483 isoform X1 |
| bmsk0000049 | | 0.126666667 | | 0.413333333 | 1.706268797 | alpha-1,3-mannosyl-glycoprotein 4-beta-N-acetylglucosaminyltransferase B isoform X1 |
| mstrg.10648 | | 0.063333333 | | 0.206666667 | 1.706268797 | cuticular protein RR-1 motif 11 precursor [Bombyx mori] |
| mstrg.22442 | | 34.13333333 | | 110.3033333 | 1.692223175 | Rab-related protein [Bombyx mori] |
| bmsk0010243 | | 0.363333333 | | 1.173333333 | 1.691247294 | Ankyrin repeats (many copies) |
| mstrg.10178 | | 1.386666667 | | 4.47 | 1.688653804 | PREDICTED: homeobox protein Nkx-2.2a isoform X2 [Bombyx mori] |
| bmsk0008951 | | 0.943333333 | | 3.036666667 | 1.686649001 | acetylcholinesterase type 1 |
| bmsk0000640 | | 0.08 | | 0.256666667 | 1.68182404 | uncharacterized protein LOC101746166 |
| bmsk0012663 | | 0.346666667 | | 1.096666667 | 1.661504056 | XP 012550772.2 SH3 and cysteine-rich domain-containing protein 3 |
| bmsk0003884 | | 0.153333333 | | 0.483333333 | 1.656347134 | glutamate [NMDA] receptor subunit 1 |
| bmsk0009602 | | 0.203333333 | | 0.64 | 1.654225163 | - |
| bmsk0014405 | | 4.943333333 | | 15.41 | 1.640310765 | XP 004925157.1 golgin subfamily A member 6-like protein 22 isoform X1 |
| bmsk0013792 | | 0.033333333 | | 0.103333333 | 1.632268215 | hypothetical protein TcasGA2 TC034914, partial [Tribolium castaneum] |
| bmsk0014891 | | 0.173333333 | | 0.536666667 | 1.63047716 | XP 021202670.1 uncharacterized protein LOC101741470 |
| bmsk0001341 | | 0.476666667 | | 1.473333333 | 1.628031223 | cytochrome b5 reductase 4 isoform X1 [Amyelois transitella] |
| bmsk0007777 | | 0.716666667 | | 2.21 | 1.624672211 | uncharacterized protein LOC101741917 isoform X1 |
| bmsk0006927 | | 0.3 | | 0.913333333 | 1.606178987 | lachesin-like [Papilio machaon] |
| bmsk0000262 | | 1.253333333 | | 3.8 | 1.600229257 | - |
| bmsk0001583 | | 0.693333333 | | 2.093333333 | 1.594181031 | protein msta |
| bmsk0012197 | | 2.283333333 | | 6.87 | 1.589168612 | XP 004929673.1 tyrosine-protein kinase CSK isoform X3 |
| bmsk0009247 | | 0.553333333 | | 1.663333333 | 1.587856574 | uncharacterized protein LOC101743839 |
| bmsk0004088 | | 0.3 | | 0.9 | 1.584962501 | Uncharacterized protein OBRU01 13078 [Operophtera brumata] |
| mstrg.9560 | | 1.633333333 | | 4.896666667 | 1.583980742 | PREDICTED: major royal jelly protein 1 [Bombyx mori] |
| bmsk0004620 | | 0.563333333 | | 1.68 | 1.576400487 | - |
| bmsk0010055 | | 15.41666667 | | 45.95 | 1.575573997 | uncharacterized protein LOC101737344 |
| bmsk0003928 | | 1.436666667 | | 4.28 | 1.574885428 | FK506-binding protein 5-like isoform X2 [Papilio polytes] |
| bmsk0013793 | | 25.76333333 | | 76.60666667 | 1.572150682 | AFO09967.1 IBP2 |
| bmsk0002996 | | 0.26 | | 0.763333333 | 1.553801569 | atrial natriuretic peptide-converting enzyme |
| bmsk0007696 | | 0.05 | | 0.146666667 | 1.552541023 | uncharacterized protein LOC105388832 [Plutella xylostella] |
| bmsk0002518 | | 3.056666667 | | 8.913333333 | 1.544005826 | ken and barbie protein isoform X1 |
| bmsk0003442 | | 0.26 | | 0.756666667 | 1.541146268 | solute carrier family 46 member 3-like |
| bmsk0015064 | | 0.036666667 | | 0.106666667 | 1.540568381 | trichohyalin |
| bmsk0000102 | | 0.236666667 | | 0.68 | 1.522678222 | enkurin [Papilio xuthus] |
| bmsk0011313 | | 3.32 | | 9.516666667 | 1.519273098 | - |
| bmsk0002130 | | 4.246666667 | | 12.15 | 1.516553537 | tetraspanin-7 |
| bmsk0001367 | | 1.34 | | 3.83 | 1.515111391 | protein split ends |
| bmsk0002531 | | 0.093333333 | | 0.266666667 | 1.514573173 | uncharacterized protein LOC101740429 |
| bmsk0003195 | | 0.13 | | 0.37 | 1.509013647 | - |
| mstrg.10647 | | 0.553333333 | | 1.573333333 | 1.507603618 | cuticular protein RR-1 motif 11 precursor [Bombyx mori] |
| bmsk0001167 | | 16.09333333 | | 45.70666667 | 1.505941433 | - |
| bmsk0010514 | | 0.15 | | 0.423333333 | 1.49683159 | XP 012547469.2 inhibin beta chain |
| bmsk0005038 | | 7.91 | | 22.17 | 1.48685917 | phosphoserine aminotransferase |
| bmsk0012936 | | 0.323333333 | | 0.903333333 | 1.482236199 | XP 021202683.1 uncharacterized protein LOC105841416 |
| mstrg.6790 | | 0.483333333 | | 1.35 | 1.481869008 | PREDICTED: nose resistant to fluoxetine protein 6-like [Bombyx mori] |
| mstrg.25400 | | 0.983333333 | | 2.726666667 | 1.471385889 | PREDICTED: leucine-rich repeat-containing protein 1-like [Bombyx mori] |
| bmsk0004648 | | 0.116666667 | | 0.323333333 | 1.470629825 | myosin-IB isoform X3 |
| bmsk0005096 | | 1.2 | | 3.29 | 1.455053178 | uncharacterized protein LOC101745836 |
| mstrg.783 | | 1.313333333 | | 3.6 | 1.454763778 | PREDICTED: cationic amino acid transporter 2 [Bombyx mori] |
| bmsk0001395 | | 0.5 | | 1.37 | 1.454175893 | AII79417.1 integrin beta5 |
| bmsk0001891 | | 3.026666667 | | 8.29 | 1.453642305 | protein kinase DC2 |
| bmsk0001285 | | 2.08 | | 5.693333333 | 1.452690041 | Spatzle 3 [Operophtera brumata] |
| bmsk0005519 | | 0.386666667 | | 1.056666667 | 1.450358035 | Palmitoyltransferase [Operophtera brumata] |
| bmsk0008670 | | 1.093333333 | | 2.956666667 | 1.43523829 | - |
| bmsk0004873 | | 0.326666667 | | 0.88 | 1.429684275 | - |
| bmsk0013403 | | 43.45 | | 116.8266667 | 1.426941537 | transferrin precursor |
| bmsk0010989 | | 0.33 | | 0.886666667 | 1.425925815 | sodium/potassium/calcium exchanger Nckx30C-like |
| bmsk0006932 | | 0.25 | | 0.666666667 | 1.415037499 | igLON family member 5-like |
| bmsk0010486 | | 3.313333333 | | 8.833333333 | 1.414674603 | XP 004928146.2 alkaline phosphatase |
| bmsk0005161 | | 0.543333333 | | 1.446666667 | 1.412823078 | tropomodulin-1 isoform X2 |
| bmsk0004194 | | 1.883333333 | | 5.013333333 | 1.412481794 | uncharacterized protein LOC101735738 isoform X2 |
| bmsk0003274 | | 0.223333333 | | 0.59 | 1.40151636 | XP 012549645.1 tyrosine-protein kinase Fer isoform X4 |
| bmsk0007856 | | 0.653333333 | | 1.72 | 1.396517411 | adipose triglyceride lipase brummer |
| bmsk0013468 | | 0.526666667 | | 1.383333333 | 1.393186778 | uncharacterized protein LOC101746443 |
| bmsk0009342 | | 0.08 | | 0.21 | 1.392317423 | ABC transporter F family member 4 isoform X1 |
| bmsk0005576 | | 15.28333333 | | 40.06666667 | 1.390443257 | beta-galactosidase |
| bmsk0011930 | | 0.2 | | 0.523333333 | 1.387730153 | uncharacterized protein LOC106140489 [Amyelois transitella] |
| bmsk0003501 | | 0.39 | | 1.016666667 | 1.382300713 | C3 and PZP-like alpha-2-macroglobulin domain-containing protein 8 |
| bmsk0010013 | | 0.46 | | 1.196666667 | 1.379315577 | Voltage-dependent calcium channel gamma-5 subunit [Papilio machaon] |
| bmsk0013632 | | 0.14 | | 0.363333333 | 1.375866902 | XP 021206642.1 histone-lysine N-methyltransferase SETD1B isoform X2 |
| bmsk0006769 | | 0.486666667 | | 1.26 | 1.372417865 | putative s-syntaxin [Danaus plexippus] |
| mstrg.13227 | | 58.88 | | 151.97 | 1.367936977 | PREDICTED: collagen and calcium-binding EGF domain-containing protein 1-like [Bombyx mori] |
| mstrg.22552 | | 94.15333333 | | 242.1233333 | 1.362658041 | uncharacterized LOC106111899 [Papilio polytes] |
| bmsk0012995 | | 0.28 | | 0.72 | 1.362570079 | radial spoke head protein 3 homolog B [Amyelois transitella] |
| bmsk0013251 | | 0.44 | | 1.13 | 1.360747344 | - |
| bmsk0001179 | | 0.886666667 | | 2.266666667 | 1.354108501 | espin |
| bmsk0001168 | | 20.53 | | 52.14 | 1.344656955 | Major Facilitator Superfamily |
| bmsk0002838 | | 0.626666667 | | 1.59 | 1.343256604 | proline-rich receptor-like protein kinase PERK12 |
| mstrg.10267 | | 5.046666667 | | 12.76333333 | 1.338602455 | PREDICTED: uncharacterized protein K02A2.6-like [Papilio polytes] |
| mstrg.20123 | | 3.123333333 | | 7.896666667 | 1.338157246 | PREDICTED: uncharacterized protein K02A2.6-like [Papilio polytes] |
| bmsk0010520 | | 0.12 | | 0.303333333 | 1.337869639 | disheveled-associated activator of morphogenesis 1 isoform X1 |
| bmsk0007479 | | 1.013333333 | | 2.553333333 | 1.333273068 | venom dipeptidyl peptidase 4 |
| bmsk0013568 | | 0.066666667 | | 0.166666667 | 1.321928095 | multidrug resistance protein homolog 49-like |
| bmsk0005226 | | 0.093333333 | | 0.233333333 | 1.321928095 | cytoplasmic dynein 1 intermediate chain isoform X9 |
| bmsk0005030 | | 110.9033333 | | 277.2366667 | 1.321815349 | - |
| bmsk0015557 | | 0.196666667 | | 0.49 | 1.317029295 | endothelin-converting enzyme 1 |
| bmsk0012162 | | 0.43 | | 1.07 | 1.315202232 | XP 021205922.1 small conductance calcium-activated potassium channel protein isoform X1 |
| bmsk0004154 | | 0.233333333 | | 0.58 | 1.313660479 | metabotropic glutamate receptor 2-like |
| bmsk0007707 | | 8.233333333 | | 20.36666667 | 1.306661338 | transient receptor potential cation channel protein painless |
| bmsk0011939 | | 0.12 | | 0.296666667 | 1.30580843 | - |
| bmsk0014231 | | 3.716666667 | | 9.18 | 1.304484849 | serine/threonine-protein kinase MARK2-like isoform X9 |
| bmsk0014982 | | 2.476666667 | | 6.103333333 | 1.301197675 | XP 004924096.1 b(0,+)-type amino acid transporter 1 isoform X1 |
| bmsk0006076 | | 0.123333333 | | 0.303333333 | 1.298341275 | zinc metalloproteinase nas-14 |
| bmsk0001572 | | 0.293333333 | | 0.72 | 1.295455884 | potassium voltage-gated channel protein Shaw |
| bmsk0015518 | | 7.63 | | 18.72666667 | 1.295339162 | R recognition motif. (a.k.a. RRM, RBD, or RNP domain) |
| bmsk0013397 | | 0.773333333 | | 1.896666667 | 1.294303847 | XP 021207886.1 ionotropic receptor 25a isoform X2 |
| bmsk0010501 | | 0.54 | | 1.323333333 | 1.293145194 | XP 012547525.1 uncharacterized protein LOC105841963 isoform X1 |
| bmsk0006344 | | 7.21 | | 17.66666667 | 1.292958694 | supervillin-like isoform X2 |
| bmsk0002398 | | 2.18 | | 5.34 | 1.292511607 | - |
| bmsk0001809 | | 0.26 | | 0.636666667 | 1.292026609 | toll-like receptor 8 |
| bmsk0009752 | | 0.356666667 | | 0.873333333 | 1.291956015 | chaoptin |
| bmsk0010740 | | 0.406666667 | | 0.99 | 1.283581783 | PAS domain |
| bmsk0007609 | | 0.83 | | 2.02 | 1.283172051 | paralytic peptide precursor |
| bmsk0003034 | | 0.856666667 | | 2.08 | 1.27977767 | oxysterol-binding protein-related protein 2 |
| bmsk0014736 | | 9.433333333 | | 22.86 | 1.276985851 | cystathionine beta-synthase-like |
| bmsk0003778 | | 1.083333333 | | 2.616666667 | 1.272252936 | tubulin polyglutamylase TTLL4-like isoform X2 |
| bmsk0000117 | | 1.68 | | 4.056666667 | 1.271833529 | succinate dehydrogenase assembly factor 3, mitochondrial |
| bmsk0006953 | | 0.516666667 | | 1.246666667 | 1.270770055 | hypothetical protein RR46 14092 [Papilio xuthus] |
| bmsk0010382 | | 0.283333333 | | 0.683333333 | 1.270089163 | uncharacterized protein LOC101746142 |
| bmsk0012528 | | 2.166666667 | | 5.183333333 | 1.258402957 | XP 004931988.1 IST1 homolog |
| bmsk0000777 | | 0.456666667 | | 1.09 | 1.255114743 | pyruvate dehydrogenase kinase |
| bmsk0004970 | | 434.03 | | 1035.94 | 1.255073777 | S-adenosylmethionine synthetase |
| bmsk0014216 | | 7.973333333 | | 19.01666667 | 1.254009497 | - |
| bmsk0012631 | | 0.193333333 | | 0.46 | 1.250543462 | cuticular protein RR-2 motif 79 precursor |
| bmsk0005659 | | 3.41 | | 8.066666667 | 1.242200902 | hypothetical protein KGM 05194 [Danaus plexippus] |
| bmsk0003694 | | 0.393333333 | | 0.93 | 1.241478262 | uncharacterized protein LOC101741477 isoform X2 |
| bmsk0002138 | | 0.14 | | 0.33 | 1.237039197 | hydrocephalus-inducing protein homolog |
| bmsk0014391 | | 2.09 | | 4.916666667 | 1.234177606 | XP 012545000.1 uncharacterized protein LOC101739017 |
| bmsk0008035 | | 0.193333333 | | 0.453333333 | 1.229481846 | roundabout homolog 3 |
| bmsk0011318 | | 0.506666667 | | 1.186666667 | 1.227805918 | XP 004924230.1 chaoptin |
| bmsk0009085 | | 5.576666667 | | 13.04666667 | 1.226206311 | pro-phenol oxidase [Bombyx mandarina] |
| bmsk0003812 | | 29.10666667 | | 68.03 | 1.22482146 | serine protease inhibitor 3 isoform X1 |
| mstrg.24509 | | 13.59666667 | | 31.72 | 1.222139765 | olfactory receptor [Bombyx mori] |
| bmsk0006628 | | 1.233333333 | | 2.87 | 1.218487967 | neuropeptide receptor A11 |
| bmsk0011979 | | 4.396666667 | | 10.18666667 | 1.212199979 | probable GPI-anchored adhesin-like protein PGA55 |
| bmsk0010276 | | 0.623333333 | | 1.443333333 | 1.211328755 | Regulator of G protein signaling domain |
| bmsk0006430 | | 0.37 | | 0.856666667 | 1.211208683 | potassium voltage-gated channel protein Shab isoform X1 |
| bmsk0002324 | | 0.403333333 | | 0.926666667 | 1.200077835 | voltage-dependent L-type calcium channel subunit beta-2 [Amyelois transitella] |
| bmsk0012298 | | 569.58 | | 1307.713333 | 1.199075925 | ribosomal protein L36 |
| bmsk0003917 | | 0.22 | | 0.503333333 | 1.19401062 | putative transcription factor capicua |
| bmsk0001728 | | 4.786666667 | | 10.94333333 | 1.192959001 | - |
| bmsk0005316 | | 1.33 | | 3.03 | 1.187891548 | - |
| bmsk0000088 | | 0.373333333 | | 0.85 | 1.186998515 | glucose transporter type 1-like |
| bmsk0014382 | | 2.196666667 | | 5 | 1.18661213 | programmed cell death protein 4-like |
| bmsk0002517 | | 584.24 | | 1323.663333 | 1.179903186 | 32 kDa apolipoprotein precursor |
| bmsk0002943 | | 6.94 | | 15.71333333 | 1.17898169 | uncharacterized protein LOC106129803 [Amyelois transitella] |
| bmsk0010168 | | 43.68333333 | | 98.9 | 1.178887573 | serine hydroxymethyltransferase isoform X1 |
| bmsk0002735 | | 9.616666667 | | 21.62 | 1.168757705 | uncharacterized protein LOC106709059 [Papilio machaon] |
| bmsk0004460 | | 0.416666667 | | 0.936666667 | 1.168642036 | hypothetical protein IscW ISCW019942 [Ixodes scapularis] |
| bmsk0007755 | | 3.016666667 | | 6.77 | 1.166200542 | uncharacterized protein LOC101742398 |
| bmsk0003960 | | 7.64 | | 17.14 | 1.165722566 | uncharacterized protein LOC101738995 |
| bmsk0007742 | | 3.213333333 | | 7.173333333 | 1.158573026 | proton-coupled amino acid transporter 1-like isoform X2 |
| mstrg.4923 | | 1.906666667 | | 4.25 | 1.156410195 | PREDICTED: lipopolysaccharide-induced tumor necrosis factor-alpha factor homolog [Bombyx mori] |
| bmsk0006958 | | 0.41 | | 0.913333333 | 1.155517578 | ASK1 |
| bmsk0008203 | | 0.23 | | 0.51 | 1.148863386 | hypothetical protein KGM 15715 [Danaus plexippus] |
| bmsk0011745 | | 9.436666667 | | 20.90666667 | 1.14761381 | - |
| bmsk0012550 | | 0.823333333 | | 1.823333333 | 1.147029791 | uncharacterized protein LOC101744721 isoform X2 |
| bmsk0003493 | | 10.58666667 | | 23.38 | 1.143026518 | - |
| mstrg.1208 | | 22.64333333 | | 49.96333333 | 1.141783377 | PREDICTED: C-type lectin 11 isoform X1 [Bombyx mori] |
| bmsk0005000 | | 2.383333333 | | 5.253333333 | 1.140252388 | C-Cbl-associated protein isoform A [Operophtera brumata] |
| bmsk0003025 | | 2.1 | | 4.613333333 | 1.135420209 | uncharacterized protein LOC101741240 isoform X2 |
| bmsk0002879 | | 2.756666667 | | 6.04 | 1.131623721 | protein rolling stone isoform X2 |
| bmsk0003353 | | 0.23 | | 0.503333333 | 1.129880283 | uncharacterized protein LOC101741926 |
| bmsk0012962 | | 5.486666667 | | 12.00333333 | 1.129433264 | uncharacterized protein LOC101736037 isoform X1 |
| bmsk0003464 | | 2.296666667 | | 5.02 | 1.128145882 | zonadhesin-like isoform X1 |
| bmsk0000650 | | 0.503333333 | | 1.096666667 | 1.123539034 | Domain of unknown function (DUF4780) |
| bmsk0005312 | | 0.28 | | 0.61 | 1.123382416 | solute carrier family 25 member 35 |
| mstrg.3604 | | 0.976666667 | | 2.12 | 1.118126101 | serpin-24, partial [Bombyx mori] |
| bmsk0001159 | | 18.48666667 | | 40.08333333 | 1.116517373 | S-adenosylmethionine decarboxylase proenzyme isoform X1 |
| bmsk0014029 | | 3.263333333 | | 7.066666667 | 1.1146835 | XP 004926734.1 zinc finger protein 26 |
| bmsk0004662 | | 4.703333333 | | 10.15333333 | 1.110197954 | Glycosyl hydrolases family 18 |
| bmsk0014644 | | 0.063333333 | | 0.136666667 | 1.109624491 | - |
| bmsk0006685 | | 14.07333333 | | 30.32 | 1.107305676 | uncharacterized protein LOC101738865 isoform X2 |
| bmsk0011143 | | 7.866666667 | | 16.92333333 | 1.1051894 | uncharacterized protein LOC101745941 |
| bmsk0006083 | | 51.63666667 | | 111.06 | 1.104871523 | protein LLP homolog |
| bmsk0005431 | | 6.603333333 | | 14.17333333 | 1.101912717 | receptor-type tyrosine-protein phosphatase N2 isoform X2 |
| bmsk0013363 | | 20.82666667 | | 44.61 | 1.098935195 | XP 004932688.1 monocarboxylate transporter 7 |
| bmsk0015080 | | 6.603333333 | | 14.14333333 | 1.098855798 | ABC transporter G family member 23 isoform X2 [Amyelois transitella] |
| mstrg.9389 | | 5.333333333 | | 11.41666667 | 1.098032083 | PREDICTED: protein snakeskin-like [Anoplophora glabripennis] |
| bmsk0007080 | | 0.276666667 | | 0.59 | 1.092566119 | coiled-coil domain-containing protein 170 |
| bmsk0004640 | | 10.71666667 | | 22.83666667 | 1.091495848 | CBP80/20-dependent translation initiation factor isoform X1 |
| bmsk0000153 | | 110.7966667 | | 236.0533333 | 1.091198377 | ATP-binding cassette sub-family F member 2 isoform X1 |
| bmsk0014370 | | 0.18 | | 0.383333333 | 1.090602549 | - |
| bmsk0004739 | | 8.23 | | 17.51333333 | 1.089489364 | peptidoglycan recognition protein precursor |
| bmsk0011972 | | 98.66 | | 209.57 | 1.086895017 | ABF51391.1 ribosomal protein L24 |
| bmsk0001933 | | 67.13333333 | | 142.3633333 | 1.084476435 | uncharacterized protein LOC106143014 [Amyelois transitella] |
| bmsk0004424 | | 0.146666667 | | 0.31 | 1.079727192 | cytochrome P450, partial |
| bmsk0015775 | | 0.826666667 | | 1.746666667 | 1.079226691 | - |
| bmsk0015991 | | 4.263333333 | | 8.996666667 | 1.077408713 | serpin 5 [Bombyx mandarina] |
| bmsk0015992 | | 4.263333333 | | 8.996666667 | 1.077408713 | - |
| bmsk0008870 | | 2.08 | | 4.383333333 | 1.075444865 | - |
| bmsk0003199 | | 18.85666667 | | 39.64666667 | 1.072124905 | diacylglycerol kinase theta isoform X6 |
| bmsk0006497 | | 0.64 | | 1.343333333 | 1.069673528 | BAI1-associated protein 3 |
| bmsk0013541 | | 22.43666667 | | 47.00333333 | 1.066904716 | choline/ethanolamine kinase isoform X1 [Papilio polytes] |
| bmsk0004479 | | 94.48666667 | | 197.77 | 1.065640933 | uncharacterized protein LOC101741832 |
| bmsk0011100 | | 0.233333333 | | 0.486666667 | 1.060541542 | Plasmodium histidine-rich protein (HRPII/III) |
| bmsk0013772 | | 0.24 | | 0.5 | 1.058893689 | XP 021201996.1 uncharacterized protein LOC110384788 |
| bmsk0007518 | | 1.696666667 | | 3.533333333 | 1.058326703 | low-density lipoprotein receptor-related protein 4-like |
| bmsk0014806 | | 0.246666667 | | 0.513333333 | 1.057333175 | uncharacterized protein LOC105842335 |
| bmsk0004685 | | 2.773333333 | | 5.77 | 1.056950291 | zinc finger CCHC domain-containing protein 4 |
| bmsk0009855 | | 1.786666667 | | 3.706666667 | 1.052851882 | lethal(2) giant larvae protein isoform X1 |
| bmsk0009170 | | 0.223333333 | | 0.463333333 | 1.052851882 | uncharacterized protein LOC101743410 |
| bmsk0015768 | | 7.173333333 | | 14.84 | 1.048775515 | ACG61177.1 serpin-14 |
| bmsk0010436 | | 0.373333333 | | 0.77 | 1.044394119 | dopamine D2-like receptor [Amyelois transitella] |
| mstrg.4922 | | 1.556666667 | | 3.19 | 1.035096375 | olfactory receptor [Bombyx mori] |
| bmsk0011832 | | 0.683333333 | | 1.396666667 | 1.031326334 | - |
| bmsk0004608 | | 12.13333333 | | 24.72333333 | 1.026897319 | perilipin-4 isoform X4 |
| bmsk0006613 | | 3.346666667 | | 6.813333333 | 1.025635927 | ATP-binding cassette sub-family G member 1 |
| bmsk0002685 | | 0.13 | | 0.263333333 | 1.018378529 | Facilitated trehalose transporter Tret1; Short=BmTRET1 |
| bmsk0006354 | | 0.266666667 | | 0.54 | 1.017921908 | protein FAM135A |
| bmsk0013058 | | 0.433333333 | | 0.876666667 | 1.016551176 | uncharacterized protein LOC105841534 |
| bmsk0008809 | | 11.65333333 | | 23.55666667 | 1.015392724 | - |
| bmsk0015355 | | 5.073333333 | | 10.25333333 | 1.015087144 | XP 012552182.1 casein kinase I alpha isoform X5 |
| bmsk0007018 | | 0.323333333 | | 0.653333333 | 1.014797002 | disks large 1 tumor suppressor protein |
| bmsk0011158 | | 0.323333333 | | 0.65 | 1.007417472 | chemosensory protein 10 isoform X1 |
| bmsk0013774 | | 2.986666667 | | 6.003333333 | 1.007227544 | - |
| bmsk0006736 | | 0.103333333 | | 0.206666667 | 1 | histone-lysine N-methyltransferase E(z) |
| bmsk0003017 | | 26.98333333 | | 13.42 | -1.007683908 | cullin-4A [Papilio xuthus] |
| bmsk0005686 | | 15.74666667 | | 7.806666667 | -1.012267889 | ATP-binding cassette sub-family F member 3 |
| bmsk0009931 | | 15.67 | | 7.766666667 | -1.012637726 | protein Gawky [Papilio xuthus] |
| bmsk0012463 | | 16.74333333 | | 8.236666667 | -1.023454264 | ubiquitin-like protein 4A [Papilio machaon] |
| mstrg.2622 | | 13.10333333 | | 6.426666667 | -1.027791312 | GTP-binding protein 2 [Papilio xuthus] |
| bmsk0006078 | | 10.07 | | 4.936666667 | -1.028454543 | Major Facilitator Superfamily |
| bmsk0004262 | | 104.2 | | 51.02666667 | -1.030031972 | 26S proteasome non-ATPase regulatory subunit 11 |
| bmsk0014201 | | 85.07333333 | | 41.66 | -1.030044143 | XP 004923158.1 rR 2'-O-methyltransferase fibrillarin |
| bmsk0012711 | | 19.66333333 | | 9.58 | -1.037410347 | XP 004931900.1 broad-complex core protein isoforms 1/2/3/4/5 |
| bmsk0011827 | | 47.79666667 | | 23.06666667 | -1.051100471 | ADZ96259.1 robl, partial |
| bmsk0008488 | | 65.47 | | 31.56333333 | -1.052584406 | thioredoxin |
| bmsk0007662 | | 16.80333333 | | 8.1 | -1.052753641 | exosome complex component RRP43-like [Amyelois transitella] |
| bmsk0003311 | | 22.22 | | 10.71 | -1.052900337 | leucine-rich repeat protein soc-2 homolog [Papilio xuthus] |
| bmsk0014374 | | 33.52333333 | | 16.15333333 | -1.053333704 | tetratricopeptide repeat protein 1-like [Amyelois transitella] |
| bmsk0010064 | | 8.953333333 | | 4.3 | -1.058088239 | Ubiquitin carboxyl-terminal hydrolase |
| mstrg.14478 | | 24.84666667 | | 11.93 | -1.058458275 | PREDICTED: myoneurin-like isoform X2 [Bombyx mori] |
| bmsk0002427 | | 44.15666667 | | 21.17333333 | -1.060382857 | nucleoporin Nup43 [Amyelois transitella] |
| bmsk0010531 | | 28.93 | | 13.86666667 | -1.060945296 | XP 012547657.1 uncharacterized protein LOC101737777 isoform X1 |
| bmsk0005504 | | 25.65333333 | | 12.26666667 | -1.064403033 | probable R methyltransferase bin3 [Amyelois transitella] |
| bmsk0007175 | | 11.71 | | 5.576666667 | -1.070266131 | multidrug resistance-associated protein 1 isoform X2 |
| bmsk0005559 | | 38.86666667 | | 18.38666667 | -1.079873427 | Golgi reassembly-stacking protein 2 |
| bmsk0012953 | | 28.7 | | 13.57 | -1.080630016 | Uncharacterized protein OBRU01 07690 [Operophtera brumata] |
| bmsk0000952 | | 9.8 | | 4.63 | -1.081769556 | AET05797.1 lasp |
| bmsk0013320 | | 9.596666667 | | 4.533333333 | -1.081961137 | XP 012551396.1 beta-secretase 1 |
| bmsk0015847 | | 27.41 | | 12.92333333 | -1.084724094 | probable ribonuclease ZC3H12B isoform X1 [Plutella xylostella] |
| bmsk0013784 | | 12.58 | | 5.92 | -1.087462841 | thyroid receptor-interacting protein 11-like |
| bmsk0012790 | | 50.15666667 | | 23.43666667 | -1.097674078 | methionine aminopeptidase 2 [Papilio polytes] |
| bmsk0012124 | | 7.986666667 | | 3.726666667 | -1.09970772 | uncharacterized protein LOC101735428 isoform X2 |
| bmsk0010816 | | 105.27 | | 49.10666667 | -1.100103552 | XP 004925604.1 26S proteasome non-ATPase regulatory subunit 8 |
| mstrg.12197 | | 26.54 | | 12.37 | -1.10132287 | PREDICTED: CLIP-associating protein [Amyelois transitella] |
| bmsk0002474 | | 32.88 | | 15.32 | -1.101794002 | solute carrier organic anion transporter family member 2A1-like isoform X1 |
| bmsk0012100 | | 10.65333333 | | 4.96 | -1.102892882 | XP 004929627.1 uncharacterized protein LOC101745487 |
| bmsk0000684 | | 3.646666667 | | 1.696666667 | -1.103875177 | protein furry isoform X3 |
| bmsk0012183 | | 8.283333333 | | 3.853333333 | -1.104104454 | XP 021205951.1 arrestin domain-containing protein 17 isoform X2 |
| bmsk0011171 | | 30.43333333 | | 14.15 | -1.104850306 | XP 004922386.1 28S ribosomal protein S9, mitochondrial |
| bmsk0009984 | | 2.136666667 | | 0.993333333 | -1.105012026 | serine/threonine-protein kinase/endoribonuclease IRE1-like, partial |
| bmsk0014665 | | 16.71666667 | | 7.753333333 | -1.108398604 | XP 012553133.1 DDB1- and CUL4-associated factor 5 isoform X1 |
| bmsk0015800 | | 30.57333333 | | 14.18 | -1.108416322 | XP 004933539.1 mediator of R polymerase II transcription subunit 20 |
| bmsk0004828 | | 30.79 | | 14.26666667 | -1.109813571 | zinc finger protein Elbow [Papilio polytes] |
| bmsk0003365 | | 150.78 | | 69.79666667 | -1.111215034 | p23-like protein |
| bmsk0005299 | | 8.916666667 | | 4.126666667 | -1.111527577 | tumor protein p63-regulated gene 1-like protein isoform X1 |
| bmsk0013492 | | 16.34 | | 7.546666667 | -1.114496526 | coproporphirynogen oxidase |
| bmsk0003263 | | 27.51 | | 12.69333333 | -1.115885162 | phenylalanyl-tR synthetase beta subunit |
| mstrg.26541 | | 13.10666667 | | 6.046666667 | -1.116088866 | molybdenum cofactor sulfurase [Bombyx mori] |
| bmsk0005382 | | 19.80333333 | | 9.116666667 | -1.119164956 | peroxisomal targeting signal 1 receptor [Amyelois transitella] |
| bmsk0001226 | | 9.47 | | 4.356666667 | -1.12013969 | serine/arginine repetitive matrix protein 1-like [Papilio xuthus] |
| bmsk0007588 | | 23.30666667 | | 10.71666667 | -1.120886447 | UDP-N-acetylglucosamine transporter isoform X2 |
| mstrg.24605 | | 19.51333333 | | 8.933333333 | -1.127189745 | PREDICTED: coiled-coil domain-containing protein 174 [Bombyx mori] |
| bmsk0012867 | | 29.86 | | 13.67 | -1.127200923 | ran-binding protein 3 |
| bmsk0012466 | | 11.25 | | 5.146666667 | -1.12821475 | BWK-1-like protein |
| bmsk0001497 | | 7.826666667 | | 3.576666667 | -1.129782332 | uncharacterized protein LOC101743924 isoform X2 |
| bmsk0001816 | | 22.50333333 | | 10.28333333 | -1.12983073 | hypothetical protein KGM 03125 [Danaus plexippus] |
| bmsk0003280 | | 42.37333333 | | 19.34 | -1.131568829 | eukaryotic initiation factor 4B protein |
| bmsk0002242 | | 86.8 | | 39.59333333 | -1.132437511 | signal peptidase complex subunit 2 |
| bmsk0006435 | | 31.45666667 | | 14.34 | -1.133320778 | ubiquitin carboxyl-terminal hydrolase 36 |
| bmsk0011389 | | 18.58333333 | | 8.456666667 | -1.135848286 | XP 004927392.1 trafficking protein particle complex subunit 11 |
| bmsk0013074 | | 18.63666667 | | 8.48 | -1.136007674 | protein FRG1 homolog [Amyelois transitella] |
| bmsk0008607 | | 13.77666667 | | 6.263333333 | -1.137224297 | serine/threonine-protein kinase GA29083 |
| bmsk0010037 | | 18.29 | | 8.303333333 | -1.139292554 | putative Wdr45l protein [Danaus plexippus] |
| bmsk0002508 | | 14.04 | | 6.37 | -1.140177658 | conserved oligomeric Golgi complex subunit 3 |
| bmsk0005273 | | 22.78 | | 10.32666667 | -1.141393104 | small androgen receptor-interacting protein |
| bmsk0010505 | | 33.67 | | 15.25333333 | -1.14233917 | nuclear nucleic acid-binding protein C1D-like [Amyelois transitella] |
| bmsk0010030 | | 52.69666667 | | 23.83 | -1.144932755 | proteasome 26S non-ATPase subunit 12 isoform X1 |
| bmsk0002167 | | 7.706666667 | | 3.473333333 | -1.14978612 | WD repeat-containing protein 59 isoform X3 |
| mstrg.22070 | | 17.35333333 | | 7.803333333 | -1.153050377 | PREDICTED: serine/Arginine-related protein 53 isoform X2 [Bombyx mori] |
| bmsk0009639 | | 26.63 | | 11.97333333 | -1.153227579 | peptidyl-tR hydrolase 2, mitochondrial-like [Papilio xuthus] |
| bmsk0011269 | | 49.65666667 | | 22.31666667 | -1.153865865 | bis(5'-nucleosyl)-tetraphosphatase [asymmetrical] [Plutella xylostella] |
| bmsk0010768 | | 23.36333333 | | 10.49666667 | -1.154314867 | XP 004928363.1 ribosome-recycling factor, mitochondrial |
| bmsk0002452 | | 44.59 | | 20.02333333 | -1.155038037 | cAMP-regulated phosphoprotein 19 [Amyelois transitella] |
| bmsk0015173 | | 10.55333333 | | 4.733333333 | -1.156770326 | serine/threonine-protein phosphatase 2A 55 kDa regulatory subunit B alpha isoform isoform X1 [Amyelois transitella] |
| bmsk0015652 | | 17.60333333 | | 7.89 | -1.157751435 | scavenger receptor type C precursor |
| bmsk0006666 | | 39.45 | | 17.67333333 | -1.158451131 | mitochondrial carrier homolog 2-like [Amyelois transitella] |
| bmsk0001252 | | 53.42333333 | | 23.91666667 | -1.159453664 | transcription factor A, mitochondrial |
| bmsk0014070 | | 17.30666667 | | 7.74 | -1.160922411 | XP 004933634.2 dipeptidyl peptidase 9 |
| bmsk0011121 | | 18.62666667 | | 8.313333333 | -1.163870556 | embryonic development factor |
| bmsk0000210 | | 36.43 | | 16.25666667 | -1.164095523 | Ubiquitin conjugating enzyme 4 [Operophtera brumata] |
| bmsk0003155 | | 6.343333333 | | 2.83 | -1.164439103 | Glucosidase II beta subunit-like |
| bmsk0011286 | | 16.92666667 | | 7.543333333 | -1.16602381 | XP 004922454.1 WD repeat-containing protein 91 isoform X1 |
| bmsk0004855 | | 18.36666667 | | 8.183333333 | -1.166329294 | D-dependent protein deacylase |
| bmsk0014968 | | 43.93666667 | | 19.56333333 | -1.167273214 | transmembrane trafficking protein precursor |
| mstrg.24646 | | 22.39666667 | | 9.943333333 | -1.171482553 | PREDICTED: F-box/WD repeat-containing protein 4-like isoform X1 [Bombyx mori] |
| bmsk0002703 | | 76.36 | | 33.89666667 | -1.171673694 | FUN14 domain-containing protein 1 isoform X1 |
| bmsk0005369 | | 9.443333333 | | 4.19 | -1.172345952 | death related ced-3/Nedd2-like protein |
| bmsk0010483 | | 6.426666667 | | 2.85 | -1.173108726 | methyltransferase-like protein 25 isoform X2 |
| bmsk0010484 | | 6.426666667 | | 2.85 | -1.173108726 | Essential protein Yae1, N terminal |
| bmsk0001734 | | 61.65333333 | | 27.33 | -1.173693437 | la protein homolog [Papilio polytes] |
| bmsk0015236 | | 55.52 | | 24.58 | -1.175522652 | XP 004921870.1 cell growth-regulating nucleolar protein |
| bmsk0009355 | | 9.063333333 | | 4.003333333 | -1.17884 | exocyst complex component 8 [Papilio machaon] |
| bmsk0011499 | | 8.16 | | 3.603333333 | -1.179237035 | 5-oxoprolinase [Amyelois transitella] |
| bmsk0013433 | | 13.24333333 | | 5.846666667 | -1.179580046 | XP 012549734.1 protein FMC1 homolog |
| bmsk0001956 | | 28.15666667 | | 12.41666667 | -1.181198625 | putative leucine-rich repeat-containing protein DDB G0290503 |
| bmsk0009429 | | 11.00333333 | | 4.84 | -1.184861685 | ubiquitin fusion degradation protein 1 homolog isoform X1 [Amyelois transitella] |
| bmsk0003047 | | 15.03 | | 6.606666667 | -1.185850547 | mediator of R polymerase II transcription subunit 27 |
| bmsk0006416 | | 25.71666667 | | 11.29666667 | -1.18680652 | zinc finger CCCH domain-containing protein 18 isoform X3 |
| bmsk0002055 | | 23.64 | | 10.38 | -1.187423592 | vacuolar protein sorting-associated protein 52 homolog isoform X1 [Amyelois transitella] |
| bmsk0000283 | | 16.37666667 | | 7.173333333 | -1.190926161 | uncharacterized protein LOC692928 |
| bmsk0013435 | | 6.56 | | 2.873333333 | -1.190970446 | Tricarboxylate carrier |
| bmsk0003857 | | 11.87333333 | | 5.196666667 | -1.192066588 | cysteine-rich hydrophobic domain-containing protein 2 [Papilio xuthus] |
| mstrg.8269 | | 25.44 | | 11.13 | -1.192645078 | cytochrome P450 CYP367B2 [Helicoverpa armigera] |
| mstrg.22680 | | 36.91333333 | | 16.14333333 | -1.193203519 | PREDICTED: snurportin-1 [Bombyx mori] |
| bmsk0014667 | | 6.083333333 | | 2.66 | -1.193435812 | XP 004923335.1 RAB6A-GEF complex partner protein 2 |
| bmsk0012471 | | 48.99666667 | | 21.38666667 | -1.195971962 | ABF51281.1 mR cap-binding protein eIF4E |
| bmsk0014267 | | 24.28 | | 10.58333333 | -1.19797433 | UPF0488 protein CG14286 [Amyelois transitella] |
| bmsk0010724 | | 33.4 | | 14.55666667 | -1.198168072 | hypoxia up-regulated protein 1 [Papilio polytes] |
| bmsk0001459 | | 20.18 | | 8.79 | -1.198991104 | ubiquitin conjugation factor E4 B isoform X2 [Amyelois transitella] |
| bmsk0001737 | | 2.27 | | 0.986666667 | -1.202057622 | R pseudouridylate synthase domain-containing protein 2 isoform X4 |
| bmsk0003203 | | 8.61 | | 3.733333333 | -1.205548911 | uncharacterized protein LOC101742879 |
| bmsk0004445 | | 19.55666667 | | 8.456666667 | -1.209499473 | cell division protein isoform X1 |
| bmsk0014709 | | 17.25333333 | | 7.456666667 | -1.210272361 | - |
| bmsk0015922 | | 18.99666667 | | 8.21 | -1.210292165 | XP 004923451.1 uncharacterized protein LOC101746975 |
| bmsk0000763 | | 10.12666667 | | 4.37 | -1.212454184 | 3-hydroxy-3-methylglutaryl-CoA reductase precursor |
| bmsk0013650 | | 23.75 | | 10.23 | -1.215121368 | XP 004930783.1 sorting nexin-6 isoform X3 |
| bmsk0005040 | | 3.536666667 | | 1.523333333 | -1.215158586 | cytochrome b5-related protein-like |
| bmsk0004033 | | 16.47666667 | | 7.093333333 | -1.215888756 | PX domain |
| bmsk0001121 | | 11.05666667 | | 4.756666667 | -1.216893677 | PEST proteolytic signal-containing nuclear protein |
| bmsk0007020 | | 9.233333333 | | 3.97 | -1.217712563 | protein sly1 homolog [Papilio polytes] |
| bmsk0013056 | | 10.19 | | 4.376666667 | -1.219249636 | formin-binding protein 4-like |
| bmsk0005180 | | 17.80333333 | | 7.633333333 | -1.221762286 | cysteine-type peptidase isoform X1 |
| bmsk0004525 | | 19.39666667 | | 8.31 | -1.222888363 | transmembrane 9 superfamily member 3 [Papilio xuthus] |
| bmsk0008363 | | 21.46666667 | | 9.17 | -1.227104549 | U3 small nucleolar ribonucleoprotein protein IMP3 isoform X1 |
| bmsk0009585 | | 15.39333333 | | 6.573333333 | -1.227608621 | SH2B adapter protein 1 |
| bmsk0006566 | | 6.816666667 | | 2.91 | -1.228047284 | uncharacterized protein LOC101744765 isoform X1 |
| bmsk0005338 | | 22.89666667 | | 9.77 | -1.228707116 | uncharacterized protein LOC101743276 |
| bmsk0003711 | | 17.07666667 | | 7.283333333 | -1.229355612 | ANTH domain |
| bmsk0010632 | | 22.09 | | 9.42 | -1.229594454 | synaptobrevin homolog YKT6 [Amyelois transitella] |
| bmsk0012510 | | 31.04666667 | | 13.23333333 | -1.230261875 | guanine nucleotide-binding protein-like 3 homolog [Amyelois transitella] |
| bmsk0007922 | | 3.61 | | 1.536666667 | -1.232194587 | RING finger protein 121 |
| bmsk0006813 | | 32.35 | | 13.74333333 | -1.235033752 | pre-mR -splicing factor SPF27 [Papilio polytes] |
| mstrg.22788 | | 8.036666667 | | 3.413333333 | -1.235415936 | PREDICTED: cytoplasmic tRNA 2-thiolation protein 2 [Bombyx mori] |
| bmsk0013113 | | 4.87 | | 2.066666667 | -1.236616058 | lipoma-preferred partner homolog [Papilio machaon] |
| bmsk0003460 | | 12.09666667 | | 5.13 | -1.237578825 | D ligase 3 [Papilio xuthus] |
| bmsk0014487 | | 44.90666667 | | 19.04 | -1.237896159 | pinin [Amyelois transitella] |
| bmsk0006902 | | 8.243333333 | | 3.493333333 | -1.238623523 | N-acetylgalactosaminyltransferase, partial |
| bmsk0000119 | | 11.18 | | 4.723333333 | -1.243042931 | uncharacterized protein LOC105392398 [Plutella xylostella] |
| bmsk0007811 | | 9.603333333 | | 4.056666667 | -1.243240492 | tudor domain-containing protein 3-like |
| bmsk0014255 | | 21.82333333 | | 9.216666667 | -1.243554498 | XP 004923124.1 uncharacterized protein LOC101737900 |
| bmsk0001716 | | 24.16 | | 10.2 | -1.244051302 | probable R polymerase II nuclear localization protein SLC7A6OS isoform X2 |
| bmsk0006735 | | 31.02333333 | | 13.08333333 | -1.245623553 | serine/arginine repetitive matrix protein 5 isoform X1 |
| bmsk0009587 | | 72.5 | | 30.57 | -1.245864443 | phosphatidylethanolamine binding protein isoform 2 |
| bmsk0008621 | | 11.70666667 | | 4.93 | -1.247670792 | uncharacterized protein LOC101745573 isoform X2 |
| bmsk0002444 | | 22.53 | | 9.48 | -1.248888349 | 28S ribosomal protein S29, mitochondrial isoform X1 |
| bmsk0000671 | | 11.36333333 | | 4.78 | -1.249303575 | Y+L amino acid transporter 2 |
| mstrg.12653 | | 11.76666667 | | 4.943333333 | -1.251149586 | PREDICTED: endonuclease G, mitochondrial, partial [Bombyx mori] |
| bmsk0008765 | | 11.44333333 | | 4.803333333 | -1.252399522 | hypothetical protein KGM 11112 [Danaus plexippus] |
| mstrg.19986 | | 3.453333333 | | 1.446666667 | -1.255257055 | PREDICTED: ATP-dependent RNA helicase DHX36-like [Athalia rosae] |
| bmsk0014743 | | 31.4 | | 13.15333333 | -1.255336104 | uncharacterized protein LOC106129259 [Amyelois transitella] |
| bmsk0011508 | | 5.056666667 | | 2.116666667 | -1.256392589 | developmental protein eyes absent |
| bmsk0008722 | | 7.736666667 | | 3.233333333 | -1.25868987 | UDP-glucuronic acid decarboxylase 1 |
| bmsk0002732 | | 37.58333333 | | 15.68 | -1.261167468 | protein ariadne-2 isoform X2 |
| bmsk0009652 | | 10.24 | | 4.266666667 | -1.263034406 | - |
| bmsk0013192 | | 45.22333333 | | 18.81333333 | -1.265311847 | XP 004921744.1 CAAX prenyl protease 1 homolog |
| bmsk0014400 | | 17.00333333 | | 7.073333333 | -1.265355444 | RSM22-like protein, mitochondrial [Operophtera brumata] |
| bmsk0002098 | | 14.96666667 | | 6.216666667 | -1.267539814 | platelet-activating factor acetylhydrolase alpha subunit-like protein |
| bmsk0010517 | | 26.29 | | 10.90666667 | -1.269303894 | XP 004928173.1 GDP-mannose 4,6 dehydratase |
| bmsk0006368 | | 33.32333333 | | 13.82333333 | -1.269427174 | ubiquitin domain-containing protein UBFD1-like |
| bmsk0013283 | | 12.87333333 | | 5.34 | -1.269474017 | ras-related GTP-binding protein 4b |
| bmsk0006488 | | 7.876666667 | | 3.266666667 | -1.269765975 | probable prefoldin subunit 4 [Plutella xylostella] |
| bmsk0006181 | | 11.02333333 | | 4.566666667 | -1.271347152 | U1 small nuclear ribonucleoprotein 70 kDa |
| bmsk0015154 | | 8.753333333 | | 3.623333333 | -1.272514976 | ABF51257.1 mitochondrial 28S ribosomal protein S25 |
| bmsk0014461 | | 5.976666667 | | 2.473333333 | -1.272884396 | XP 004925106.1 UPF0669 protein C6orf120 homolog isoform X2 |
| bmsk0003844 | | 31.35333333 | | 12.94333333 | -1.276409625 | SAP domain-containing ribonucleoprotein |
| bmsk0011388 | | 26.60333333 | | 10.97333333 | -1.277605188 | XP 004934317.1 SAP30-binding protein |
| bmsk0004275 | | 20.15666667 | | 8.303333333 | -1.279494557 | translocation protein SEC62 [Amyelois transitella] |
| bmsk0006696 | | 12.05 | | 4.963333333 | -1.279651893 | TBC1 domain family member 14 |
| mstrg.6998 | | 22.32333333 | | 9.19 | -1.280415701 | PREDICTED: COMM domain-containing protein 2-like isoform X3 [Bombyx mori] |
| bmsk0001319 | | 40.92666667 | | 16.82333333 | -1.282577583 | myotrophin-like [Amyelois transitella] |
| mstrg.18231 | | 11.56333333 | | 4.74 | -1.286598376 | PREDICTED: phosphatidylinositol N-acetylglucosaminyltransferase subunit H isoform X2 [Bombyx mori] |
| bmsk0011349 | | 1.09 | | 0.446666667 | -1.287057635 | - |
| bmsk0004527 | | 5.053333333 | | 2.07 | -1.28760458 | matrix metalloproteinase-14-like [Plutella xylostella] |
| bmsk0013367 | | 19.10666667 | | 7.826666667 | -1.287606201 | - |
| bmsk0006400 | | 8.936666667 | | 3.66 | -1.287893165 | transmembrane protein 170A [Amyelois transitella] |
| bmsk0010258 | | 25.64333333 | | 10.49333333 | -1.289110767 | sex combs extra |
| bmsk0011671 | | 220.48 | | 90.10666667 | -1.290942038 | ABF51286.1 FK506-binding protein |
| bmsk0005074 | | 15.54333333 | | 6.34 | -1.293741183 | exosome complex exonuclease RRP44 |
| bmsk0000776 | | 6.956666667 | | 2.836666667 | -1.294199563 | methionine--tR ligase, cytoplasmic |
| bmsk0012213 | | 27.21666667 | | 11.09666667 | -1.294364015 | BAF62110.1 phosphomevalonate kinase |
| bmsk0002453 | | 5.97 | | 2.433333333 | -1.294796968 | structural maintenance of chromosomes protein 6 |
| bmsk0013221 | | 12.15333333 | | 4.953333333 | -1.294880446 | XP 004932621.1 C-Myc-binding protein |
| bmsk0010519 | | 17.6 | | 7.17 | -1.295530405 | uncharacterized protein LOC106711973 isoform X1 [Papilio machaon] |
| mstrg.10273 | | 19.99333333 | | 8.143333333 | -1.295827659 | PREDICTED: galactosylgalactosylxylosylprotein 3-beta-glucuronosyltransferase I [Bombyx mori] |
| bmsk0006475 | | 16.50333333 | | 6.713333333 | -1.297656266 | tether containing UBX domain for GLUT4 |
| bmsk0009231 | | 17.72 | | 7.206666667 | -1.297974582 | 39S ribosomal protein L18, mitochondrial |
| bmsk0003754 | | 33.80333333 | | 13.74333333 | -1.298433557 | AFG3-like protein 2 |
| bmsk0011234 | | 16.49 | | 6.703333333 | -1.298640818 | sorting nexin-2 |
| bmsk0002704 | | 14.27 | | 5.8 | -1.298860529 | probable leucine--tR ligase, mitochondrial [Amyelois transitella] |
| bmsk0009161 | | 7.833333333 | | 3.183333333 | -1.299088119 | vacuolar protein sorting-associated protein 45 |
| bmsk0013751 | | 23 | | 9.346666667 | -1.299110013 | XP 004926023.1 uncharacterized protein LOC101736712 |
| mstrg.4550 | | 26.93666667 | | 10.93666667 | -1.300398239 | PREDICTED: protein bric-a-brac 1-like isoform X3 [Bombyx mori] |
| bmsk0010516 | | 29.55333333 | | 11.99666667 | -1.30068726 | XP 004928171.1 integrator complex subunit 11 |
| mstrg.18868 | | 11.75666667 | | 4.766666667 | -1.302426429 | PREDICTED: macrophage erythroblast attacher isoform X1 [Bombyx mori] |
| mstrg.14077 | | 13.02333333 | | 5.28 | -1.30248892 | PREDICTED: spermatogenesis-associated protein 6 isoform X2 [Bombyx mori] |
| bmsk0009600 | | 7.55 | | 3.053333333 | -1.306091547 | CCR4-NOT transcription complex subunit 10-like [Amyelois transitella] |
| bmsk0010177 | | 7.476666667 | | 3.016666667 | -1.309439923 | endochitinase A isoform X1 [Papilio xuthus] |
| bmsk0006596 | | 11.27 | | 4.546666667 | -1.309606372 | mismatch repair endonuclease PMS2 isoform X1 |
| bmsk0006036 | | 2.363333333 | | 0.953333333 | -1.30977048 | uncharacterized protein LOC106712628 [Papilio machaon] |
| bmsk0007661 | | 33.09 | | 13.32333333 | -1.31244022 | zinc transporter 9 [Amyelois transitella] |
| bmsk0013748 | | 4.693333333 | | 1.886666667 | -1.314773376 | sid-1-related gene1 precursor |
| bmsk0004496 | | 8.293333333 | | 3.33 | -1.316429902 | exportin-6 |
| bmsk0003055 | | 56.55 | | 22.69333333 | -1.317258488 | neuroglian |
| bmsk0004955 | | 24.86666667 | | 9.97 | -1.31854772 | 60S ribosomal export protein NMD3 |
| bmsk0012659 | | 30.18666667 | | 12.07666667 | -1.321689152 | THUMP domain-containing protein 1 [Amyelois transitella] |
| bmsk0014271 | | 37.62333333 | | 14.98 | -1.32859005 | vacuolar protein sorting-associated protein 26B-like [Amyelois transitella] |
| bmsk0007439 | | 10.20333333 | | 4.056666667 | -1.330673877 | probable arginine--tR ligase, mitochondrial [Amyelois transitella] |
| mstrg.24451 | | 10.09333333 | | 4.01 | -1.331728563 | PREDICTED: serine/threonine-protein kinase 40-like [Bombyx mori] |
| bmsk0006329 | | 13.58666667 | | 5.39 | -1.333834373 | maternal protein tudor-like isoform X3 [Amyelois transitella] |
| bmsk0008689 | | 21.50666667 | | 8.523333333 | -1.335294278 | transcription termination factor 5, mitochondrial-like |
| bmsk0009976 | | 8.646666667 | | 3.426666667 | -1.335338215 | tyrosine--tR ligase, mitochondrial [Papilio machaon] |
| bmsk0002374 | | 10.25 | | 4.056666667 | -1.337257242 | hypothetical protein KGM 11568 [Danaus plexippus] |
| bmsk0002455 | | 2.796666667 | | 1.106666667 | -1.337487569 | protein pigeon |
| bmsk0013209 | | 10.75333333 | | 4.25 | -1.339249191 | Sarah [Operophtera brumata] |
| bmsk0013529 | | 61.56333333 | | 24.31666667 | -1.340125871 | XP 012549714.1 DJ-1 beta isoform X2 |
| bmsk0000275 | | 13.97666667 | | 5.52 | -1.340280157 | geminin isoform X2 [Amyelois transitella] |
| bmsk0015227 | | 11.13 | | 4.393333333 | -1.341065723 | RING-variant domain |
| bmsk0002701 | | 15.41 | | 6.08 | -1.341723633 | negative elongation factor D [Amyelois transitella] |
| bmsk0005720 | | 23.43666667 | | 9.213333333 | -1.346972279 | Vesicle transport protein SEC20 [Papilio machaon] |
| bmsk0014911 | | 3.223333333 | | 1.266666667 | -1.347516471 | - |
| bmsk0008828 | | 16.81333333 | | 6.6 | -1.349067845 | peptidyl-prolyl cis-trans isomerase isoform X1 |
| bmsk0012493 | | 6.176666667 | | 2.423333333 | -1.349835612 | hypothetical protein KGM 05169 [Danaus plexippus] |
| bmsk0003845 | | 5.586666667 | | 2.19 | -1.351056873 | probable methyltransferase BMT2 homolog |
| bmsk0002550 | | 7.43 | | 2.91 | -1.352343058 | uncharacterized protein KIAA0754 |
| bmsk0011561 | | 2.843333333 | | 1.113333333 | -1.352697639 | gastrula zinc finger protein XlCGF26.1-like isoform X2 [Amyelois transitella] |
| bmsk0012782 | | 16.40666667 | | 6.42 | -1.353636955 | U2 small nuclear ribonucleoprotein A' |
| bmsk0011480 | | 20.86666667 | | 8.163333333 | -1.353969883 | partitioning defective protein 6 [Papilio polytes] |
| bmsk0006408 | | 14.11666667 | | 5.51 | -1.357275245 | glycogen-binding subunit 76A isoform X3 |
| bmsk0008753 | | 30.10666667 | | 11.74 | -1.358650577 | nuclear cap-binding protein subunit 2 |
| bmsk0008209 | | 23.44 | | 9.113333333 | -1.362921828 | WD repeat-containing protein 18 |
| bmsk0002926 | | 13.58666667 | | 5.27 | -1.366316684 | leucine-rich PPR motif-containing protein, mitochondrial |
| bmsk0006434 | | 96.97333333 | | 37.59333333 | -1.367111233 | ubiquitin-conjugating enzyme E2 variant 2 |
| bmsk0003897 | | 4.63 | | 1.793333333 | -1.368368521 | copper homeostasis protein cutC homolog isoform X1 |
| mstrg.6810 | | 21.84333333 | | 8.46 | -1.368463463 | ubiquinone biosynthesis protein COQ4 homolog, mitochondrial [Bombyx mori] |
| bmsk0007242 | | 47.98333333 | | 18.49333333 | -1.375528096 | protein dpy-30 homolog |
| bmsk0005423 | | 12.93 | | 4.983333333 | -1.375539291 | pelota-like protein |
| bmsk0001216 | | 3.703333333 | | 1.426666667 | -1.376176115 | uncharacterized protein LOC101741433 |
| bmsk0004926 | | 9.943333333 | | 3.83 | -1.37638518 | F-box only protein 28 isoform X1 |
| bmsk0003494 | | 11.26666667 | | 4.336666667 | -1.377402284 | vacuolar protein sorting-associated protein 72 homolog [Amyelois transitella] |
| bmsk0003048 | | 10.40666667 | | 3.996666667 | -1.380638879 | RCC1-like G exchanging factor-like protein |
| bmsk0007090 | | 9.686666667 | | 3.72 | -1.380697676 | transmembrane protein 177 |
| bmsk0003449 | | 20.44333333 | | 7.836666667 | -1.383318412 | putative peptidyl-tR hydrolase PTRHD1 |
| bmsk0013136 | | 9.64 | | 3.68 | -1.38932738 | XP 012543924.1 MMS19 nucleotide excision repair protein homolog |
| bmsk0006253 | | 13.02 | | 4.97 | -1.389411692 | serine palmitoyltransferase 1-like [Plutella xylostella] |
| bmsk0007290 | | 76.73666667 | | 29.29 | -1.389507904 | spermidine synthase |
| bmsk0005819 | | 14.95333333 | | 5.7 | -1.391433295 | mediator of R polymerase II transcription subunit 19 |
| bmsk0007436 | | 6.463333333 | | 2.45 | -1.399496648 | uncharacterized protein LOC105842657, partial |
| bmsk0013681 | | 3.38 | | 1.28 | -1.400879436 | XP 004930797.1 putative zinc finger and SCAN domain-containing protein 5D |
| bmsk0007127 | | 53.29 | | 20.16 | -1.402369194 | barrier-to-autointegration factor B |
| bmsk0014440 | | 7.77 | | 2.936666667 | -1.40373508 | segment polarity protein dishevelled, partial |
| bmsk0003056 | | 9.416666667 | | 3.553333333 | -1.406043429 | neuroglian [Mythimna separata] |
| bmsk0008919 | | 13.17666667 | | 4.963333333 | -1.408604201 | mR cap guanine-N7 methyltransferase [Papilio machaon] |
| bmsk0002097 | | 7.37 | | 2.773333333 | -1.410043592 | OTU domain-containing protein 6B |
| bmsk0014273 | | 5.026666667 | | 1.89 | -1.411215788 | porphobilinogen deaminase [Papilio xuthus] |
| bmsk0015615 | | 19.55666667 | | 7.343333333 | -1.413153497 | zinc finger protein 41-like isoform X1 [Amyelois transitella] |
| bmsk0014323 | | 20.30666667 | | 7.613333333 | -1.415353291 | R polymerase Rpb8 |
| bmsk0002954 | | 15.15666667 | | 5.68 | -1.415989669 | serine/threonine-protein kinase OSR1 |
| bmsk0013261 | | 4.056666667 | | 1.52 | -1.416223439 | uncharacterized protein LOC106137584 [Amyelois transitella] |
| mstrg.20636 | | 12.64333333 | | 4.73 | -1.418464782 | PREDICTED: ATP-dependent RNA helicase DHX36 [Bombyx mori] |
| bmsk0003810 | | 16.88666667 | | 6.316666667 | -1.418649229 | ralA-binding protein 1 isoform X2 |
| bmsk0003428 | | 9.21 | | 3.44 | -1.420792591 | uncharacterized protein LOC101736946 |
| bmsk0008503 | | 4.32 | | 1.613333333 | -1.420986766 | death-associated small cytoplasmic leucine-rich protein isoform X2 |
| bmsk0013237 | | 27.36666667 | | 10.22 | -1.421024525 | nuclear receptor-binding factor 2-like [Papilio polytes] |
| mstrg.16855 | | 11.25333333 | | 4.196666667 | -1.423036621 | PREDICTED: mitogen-activated protein kinase kinase kinase kinase 5 isoform X1 [Bombyx mori] |
| bmsk0003561 | | 11.09333333 | | 4.136666667 | -1.423152318 | uncharacterized protein LOC106122376 [Papilio xuthus] |
| mstrg.19799 | | 15.88666667 | | 5.913333333 | -1.425772942 | PREDICTED: F-box only protein 33 [Bemisia tabaci] |
| bmsk0001134 | | 9.25 | | 3.44 | -1.427044801 | bone morphogenetic protein receptor type-1B isoform X2 [Papilio machaon] |
| mstrg.1644 | | 21.51 | | 7.993333333 | -1.428138367 | PREDICTED: methylosome protein 50-like [Bombyx mori] |
| bmsk0008345 | | 3.503333333 | | 1.3 | -1.43021664 | probable tR pseudouridine synthase 2 [Papilio polytes] |
| bmsk0005800 | | 2.903333333 | | 1.076666667 | -1.431138554 | dual specificity protein phosphatase 19-like [Plutella xylostella] |
| bmsk0004114 | | 6.986666667 | | 2.59 | -1.431652213 | chromatin target of PRMT1 protein |
| mstrg.983 | | 16.30666667 | | 6.043333333 | -1.432045479 | PREDICTED: E3 ubiquitin-protein ligase UHRF1-like isoform X1 [Bombyx mori] |
| mstrg.16882 | | 11.85333333 | | 4.386666667 | -1.434095835 | PREDICTED: zinc finger protein 91-like [Amyelois transitella] |
| bmsk0011823 | | 8.44 | | 3.123333333 | -1.434156452 | zinc transporter 2-like [Amyelois transitella] |
| bmsk0002924 | | 50.67 | | 18.75 | -1.434241233 | ADP-ribosylation factor 6 |
| bmsk0007050 | | 13.57333333 | | 5.006666667 | -1.438852749 | proton-coupled amino acid transporter 2 |
| bmsk0002576 | | 3.253333333 | | 1.2 | -1.438884241 | SHC-transforming protein 1 |
| bmsk0013400 | | 12.57666667 | | 4.636666667 | -1.43958968 | uncharacterized protein LOC101738318 |
| bmsk0014988 | | 5.976666667 | | 2.2 | -1.441837559 | translation initiation factor IF-2, mitochondrial isoform X1 [Amyelois transitella] |
| mstrg.14474 | | 23.63 | | 8.69 | -1.443191547 | PREDICTED: zinc finger protein 729-like isoform X2 [Bombyx mori] |
| bmsk0015544 | | 23.28 | | 8.523333333 | -1.449601398 | - |
| bmsk0004103 | | 12.54666667 | | 4.59 | -1.450738069 | cyclin-H [Amyelois transitella] |
| bmsk0006033 | | 6.683333333 | | 2.433333333 | -1.457633868 | WD repeat domain-containing protein 83 |
| bmsk0002688 | | 8.37 | | 3.046666667 | -1.457995958 | phosphatidylinositol glycan anchor biosynthesis class U protein |
| mstrg.19051 | | 3.44 | | 1.246666667 | -1.464332796 | olfactory receptor [Bombyx mori] |
| bmsk0015226 | | 4.056666667 | | 1.47 | -1.464478607 | XP 004927251.1 E3 ubiquitin-protein ligase MARCH5 |
| bmsk0012555 | | 10.59333333 | | 3.833333333 | -1.466485264 | dedicator of cytokinesis protein 3-like [Papilio polytes] |
| bmsk0007692 | | 14.91 | | 5.376666667 | -1.47149632 | CRAL-TRIO domain-containing protein C3H8.02 [Papilio xuthus] |
| mstrg.6696 | | 23.34666667 | | 8.393333333 | -1.475900801 | PREDICTED: protein-associating with the carboxyl-terminal domain of ezrin [Bombyx mori] |
| bmsk0010277 | | 3.226666667 | | 1.16 | -1.475919741 | uncharacterized protein LOC106142693 isoform X2 [Amyelois transitella] |
| bmsk0003366 | | 7.67 | | 2.756666667 | -1.476301749 | programmed cell death protein 2-like [Amyelois transitella] |
| bmsk0008683 | | 7.866666667 | | 2.826666667 | -1.47665069 | D replication complex GINS protein SLD5 |
| bmsk0002390 | | 10.58333333 | | 3.796666667 | -1.478988845 | Transcription factor 2B [Operophtera brumata] |
| bmsk0012277 | | 11.91333333 | | 4.266666667 | -1.481395824 | very-long-chain (3R)-3-hydroxyacyl-CoA dehydratase [Papilio polytes] |
| bmsk0000451 | | 3.596666667 | | 1.286666667 | -1.483022112 | - |
| bmsk0010643 | | 2.236666667 | | 0.796666667 | -1.489302148 | leucine-rich repeat-containing protein 58-like [Amyelois transitella] |
| bmsk0014235 | | 41.68 | | 14.80333333 | -1.493433207 | ABF51346.1 LRP16 protein |
| bmsk0013848 | | 5.176666667 | | 1.836666667 | -1.494933606 | serine/threonine-protein kinase Genghis Khan |
| bmsk0013491 | | 8.99 | | 3.176666667 | -1.500807402 | XP 021206718.1 tyrosine-protein phosphatase non-receptor type 21 |
| bmsk0011593 | | 17.48 | | 6.173333333 | -1.501583587 | tetratricopeptide repeat protein 4 [Papilio machaon] |
| bmsk0008650 | | 129.09 | | 45.59 | -1.501587932 | cytosolic non-specific dipeptidase [Papilio machaon] |
| bmsk0002307 | | 1.89 | | 0.666666667 | -1.503348735 | uncharacterized protein C630.12 isoform X1 |
| mstrg.1615 | | 3.12 | | 1.1 | -1.504042505 | PREDICTED: cell division cycle 2 like-1 isoform X1 [Bombyx mori] |
| bmsk0006609 | | 7.906666667 | | 2.786666667 | -1.504529162 | glucose-fructose oxidoreductase domain-containing protein 1 [Amyelois transitella] |
| bmsk0006273 | | 8.453333333 | | 2.976666667 | -1.505822665 | sperm-associated antigen 7 homolog [Amyelois transitella] |
| bmsk0007253 | | 9.42 | | 3.313333333 | -1.507443709 | PH domain |
| bmsk0004635 | | 16.49 | | 5.796666667 | -1.508295967 | carboxyl/cholinesterase 7 precursor |
| bmsk0011071 | | 4.66 | | 1.633333333 | -1.512510706 | uncharacterized protein LOC106132531 [Amyelois transitella] |
| mstrg.22856 | | 8.973333333 | | 3.143333333 | -1.513348734 | PREDICTED: GPI ethanolamine phosphate transferase 1 [Bombyx mori] |
| bmsk0001935 | | 3.31 | | 1.156666667 | -1.516858055 | endoplasmic reticulum-Golgi intermediate compartment protein 3 [Amyelois transitella] |
| bmsk0006053 | | 2.74 | | 0.956666667 | -1.518087657 | uncharacterized protein LOC101741044 isoform X1 |
| bmsk0002176 | | 2.063333333 | | 0.72 | -1.518908097 | mitoguardin |
| bmsk0008312 | | 11.23666667 | | 3.91 | -1.522973614 | neurobeachin-like isoform X3 |
| bmsk0012538 | | 3.143333333 | | 1.09 | -1.527967135 | putative uncharacterized protein DDB G0291608 isoform X2 |
| bmsk0007058 | | 59.28 | | 20.55333333 | -1.528173059 | glycogen phosphorylase |
| bmsk0010421 | | 12.3 | | 4.256666667 | -1.530862291 | XP 004925946.1 protein Smaug |
| bmsk0009471 | | 6.323333333 | | 2.186666667 | -1.531951959 | cell cycle checkpoint control protein RAD9A [Amyelois transitella] |
| bmsk0007594 | | 42.08 | | 14.54 | -1.533107435 | transaldolase |
| bmsk0007726 | | 1.806666667 | | 0.623333333 | -1.535254581 | histidine--tR ligase, cytoplasmic |
| bmsk0014433 | | 4.596666667 | | 1.583333333 | -1.537623038 | XP 012544975.1 protein hu-li tai shao |
| bmsk0002762 | | 6.706666667 | | 2.31 | -1.537703048 | CD2 antigen cytoplasmic tail-binding protein 2 homolog |
| mstrg.5081 | | 17.75333333 | | 6.093333333 | -1.542786358 | PREDICTED: FAST kinase domain-containing protein 5 [Bombyx mori] |
| bmsk0008248 | | 1.743333333 | | 0.596666667 | -1.546851359 | E3 ubiquitin-protein ligase SH3RF1 isoform X2 |
| bmsk0015510 | | 11.51 | | 3.933333333 | -1.549063475 | uncharacterized protein LOC101738244 |
| bmsk0008397 | | 3.176666667 | | 1.08 | -1.556482401 | KWMTBOMO08896 |
| bmsk0001544 | | 6.633333333 | | 2.253333333 | -1.557673279 | - |
| bmsk0001487 | | 12.05 | | 4.093333333 | -1.557685086 | Uncharacterized protein OBRU01 21543 [Operophtera brumata] |
| bmsk0003289 | | 3.466666667 | | 1.176666667 | -1.55884344 | vacuolar protein sorting-associated protein 16 homolog [Plutella xylostella] |
| bmsk0008758 | | 6.003333333 | | 2.036666667 | -1.559553896 | DTW domain-containing protein 1 |
| bmsk0014038 | | 4.33 | | 1.466666667 | -1.561826002 | zinc finger protein 37-like isoform X3 |
| mstrg.8172 | | 10.31333333 | | 3.49 | -1.563211755 | hypothetical protein g.7559 [Pectinophora gossypiella] |
| bmsk0011070 | | 7.163333333 | | 2.42 | -1.565624029 | - |
| bmsk0004497 | | 27.19 | | 9.153333333 | -1.570707027 | histone R hairpin-binding protein |
| bmsk0007793 | | 9.56 | | 3.216666667 | -1.571444177 | mitochondrial inner membrane protease subunit 1 |
| bmsk0010566 | | 1.156666667 | | 0.386666667 | -1.580810857 | REST corepressor isoform X3 [Papilio xuthus] |
| bmsk0012516 | | 2.803333333 | | 0.936666667 | -1.58153567 | XP 012550828.1 ATP-binding cassette sub-family G member 8 |
| bmsk0001877 | | 1.17 | | 0.39 | -1.584962501 | golgin subfamily A member 1 isoform X2 |
| mstrg.5707 | | 6.096666667 | | 2.03 | -1.586540942 | PREDICTED: hamartin [Bombyx mori] |
| bmsk0007188 | | 3.313333333 | | 1.1 | -1.590779827 | gastrula zinc finger protein XlCGF57.1-like |
| bmsk0006699 | | 19.32333333 | | 6.413333333 | -1.591197687 | glycine cleavage system H protein, mitochondrial-like isoform X2 [Papilio machaon] |
| bmsk0007122 | | 11.41 | | 3.783333333 | -1.592568995 | rab3 GTPase-activating protein regulatory subunit |
| bmsk0014866 | | 16.06 | | 5.313333333 | -1.595782764 | XP 004921839.1 putative transferase CAF17 homolog, mitochondrial |
| bmsk0006984 | | 9.36 | | 3.086666667 | -1.600458837 | ATP-binding cassette sub-family G member 4 |
| bmsk0009276 | | 2.286666667 | | 0.753333333 | -1.601885804 | glucose dehydrogenase [FAD, quinone]-like |
| bmsk0012328 | | 2.176666667 | | 0.716666667 | -1.602746332 | zinc finger protein 2 homolog isoform X1 |
| bmsk0015805 | | 1.8 | | 0.59 | -1.609210047 | protein lingerer isoform X3 |
| bmsk0002489 | | 4.413333333 | | 1.443333333 | -1.612464192 | zinc finger protein 184 |
| bmsk0007424 | | 3.163333333 | | 1.023333333 | -1.628169432 | uncharacterized protein LOC101746767 |
| bmsk0012674 | | 9.373333333 | | 3.03 | -1.629244395 | eIF2B-gamma protein isoform X1 |
| bmsk0001869 | | 17.46666667 | | 5.613333333 | -1.637674673 | glucose-6-phosphate 1-dehydrogenase [Amyelois transitella] |
| bmsk0000069 | | 4.716666667 | | 1.51 | -1.643219098 | mitogen-activated protein kinase kinase kinase 12-like |
| bmsk0006207 | | 5.293333333 | | 1.693333333 | -1.64431051 | uncharacterized protein C7orf26 homolog |
| bmsk0000473 | | 1.18 | | 0.376666667 | -1.647426588 | - |
| bmsk0013387 | | 17.88333333 | | 5.706666667 | -1.647895469 | AFC01230.1 DnaJ-16 |
| bmsk0010478 | | 15.82333333 | | 5.023333333 | -1.655336633 | XP 004928141.1 mitochondrial 2-oxodicarboxylate carrier |
| bmsk0009674 | | 6.62 | | 2.096666667 | -1.658733701 | GPN-loop GTPase 3 |
| bmsk0003934 | | 26.29 | | 8.303333333 | -1.662751621 | katanin p60 ATPase-containing subunit A-like 1 |
| bmsk0015682 | | 2.733333333 | | 0.86 | -1.668252844 | glycosyltransferase isoform X1 |
| bmsk0008711 | | 8.43 | | 2.633333333 | -1.678642479 | decapping nuclease DXO homolog isoform X1 |
| bmsk0003158 | | 1.003333333 | | 0.313333333 | -1.679030825 | UPF0565 protein C2orf69 homolog |
| bmsk0002818 | | 2.733333333 | | 0.853333333 | -1.6794801 | myb/SANT-like D -binding domain-containing protein 3, partial [Plutella xylostella] |
| bmsk0011347 | | 7.383333333 | | 2.3 | -1.682638432 | XP 004924240.1 beta-hexosaminidase subunit beta |
| bmsk0003334 | | 6.293333333 | | 1.96 | -1.682970705 | Homeodomain |
| bmsk0003915 | | 1.863333333 | | 0.58 | -1.683760977 | probable D mismatch repair protein Msh6 isoform X2 |
| bmsk0008138 | | 16.99 | | 5.286666667 | -1.684255582 | DH dehydrogenase [ubiquinone] complex I, assembly factor 7 homolog |
| bmsk0011685 | | 10.27333333 | | 3.18 | -1.691805691 | XP 004925346.1 sister chromatid cohesion protein DCC1 isoform X1 |
| bmsk0008721 | | 0.456666667 | | 0.14 | -1.70571466 | beta-1,3-galactosyltransferase 6 isoform X2 |
| bmsk0013866 | | 5.246666667 | | 1.606666667 | -1.707330489 | Golgi pH regulator [Papilio xuthus] |
| bmsk0000005 | | 12.13 | | 3.706666667 | -1.710385263 | - |
| bmsk0005145 | | 9.22 | | 2.813333333 | -1.712486252 | angiomotin |
| bmsk0014114 | | 13.93666667 | | 4.243333333 | -1.715615624 | protein phosphatase 1 regulatory subunit 3B-B [Amyelois transitella] |
| bmsk0005942 | | 8.196666667 | | 2.476666667 | -1.726637619 | uncharacterized protein LOC101740704, partial |
| bmsk0002872 | | 16.05333333 | | 4.84 | -1.729793939 | 6-pyruvoyltetrahydropterin synthase |
| bmsk0010675 | | 0.696666667 | | 0.21 | -1.730079209 | serine/threonine-protein phosphatase 4 regulatory subunit 2 [Amyelois transitella] |
| bmsk0003562 | | 45.31333333 | | 13.64666667 | -1.73138702 | uncharacterized protein LOC101743105 |
| bmsk0012131 | | 7.96 | | 2.393333333 | -1.733747087 | G patch domain and KOW motifs-containing protein [Plutella xylostella] |
| bmsk0013349 | | 10.59666667 | | 3.156666667 | -1.747136686 | XP 004932676.1 protein DPCD |
| bmsk0002127 | | 15.62666667 | | 4.63 | -1.75492597 | ubiquitin-conjugating enzyme E2Q-like protein CG4502 [Papilio polytes] |
| bmsk0001916 | | 8.666666667 | | 2.56 | -1.759333407 | mitochondrial sodium/hydrogen exchanger 9B2 isoform X1 |
| bmsk0015193 | | 9.176666667 | | 2.696666667 | -1.766793002 | XP 004933067.1 gastrula zinc finger protein XlCGF17.1-like |
| bmsk0009183 | | 8.97 | | 2.633333333 | -1.768217833 | mitochondrial inner membrane protease subunit 2 |
| bmsk0005477 | | 3.833333333 | | 1.123333333 | -1.770813365 | uncharacterized protein LOC105842129 |
| bmsk0002367 | | 0.913333333 | | 0.266666667 | -1.776103988 | D repair protein complementing XP-A cells homolog [Papilio machaon] |
| bmsk0000365 | | 3.473333333 | | 1.013333333 | -1.777212049 | tetratricopeptide repeat protein 39B isoform X2 |
| bmsk0013438 | | 19.44 | | 5.67 | -1.777607579 | XP 004930689.1 PWWP domain-containing protein 2A isoform X1 |
| bmsk0004306 | | 11.54666667 | | 3.363333333 | -1.779512756 | Outer membrane efflux protein [Operophtera brumata] |
| bmsk0010324 | | 10.56666667 | | 3.056666667 | -1.789489201 | lactosylceramide 4-alpha-galactosyltransferase-like |
| bmsk0001127 | | 4.683333333 | | 1.353333333 | -1.791018498 | 3-hydroxyacyl-CoA dehydrogenase |
| bmsk0011716 | | 2.47 | | 0.713333333 | -1.791862746 | acyl-CoA synthetase family member 3, mitochondrial |
| bmsk0008872 | | 11.57 | | 3.303333333 | -1.808394403 | uncharacterized protein LOC101740766 |
| mstrg.14068 | | 2.353333333 | | 0.67 | -1.812472682 | PREDICTED: 5'-3' exoribonuclease 1 isoform X2 [Bombyx mori] |
| bmsk0002190 | | 9.043333333 | | 2.573333333 | -1.813216295 | coiled-coil domain-containing protein 43 |
| bmsk0007442 | | 5.38 | | 1.526666667 | -1.817221075 | GDP-fucose transporter 1 |
| bmsk0007950 | | 1.316666667 | | 0.373333333 | -1.818353921 | uncharacterized protein LOC106719359 [Papilio machaon] |
| bmsk0002582 | | 4.056666667 | | 1.15 | -1.818660901 | uncharacterized protein LOC106124467 [Papilio xuthus] |
| bmsk0009295 | | 0.33 | | 0.093333333 | -1.822001698 | Glucose dehydrogenase [acceptor], partial [Operophtera brumata] |
| bmsk0010570 | | 1.426666667 | | 0.403333333 | -1.822603749 | protein crooked neck |
| bmsk0012767 | | 5.853333333 | | 1.653333333 | -1.823880819 | rab-like protein 3 isoform X1 [Amyelois transitella] |
| bmsk0015709 | | 29.45 | | 8.316666667 | -1.824190319 | heat shock protein hsp20.1 |
| bmsk0010769 | | 1.316666667 | | 0.37 | -1.831292977 | poly(A) R polymerase, mitochondrial |
| bmsk0002729 | | 15.14 | | 4.253333333 | -1.831699377 | uncharacterized protein LOC101746063 isoform X3 |
| bmsk0005946 | | 12.70666667 | | 3.556666667 | -1.836987943 | uncharacterized protein LOC101743619 |
| bmsk0004687 | | 5.436666667 | | 1.52 | -1.838651053 | partner of Y14 and mago isoform X1 |
| bmsk0013444 | | 3.29 | | 0.916666667 | -1.843618466 | AFC01222.1 DnaJ-8 |
| bmsk0002483 | | 25.1 | | 6.99 | -1.844323003 | protein disulfide-isomerase A6 [Amyelois transitella] |
| bmsk0003310 | | 6.213333333 | | 1.72 | -1.852958889 | protein PFC0760c-like |
| bmsk0015252 | | 36.97333333 | | 10.22 | -1.855089919 | methenyltetrahydrofolate synthase domain-containing protein [Amyelois transitella] |
| bmsk0013578 | | 8.986666667 | | 2.466666667 | -1.865223321 | XP 012549767.1 R -binding protein 26 isoform X1 |
| bmsk0008750 | | 1.616666667 | | 0.44 | -1.877446818 | D(P) transhydrogenase, mitochondrial |
| bmsk0001059 | | 1.813333333 | | 0.493333333 | -1.878009476 | uncharacterized protein LOC101737726 isoform X2 |
| bmsk0010851 | | 2.563333333 | | 0.696666667 | -1.879480656 | XP 004926993.1 CCR4-NOT transcription complex subunit 11 |
| bmsk0005435 | | 1.043333333 | | 0.283333333 | -1.880627911 | protein-lysine N-methyltransferase EEF2KMT isoform X3 |
| bmsk0015078 | | 0.716666667 | | 0.193333333 | -1.890211854 | XP 012551678.1 myb-like protein X isoform X3 |
| bmsk0012150 | | 22.42333333 | | 6.036666667 | -1.893176712 | XP 021205945.1 tyrosine--tR ligase, cytoplasmic-like |
| bmsk0000101 | | 4.47 | | 1.2 | -1.897240426 | pyruvate dehydrogenase phosphatase regulatory subunit, mitochondrial isoform X1 [Papilio machaon] |
| bmsk0005373 | | 3.353333333 | | 0.896666667 | -1.902952227 | uncharacterized protein LOC101746064 |
| bmsk0014985 | | 1.636666667 | | 0.436666667 | -1.906156213 | uridine diphosphate glucose pyrophosphatase-like [Papilio xuthus] |
| bmsk0006608 | | 3.5 | | 0.93 | -1.912052301 | 10 kDa heat shock protein, mitochondrial |
| mstrg.5302 | | 5.686666667 | | 1.503333333 | -1.919418308 | - |
| bmsk0006049 | | 9.636666667 | | 2.536666667 | -1.92560025 | ferrochelatase, mitochondrial-like |
| bmsk0007466 | | 0.203333333 | | 0.053333333 | -1.930737338 | dehydrodolichyl diphosphate synthase |
| bmsk0007467 | | 0.203333333 | | 0.053333333 | -1.930737338 | Putative undecaprenyl diphosphate synthase |
| bmsk0010289 | | 1.726666667 | | 0.45 | -1.939992691 | XP 021207808.1 protein tweety isoform X2 |
| bmsk0014394 | | 1.496666667 | | 0.39 | -1.940206915 | nucleolin 2-like |
| bmsk0002236 | | 2.073333333 | | 0.54 | -1.940920767 | probable serine carboxypeptidase CPVL |
| bmsk0014728 | | 1.323333333 | | 0.34 | -1.960569855 | XP 012544415.1 dimethyladenosine transferase 2, mitochondrial |
| bmsk0003532 | | 3.46 | | 0.886666667 | -1.964308293 | soluble guanylate cyclase 89Da |
| bmsk0012469 | | 7.08 | | 1.796666667 | -1.978426588 | Spermine/spermidine synthase domain |
| bmsk0002250 | | 1.413333333 | | 0.356666667 | -1.986453468 | intraflagellar transport protein 80 homolog isoform X1 [Amyelois transitella] |
| bmsk0014245 | | 1.143333333 | | 0.286666667 | -1.995800011 | XP 012552909.1 uncharacterized protein LOC101745557 |
| bmsk0012470 | | 18.38666667 | | 4.603333333 | -1.997909137 | XP 004924801.1 spermine synthase |
| bmsk0012651 | | 1.213333333 | | 0.303333333 | -2 | uncharacterized protein LOC106132394 isoform X2 [Amyelois transitella] |
| bmsk0006208 | | 5.686666667 | | 1.42 | -2.001692311 | cleavage and polyadenylation specific factor 4 |
| bmsk0008759 | | 3.97 | | 0.986666667 | -2.008504332 | Wd-repeat protein [Operophtera brumata] |
| bmsk0002405 | | 10.11 | | 2.486666667 | -2.023497962 | transcriptional adapter 2A |
| bmsk0002918 | | 2.646666667 | | 0.65 | -2.025664883 | Golgi casein kinase, C-terminal, Fam20 |
| bmsk0008495 | | 1.783333333 | | 0.436666667 | -2.02997208 | uncharacterized protein LOC101739413 |
| bmsk0002463 | | 15.60333333 | | 3.756666667 | -2.05432925 | epidermal retinal dehydrogenase isoform X1 |
| bmsk0006402 | | 3.236666667 | | 0.776666667 | -2.059141341 | lisH domain and HEAT repeat-containing protein KIAA1468-like |
| bmsk0008804 | | 5.846666667 | | 1.376666667 | -2.086435061 | transcription factor E2F7 |
| bmsk0008685 | | 4.616666667 | | 1.076666667 | -2.100279906 | AP-3 complex subunit delta |
| bmsk0009111 | | 2.446666667 | | 0.57 | -2.101783738 | cell division control protein 45 homolog [Papilio machaon] |
| bmsk0009805 | | 3.733333333 | | 0.866666667 | -2.106915204 | diphthine methyltransferase isoform X1 |
| bmsk0014905 | | 55.69333333 | | 12.92333333 | -2.107526408 | tryptophan--tR ligase, cytoplasmic [Amyelois transitella] |
| bmsk0014142 | | 1.11 | | 0.256666667 | -2.112591826 | - |
| bmsk0010900 | | 3.55 | | 0.816666667 | -2.119999776 | protein prickle-like |
| bmsk0009142 | | 0.333333333 | | 0.076666667 | -2.120294234 | mitochondrial fission process protein 1 |
| bmsk0006838 | | 7.596666667 | | 1.74 | -2.126279213 | sprT-like domain-containing protein Spartan [Papilio machaon] |
| bmsk0011267 | | 1.186666667 | | 0.27 | -2.135883428 | phosphorylated adapter R export protein [Amyelois transitella] |
| bmsk0001191 | | 1.46 | | 0.33 | -2.14543044 | zinc finger protein 615 isoform X4 |
| mstrg.15013 | | 1.463333333 | | 0.33 | -2.148720509 | UPF0368 protein Cxorf26-like protein [Pararge aegeria] |
| bmsk0012968 | | 6.283333333 | | 1.41 | -2.155834955 | XP 004924294.1 peroxisomal targeting signal 2 receptor isoform X1 |
| bmsk0008332 | | 2.943333333 | | 0.66 | -2.156913008 | protein OPI10 homolog [Amyelois transitella] |
| bmsk0000474 | | 0.793333333 | | 0.176666667 | -2.166897309 | uncharacterized protein LOC106720810 [Papilio machaon] |
| bmsk0014145 | | 1.05 | | 0.233333333 | -2.169925001 | - |
| bmsk0002454 | | 7.89 | | 1.743333333 | -2.178176854 | cyclin E |
| bmsk0007680 | | 1.95 | | 0.43 | -2.181065559 | hypothetical protein KGM 05068 [Danaus plexippus] |
| bmsk0000396 | | 1.286666667 | | 0.28 | -2.200139614 | acetyl-coenzyme A synthetase isoform X1 |
| bmsk0009569 | | 3.066666667 | | 0.666666667 | -2.201633861 | uncharacterized protein LOC101746691 |
| bmsk0001958 | | 0.843333333 | | 0.183333333 | -2.201633861 | - |
| bmsk0011378 | | 27.35333333 | | 5.85 | -2.225208124 | putative odorant-degrading enzyme [Antheraea polyphemus] |
| bmsk0012461 | | 0.656666667 | | 0.14 | -2.229734397 | D -binding protein RFX2 |
| bmsk0005257 | | 0.486666667 | | 0.103333333 | -2.235628248 | lymphokine-activated killer T-cell-originated protein kinase homolog [Amyelois transitella] |
| bmsk0013099 | | 0.993333333 | | 0.21 | -2.241888597 | XP 004925483.1 probable cysteine--tR ligase, mitochondrial |
| bmsk0015176 | | 1.706666667 | | 0.356666667 | -2.258533014 | homeobox protein slou |
| bmsk0011061 | | 1.55 | | 0.323333333 | -2.261174064 | XP 004933425.1 GPI ethanolamine phosphate transferase 3 isoform X2 |
| bmsk0000413 | | 3.97 | | 0.826666667 | -2.263761387 | - |
| bmsk0000139 | | 0.276666667 | | 0.056666667 | -2.28757659 | Uncharacterized protein OBRU01 18498 [Operophtera brumata] |
| bmsk0001735 | | 1.013333333 | | 0.206666667 | -2.293731203 | protein asteroid |
| bmsk0008527 | | 1.693333333 | | 0.34 | -2.316259345 | surfeit 5 |
| bmsk0013528 | | 6.583333333 | | 1.32 | -2.318280318 | zinc finger protein 468-like [Papilio machaon] |
| bmsk0010919 | | 5.263333333 | | 1.053333333 | -2.321014708 | XP 021203679.1 cleavage and polyadenylation specificity factor 73 |
| bmsk0007392 | | 0.133333333 | | 0.026666667 | -2.321928095 | Exocyst complex component 7 [Papilio machaon] |
| bmsk0004614 | | 3.086666667 | | 0.613333333 | -2.331306427 | centrosomal protein of 131 kDa |
| bmsk0005953 | | 0.273333333 | | 0.053333333 | -2.357552005 | zinc finger protein 271-like isoform X2 |
| bmsk0011802 | | 0.26 | | 0.05 | -2.378511623 | XP 021203661.1 nuclear receptor coactivator 6-like, partial |
| bmsk0006006 | | 2.78 | | 0.526666667 | -2.400122825 | exopolyphosphatase PRUNE1 isoform X3 |
| bmsk0014080 | | 21.77666667 | | 4.053333333 | -2.425602411 | interleukin enhancer binding factor isoform X2 |
| bmsk0013662 | | 2.23 | | 0.41 | -2.443347895 | uncharacterized protein LOC101743235 isoform X3 |
| bmsk0003272 | | 12.45666667 | | 2.266666667 | -2.458273912 | malate dehydrogenase isoform X1 |
| bmsk0000928 | | 3.25 | | 0.586666667 | -2.46982679 | - |
| bmsk0013369 | | 1.833333333 | | 0.33 | -2.473931188 | uncharacterized protein LOC106135696 [Amyelois transitella] |
| bmsk0008142 | | 2.48 | | 0.436666667 | -2.50573581 | uncharacterized protein LOC101743157 isoform X2 |
| bmsk0014807 | | 5.506666667 | | 0.963333333 | -2.515072289 | zinc finger protein 774-like |
| bmsk0015760 | | 2.82 | | 0.483333333 | -2.544604763 | lipoma HMGIC fusion partner-like 3 protein |
| bmsk0005277 | | 1.33 | | 0.226666667 | -2.552782095 | beta-1,3-glucosyltransferase isoform X5 |
| bmsk0009316 | | 2.096666667 | | 0.35 | -2.582670689 | Homeodomain |
| bmsk0002449 | | 2.436666667 | | 0.406666667 | -2.582990258 | arrestin domain-containing protein 4 |
| bmsk0014140 | | 2.47 | | 0.41 | -2.590815227 | XP 004926346.1 uncharacterized protein LOC101745670 isoform X2 |
| bmsk0014447 | | 2.86 | | 0.47 | -2.605282485 | Ankyrin repeats (many copies) |
| mstrg.2380 | | 0.996666667 | | 0.163333333 | -2.60929183 | PREDICTED: zinc finger protein 16-like isoform X2 [Bombyx mori] |
| bmsk0007943 | | 0.43 | | 0.07 | -2.618909833 | - |
| bmsk0012536 | | 0.493333333 | | 0.08 | -2.624490865 | 27 kDa glycoprotein-like |
| mstrg.10249 | | 3.086666667 | | 0.5 | -2.626049693 | PREDICTED: proline-rich protein 11-like [Amyelois transitella] |
| bmsk0006084 | | 1.18 | | 0.19 | -2.634715536 | cholinesterase 2 |
| bmsk0014009 | | 0.676666667 | | 0.106666667 | -2.665335917 | XP 021204008.1 zinc finger protein 836 isoform X2 |
| bmsk0003101 | | 2.75 | | 0.433333333 | -2.665882496 | scavenger receptor class B member 1-like isoform X1 |
| mstrg.18872 | | 1.723333333 | | 0.27 | -2.674170467 | PREDICTED: cilia- and flagella-associated protein 58-like [Bombyx mori] |
| bmsk0010415 | | 1.926666667 | | 0.3 | -2.683072586 | XP 021203506.1 zinc finger protein 77 isoform X2 |
| bmsk0008405 | | 5.213333333 | | 0.8 | -2.704134202 | iron-sulfur protein NUBPL |
| bmsk0015361 | | 0.436666667 | | 0.066666667 | -2.711494907 | protein O-Glc case |
| bmsk0007813 | | 2.23 | | 0.34 | -2.713437059 | tetraspanin-9 isoform X2 |
| bmsk0005250 | | 0.68 | | 0.103333333 | -2.718229032 | - |
| bmsk0011526 | | 0.623333333 | | 0.09 | -2.792006958 | XP 021207167.1 intraflagellar transport protein 52 homolog isoform X2 |
| bmsk0006171 | | 2.173333333 | | 0.313333333 | -2.794139303 | cell division cycle 25 isoform X1 |
| bmsk0014025 | | 0.74 | | 0.106666667 | -2.794415866 | XP 021204024.1 zinc finger protein 569 isoform X1 |
| bmsk0009104 | | 8.973333333 | | 1.29 | -2.798272938 | crossover junction endonuclease EME1 isoform X2 |
| bmsk0002039 | | 1.516666667 | | 0.216666667 | -2.807354922 | zinc finger CCHC domain-containing protein 13-like |
| bmsk0014187 | | 1.516666667 | | 0.216666667 | -2.807354922 | - |
| bmsk0005605 | | 0.97 | | 0.133333333 | -2.862947248 | - |
| bmsk0002283 | | 3.623333333 | | 0.483333333 | -2.906227135 | tyrosine-protein phosphatase non-receptor type 23 isoform X1 [Amyelois transitella] |
| bmsk0003282 | | 1.393333333 | | 0.183333333 | -2.925999419 | transmembrane and ubiquitin-like domain-containing protein 1 isoform X1 |
| bmsk0010142 | | 5.706666667 | | 0.75 | -2.927685795 | U6 snR -associated Sm-like protein LSm5 |
| bmsk0015036 | | 8.626666667 | | 1.116666667 | -2.949604617 | CREB-regulated transcription coactivator 1 |
| bmsk0010262 | | 3.976666667 | | 0.513333333 | -2.953591787 | ABD36119.1 G10 protein |
| bmsk0009433 | | 10.76 | | 1.38 | -2.962937906 | leucine-zipper-like transcriptional regulator 1 [Amyelois transitella] |
| bmsk0004933 | | 1.593333333 | | 0.203333333 | -2.97012947 | - |
| bmsk0000931 | | 0.683333333 | | 0.086666667 | -2.979040381 | Uncharacterized protein OBRU01 01760 [Operophtera brumata] |
| bmsk0008295 | | 2.343333333 | | 0.296666667 | -2.981647448 | probable asparagine--tR ligase, mitochondrial |
| bmsk0001827 | | 10.31333333 | | 1.286666667 | -3.002800444 | probable ATP-dependent R helicase DDX17 |
| bmsk0013393 | | 0.296666667 | | 0.036666667 | -3.016301812 | XP 004932766.1 catalase |
| bmsk0000541 | | 0.33 | | 0.04 | -3.044394119 | ubiquitin-conjugating enzyme E2 G2 |
| bmsk0011737 | | 0.33 | | 0.04 | -3.044394119 | Transposase IS4 |
| bmsk0000425 | | 0.363333333 | | 0.043333333 | -3.067744607 | polypeptide N-acetylgalactosaminyltransferase 11 |
| bmsk0007939 | | 0.696666667 | | 0.08 | -3.122396631 | - |
| bmsk0007919 | | 0.116666667 | | 0.013333333 | -3.129283017 | actin cytoskeleton-regulatory complex protein pan1 |
| bmsk0013847 | | 0.263333333 | | 0.03 | -3.133855747 | thioredoxin-related transmembrane protein 1-like |
| bmsk0015864 | | 0.996666667 | | 0.113333333 | -3.136538833 | lysozyme-like |
| bmsk0007170 | | 2.12 | | 0.24 | -3.142957954 | D repair endonuclease XPF [Amyelois transitella] |
| bmsk0015370 | | 1.626666667 | | 0.183333333 | -3.149377624 | reverse transcriptase |
| bmsk0001898 | | 11.6 | | 1.29 | -3.168681835 | AAA domain |
| bmsk0007789 | | 0.45 | | 0.05 | -3.169925001 | histone deacetylase complex subunit SAP30 homolog |
| bmsk0007631 | | 3.286666667 | | 0.356666667 | -3.20397685 | ankyrin repeat, SAM and basic leucine zipper domain-containing protein 1 |
| bmsk0009464 | | 2.15 | | 0.223333333 | -3.26706616 | protein nessun dorma |
| bmsk0007498 | | 10.19666667 | | 1.046666667 | -3.284223643 | uncharacterized protein LOC105841917 isoform X1 |
| bmsk0007999 | | 1.203333333 | | 0.123333333 | -3.286401661 | - |
| bmsk0008585 | | 5.203333333 | | 0.503333333 | -3.369850083 | polycomb protein Pcl isoform X3 |
| bmsk0008182 | | 0.66 | | 0.06 | -3.459431619 | - |
| bmsk0000817 | | 0.076666667 | | 0.006666667 | -3.523561956 | dihydrofolate reductase-like isoform X1 |
| bmsk0012253 | | 4.386666667 | | 0.373333333 | -3.554588852 | uncharacterized protein LOC106129070 [Amyelois transitella] |
| bmsk0010414 | | 0.59 | | 0.05 | -3.560714954 | putative cuticle protein [Operophtera brumata] |
| bmsk0009117 | | 1.976666667 | | 0.163333333 | -3.59717845 | Domain of unknown function (DUF1899) |
| bmsk0011302 | | 0.123333333 | | 0.01 | -3.624490865 | uncharacterized protein LOC106137262 [Amyelois transitella] |
| bmsk0013302 | | 19.1 | | 1.533333333 | -3.638829373 | juvenile hormone epoxide hydrolase-like protein 1 |
| bmsk0008050 | | 0.55 | | 0.043333333 | -3.665882496 | - |
| bmsk0003141 | | 1.456666667 | | 0.113333333 | -3.684026628 | atonal |
| bmsk0005364 | | 0.173333333 | | 0.013333333 | -3.700439718 | MORN repeat-containing protein 3 |
| bmsk0006988 | | 4.21 | | 0.323333333 | -3.702726082 | protein expanded |
| bmsk0005434 | | 2.153333333 | | 0.163333333 | -3.720680511 | S1 R -binding domain-containing protein 1 isoform X1 |
| bmsk0015308 | | 0.66 | | 0.05 | -3.722466024 | ADM32522.1 htra2 |
| bmsk0009423 | | 3.103333333 | | 0.233333333 | -3.733354341 | FancJ-like protein |
| bmsk0013592 | | 0.773333333 | | 0.056666667 | -3.770518154 | - |
| bmsk0010094 | | 1.603333333 | | 0.11 | -3.865498964 | putative homeodomain transcription factor, partial |
| bmsk0013188 | | 0.456666667 | | 0.03 | -3.928107082 | lysine-specific histone demethylase 1A |
| bmsk0014920 | | 3.543333333 | | 0.23 | -3.945401425 | uncharacterized protein LOC106129316 [Amyelois transitella] |
| bmsk0014449 | | 0.996666667 | | 0.06 | -4.054076673 | uncharacterized protein LOC105841476 |
| bmsk0007946 | | 0.61 | | 0.036666667 | -4.05626822 | - |
| bmsk0003328 | | 0.726666667 | | 0.043333333 | -4.067744607 | Hox cluster protein Shx15 |
| bmsk0010890 | | 0.636666667 | | 0.036666667 | -4.117997209 | XP 004926205.1 transmembrane protein 209 |
| bmsk0000945 | | 0.35 | | 0.02 | -4.129283017 | dymeclin [Papilio machaon] |
| bmsk0011228 | | 0.116666667 | | 0.006666667 | -4.129283017 | XP 021206434.1 putative R -binding protein 15B isoform X1 |
| bmsk0000376 | | 1.233333333 | | 0.07 | -4.139064038 | THAP domain-containing protein 9, partial [Camponotus floridanus] |
| bmsk0013485 | | 0.24 | | 0.013333333 | -4.169925001 | XP 021206611.1 ovarian tumor protein isoform X1 |
| bmsk0011110 | | 9.196666667 | | 0.483333333 | -4.250020651 | XP 004933401.1 D -directed R polymerases I, II, and III subunit RPABC1 |
| bmsk0004477 | | 0.643333333 | | 0.033333333 | -4.270528942 | NK-tumor recognition protein |
| bmsk0001142 | | 0.52 | | 0.026666667 | -4.285402219 | uncharacterized protein LOC106116963 [Papilio xuthus] |
| bmsk0015117 | | 0.283333333 | | 0.013333333 | -4.409390936 | erythroid differentiation-related factor 1 |
| bmsk0011674 | | 1.7 | | 0.08 | -4.409390936 | uncharacterized protein LOC101744317 isoform X2 |
| bmsk0002215 | | 0.356666667 | | 0.016666667 | -4.419538892 | cytosolic carboxypeptidase 6 |
| bmsk0005479 | | 0.613333333 | | 0.026666667 | -4.523561956 | partitioning defective 3 homolog |
| bmsk0014203 | | 1.336666667 | | 0.056666667 | -4.559995585 | - |
| bmsk0001187 | | 0.08 | | 0.003333333 | -4.584962501 | protein MCM10 homolog isoform X1 |
| bmsk0004065 | | 0.246666667 | | 0.01 | -4.624490865 | - |
| bmsk0000555 | | 2.34 | | 0.093333333 | -4.647972298 | glucuronyltransferase |
| bmsk0006842 | | 1.763333333 | | 0.066666667 | -4.725195817 | nesprin-1-like isoform X1 |
| bmsk0012662 | | 2.826666667 | | 0.1 | -4.821029859 | transcription cofactor vestigial-like protein 4 [Amyelois transitella] |
| bmsk0000513 | | 12.08333333 | | 0.41 | -4.881250774 | uncharacterized protein LOC105842551 |
| bmsk0015876 | | 0.493333333 | | 0.016666667 | -4.887525271 | - |
| bmsk0002288 | | 5.333333333 | | 0.16 | -5.058893689 | Microtubule-binding calmodulin-regulated spectrin-associated |
| bmsk0000428 | | 0.583333333 | | 0.016666667 | -5.129283017 | discoidin domain-containing receptor 2-like |
| bmsk0004644 | | 3.98 | | 0.113333333 | -5.13412428 | phosphopantothenoylcysteine decarboxylase [Papilio polytes] |
| bmsk0011270 | | 3.586666667 | | 0.09 | -5.31657486 | XP 021206608.1 protein MLP1 isoform X3 |
| bmsk0009188 | | 4.193333333 | | 0.1 | -5.390025611 | - |
| bmsk0001677 | | 1.603333333 | | 0.033333333 | -5.587964989 | uncharacterized protein LOC110385729 |
| bmsk0012933 | | 6.32 | | 0.113333333 | -5.801280408 | kinesin-like protein KIF18A isoform X1 |
| bmsk0009991 | | 0.373333333 | | 0.006666667 | -5.807354922 | ubiquitin carboxyl-terminal hydrolase 7-like isoform X1 [Papilio xuthus] |
| bmsk0005378 | | 7.986666667 | | 0.14 | -5.83409477 | - |
| bmsk0004249 | | 0.06 | | 0.001 | -5.906890596 | rac GTPase-activating protein 1 isoform X2 |
| bmsk0000761 | | 0.06 | | 0.001 | -5.906890596 | Leucine rich repeat |
| bmsk0007297 | | 0.07 | | 0.001 | -6.129283017 | remodeling and spacing factor 1-like |
| bmsk0009225 | | 0.07 | | 0.001 | -6.129283017 | uncharacterized protein LOC101742265 isoform X1 |
| bmsk0007525 | | 0.086666667 | | 0.001 | -6.437405312 | probable phosphorylase b kinase regulatory subunit alpha isoform X2 |
| bmsk0013077 | | 0.096666667 | | 0.001 | -6.594946589 | tubulin-folding cofactor B [Amyelois transitella] |
| bmsk0007988 | | 0.1 | | 0.001 | -6.64385619 | NEDD8 ultimate buster 1 isoform X2 |
| bmsk0001575 | | 0.113333333 | | 0.001 | -6.824428435 | Guanylate kinase |
| bmsk0004370 | | 0.116666667 | | 0.001 | -6.866248611 | uncharacterized protein LOC101744580 |
| bmsk0011173 | | 0.13 | | 0.001 | -7.022367813 | XP 021206237.1 uncharacterized protein LOC101745797 |
| bmsk0014816 | | 2.206666667 | | 0.016666667 | -7.048759312 | XP 021202427.1 uncharacterized protein LOC101737526 |
| bmsk0007464 | | 0.136666667 | | 0.001 | -7.094517599 | gem-associated protein 5-like |
| bmsk0005090 | | 10.04 | | 0.073333333 | -7.097074436 | synaptic vesicle glycoprotein 2B isoform X2 |
| bmsk0004499 | | 0.15 | | 0.001 | -7.22881869 | methyltransferase-like protein 16 [Papilio xuthus] |
| bmsk0012170 | | 0.153333333 | | 0.001 | -7.26052755 | - |
| bmsk0014698 | | 0.156666667 | | 0.001 | -7.291554446 | uncharacterized protein LOC106721620 [Papilio machaon] |
| bmsk0002854 | | 0.17 | | 0.001 | -7.409390936 | lamin-C |
| bmsk0015098 | | 0.17 | | 0.001 | -7.409390936 | tak1 isoform X1 |
| bmsk0012747 | | 0.18 | | 0.001 | -7.491853096 | KWMTBOMO13160 |
| bmsk0001920 | | 0.186666667 | | 0.001 | -7.544320516 | protein CWC15 homolog [Amyelois transitella] |
| bmsk0003628 | | 3.22 | | 0.016666667 | -7.593951284 | collagen alpha chain CG42342 |
| bmsk0001650 | | 0.2 | | 0.001 | -7.64385619 | laminin subunit alpha-2 |
| bmsk0006789 | | 0.206666667 | | 0.001 | -7.691161905 | transmembrane protease serine 2-like |
| bmsk0005619 | | 0.22 | | 0.001 | -7.781359714 | cationic peptide CP8 precursor [Bombyx mandarina] |
| bmsk0015843 | | 0.223333333 | | 0.001 | -7.803054785 | XP 012552062.2 trichohyalin isoform X1 |
| bmsk0008350 | | 2.996666667 | | 0.013333333 | -7.812177306 | transcription factor E74, partial |
| bmsk0015832 | | 0.226666667 | | 0.001 | -7.824428435 | putative ferric-chelate reductase 1 homolog [Amyelois transitella] |
| bmsk0015716 | | 0.233333333 | | 0.001 | -7.866248611 | E1-E2 ATPase |
| bmsk0002569 | | 0.233333333 | | 0.001 | -7.866248611 | uncharacterized protein LOC101739478 |
| bmsk0009591 | | 0.233333333 | | 0.001 | -7.866248611 | uncharacterized protein LOC106133235 [Amyelois transitella] |
| bmsk0007838 | | 0.246666667 | | 0.001 | -7.94641896 | - |
| bmsk0005978 | | 0.246666667 | | 0.001 | -7.94641896 | uncharacterized protein LOC106136821 [Amyelois transitella] |
| bmsk0005726 | | 0.256666667 | | 0.001 | -8.003752135 | D repair protein RAD51 homolog 4 isoform X4 |
| bmsk0003064 | | 0.263333333 | | 0.001 | -8.040746342 | transmembrane protein 205 |
| bmsk0005536 | | 11.74666667 | | 0.043333333 | -8.082558491 | mucin-3A [Papilio machaon] |
| bmsk0010097 | | 0.286666667 | | 0.001 | -8.163230349 | uncharacterized protein LOC101744784 |
| bmsk0011517 | | 0.3 | | 0.001 | -8.22881869 | low molecular 30 kDa lipoprotein PBMHPC-23 precursor |
| bmsk0003326 | | 0.336666667 | | 0.001 | -8.395177077 | uncharacterized protein LOC101742071 |
| bmsk0005693 | | 0.363333333 | | 0.001 | -8.505149919 | transcription initiation factor TFIID subunit 2 [Papilio xuthus] |
| bmsk0007451 | | 0.376666667 | | 0.001 | -8.557144557 | - |
| bmsk0003331 | | 0.39 | | 0.001 | -8.607330314 | transcription factor deformed |
| bmsk0006909 | | 15.70333333 | | 0.036666667 | -8.742385998 | UDP-glucoronosyl and UDP-glucosyl transferase |
| bmsk0009010 | | 0.43 | | 0.001 | -8.74819285 | cytochrome P450, family 337, subfamily a, polypeptide 1 precursor |
| bmsk0014998 | | 0.43 | | 0.001 | -8.74819285 | XP 004934176.3 histone H2A-like, partial |
| bmsk0006073 | | 0.443333333 | | 0.001 | -8.79224803 | zinc finger protein Xfin-like |
| bmsk0009719 | | 0.45 | | 0.001 | -8.813781191 | - |
| bmsk0014643 | | 0.453333333 | | 0.001 | -8.824428435 | D polymerase epsilon catalytic subunit A [Amyelois transitella] |
| bmsk0000782 | | 0.46 | | 0.001 | -8.845490051 | uncharacterized protein LOC101744603 |
| bmsk0006612 | | 0.466666667 | | 0.001 | -8.866248611 | WW domain-binding protein 11 |
| bmsk0004247 | | 0.5 | | 0.001 | -8.965784285 | uncharacterized protein LOC105389364 [Plutella xylostella] |
| bmsk0006009 | | 0.536666667 | | 0.001 | -9.067882472 | zinc finger protein 28-like |
| bmsk0009550 | | 0.566666667 | | 0.001 | -9.14635653 | - |
| bmsk0001298 | | 0.603333333 | | 0.001 | -9.236811481 | THAP domain |
| bmsk0015662 | | 0.643333333 | | 0.001 | -9.329422631 | - |
| bmsk0008321 | | 0.666666667 | | 0.001 | -9.380821784 | - |
| bmsk0010923 | | 0.79 | | 0.001 | -9.625708843 | pyroglutamyl-peptidase 1 isoform X1 |
| bmsk0009824 | | 0.79 | | 0.001 | -9.625708843 | uncharacterized protein LOC105841610 isoform X1 |
| bmsk0006352 | | 0.793333333 | | 0.001 | -9.631783357 | C-type lectin domain family 4 member F |
| bmsk0010797 | | 0.836666667 | | 0.001 | -9.708509148 | connectin-like |
| bmsk0000289 | | 0.913333333 | | 0.001 | -9.834997677 | ABF51522.1 thioesterase superfamily member 2 |
| bmsk0004642 | | 0.913333333 | | 0.001 | -9.834997677 | uncharacterized protein LOC101735994 |
| bmsk0014031 | | 29.28666667 | | 0.03 | -9.931065783 | adenylate cyclase |
| bmsk0001985 | | 1.003333333 | | 0.001 | -9.970585271 | dipeptidase 1-like |
| bmsk0013591 | | 1.016666667 | | 0.001 | -9.989631027 | XP 012549834.1 scavenger receptor class B member 1 |
| bmsk0014925 | | 1.46 | | 0.001 | -10.51175265 | potassium channel subfamily K member 9 [Amyelois transitella] |
| bmsk0010082 | | 1.54 | | 0.001 | -10.58871464 | lysophospholipid acyltransferase 1 isoform X1 |
| bmsk0007785 | | 1.57 | | 0.001 | -10.61654884 | - |
| bmsk0004089 | | 1.753333333 | | 0.001 | -10.77588458 | regulator of G-protein signaling loco |
| bmsk0005260 | | 1.87 | | 0.001 | -10.86882255 | zinc finger protein 433-like |
| bmsk0000298 | | 1.993333333 | | 0.001 | -10.96096727 | RUS1 family protein C16orf58 homolog isoform X1 |
| bmsk0002015 | | 2.24 | | 0.001 | -11.12928302 | diacylglycerol O-acyltransferase 1 isoform X1 |
| bmsk0012774 | | 2.286666667 | | 0.001 | -11.15903036 | Rapamycin-insensitive companion of mTOR, domain 5 |
| bmsk0015417 | | 25.73 | | 0.01 | -11.32923574 | XP 004926113.1 uncharacterized protein LOC101740865 |
| bmsk0011322 | | 2.74 | | 0.001 | -11.41996018 | oxysterol-binding protein-related protein 9 |
| bmsk0011012 | | 2.803333333 | | 0.001 | -11.45292758 | polynucleotide 5'-hydroxyl-kinase NOL9 [Amyelois transitella] |
| bmsk0012998 | | 2.903333333 | | 0.001 | -11.5034945 | histone H2AX-like protein |
| bmsk0009814 | | 2.926666667 | | 0.001 | -11.51504272 | - |
| bmsk0001519 | | 4.07 | | 0.001 | -11.99081308 | melanoma-associated antigen G1-like [Papilio machaon] |
| bmsk0007267 | | 4.146666667 | | 0.001 | -12.01773636 | uncharacterized protein LOC101739362 |
| bmsk0012885 | | 5.25 | | 0.001 | -12.35810171 | XP 004927162.1 esterase E4 isoform X1 |
| **Differentially expressed genes of silkworm between 5-day embryos and 5-day larvae during *N. bombycis* infection** | | | | | | |
| id | I-E5_mean | | I-L5_mean | | log2(fc) | Description |
| bmsk0002727 | 0.306666667 | | 18.98666667 | | 5.952171475 | wing disc-specific protein |
| bmsk0013118 | 23.78 | | 70.82666667 | | 1.574543931 | XP 021201818.1 axoneme-associated protein mst101(2) isoform X1 |
| bmsk0004457 | 23.19333333 | | 50.29 | | 1.116561375 | clustered mitochondria protein homolog [Amyelois transitella] |
| mstrg.10278 | 3.536666667 | | 12.35333333 | | 1.804438225 | PREDICTED: fasciclin-2-like [Bombyx mori] |
| mstrg.22552 | 94.15333333 | | 222.6833333 | | 1.241909507 | uncharacterized LOC106111899 [Papilio polytes] |
| mstrg.18926 | 7.033333333 | | 45.02 | | 2.678285559 | cytochrome P450, partial [Bombyx mori] |
| bmsk0010055 | 15.41666667 | | 33.73333333 | | 1.129684019 | uncharacterized protein LOC101737344 |
| bmsk0009407 | 7.903333333 | | 29.64 | | 1.907012286 | fatty acid-binding protein, muscle-like [Plutella xylostella] |
| bmsk0014088 | 34.46 | | 78.03 | | 1.179106198 | metal transporter CNNM4-like [Amyelois transitella] |
| bmsk0012509 | 337.98 | | 955.6466667 | | 1.499539429 | calreticulin |
| bmsk0014309 | 1005.363333 | | 2055.63 | | 1.031863634 | XP 004934243.2 ADP,ATP carrier protein |
| bmsk0000947 | 3.303333333 | | 14.24 | | 2.107954685 | cuticular protein hypothetical 3 precursor |
| bmsk0005038 | 7.91 | | 26.11333333 | | 1.723037028 | phosphoserine aminotransferase |
| bmsk0015756 | 7.393333333 | | 26.53 | | 1.843327811 | XP 004933525.1 growth arrest and D damage-inducible protein GADD45 alpha |
| bmsk0002625 | 31.86333333 | | 94.25333333 | | 1.564646441 | facilitated trehalose transporter Tret1-like isoform X2 |
| bmsk0014513 | 9.28 | | 22.45666667 | | 1.274947088 | XP 004925716.1 atlastin |
| bmsk0001873 | 1.923333333 | | 13.65333333 | | 2.827572491 | fatty acid transport protein |
| bmsk0000689 | 0.906666667 | | 8.893333333 | | 3.29408011 | phenylalanine hydroxylase |
| bmsk0004554 | 127.0966667 | | 288.5233333 | | 1.182761803 | mitochondrial cytochrome c oxidase subunit VIa |
| mstrg.1208 | 22.64333333 | | 89.67666667 | | 1.9856463 | PREDICTED: C-type lectin 11 isoform X1 [Bombyx mori] |
| bmsk0013626 | 23.84 | | 1.476666667 | | -4.012968133 | kinesin-like protein KIF23 [Plutella xylostella] |
| bmsk0001531 | 63.03666667 | | 170.1466667 | | 1.432515735 | sterol carrier protein x |
| bmsk0005520 | 137.5266667 | | 284.7566667 | | 1.050018232 | Bax inhibitor-1-like protein |
| bmsk0012276 | 31.14666667 | | 63.45 | | 1.026542391 | DH dehydrogenase (ubiquinone) 1 alpha subcomplex, 13 |
| bmsk0006278 | 78.34333333 | | 161.2633333 | | 1.04153603 | signal sequence receptor precursor |
| bmsk0011777 | 147.11 | | 335.65 | | 1.190062323 | XP 004927649.1 DH dehydrogenase [ubiquinone] 1 alpha subcomplex subunit 10, mitochondrial |
| bmsk0016018 | 0.583333333 | | 14.44666667 | | 4.630272326 | gloverin 4 precursor |
| bmsk0015991 | 4.263333333 | | 13.41666667 | | 1.653972519 | serpin 5 [Bombyx mandarina] |
| bmsk0015992 | 4.263333333 | | 13.41666667 | | 1.653972519 | - |
| bmsk0009855 | 1.786666667 | | 4.1 | | 1.19835341 | lethal(2) giant larvae protein isoform X1 |
| bmsk0001657 | 4.66 | | 9.55 | | 1.035170778 | Na,K-ATPase beta-subunit |
| bmsk0008930 | 5.953333333 | | 21.49333333 | | 1.852119663 | probable uridine nucleosidase 2 |
| mstrg.4923 | 1.906666667 | | 4.68 | | 1.295455884 | PREDICTED: lipopolysaccharide-induced tumor necrosis factor-alpha factor homolog [Bombyx mori] |
| bmsk0004663 | 1.016666667 | | 4.526666667 | | 2.154602332 | mucin-5AC |
| mstrg.9233 | 52.32666667 | | 113.7966667 | | 1.120840035 | Pastrel [Operophtera brumata] |
| mstrg.14280 | 9.4 | | 31.85666667 | | 1.760862656 | PREDICTED: pancreatic lipase-related protein 2-like [Bombyx mori] |
| bmsk0010362 | 0.263333333 | | 3.623333333 | | 3.782355477 | XP 012546023.1 cytochrome P450 CYP9A21 isoform X1 |
| bmsk0008425 | 3.006666667 | | 6.306666667 | | 1.06871275 | cysteine-rich with EGF-like domain protein 2 isoform X1 |
| mstrg.22714 | 15.59333333 | | 0.663333333 | | -4.555051526 | Myosin-J heavy chain [Operophtera brumata] |
| mstrg.11341 | 4.346666667 | | 13.52666667 | | 1.637824996 | PREDICTED: chemosensory protein 9 isoform X1 [Bombyx mori] |
| bmsk0010372 | 5.493333333 | | 15.28 | | 1.475890802 | Ets-domain |
| mstrg.6823 | 4.903333333 | | 12.42333333 | | 1.341217572 | 1-acylglycerol-3-phosphate O-acyltransferase 2 [Bombyx mori] |
| bmsk0013211 | 11.76333333 | | 24.8 | | 1.076043191 | organic cation transporter protein isoform X2 |
| bmsk0010141 | 16.67666667 | | 1.883333333 | | -3.14647068 | Targeting protein for Xklp2-B [Operophtera brumata] |
| bmsk0009465 | 17.61 | | 2.616666667 | | -2.750592851 | uncharacterized protein LOC101743179 |
| bmsk0004507 | 1.173333333 | | 4.916666667 | | 2.06706762 | septin-2 [Amyelois transitella] |
| bmsk0014867 | 17.06333333 | | 34.37333333 | | 1.010390257 | XP 004921838.1 uncharacterized protein LOC101745117 |
| bmsk0005576 | 15.28333333 | | 50.28 | | 1.718025417 | beta-galactosidase |
| mstrg.7387 | 14.07333333 | | 35.23666667 | | 1.324113376 | salivary purine nucleosidase [Pararge aegeria] |
| mstrg.9560 | 1.633333333 | | 4.093333333 | | 1.325456906 | PREDICTED: major royal jelly protein 1 [Bombyx mori] |
| bmsk0007555 | 5.04 | | 10.40666667 | | 1.046012398 | branched-chain-amino-acid aminotransferase, cytosolic |
| bmsk0007556 | 5.04 | | 10.40666667 | | 1.046012398 | succinate dehydrogenase assembly factor 2-B, mitochondrial isoform X2 |
| bmsk0014790 | 1.11 | | 5.493333333 | | 2.30712216 | serine protease easter-like |
| bmsk0000201 | 19.11666667 | | 38.99666667 | | 1.028519826 | kettin protein [Danaus plexippus] |
| bmsk0004448 | 64.61333333 | | 130.2233333 | | 1.011084164 | DH dehydrogenase [ubiquinone] 1 alpha subcomplex subunit 6 |
| bmsk0012894 | 12.12 | | 1.273333333 | | -3.250707656 | XP 004924762.1 protein Spindly |
| bmsk0006692 | 2.816666667 | | 15.94333333 | | 2.500892545 | facilitated trehalose transporter Tret1-like |
| bmsk0005258 | 0.05 | | 1.313333333 | | 4.715161224 | ankyrin repeat domain-containing protein 29 isoform X3 |
| bmsk0006303 | 32.21666667 | | 84.71333333 | | 1.394781827 | DH-ubiquinone reductase |
| bmsk0009941 | 17.24333333 | | 45.84 | | 1.410568353 | SOCS2-12 protein |
| bmsk0013357 | 18.46 | | 57.32333333 | | 1.634719951 | kazal-type proteinase inhibitor precursor |
| bmsk0013403 | 43.45 | | 170 | | 1.968106664 | transferrin precursor |
| bmsk0002063 | 3.873333333 | | 9.363333333 | | 1.273446556 | junctophilin-1 isoform X1 |
| bmsk0013793 | 25.76333333 | | 55.51666667 | | 1.107601683 | AFO09967.1 IBP2 |
| bmsk0008529 | 66.08666667 | | 167.9066667 | | 1.345228378 | DH dehydrogenase [ubiquinone] 1 beta subcomplex subunit 8, mitochondrial |
| bmsk0001286 | 1.556666667 | | 5.68 | | 1.867430881 | protein spaetzle 3 |
| mstrg.23037 | 52.99666667 | | 110.6366667 | | 1.061856069 | PREDICTED: electron transfer flavoprotein subunit alpha, mitochondrial [Bombyx mori] |
| bmsk0005620 | 3.486666667 | | 7.703333333 | | 1.143634408 | 5-hydroxytryptamine receptor |
| mstrg.7205 | 89.16 | | 191.3833333 | | 1.101996676 | PREDICTED: inter-alpha-trypsin inhibitor heavy chain H4-like isoform X1 [Bombyx mori] |
| bmsk0011240 | 38.35333333 | | 87.44333333 | | 1.188996428 | DH dehydrogenase [ubiquinone] iron-sulfur protein 4, mitochondrial |
| bmsk0010583 | 4.336666667 | | 15.49666667 | | 1.837299464 | gamma-interferon-inducible lysosomal thiol reductase-like [Papilio xuthus] |
| bmsk0013518 | 13.10333333 | | 1.533333333 | | -3.095190597 | importin subunit alpha-5-like isoform X2 |
| bmsk0005455 | 0.86 | | 2.95 | | 1.77830639 | putative fatty acyl-CoA reductase CG5065 |
| mstrg.18757 | 13.81 | | 33.70333333 | | 1.287177965 | PREDICTED: sugar transporter 4 isoform X1 [Bombyx mori] |
| bmsk0000847 | 38.86 | | 81.3 | | 1.064969451 | DH dehydrogenase [ubiquinone] 1 alpha subcomplex subunit 8 |
| bmsk0004535 | 9.7 | | 20.32333333 | | 1.067080393 | transmembrane protein 120 homolog isoform X3 |
| bmsk0006524 | 1.71 | | 11.94 | | 2.803734606 | pupal cuticle protein C1B-like |
| bmsk0006121 | 3.07 | | 8.576666667 | | 1.482178396 | probable R -binding protein orb2 isoform X2 |
| bmsk0004428 | 17.92666667 | | 72.69666667 | | 2.01978196 | putative exonuclease GOR-like protein [Operophtera brumata] |
| bmsk0000608 | 5.78 | | 15.77 | | 1.448041262 | basement membrane-specific heparan sulfate proteoglycan core protein-like isoform X3 |
| bmsk0010493 | 5.44 | | 26.46 | | 2.282134505 | XP 004928152.1 uncharacterized protein LOC101744658 |
| bmsk0014354 | 3.663333333 | | 7.576666667 | | 1.048406298 | serine hydrolase-like protein [Plutella xylostella] |
| bmsk0009253 | 1.99 | | 7.533333333 | | 1.920519936 | uncharacterized protein LOC106129912 [Amyelois transitella] |
| bmsk0011037 | 29.88 | | 69.43 | | 1.216379023 | XP 012552008.1 UDP-glucose 4-epimerase |
| bmsk0006408 | 14.11666667 | | 2.596666667 | | -2.442666736 | glycogen-binding subunit 76A isoform X3 |
| bmsk0011378 | 27.35333333 | | 3.14 | | -3.122880189 | putative odorant-degrading enzyme [Antheraea polyphemus] |
| bmsk0002882 | 0.293333333 | | 0.726666667 | | 1.308752706 | protein rolling stone-like |
| bmsk0000504 | 0.706666667 | | 2.03 | | 1.522377963 | fibrinogen alpha chain [Papilio xuthus] |
| bmsk0013177 | 18.76666667 | | 40.36 | | 1.104753753 | acetoacetyl-CoA thiolase |
| bmsk0006648 | 16.69666667 | | 2.943333333 | | -2.504037269 | uncharacterized protein LOC101747195 |
| bmsk0010889 | 20.62666667 | | 1.8 | | -3.518441884 | ABF51274.1 AAA family ATPase |
| bmsk0002005 | 1.816666667 | | 5.26 | | 1.53376907 | tensin isoform X4 |
| bmsk0012298 | 569.58 | | 1307.726667 | | 1.199090635 | ribosomal protein L36 |
| bmsk0014899 | 0.64 | | 2.956666667 | | 2.207827794 | papilin |
| bmsk0009503 | 33.21333333 | | 3.87 | | -3.101357049 | cell division cycle 2 |
| bmsk0003816 | 1.65 | | 3.546666667 | | 1.103997721 | serine protease inhibitor 12 isoform X1 |
| bmsk0015618 | 11.46 | | 0.001 | | -13.48431942 | XP 021201813.1 trithorax group protein osa |
| bmsk0001387 | 10.62666667 | | 1.12 | | -3.246118491 | ATP-dependent D helicase PIF1 |
| mstrg.10178 | 1.386666667 | | 3.156666667 | | 1.186780897 | PREDICTED: homeobox protein Nkx-2.2a isoform X2 [Bombyx mori] |
| bmsk0007586 | 38.31333333 | | 85.49666667 | | 1.158021625 | cytochrome b-c1 complex subunit 8 [Plutella xylostella] |
| bmsk0015248 | 0.953333333 | | 2.666666667 | | 1.483984853 | XP 004922070.2 uncharacterized protein LOC101737006 isoform X1 |
| bmsk0008717 | 6.09 | | 20.21333333 | | 1.73079312 | - |
| bmsk0000066 | 0.62 | | 1.583333333 | | 1.352624892 | Immunoglobulin I-set domain |
| bmsk0005659 | 3.41 | | 7.186666667 | | 1.075551033 | hypothetical protein KGM 05194 [Danaus plexippus] |
| bmsk0014700 | 118.8066667 | | 240.26 | | 1.015980686 | RS29 BOMMO RecName: Full=40S ribosomal protein S29 |
| bmsk0001395 | 0.5 | | 1.163333333 | | 1.218264536 | AII79417.1 integrin beta5 |
| bmsk0010016 | 1.853333333 | | 4.326666667 | | 1.223133595 | keratin, type I cytoskeletal 10 isoform X1 |
| bmsk0000346 | 0.001 | | 4.233333333 | | 12.04757838 | abnormal cell migration protein 10 isoform X2 |
| bmsk0010373 | 3.12 | | 11.54 | | 1.88702529 | transcription factor Ets |
| bmsk0010532 | 22.79666667 | | 3.616666667 | | -2.656090347 | uncharacterized protein LOC106719879 [Papilio machaon] |
| bmsk0014950 | 11.29 | | 1.02 | | -3.468404429 | chromosome-associated kinesin KIF4 [Amyelois transitella] |
| bmsk0013199 | 2.143333333 | | 5.44 | | 1.343750415 | organic cation transporter [Danaus plexippus] |
| bmsk0002698 | 0.793333333 | | 2.066666667 | | 1.381306642 | potassium voltage-gated channel subfamily KQT member 1 isoform X1 |
| bmsk0001268 | 11.55666667 | | 25.62333333 | | 1.148732832 | uncharacterized protein LOC106143374 [Amyelois transitella] |
| bmsk0004717 | 0.6 | | 1.563333333 | | 1.381591016 | Calponin homology (CH) domain |
| bmsk0003121 | 89.46 | | 214.6966667 | | 1.262985129 | putative DH=ubiquinone dehydrogenase [Danaus plexippus] |
| bmsk0005264 | 0.103333333 | | 1.946666667 | | 4.235628248 | cuticular protein glycine-rich 14 precursor |
| bmsk0011701 | 16.62333333 | | 2.056666667 | | -3.014829809 | XP 012545172.1 uncharacterized protein LOC101742011 isoform X1 |
| bmsk0003847 | 21.78666667 | | 4.72 | | -2.206586718 | histone acetyltransferase type B catalytic subunit [Papilio polytes] |
| bmsk0005158 | 9.8 | | 22.67333333 | | 1.210142851 | - |
| bmsk0006832 | 1.556666667 | | 5.176666667 | | 1.733563375 | scavenger receptor class B member 4 |
| bmsk0003167 | 0.773333333 | | 3.04 | | 1.974909019 | synaptic vesicle glycoprotein 2A |
| bmsk0007905 | 3.986666667 | | 8.113333333 | | 1.025111779 | protein toll-like isoform X1 |
| bmsk0001318 | 14.58 | | 1.786666667 | | -3.028648315 | uncharacterized protein LOC101741830 isoform X1 |
| bmsk0004662 | 4.703333333 | | 13.40333333 | | 1.510836348 | Glycosyl hydrolases family 18 |
| mstrg.6790 | 0.483333333 | | 1.803333333 | | 1.899575694 | PREDICTED: nose resistant to fluoxetine protein 6-like [Bombyx mori] |
| bmsk0010947 | 99.10333333 | | 28.68333333 | | -1.788720892 | ABF51484.1 SUMO-1 activating enzyme |
| bmsk0013813 | 1.106666667 | | 5.093333333 | | 2.202389397 | XP 021202015.1 uncharacterized protein LOC105841275 |
| bmsk0007479 | 1.013333333 | | 4.31 | | 2.088579046 | venom dipeptidyl peptidase 4 |
| bmsk0013576 | 0.846666667 | | 2.696666667 | | 1.671311206 | acetyl-CoA carboxylase isoform X1 |
| bmsk0003025 | 2.1 | | 4.823333333 | | 1.199641188 | uncharacterized protein LOC101741240 isoform X2 |
| mstrg.20382 | 101.7266667 | | 23.18 | | -2.133745446 | PREDICTED: probable histone-binding protein Caf1 [Bombyx mori] |
| bmsk0002541 | 1.98 | | 0.066666667 | | -4.892391026 | PAS domain-containing serine/threonine-protein kinase [Amyelois transitella] |
| bmsk0005392 | 20.77666667 | | 2.86 | | -2.86087716 | Protein tantalus [Operophtera brumata] |
| bmsk0009266 | 0.966666667 | | 2.083333333 | | 1.10780329 | uncharacterized protein LOC101742743 |
| bmsk0008770 | 51.51333333 | | 13.00333333 | | -1.9860644 | E3 SUMO-protein ligase RanBP2-like [Amyelois transitella] |
| bmsk0006480 | 39.96 | | 8.456666667 | | -2.240395564 | megator [Danaus plexippus] |
| bmsk0006873 | 0.583333333 | | 2.826666667 | | 2.276709343 | glutamate transporter isoform X1 |
| bmsk0003031 | 1.946666667 | | 7.256666667 | | 1.898301133 | epidermal retinol dehydrogenase 2 |
| bmsk0004519 | 0.001 | | 1.426666667 | | 10.47843258 | cytochrome P450, family 315, subfamily a, polypeptide 1 isoform X1 |
| bmsk0012543 | 5.75 | | 0.466666667 | | -3.62309763 | XP 021207491.1 lysine-specific demethylase 8 |
| mstrg.11868 | 2.303333333 | | 6.64 | | 1.527460032 | PREDICTED: androgen-dependent TFPI-regulating protein-like [Bombyx mori] |
| bmsk0006406 | 25.94333333 | | 3.366666667 | | -2.945971064 | CDT1 |
| bmsk0008279 | 0.001 | | 2.526666667 | | 11.30301963 | uncharacterized protein LOC101738072 isoform X3 |
| mstrg.10782 | 40.67 | | 4.536666667 | | -3.164260425 | PREDICTED: cyclin-dependent kinases regulatory subunit [Bombyx mori] |
| bmsk0001519 | 4.07 | | 0.53 | | -2.94096453 | melanoma-associated antigen G1-like [Papilio machaon] |
| bmsk0008786 | 1.403333333 | | 4.96 | | 1.821482388 | uncharacterized protein LOC106117433 [Papilio xuthus] |
| bmsk0011490 | 4.063333333 | | 10.03666667 | | 1.304544582 | - |
| bmsk0002943 | 6.94 | | 0.001 | | -12.76071995 | uncharacterized protein LOC106129803 [Amyelois transitella] |
| bmsk0002327 | 25.64666667 | | 6.766666667 | | -1.922254102 | hypothetical protein KGM 02132 [Danaus plexippus] |
| bmsk0006521 | 0.083333333 | | 4.193333333 | | 5.653060017 | - |
| bmsk0000602 | 4.806666667 | | 11.57666667 | | 1.268111246 | solute carrier family 35 member B1 homolog, partial |
| bmsk0001300 | 62.42 | | 22.25 | | -1.488203021 | PKG-Ib |
| bmsk0012222 | 256.3633333 | | 65.78 | | -1.96246902 | uncharacterized protein LOC103519968 isoform X3 [Diaphorina citri] |
| bmsk0003026 | 13.8 | | 2.756666667 | | -2.323671533 | chromatin accessibility complex protein 1 |
| bmsk0010274 | 2.076666667 | | 0.001 | | -11.02005395 | TATA-binding protein-associated factor 172 |
| bmsk0003182 | 32.38666667 | | 9.793333333 | | -1.725528094 | D replication licensing factor Mcm3 |
| bmsk0004410 | 10.58666667 | | 2.026666667 | | -2.385067684 | replication protein A1 |
| bmsk0004093 | 27.34666667 | | 4.466666667 | | -2.614094491 | uncharacterized protein LOC106135326 [Amyelois transitella] |
| bmsk0013580 | 1.396666667 | | 3.16 | | 1.177936815 | probable multidrug resistance-associated protein lethal(2)03659 |
| bmsk0009942 | 9.483333333 | | 0.001 | | -13.21117853 | uncharacterized protein LOC101740899 |
| mstrg.12765 | 3.423333333 | | 0.04 | | -6.419257966 | PREDICTED: uncharacterized protein LOC101745265 [Bombyx mori] |
| bmsk0006522 | 0.09 | | 3.626666667 | | 5.332575339 | - |
| bmsk0000510 | 0.073333333 | | 0.356666667 | | 2.282035368 | autophagy-related protein 9A |
| bmsk0009476 | 21.01 | | 5.136666667 | | -2.032171801 | diphthamide synthesis protein |
| bmsk0003853 | 0.006666667 | | 5.613333333 | | 9.717676423 | cuticular protein RR-2 motif 70 precursor |
| bmsk0005005 | 0.606666667 | | 1.643333333 | | 1.437649196 | uncharacterized protein LOC101743820 |
| bmsk0005059 | 5.276666667 | | 12.34 | | 1.22564364 | DH dehydrogenase [ubiquinone] 1 beta subcomplex subunit 2, mitochondrial |
| bmsk0003934 | 26.29 | | 8.13 | | -1.693186885 | katanin p60 ATPase-containing subunit A-like 1 |
| bmsk0005610 | 0.04 | | 0.376666667 | | 3.235216462 | guanylate cyclase 32E-like |
| bmsk0004351 | 22.75333333 | | 5.75 | | -1.984444053 | C-5 cytosine-specific D methylase |
| bmsk0016045 | 1.146666667 | | 3.706666667 | | 1.692676318 | XP 012544269.1 uncharacterized protein LOC105841366 |
| bmsk0011477 | 5.756666667 | | 13.16666667 | | 1.19358457 | tubulointerstitial nephritis antigen precursor |
| bmsk0013438 | 19.44 | | 3.69 | | -2.397335498 | XP 004930689.1 PWWP domain-containing protein 2A isoform X1 |
| bmsk0007131 | 19.77 | | 5.093333333 | | -1.956630828 | pre-mR -processing factor 40 homolog A isoform X1 |
| bmsk0004685 | 2.773333333 | | 6.783333333 | | 1.290373361 | zinc finger CCHC domain-containing protein 4 |
| bmsk0009273 | 54.62 | | 17.05666667 | | -1.679093581 | serine/threonine-protein phosphatase 4 catalytic subunit [Papilio polytes] |
| bmsk0012917 | 0.996666667 | | 2.006666667 | | 1.009618003 | elongation of very long chain fatty acids protein 7-like [Plutella xylostella] |
| bmsk0002004 | 0.876666667 | | 2.826666667 | | 1.689001465 | v-src sarcoma (Schmidt-Ruppin A-2) viral oncogene homolog isoform X2 |
| bmsk0001105 | 0.896666667 | | 1.926666667 | | 1.10346332 | uncharacterized protein LOC101746935 isoform X1 |
| bmsk0001934 | 0.556666667 | | 1.22 | | 1.131995546 | very low-density lipoprotein receptor isoform 1 precursor |
| bmsk0005579 | 0.001 | | 0.486666667 | | 8.926790153 | neural cell adhesion molecule 2-like isoform X1 |
| bmsk0015292 | 2.26 | | 7.383333333 | | 1.70794952 | actin-binding LIM protein 3 isoform X3 [Plutella xylostella] |
| bmsk0015675 | 0.666666667 | | 1.843333333 | | 1.46727948 | XP 012546497.1 uncharacterized protein LOC101746247 isoform X2 |
| bmsk0006195 | 2.84 | | 6.056666667 | | 1.092633084 | alanine aminotransferase 1 [Plutella xylostella] |
| bmsk0010507 | 2.513333333 | | 9.64 | | 1.939431124 | helix-loop-helix protein delilah-like [Amyelois transitella] |
| bmsk0000910 | 0.643333333 | | 3.236666667 | | 2.330870448 | coumarate--CoA ligase 1 |
| mstrg.5283 | 5.28 | | 11.59666667 | | 1.135100344 | olfactory receptor [Bombyx mori] |
| bmsk0006462 | 8.343333333 | | 1.316666667 | | -2.663733733 | uncharacterized protein LOC101741084 isoform X2 |
| bmsk0001336 | 0.001 | | 1.39 | | 10.44086917 | putative inorganic phosphate cotransporter isoform X3 |
| bmsk0006205 | 14.54 | | 4.14 | | -1.812324596 | serine/threonine-protein kinase SMG1 |
| bmsk0009340 | 148.3466667 | | 37.52333333 | | -1.983112612 | heterochromatin protein 1beta-like protein |
| bmsk0000230 | 0.54 | | 1.2 | | 1.152003093 | cyclic nucleotide-gated cation channel subunit A |
| bmsk0005898 | 11.46333333 | | 3.073333333 | | -1.89915046 | RING finger and WD repeat domain-containing protein 3 [Operophtera brumata] |
| bmsk0001115 | 15.68666667 | | 4.08 | | -1.942897763 | Nucleolar pre-ribosomal-associated protein 1 [Operophtera brumata] |
| mstrg.6241 | 0.096666667 | | 1.233333333 | | 3.673400465 | PREDICTED: hemicentin-1-like [Bombyx mori] |
| bmsk0010223 | 20.41666667 | | 5.323333333 | | -1.939345531 | uncharacterized protein LOC101735977 isoform X1 |
| bmsk0004647 | 25.33666667 | | 7.056666667 | | -1.844167965 | cyclin dependent kinase 12 isoform X1 |
| bmsk0005260 | 1.87 | | 0.001 | | -10.86882255 | zinc finger protein 433-like |
| bmsk0014875 | 3.486666667 | | 0.001 | | -11.76763273 | synaptotagmin-16 |
| bmsk0000809 | 11.72333333 | | 2.413333333 | | -2.280283733 | Helicase conserved C-terminal domain |
| bmsk0007164 | 15.19666667 | | 4.896666667 | | -1.633883013 | synaptojanin-1 |
| bmsk0004500 | 35.57333333 | | 9.813333333 | | -1.857980995 | cap-specific mR (nucleoside-2'-O-)-methyltransferase 1 |
| bmsk0014673 | 0.32 | | 1.143333333 | | 1.837102265 | XP 012544466.1 uncharacterized protein LOC105841398 isoform X2 |
| bmsk0009461 | 0.616666667 | | 1.973333333 | | 1.678071905 | cellular retinoic acid binding protein |
| bmsk0007743 | 0.33 | | 1.316666667 | | 1.996352223 | yellow-y isoform X1 |
| bmsk0010051 | 49.46666667 | | 13.57666667 | | -1.865327373 | - |
| bmsk0009113 | 11.45666667 | | 2.21 | | -2.374069076 | R polymerase II-associated protein 1 isoform X2 |
| bmsk0007422 | 1.486666667 | | 3.646666667 | | 1.294497123 | proton-coupled amino acid transporter 4-like |
| bmsk0007418 | 0.14 | | 1.156666667 | | 3.04647443 | elongation of very long chain fatty acids protein 4 |
| bmsk0013066 | 0.033333333 | | 1.44 | | 5.432959407 | ecdysteroid regulated protein [Danaus plexippus] |
| mstrg.9020 | 8.933333333 | | 2.336666667 | | -1.934746651 | PREDICTED: protein asunder-like [Bombyx mori] |
| mstrg.23050 | 0.576666667 | | 1.38 | | 1.25885873 | PREDICTED: protein rolling stone-like [Bombyx mori] |
| bmsk0014724 | 11.59666667 | | 2.576666667 | | -2.17013236 | XP 004924536.1 D -directed R polymerase III subunit RPC6 |
| bmsk0012253 | 4.386666667 | | 0.116666667 | | -5.232660757 | uncharacterized protein LOC106129070 [Amyelois transitella] |
| bmsk0014299 | 27.13 | | 3.263333333 | | -3.055470783 | XP 012552431.1 uncharacterized protein LOC101736466 |
| bmsk0000298 | 1.993333333 | | 0.001 | | -10.96096727 | RUS1 family protein C16orf58 homolog isoform X1 |
| bmsk0013111 | 12.44 | | 0.85 | | -3.871379834 | Uncharacterized protein OBRU01 08225 [Operophtera brumata] |
| bmsk0015405 | 0.001 | | 1.336666667 | | 10.38442402 | antibacterial peptide enbocin 3 precursor |
| bmsk0006766 | 0.443333333 | | 0.916666667 | | 1.048005373 | uncharacterized protein LOC106709759 [Papilio machaon] |
| bmsk0006458 | 0.001 | | 15.51 | | 13.92091107 | phosphoglycolate phosphatase-like [Plutella xylostella] |
| bmsk0002838 | 0.626666667 | | 1.85 | | 1.56175511 | proline-rich receptor-like protein kinase PERK12 |
| bmsk0001980 | 0.001 | | 0.316666667 | | 8.306821202 | collagen alpha-2(IV) chain |
| bmsk0009481 | 1.453333333 | | 0.001 | | -10.50514992 | CDP-diacylglycerol--inositol 3-phosphatidyltransferase |
| bmsk0015083 | 5.98 | | 0.96 | | -2.639039173 | myb-like protein X isoform X2 |
| bmsk0000917 | 0.31 | | 0.646666667 | | 1.060754031 | nonclathrin coat protein zeta 1-COP isoform X1 |
| bmsk0012550 | 0.823333333 | | 1.78 | | 1.1123287 | uncharacterized protein LOC101744721 isoform X2 |
| bmsk0006537 | 0.103333333 | | 0.383333333 | | 1.891293741 | cuticular protein hypothetical 12 precursor |
| bmsk0006647 | 40.57 | | 12.38333333 | | -1.712013593 | mediator of R polymerase II transcription subunit 10 [Papilio polytes] |
| bmsk0004945 | 8.74 | | 1.94 | | -2.171576627 | protein timeless homolog isoform X1 |
| bmsk0008948 | 0.001 | | 2.48 | | 11.27612441 | Haemolymph juvenile hormone binding protein (JHBP) |
| bmsk0005644 | 4.54 | | 0.873333333 | | -2.378087986 | D polymerase delta catalytic subunit |
| bmsk0005645 | 4.54 | | 0.873333333 | | -2.378087986 | - |
| bmsk0008110 | 0.223333333 | | 0.546666667 | | 1.291462814 | transient receptor potential protein isoform X1 |
| bmsk0005652 | 21.34666667 | | 5.626666667 | | -1.923658404 | caspase-1 |
| bmsk0003874 | 1.663333333 | | 0.001 | | -10.6998616 | chitinase precursor |
| bmsk0015783 | 0.27 | | 0.823333333 | | 1.608517229 | XP 021208504.1 vacuolar ATPase subunit C isoform X1 |
| bmsk0006908 | 25.69666667 | | 5.89 | | -2.125241688 | protein KBP homolog |
| bmsk0011313 | 3.32 | | 10.06333333 | | 1.599853109 | - |
| bmsk0010013 | 0.46 | | 1.143333333 | | 1.313540309 | Voltage-dependent calcium channel gamma-5 subunit [Papilio machaon] |
| bmsk0006407 | 76.73666667 | | 23.97 | | -1.678686189 | SRSF protein kinase 2 [Papilio machaon] |
| bmsk0005976 | 0.001 | | 2.42 | | 11.24079133 | gelsolin |
| bmsk0001421 | 28.24333333 | | 7.176666667 | | -1.97652455 | pre-mR -splicing regulator female-lethal(2)D [Amyelois transitella] |
| bmsk0013083 | 32.18333333 | | 6.943333333 | | -2.21261342 | protein-methionine sulfoxide oxidase mical3a-like isoform X1 |
| bmsk0016032 | 0.536666667 | | 1.833333333 | | 1.77237093 | XP 012544278.1 endoplasmic reticulum metallopeptidase 1 isoform X2 |
| bmsk0014643 | 0.453333333 | | 0.001 | | -8.824428435 | D polymerase epsilon catalytic subunit A [Amyelois transitella] |
| bmsk0006433 | 7.523333333 | | 1.716666667 | | -2.131762081 | probable ATP-dependent R helicase DDX20 |
| bmsk0003649 | 0.946666667 | | 2.326666667 | | 1.297336107 | - |
| bmsk0007323 | 1.1 | | 0.001 | | -10.10328781 | uncharacterized protein LOC106132264 [Amyelois transitella] |
| bmsk0006194 | 0.046666667 | | 0.536666667 | | 3.523561956 | Alanine aminotransferase 1 [Papilio xuthus] |
| bmsk0007619 | 4.626666667 | | 0.593333333 | | -2.963058422 | anaphase-promoting complex subunit 4 [Papilio xuthus] |
| bmsk0012279 | 356.2066667 | | 116.3233333 | | -1.614574001 | aliphatic nitrilase |
| bmsk0010605 | 24.79666667 | | 5.416666667 | | -2.19466898 | uncharacterized protein LOC100174822 |
| bmsk0001946 | 30.35333333 | | 8.756666667 | | -1.793401259 | WD40 protein |
| bmsk0002756 | 0.17 | | 0.466666667 | | 1.456857675 | cilia- and flagella-associated protein 57 isoform X2 [Amyelois transitella] |
| bmsk0001487 | 12.05 | | 2.803333333 | | -2.103817942 | Uncharacterized protein OBRU01 21543 [Operophtera brumata] |
| bmsk0003698 | 0.37 | | 0.763333333 | | 1.044787922 | transient receptor potential channel pyrexia, partial |
| bmsk0013154 | 28.6 | | 7.52 | | -1.92721058 | XP 004921720.1 THO complex subunit 7 homolog isoform X1 |
| bmsk0006352 | 0.793333333 | | 0.001 | | -9.631783357 | C-type lectin domain family 4 member F |
| bmsk0015614 | 0.586666667 | | 3.873333333 | | 2.722962735 | hexokinase-2-like [Amyelois transitella] |
| bmsk0001860 | 32.93 | | 7.713333333 | | -2.093976148 | acinus |
| bmsk0013617 | 0.056666667 | | 0.78 | | 3.782901878 | uncharacterized protein LOC101741500 |
| bmsk0011956 | 0.003333333 | | 0.433333333 | | 7.022367813 | collagen and calcium-binding EGF domain-containing protein 1-like [Papilio machaon] |
| bmsk0010179 | 0.463333333 | | 0.001 | | -8.855906667 | serine/threonine-protein kinase N |
| bmsk0007442 | 5.38 | | 1.23 | | -2.128947857 | GDP-fucose transporter 1 |
| bmsk0008398 | 0.266666667 | | 1.01 | | 1.921245889 | protein vav isoform X1 |
| mstrg.13448 | 14.44 | | 3.073333333 | | -2.232194587 | PREDICTED: chromatin assembly factor 1 subunit A [Bombyx mori] |
| bmsk0002156 | 0.996666667 | | 0.093333333 | | -3.416646752 | ATPase WRNIP1 |
| bmsk0009618 | 1.456666667 | | 0.001 | | -10.50845506 | protein lethal(2)k10201-like [Plutella xylostella] |
| bmsk0007714 | 0.18 | | 0.613333333 | | 1.768674454 | cAMP-dependent protein kinase catalytic subunit-like |
| bmsk0009246 | 1.273333333 | | 0.106666667 | | -3.577428828 | bleomycin hydrolase isoform X1 [Amyelois transitella] |
| bmsk0001937 | 1.85 | | 3.953333333 | | 1.095544333 | E3 ubiquitin-protein ligase Su(dx) |
| bmsk0003803 | 15.27 | | 4.143333333 | | -1.881836266 | mediator of R polymerase II transcription subunit 24 |
| bmsk0009188 | 4.193333333 | | 0.036666667 | | -6.837484588 | - |
| bmsk0013372 | 0.506666667 | | 1.793333333 | | 1.823534849 | hypothetical protein RR48 13994 [Papilio machaon] |
| bmsk0005470 | 1.32 | | 0.13 | | -3.343954401 | BTB/POZ domain-containing adapter for CUL3-mediated RhoA degradation protein 3 |
| bmsk0004171 | 0.02 | | 0.606666667 | | 4.922832139 | putative inorganic phosphate cotransporter |
| bmsk0006099 | 11.94666667 | | 3.356666667 | | -1.831506954 | zinc finger protein 600-like [Amyelois transitella] |
| bmsk0003933 | 0.001 | | 1.5 | | 10.55074679 | - |
| bmsk0003555 | 0.113333333 | | 0.266666667 | | 1.234465254 | - |
| bmsk0008814 | 2.76 | | 0.353333333 | | -2.965566503 | coiled-coil domain-containing protein 39 [Amyelois transitella] |
| bmsk0008035 | 0.193333333 | | 0.533333333 | | 1.4639471 | roundabout homolog 3 |
| bmsk0010252 | 6.113333333 | | 19.13666667 | | 1.646308417 | Aconitase family (aconitate hydratase) |
| bmsk0012967 | 0.35 | | 0.923333333 | | 1.399496648 | XP 004924293.1 arrestin homolog |
| bmsk0015988 | 25.72333333 | | 6.946666667 | | -1.888684828 | XP 012544259.1 MIP18 family protein CG30152 |
| bmsk0000060 | 26.12 | | 8.09 | | -1.690943289 | transcriptional repressor CTCF |
| bmsk0014822 | 0.546666667 | | 1.536666667 | | 1.491070936 | XP 012545358.1 nephrin isoform X1 |
| bmsk0005823 | 24.11 | | 5.143333333 | | -2.22885609 | B-cell CLL/lymphoma 7 protein family member A |
| bmsk0005118 | 0.556666667 | | 0.001 | | -9.120669887 | STAM-binding protein-like A |
| bmsk0013564 | 14.04333333 | | 3.773333333 | | -1.895973958 | putative tR pseudouridine synthase Pus10 [Papilio polytes] |
| bmsk0014415 | 0.001 | | 0.433333333 | | 8.759333407 | putative uncharacterized protein DDB G0282133 [Amyelois transitella] |
| bmsk0015116 | 12.49333333 | | 3.346666667 | | -1.900361684 | XP 004933026.1 zinc finger protein on ecdysone puffs isoform X1 |
| bmsk0007531 | 18.42666667 | | 4.436666667 | | -2.054247044 | coiled-coil domain-containing protein 94 [Amyelois transitella] |
| bmsk0008031 | 3.713333333 | | 0.001 | | -11.85849911 | cytochrome P450 CYP339A1 |
| bmsk0001813 | 17.32666667 | | 4.223333333 | | -2.036540109 | D primase large subunit |
| bmsk0005846 | 7.826666667 | | 1.016666667 | | -2.944551261 | regulator of telomere elongation helicase 1 homolog isoform X2 |
| bmsk0014529 | 13.72 | | 4.246666667 | | -1.691877705 | XP 012545467.2 patj homolog |
| bmsk0010545 | 35.45 | | 11.64666667 | | -1.60586852 | XP 004928198.1 nuclear R export factor 1 |
| mstrg.25461 | 51.77666667 | | 12.42333333 | | -2.059249771 | geminin [Bombyx mori] |
| bmsk0004922 | 27.60666667 | | 6.08 | | -2.182873473 | dnaJ (Hsp40) homolog 10 isoform X1 |
| bmsk0014400 | 17.00333333 | | 4.973333333 | | -1.773532565 | RSM22-like protein, mitochondrial [Operophtera brumata] |
| bmsk0006639 | 1.283333333 | | 0.001 | | -10.32568023 | trimethylguanosine synthase |
| bmsk0002405 | 10.11 | | 2.63 | | -1.942648293 | transcriptional adapter 2A |
| bmsk0004103 | 12.54666667 | | 3.483333333 | | -1.848763686 | cyclin-H [Amyelois transitella] |
| bmsk0008366 | 6.42 | | 1.906666667 | | -1.751520651 | R -binding protein 45 |
| bmsk0007865 | 0.001 | | 0.096666667 | | 6.594946589 | tyrosine-protein phosphatase non-receptor type 11-like |
| bmsk0002438 | 22.42333333 | | 6.463333333 | | -1.794650455 | diptheria toxin resistance protein |
| bmsk0003535 | 10.3 | | 2.51 | | -2.036885068 | extra sex combs |
| bmsk0005684 | 14.70333333 | | 2.966666667 | | -2.309228519 | ABD36106.1 E2F transcription factor 4-like protein |
| mstrg.1677 | 25.64666667 | | 6.84 | | -1.906703099 | PREDICTED: DNA primase small subunit [Bombyx mori] |
| mstrg.8252 | 21.23666667 | | 7.293333333 | | -1.541907099 | PREDICTED: PAX-interacting protein 1 [Bombyx mori] |
| bmsk0013319 | 20.06333333 | | 4.843333333 | | -2.050489114 | Replication factor C 38kD subunit [Operophtera brumata] |
| bmsk0000671 | 11.36333333 | | 3.69 | | -1.622693377 | Y+L amino acid transporter 2 |
| bmsk0000002 | 0.001 | | 0.25 | | 7.965784285 | anion exchange protein 3 isoform X1 |
| bmsk0008201 | 0.41 | | 2.703333333 | | 2.721043599 | - |
| bmsk0015160 | 0.01 | | 0.85 | | 6.409390936 | lysyl oxidase homolog 3 [Papilio polytes] |
| bmsk0008585 | 5.203333333 | | 0.36 | | -3.85336732 | polycomb protein Pcl isoform X3 |
| bmsk0002269 | 0.14 | | 0.993333333 | | 2.826851098 | cuticular protein RR-2 motif 60 precursor |
| bmsk0002647 | 0.083333333 | | 0.276666667 | | 1.731183242 | myosin-2 heavy chain |
| bmsk0010293 | 1.236666667 | | 0.26 | | -2.249873158 | sulfotransferase |
| bmsk0014260 | 5.286666667 | | 1.673333333 | | -1.659633502 | uncharacterized protein LOC101737620 isoform X3 |
| bmsk0005006 | 24.26333333 | | 7.06 | | -1.781037675 | cell division cycle and apoptosis regulator protein 1 |
| bmsk0011828 | 2.27 | | 0.203333333 | | -3.48077365 | XP 004926159.1 KH domain-containing, R -binding, signal transduction-associated protein 3 isoform X1 |
| mstrg.10249 | 3.086666667 | | 0.596666667 | | -2.371052606 | PREDICTED: proline-rich protein 11-like [Amyelois transitella] |
| bmsk0008808 | 0.436666667 | | 0.973333333 | | 1.156401557 | oxygen resistance gene 1 isoform X6 |
| bmsk0003797 | 0.026666667 | | 0.403333333 | | 3.918863237 | uncharacterized protein LOC106707703 isoform X1 [Papilio machaon] |
| bmsk0005543 | 3.463333333 | | 0.406666667 | | -3.090242601 | tectonin beta-propeller repeat-containing protein |
| bmsk0005091 | 0.263333333 | | 1.293333333 | | 2.296132094 | proton-associated sugar transporter A-like [Papilio polytes] |
| bmsk0007122 | 11.41 | | 3.31 | | -1.785395669 | rab3 GTPase-activating protein regulatory subunit |
| bmsk0007649 | 18.62333333 | | 7.196666667 | | -1.371710577 | THO complex subunit 2 isoform X1 |
| bmsk0012088 | 0.606666667 | | 0.001 | | -9.244760234 | XP 021205925.1 bumetanide-sensitive sodium-(potassium)-chloride cotransporter isoform X2 |
| bmsk0005534 | 7.453333333 | | 1.873333333 | | -1.992278153 | telomerase-binding protein EST1A, partial |
| bmsk0011878 | 6.683333333 | | 1.46 | | -2.194599462 | ADD10141.1 origin recognition complex subunit 5 |
| bmsk0008511 | 2.01 | | 0.206666667 | | -3.281817882 | GTPase-activating Rap/Ran-GAP domain-like protein 3 isoform X3 |
| bmsk0010405 | 0.001 | | 1.166666667 | | 10.18817671 | cuticular protein glycine-rich 26 precursor |
| bmsk0003085 | 0.001 | | 0.613333333 | | 9.26052755 | synaptic vesicle glycoprotein 2A |
| bmsk0014664 | 7.08 | | 1.636666667 | | -2.112988836 | XP 004923342.1 tubulin--tyrosine ligase-like protein 12 |
| bmsk0010072 | 20.09666667 | | 6.773333333 | | -1.569018327 | putative dual specificity mitogen-activated protein kinase kinase 4 MAPKK4 [Danaus plexippus] |
| bmsk0008264 | 0.001 | | 0.58 | | 9.17990909 | Uncharacterized protein OBRU01 19226 [Operophtera brumata] |
| bmsk0006838 | 7.596666667 | | 1.43 | | -2.409351372 | sprT-like domain-containing protein Spartan [Papilio machaon] |
| bmsk0007054 | 1.423333333 | | 0.001 | | -10.47505785 | breast cancer type 1 susceptibility protein homolog |
| bmsk0010421 | 12.3 | | 4.266666667 | | -1.527477006 | XP 004925946.1 protein Smaug |
| bmsk0013833 | 1.863333333 | | 3.753333333 | | 1.010286639 | XP 021202030.1 pleckstrin homology-like domain family B member 1 |
| bmsk0012742 | 0.426666667 | | 0.953333333 | | 1.159871337 | uncharacterized protein LOC105391048 [Plutella xylostella] |
| bmsk0001298 | 0.603333333 | | 0.001 | | -9.236811481 | THAP domain |
| bmsk0008887 | 40.18333333 | | 13.01666667 | | -1.626237197 | minichromosome maintenance complex component 7 |
| bmsk0010815 | 70.72666667 | | 24.91333333 | | -1.505336206 | nuclear valosin-containing protein-like [Amyelois transitella] |
| bmsk0007006 | 28.65333333 | | 7.313333333 | | -1.970101957 | kelch domain-containing protein 3 [Papilio machaon] |
| bmsk0007688 | 44.09 | | 14.3 | | -1.62443633 | apoptosis inhibitor 5 |
| bmsk0006339 | 2.513333333 | | 5.126666667 | | 1.028419075 | protein cueball |
| bmsk0001495 | 17.39333333 | | 35.50666667 | | 1.029555484 | XP 004924437.1 glyoxylate reductase/hydroxypyruvate reductase |
| bmsk0002288 | 5.333333333 | | 0.52 | | -3.358453971 | Microtubule-binding calmodulin-regulated spectrin-associated |
| bmsk0010064 | 8.953333333 | | 3.053333333 | | -1.552039801 | Ubiquitin carboxyl-terminal hydrolase |
| bmsk0011903 | 8.083333333 | | 1.54 | | -2.392019991 | ubiquitin-conjugating enzyme E2 T-like [Papilio polytes] |
| bmsk0007321 | 7.62 | | 1.333333333 | | -2.514753498 | D replication complex GINS protein PSF1-like [Plutella xylostella] |
| bmsk0012998 | 2.903333333 | | 0.001 | | -11.5034945 | histone H2AX-like protein |
| bmsk0009303 | 17.43 | | 5.81 | | -1.584962501 | hypothetical protein KGM 15598 [Danaus plexippus] |
| bmsk0014934 | 11.98666667 | | 3.253333333 | | -1.881439968 | protein UXT homolog [Amyelois transitella] |
| bmsk0004455 | 42.47666667 | | 15.65666667 | | -1.439893463 | AP-2 complex subunit alpha |
| bmsk0001406 | 12.16 | | 2.713333333 | | -2.16400503 | peptidyl-prolyl cis-trans isomerase-like 4 [Papilio machaon] |
| bmsk0008073 | 0.326666667 | | 1.516666667 | | 2.215012891 | stromal interaction molecule 1 precursor |
| bmsk0000118 | 7.67 | | 2.236666667 | | -1.777876312 | serine/threonine-protein kinase prp4 isoform X1 [Papilio machaon] |
| bmsk0013466 | 21.2 | | 5.386666667 | | -1.976599567 | XP 004930707.1 THAP domain-containing protein 1 |
| bmsk0006085 | 0.001 | | 0.333333333 | | 8.380821784 | carboxylesterase 5A-like |
| bmsk0012893 | 3.403333333 | | 0.89 | | -1.935071219 | exocyst complex component 4 |
| bmsk0013101 | 0.001 | | 0.37 | | 8.531381461 | aminoacylase |
| bmsk0006436 | 16.52 | | 5.08 | | -1.701313285 | protein disulfide-isomerase TMX3 [Amyelois transitella] |
| bmsk0010596 | 3.99 | | 1.336666667 | | -1.57774901 | uncharacterized protein LOC101744659 isoform X1 |
| bmsk0016013 | 0.123333333 | | 0.536666667 | | 2.121463512 | UDP-glucoronosyl and UDP-glucosyl transferase |
| bmsk0010613 | 0.35 | | 0.003333333 | | -6.714245518 | XP 004928406.1 inositol-tetrakisphosphate 1-kinase |
| mstrg.1644 | 21.51 | | 6.793333333 | | -1.662815974 | PREDICTED: methylosome protein 50-like [Bombyx mori] |
| bmsk0008783 | 29.57333333 | | 9.316666667 | | -1.666411082 | transcription factor-like protein |
| bmsk0012685 | 5.686666667 | | 1.54 | | -1.88465289 | XP 004931917.1 GTP-binding protein Di-Ras2 |
| bmsk0011937 | 26.19666667 | | 9.093333333 | | -1.526502107 | XP 004927331.1 SOSS complex subunit B homolog |
| bmsk0014681 | 5.52 | | 1.883333333 | | -1.5513799 | XP 004924600.1 uncharacterized protein LOC101746938 |
| bmsk0009334 | 5.746666667 | | 2.043333333 | | -1.491800795 | histone-lysine N-methyltransferase SETD1 isoform X1 |
| bmsk0014925 | 1.46 | | 0.001 | | -10.51175265 | potassium channel subfamily K member 9 [Amyelois transitella] |
| bmsk0015252 | 36.97333333 | | 10.23333333 | | -1.853208961 | methenyltetrahydrofolate synthase domain-containing protein [Amyelois transitella] |
| bmsk0015842 | 2.21 | | 0.556666667 | | -1.989160768 | uncharacterized protein LOC101735769 |
| bmsk0015014 | 0.001 | | 0.486666667 | | 8.926790153 | CD80-like C2-set immunoglobulin domain |
| bmsk0004644 | 3.98 | | 1.2 | | -1.729734025 | phosphopantothenoylcysteine decarboxylase [Papilio polytes] |
| bmsk0005348 | 0.001 | | 0.156666667 | | 7.291554446 | integrin alpha-PS4-like |
| bmsk0002845 | 8.026666667 | | 1.836666667 | | -2.127711168 | uncharacterized protein LOC110386756 |
| bmsk0002176 | 2.063333333 | | 0.433333333 | | -2.251427786 | mitoguardin |
| bmsk0004577 | 45.15333333 | | 15.38666667 | | -1.553151772 | FACT complex subunit Ssrp1 [Amyelois transitella] |
| bmsk0005578 | 27.48333333 | | 8.806666667 | | -1.641889027 | uncharacterized protein LOC106131814 [Amyelois transitella] |
| bmsk0003628 | 3.22 | | 0.001 | | -11.65284497 | collagen alpha chain CG42342 |
| bmsk0010119 | 0.77 | | 0.001 | | -9.588714636 | serine protease easter-like |
| bmsk0004368 | 147.1833333 | | 54.29333333 | | -1.438767348 | ubiquitin-conjugating enzyme E2 isoform 1 |
| bmsk0001305 | 2.606666667 | | 0.686666667 | | -1.92452427 | rap1 GTPase-activating protein 1 isoform X2 |
| bmsk0002346 | 0.143333333 | | 0.556666667 | | 1.957439538 | ARRH HELVI RecName= Full=Arrestin homolog |
| bmsk0009117 | 1.976666667 | | 0.001 | | -10.94885389 | Domain of unknown function (DUF1899) |
| bmsk0002018 | 31.64333333 | | 9.413333333 | | -1.749123995 | mediator of R polymerase II transcription subunit 11 |
| bmsk0002447 | 94.33666667 | | 31.35666667 | | -1.589046421 | ribonucleoside diphosphate reductase small subunit |
| mstrg.14230 | 58.57666667 | | 16.99 | | -1.785640246 | PREDICTED: chromatin modification-related protein MEAF6 [Amyelois transitella] |
| bmsk0000541 | 0.33 | | 0.001 | | -8.366322214 | ubiquitin-conjugating enzyme E2 G2 |
| bmsk0001686 | 14.43333333 | | 4.91 | | -1.555609595 | pre-mR -splicing factor CWC22 homolog |
| bmsk0009721 | 3.59 | | 0.67 | | -2.421750843 | 7,8-dihydro-8-oxoguanine triphosphatase-like |
| bmsk0001053 | 6.533333333 | | 0.883333333 | | -2.88678939 | ADM32512.1 atf2 |
| bmsk0003341 | 1.073333333 | | 0.001 | | -10.06788247 | abdominal-A transcript variant E |
| bmsk0011414 | 29.98 | | 9.946666667 | | -1.591715348 | ACP43577.1 cyclin |
| bmsk0014145 | 1.05 | | 0.226666667 | | -2.211745177 | - |
| mstrg.13596 | 6.923333333 | | 1.953333333 | | -1.825528646 | PREDICTED: protein prenyltransferase alpha subunit repeat-containing protein 1 [Bombyx mori] |
| bmsk0011712 | 2.303333333 | | 0.58 | | -1.989598405 | XP 012546765.1 transmembrane protein 68 isoform X1 |
| bmsk0010177 | 7.476666667 | | 2.156666667 | | -1.793592003 | endochitinase A isoform X1 [Papilio xuthus] |
| bmsk0001471 | 5.326666667 | | 1.846666667 | | -1.528309527 | monocyte to macrophage differentiation factor isoform X2 |
| bmsk0007242 | 47.98333333 | | 12.57666667 | | -1.931783784 | protein dpy-30 homolog |
| bmsk0005604 | 0.09 | | 0.33 | | 1.874469118 | tyrosine decarboxylase [Amyelois transitella] |
| bmsk0010347 | 8.756666667 | | 1.866666667 | | -2.229917468 | XP 004926405.1 transcription factor Adf-1 |
| bmsk0014571 | 3.18 | | 0.426666667 | | -2.897845456 | XP 012553118.2 putative leucine-rich repeat-containing protein DDB G0290503 |
| bmsk0001792 | 0.001 | | 0.266666667 | | 8.058893689 | integrin beta3 |
| bmsk0004926 | 9.943333333 | | 2.67 | | -1.89688983 | F-box only protein 28 isoform X1 |
| bmsk0001846 | 15.08666667 | | 4.706666667 | | -1.680496496 | transcription elongation factor S-II |
| bmsk0014119 | 0.36 | | 1.11 | | 1.624490865 | XP 004926342.1 uncharacterized protein LOC101745081 isoform X2 |
| bmsk0008359 | 2.26 | | 0.403333333 | | -2.486278226 | uncharacterized protein LOC101738399 isoform X3 |
| bmsk0003132 | 0.133333333 | | 1.433333333 | | 3.426264755 | - |
| bmsk0003412 | 40.11 | | 14.48 | | -1.469900364 | splicing factor 3A subunit 3 |
| bmsk0005218 | 0.001 | | 0.326666667 | | 8.351675438 | phospholipase B1, membrane-associated-like |
| bmsk0004463 | 8.816666667 | | 2.3 | | -1.938599455 | protein FAM117B-like isoform X2 [Papilio machaon] |
| bmsk0012891 | 7.956666667 | | 2.51 | | -1.664476796 | rap1 GTPase-GDP dissociation stimulator 1-B [Amyelois transitella] |
| bmsk0000275 | 13.97666667 | | 3.923333333 | | -1.83286851 | geminin isoform X2 [Amyelois transitella] |
| bmsk0010529 | 0.173333333 | | 0.91 | | 2.392317423 | UDP-glycosyltransferase UGT50A1 precursor |
| bmsk0010323 | 3.893333333 | | 0.77 | | -2.338075517 | zinc finger protein 267-like isoform X1 |
| bmsk0004857 | 18.70666667 | | 5.56 | | -1.75039572 | PCI domain-containing protein 2 homolog |
| bmsk0011480 | 20.86666667 | | 6.773333333 | | -1.623262255 | partitioning defective protein 6 [Papilio polytes] |
| bmsk0003849 | 0.063333333 | | 0.693333333 | | 3.452512205 | cuticular protein RR-2 motif 67 precursor |
| mstrg.16172 | 3.85 | | 1.08 | | -1.833827133 | PREDICTED: gastrula zinc finger protein XlCGF46.1-like, partial [Bombyx mori] |
| bmsk0004254 | 20.81 | | 7.553333333 | | -1.462091605 | SUZ domain-containing protein 1 |
| bmsk0002762 | 6.706666667 | | 1.46 | | -2.19962753 | CD2 antigen cytoplasmic tail-binding protein 2 homolog |
| bmsk0006064 | 8.04 | | 2.4 | | -1.744161096 | hypothetical protein KGM 01363 [Danaus plexippus] |
| bmsk0005493 | 16.36 | | 4.35 | | -1.911085442 | tetratricopeptide repeat protein 37 [Papilio xuthus] |
| bmsk0007483 | 7.84 | | 1.783333333 | | -2.136277264 | TBC domain-containing protein kinase-like protein |
| bmsk0000075 | 14.67666667 | | 3.553333333 | | -2.046279406 | Bmptp-Z and Bmap-A fusion protein gamma |
| bmsk0006564 | 13.83 | | 4.34 | | -1.672034209 | uncharacterized protein LOC101745002 |
| bmsk0005008 | 24.46666667 | | 8.91 | | -1.457320225 | striatin-interacting protein 1 [Papilio polytes] |
| bmsk0012201 | 25.28 | | 6.273333333 | | -2.010692336 | cyclin dependent kinase 7 |
| bmsk0004534 | 29.43666667 | | 10.79333333 | | -1.447473829 | sarcolemmal membrane-associated protein [Papilio machaon] |
| bmsk0009323 | 19.29666667 | | 5.376666667 | | -1.843567718 | synaptosomal-associated protein 29 [Papilio polytes] |
| bmsk0012221 | 1.07 | | 0.25 | | -2.097610797 | XP 004929694.1 endophilin-A isoform X2 |
| bmsk0010009 | 0.001 | | 0.136666667 | | 7.094517599 | uncharacterized protein LOC106119184 [Papilio xuthus] |
| bmsk0012516 | 2.803333333 | | 0.42 | | -2.738682067 | XP 012550828.1 ATP-binding cassette sub-family G member 8 |
| bmsk0008790 | 0.126666667 | | 0.486666667 | | 1.941897045 | uncharacterized protein LOC101735403 |
| bmsk0001367 | 1.34 | | 4.63 | | 1.788779193 | protein split ends |
| bmsk0001042 | 0.001 | | 0.236666667 | | 7.886712714 | aldo-keto reductase AKR2E4-like |
| bmsk0012632 | 11.24 | | 2.413333333 | | -2.219542934 | gonadal protein gdl [Papilio polytes] |
| bmsk0015832 | 0.226666667 | | 0.001 | | -7.824428435 | putative ferric-chelate reductase 1 homolog [Amyelois transitella] |
| bmsk0002924 | 50.67 | | 17.41 | | -1.541215626 | ADP-ribosylation factor 6 |
| bmsk0015804 | 10.21333333 | | 2.586666667 | | -1.98128774 | XP 021208540.1 TIMELESS-interacting protein |
| bmsk0011908 | 0.001 | | 0.196666667 | | 7.619608644 | uncharacterized protein LOC106102885 precursor [Papilio polytes] |
| bmsk0015119 | 0.001 | | 0.466666667 | | 8.866248611 | facilitated trehalose transporter Tret1-like |
| bmsk0012091 | 1.7 | | 0.04 | | -5.409390936 | - |
| bmsk0011936 | 0.886666667 | | 1.986666667 | | 1.163886085 | XP 004927327.1 DH dehydrogenase [ubiquinone] 1 beta subcomplex subunit 3 |
| bmsk0006061 | 0.44 | | 0.001 | | -8.781359714 | Peptidase M1 N-terminal domain |
| bmsk0015467 | 15.59333333 | | 4.453333333 | | -1.807971854 | XP 004926638.1 UPF0428 protein CXorf56 homolog |
| bmsk0008018 | 1.496666667 | | 14.18666667 | | 3.244710801 | - |
| bmsk0015085 | 9.98 | | 2.906666667 | | -1.779674181 | XP 012551699.1 biorientation of chromosomes in cell division protein 1-like 1 isoform X1 |
| bmsk0000650 | 0.503333333 | | 1.193333333 | | 1.245411038 | Domain of unknown function (DUF4780) |
| mstrg.8172 | 10.31333333 | | 2.98 | | -1.79112646 | hypothetical protein g.7559 [Pectinophora gossypiella] |
| bmsk0005912 | 2.753333333 | | 0.616666667 | | -2.158616511 | zinc finger protein 28-like [Amyelois transitella] |
| bmsk0000879 | 0.033333333 | | 0.433333333 | | 3.700439718 | - |
| bmsk0013237 | 27.36666667 | | 6.493333333 | | -2.075388545 | nuclear receptor-binding factor 2-like [Papilio polytes] |
| bmsk0014853 | 21.67 | | 6.706666667 | | -1.692031349 | XP 021202871.1 D replication licensing factor Mcm5 |
| bmsk0013010 | 25.86666667 | | 6.75 | | -1.938134744 | Prefoldin subunit 2 [Operophtera brumata] |
| bmsk0002815 | 28.22 | | 10.17333333 | | -1.471925526 | argonaute 1 |
| bmsk0011039 | 0.2 | | 0.001 | | -7.64385619 | XP 021208414.1 protein disabled |
| bmsk0002350 | 0.31 | | 0.866666667 | | 1.483209002 | uncharacterized protein LOC101741863 |
| bmsk0014880 | 13.02 | | 4.513333333 | | -1.52846421 | BTB/POZ domain containing protein |
| bmsk0007182 | 34.04 | | 11.15333333 | | -1.609756092 | R polymerase II transcriptional coactivator [Papilio machaon] |
| bmsk0013168 | 14.02666667 | | 5.27 | | -1.412297337 | Retrovirus-related Pol polyprotein from transposon TNT 1-94 [Papilio machaon] |
| bmsk0011896 | 0.136666667 | | 0.99 | | 2.856767116 | - |
| mstrg.13277 | 0.193333333 | | 0.72 | | 1.896906507 | PREDICTED: uncharacterized protein LOC101743025 [Bombyx mori] |
| bmsk0008619 | 0.063333333 | | 0.65 | | 3.3594028 | uncharacterized protein LOC101738615 isoform X1 |
| bmsk0012319 | 0.016666667 | | 0.193333333 | | 3.5360529 | Baculovirus FP protein |
| bmsk0008503 | 4.32 | | 1.273333333 | | -1.762421175 | death-associated small cytoplasmic leucine-rich protein isoform X2 |
| bmsk0012785 | 0.23 | | 0.496666667 | | 1.110644064 | hypothetical protein KGM 01235 [Danaus plexippus] |
| bmsk0012065 | 0.01 | | 0.663333333 | | 6.05166212 | cuticular protein RR-2 motif 138 precursor |
| bmsk0011925 | 0.033333333 | | 0.176666667 | | 2.40599236 | uncharacterized protein LOC106102902 [Papilio polytes] |
| bmsk0012084 | 0.943333333 | | 0.001 | | -9.881623837 | 2-oxoglutarate and iron-dependent oxygenase domain-containing protein 3-like [Papilio polytes] |
| bmsk0007660 | 11.77666667 | | 3.706666667 | | -1.667736961 | uncharacterized protein LOC101739083 isoform X1 |
| mstrg.24764 | 0.5 | | 1.103333333 | | 1.141868716 | PREDICTED: uncharacterized protein LOC101744165 [Bombyx mori] |
| bmsk0009351 | 0.001 | | 0.04 | | 5.321928095 | small G protein signaling modulator 2-like isoform X4 [Papilio polytes] |
| bmsk0001358 | 25.60333333 | | 9.403333333 | | -1.445087484 | pre-mR 3' end processing protein WDR33-like [Papilio xuthus] |
| bmsk0005626 | 0.24 | | 0.956666667 | | 1.994981925 | - |
| bmsk0006994 | 2.186666667 | | 0.386666667 | | -2.499571009 | phosphatidylinositol 3-kinase 60 |
| bmsk0011452 | 3.76 | | 1.036666667 | | -1.858780582 | XP 021204634.1 facilitated trehalose transporter Tret1-2 homolog |
| bmsk0006084 | 1.18 | | 0.09 | | -3.712718048 | cholinesterase 2 |
| bmsk0001887 | 120.67 | | 50.44 | | -1.258426869 | DnaJ-9 |
| bmsk0012004 | 1.903333333 | | 0.053333333 | | -5.157346935 | - |
| bmsk0008668 | 0.04 | | 0.143333333 | | 1.841302254 | uncharacterized protein LOC105842023 |
| bmsk0007126 | 23.87666667 | | 7.11 | | -1.747679976 | ribonucleoside-diphosphate reductase large subunit isoform X1 [Amyelois transitella] |
| bmsk0007984 | 99.75333333 | | 39.2 | | -1.347511397 | host cell factor 1 |
| bmsk0007656 | 0.013333333 | | 0.543333333 | | 5.348728154 | bestrophin-4 isoform X2 |
| bmsk0001732 | 5.553333333 | | 1.563333333 | | -1.828728573 | TBC1 domain family member 24 isoform X1 |
| bmsk0009914 | 0.01 | | 0.356666667 | | 5.156504486 | SOCS box |
| bmsk0010094 | 1.603333333 | | 0.153333333 | | -3.386331128 | putative homeodomain transcription factor, partial |
| bmsk0011796 | 1.026666667 | | 0.001 | | -10.00375213 | XP 004927290.1 uncharacterized protein LOC101742687 isoform X1 |
| bmsk0010352 | 9.653333333 | | 2.603333333 | | -1.890667149 | uncharacterized protein LOC101745082 isoform X2 |
| bmsk0012650 | 0.136666667 | | 0.336666667 | | 1.300659478 | XP 012550801.1 uncharacterized protein LOC101742417 |
| bmsk0006475 | 16.50333333 | | 5.233333333 | | -1.65695539 | tether containing UBX domain for GLUT4 |
| mstrg.4132 | 25.4 | | 8.656666667 | | -1.552944984 | PREDICTED: galectin-9-like isoform X1 [Bombyx mori] |
| bmsk0012173 | 23.06666667 | | 7.99 | | -1.529542129 | microfibrillar-associated protein 1 [Amyelois transitella] |
| bmsk0001472 | 4.06 | | 1.083333333 | | -1.90600251 | BLOC-1-related complex subunit 6 isoform X1 |
| bmsk0004594 | 15.28666667 | | 4.903333333 | | -1.640439108 | replication protein A3 |
| bmsk0006617 | 33.95333333 | | 9.823333333 | | -1.789268657 | uncharacterized protein LOC100286771 isoform X1 |
| bmsk0014140 | 2.47 | | 0.383333333 | | -2.687839681 | XP 004926346.1 uncharacterized protein LOC101745670 isoform X2 |
| bmsk0007094 | 12.26333333 | | 3.26 | | -1.911407306 | coiled-coil domain-containing protein 97 [Amyelois transitella] |
| bmsk0013610 | 4.523333333 | | 0.636666667 | | -2.828776177 | XP 004930850.3 uncharacterized protein LOC101741081 |
| bmsk0010960 | 14.91666667 | | 4.62 | | -1.690960425 | putative Lancl1 protein [Danaus plexippus] |
| bmsk0001482 | 0.056666667 | | 0.44 | | 2.956931278 | neuropeptide receptor A34 |
| bmsk0011665 | 0.001 | | 0.74 | | 9.531381461 | XP 012545118.1 uncharacterized protein LOC100216500 isoform X2 |
| bmsk0013787 | 8.053333333 | | 2.026666667 | | -1.990477226 | putative zinc finger protein 840 isoform X1 [Amyelois transitella] |
| bmsk0011212 | 10.14 | | 2.396666667 | | -2.080956477 | D replication complex GINS protein PSF3-like [Plutella xylostella] |
| bmsk0007856 | 0.653333333 | | 2.65 | | 2.020101206 | adipose triglyceride lipase brummer |
| bmsk0014232 | 4.51 | | 0.823333333 | | -2.453578892 | XP 004923145.1 poly(A) polymerase type 3 |
| bmsk0001716 | 24.16 | | 7.936666667 | | -1.606015335 | probable R polymerase II nuclear localization protein SLC7A6OS isoform X2 |
| bmsk0010002 | 0.083333333 | | 1.526666667 | | 4.195347598 | inactive rhomboid protein 1, partial |
| bmsk0005420 | 34.53333333 | | 10.77666667 | | -1.68007859 | D -directed R polymerase II subunit RPB11 |
| bmsk0000261 | 1.136666667 | | 0.02 | | -5.828665428 | - |
| bmsk0005275 | 0.383333333 | | 0.016666667 | | -4.523561956 | glutamate receptor ionotropic, delta-2 |
| bmsk0009850 | 14.71666667 | | 5.583333333 | | -1.398252342 | protein shuttle craft |
| bmsk0010272 | 3.523333333 | | 0.403333333 | | -3.126896424 | endonuclease III-like protein 1 isoform X3 |
| bmsk0003827 | 0.573333333 | | 0.09 | | -2.671377253 | ras association domain-containing protein 8 isoform X1 [Papilio polytes] |
| bmsk0014487 | 44.90666667 | | 13.66 | | -1.716972154 | pinin [Amyelois transitella] |
| mstrg.26250 | 0.001 | | 0.87 | | 9.764871591 | hypothetical protein L798_04777 [Zootermopsis nevadensis] |
| bmsk0013792 | 0.033333333 | | 0.216666667 | | 2.700439718 | hypothetical protein TcasGA2 TC034914, partial [Tribolium castaneum] |
| bmsk0006171 | 2.173333333 | | 0.26 | | -3.063325935 | cell division cycle 25 isoform X1 |
| bmsk0010923 | 0.79 | | 0.001 | | -9.625708843 | pyroglutamyl-peptidase 1 isoform X1 |
| bmsk0002073 | 24.69 | | 7.96 | | -1.633086501 | conserved oligomeric Golgi complex subunit 6 [Amyelois transitella] |
| bmsk0010569 | 0.733333333 | | 0.126666667 | | -2.5334322 | supporter of activation of yellow protein |
| bmsk0014271 | 37.62333333 | | 12.18666667 | | -1.626324104 | vacuolar protein sorting-associated protein 26B-like [Amyelois transitella] |
| bmsk0003311 | 22.22 | | 7.86 | | -1.499257599 | leucine-rich repeat protein soc-2 homolog [Papilio xuthus] |
| bmsk0000801 | 0.03 | | 0.893333333 | | 4.896164189 | chorion class CA protein ERA.2-like isoform X1 |
| bmsk0010301 | 0.033333333 | | 0.403333333 | | 3.596935142 | XP 004927262.1 15-hydroxyprostaglandin dehydrogenase [ D(+)] |
| bmsk0008348 | 15.39666667 | | 5.153333333 | | -1.579040227 | nudC domain-containing protein 1 [Amyelois transitella] |
| bmsk0004094 | 10.57666667 | | 3.533333333 | | -1.581783256 | D -directed R polymerase III subunit RPC4 |
| bmsk0007019 | 15.47666667 | | 4.8 | | -1.688988469 | calcium/calmodulin-dependent protein kinase type 1 |
| bmsk0000360 | 0.166666667 | | 0.001 | | -7.380821784 | potassium channel subfamily T member 2 [Amyelois transitella] |
| bmsk0010842 | 0.213333333 | | 0.473333333 | | 1.14974712 | myosin-7B isoform X2 [Papilio xuthus] |
| bmsk0014218 | 10.69 | | 3.24 | | -1.722196135 | XP 004923153.1 D polymerase epsilon subunit 3 |
| bmsk0002205 | 29.8 | | 11.29 | | -1.400266845 | nuclear pore complex protein Nup93-like [Amyelois transitella] |
| bmsk0015955 | 0.07 | | 0.456666667 | | 2.70571466 | - |
| bmsk0007820 | 14.50333333 | | 3.503333333 | | -2.049584348 | protein SPT2 homolog |
| bmsk0002487 | 9.533333333 | | 3.51 | | -1.441509711 | N-acetylgalactosamine kinase |
| bmsk0007687 | 31.09666667 | | 10.72666667 | | -1.535558117 | mediator of R polymerase II transcription subunit 7 [Amyelois transitella] |
| bmsk0002097 | 7.37 | | 1.796666667 | | -2.036341847 | OTU domain-containing protein 6B |
| bmsk0000106 | 0.056666667 | | 0.001 | | -5.824428435 | laminin subunit alpha |
| bmsk0004033 | 16.47666667 | | 5.233333333 | | -1.654622347 | PX domain |
| bmsk0008780 | 10.1 | | 2.883333333 | | -1.808545756 | cyclin-C |
| bmsk0012661 | 52.58 | | 19.59 | | -1.424396744 | XP 021207427.1 ca2 /calmodulin-dependent protein kinase II isoform X1 |
| bmsk0005563 | 10.32333333 | | 3 | | -1.782874476 | lys-63-specific deubiquitinase BRCC36-like isoform X1 [Amyelois transitella] |
| bmsk0005172 | 0.06 | | 0.916666667 | | 3.933362807 | uncharacterized protein LOC101736774 isoform X2 |
| bmsk0014816 | 2.206666667 | | 0.036666667 | | -5.911255788 | XP 021202427.1 uncharacterized protein LOC101737526 |
| bmsk0014282 | 10.42666667 | | 3.3 | | -1.659740082 | XP 004923111.1 BUD13 homolog |
| bmsk0002036 | 0.033333333 | | 0.323333333 | | 3.277984747 | protein phosphatase PHLPP-like protein isoform X1 |
| bmsk0000615 | 2.346666667 | | 0.666666667 | | -1.815575429 | chromatin complexes subunit BAP18-like isoform X4 |
| mstrg.19357 | 11.37 | | 3.24 | | -1.811166536 | PREDICTED: zinc finger protein 267-like [Amyelois transitella] |
| mstrg.3832 | 0.373333333 | | 1.206666667 | | 1.692490965 | PREDICTED: acylphosphatase-2 [Bombyx mori] |
| bmsk0006974 | 168.3666667 | | 62.85333333 | | -1.421545378 | mitochondrial aldehyde dehydrogenase |
| mstrg.6998 | 22.32333333 | | 7.31 | | -1.610609156 | PREDICTED: COMM domain-containing protein 2-like isoform X3 [Bombyx mori] |
| bmsk0015379 | 7.563333333 | | 2.086666667 | | -1.857822047 | XP 004926155.1 ubiquitin-like domain-containing CTD phosphatase 1 |
| bmsk0008191 | 0.233333333 | | 0.001 | | -7.866248611 | myosin light chain kinase, smooth muscle-like [Amyelois transitella] |
| bmsk0000271 | 0.001 | | 0.113333333 | | 6.824428435 | serine/threonine-protein kinase OSR1-like [Amyelois transitella] |
| bmsk0004890 | 47.39666667 | | 18.65 | | -1.34560997 | U1 small nuclear ribonucleoprotein C [Amyelois transitella] |
| bmsk0015683 | 0.053333333 | | 0.126666667 | | 1.247927513 | facilitated trehalose transporter Tret1-like |
| bmsk0004384 | 0.593333333 | | 0.001 | | -9.212699025 | spindle assembly abnormal protein 6 homolog isoform X2 |
| bmsk0002893 | 0.536666667 | | 1.293333333 | | 1.268995964 | p38 map kinase |
| mstrg.26581 | 0.746666667 | | 1.813333333 | | 1.280107919 | insulin-like precursor polypeptide 2 [Spodoptera littoralis] |
| bmsk0013339 | 18.78666667 | | 7.096666667 | | -1.404495662 | ACT83403.2 cyclin dependent kinase 8 |
| bmsk0015581 | 5.81 | | 0.543333333 | | -3.4186287 | - |
| bmsk0004701 | 7.613333333 | | 2.43 | | -1.647571931 | uncharacterized protein LOC101739530 isoform X1 |
| bmsk0009296 | 0.163333333 | | 0.983333333 | | 2.5898613 | L-asparaginase |
| bmsk0001677 | 1.603333333 | | 0.001 | | -10.64685868 | uncharacterized protein LOC110385729 |
| bmsk0007895 | 71.28 | | 26.66666667 | | -1.418459838 | meiotic recombination 11 |
| bmsk0004639 | 28.69 | | 11.24666667 | | -1.351050495 | SWI/SNF-related matrix-associated actin-dependent regulator of chromatin subfamily A containing DEAD/H box 1 homolog isoform X1 |
| mstrg.23045 | 13.31 | | 5.23 | | -1.34762772 | PREDICTED: voltage-dependent calcium channel subunit alpha-2/delta-3 isoform X4 [Bombyx mori] |
| bmsk0013396 | 560.3266667 | | 225.2733333 | | -1.314591609 | XP 004932707.1 uncharacterized protein LOC101740613 |
| bmsk0011020 | 0.09 | | 0.206666667 | | 1.199308808 | XP 012552026.1 receptor-type tyrosine-protein phosphatase kappa isoform X1 |
| bmsk0009010 | 0.43 | | 0.001 | | -8.74819285 | cytochrome P450, family 337, subfamily a, polypeptide 1 precursor |
| bmsk0013115 | 16.31 | | 5.563333333 | | -1.551735328 | XP 021203221.1 methionine synthase reductase isoform X1 |
| bmsk0005238 | 0.1 | | 0.543333333 | | 2.441837559 | - |
| bmsk0001534 | 24.91 | | 8.113333333 | | -1.618358354 | myeloid leukemia factor |
| bmsk0010697 | 5.356666667 | | 1.093333333 | | -2.292602209 | dopamine N-acetyltransferase-like |
| bmsk0007176 | 21.83333333 | | 9.023333333 | | -1.274800019 | splicing factor U2af 38 kDa subunit |
| bmsk0007287 | 8.126666667 | | 2.643333333 | | -1.620305355 | transcription initiation factor IIE subunit beta [Papilio xuthus] |
| bmsk0000962 | 7.766666667 | | 2.34 | | -1.730787019 | LIM and SH3 domain protein Lasp isoform X4 [Papilio polytes] |
| bmsk0002180 | 2.726666667 | | 0.376666667 | | -2.855778071 | peptidyl-prolyl cis-trans isomerase G-like isoform X3 |
| bmsk0002861 | 10.3 | | 4.073333333 | | -1.338362553 | ubiquitin-protein ligase E3C [Amyelois transitella] |
| bmsk0006823 | 1.05 | | 0.213333333 | | -2.299208018 | DDB1- and CUL4-associated factor 7 [Papilio xuthus] |
| bmsk0000478 | 22.22666667 | | 6.85 | | -1.69811571 | carnitine O-palmitoyltransferase 1, liver isoform [Papilio machaon] |
| bmsk0014235 | 41.68 | | 15.93 | | -1.387609011 | ABF51346.1 LRP16 protein |
| bmsk0001622 | 0.193333333 | | 0.523333333 | | 1.436639754 | uncharacterized protein LOC105842097 |
| bmsk0013485 | 0.24 | | 0.006666667 | | -5.169925001 | XP 021206611.1 ovarian tumor protein isoform X1 |
| bmsk0006650 | 4.48 | | 1.083333333 | | -2.048021515 | mitochondrial thiamine pyrophosphate carrier |
| bmsk0003781 | 11.74333333 | | 4.383333333 | | -1.421741675 | putative D helicase Ino80 [Amyelois transitella] |
| mstrg.22788 | 8.036666667 | | 2.496666667 | | -1.686594027 | PREDICTED: cytoplasmic tRNA 2-thiolation protein 2 [Bombyx mori] |
| mstrg.22856 | 8.973333333 | | 3.17 | | -1.501161164 | PREDICTED: GPI ethanolamine phosphate transferase 1 [Bombyx mori] |
| bmsk0006869 | 0.01 | | 0.313333333 | | 4.969626351 | Uncharacterized protein OBRU01 18342 [Operophtera brumata] |
| bmsk0008347 | 2.423333333 | | 0.406666667 | | -2.575074216 | box C/D snoR protein 1 |
| bmsk0001682 | 0.133333333 | | 0.001 | | -7.058893689 | small GTP binding protein RAB5 |
| bmsk0010548 | 0.696666667 | | 2.206666667 | | 1.663328275 | Uncharacterized protein OBRU01 02397 [Operophtera brumata] |
| bmsk0010814 | 0.183333333 | | 0.41 | | 1.161154792 | uncharacterized protein K02A2.6-like [Papilio machaon] |
| bmsk0014247 | 0.116666667 | | 0.726666667 | | 2.638901308 | ABW86946.1 sex peptide receptor |
| bmsk0013579 | 13.71666667 | | 4.35 | | -1.656842624 | Nup53/35/40-type R recognition motif |
| bmsk0011205 | 18.13666667 | | 5.923333333 | | -1.614428147 | BAD74000.1 G protein alpha subunit Go |
| bmsk0010685 | 10.96333333 | | 3.353333333 | | -1.709018703 | Myb-MuvB complex subunit Lin-52 |
| bmsk0003557 | 0.06 | | 0.236666667 | | 1.979822118 | heterogeneous nuclear ribonucleoprotein R isoform X1 [Papilio xuthus] |
| mstrg.13150 | 17.35333333 | | 6.22 | | -1.480226326 | PREDICTED: snRNA-activating protein complex subunit 3 [Papilio machaon] |
| bmsk0009104 | 8.973333333 | | 1.973333333 | | -2.185009329 | crossover junction endonuclease EME1 isoform X2 |
| bmsk0013279 | 4.763333333 | | 1.49 | | -1.67665918 | protein FRA10AC1 homolog isoform X2 [Amyelois transitella] |
| bmsk0010597 | 13.87333333 | | 4.35 | | -1.673227158 | XP 021205066.1 protein SMG9 |
| bmsk0008846 | 17.06333333 | | 5.79 | | -1.559264252 | replication factor C4 |
| bmsk0011173 | 0.13 | | 0.001 | | -7.022367813 | XP 021206237.1 uncharacterized protein LOC101745797 |
| bmsk0011685 | 10.27333333 | | 2.963333333 | | -1.793611538 | XP 004925346.1 sister chromatid cohesion protein DCC1 isoform X1 |
| bmsk0004815 | 0.001 | | 0.09 | | 6.491853096 | 2-oxoisovalerate dehydrogenase subunit beta, mitochondrial |
| bmsk0011526 | 0.623333333 | | 0.04 | | -3.961931959 | XP 021207167.1 intraflagellar transport protein 52 homolog isoform X2 |
| bmsk0013226 | 1.773333333 | | 0.46 | | -1.946757979 | - |
| bmsk0013228 | 1.773333333 | | 0.46 | | -1.946757979 | - |
| bmsk0005517 | 22.08666667 | | 8.903333333 | | -1.310758227 | D -directed R polymerase III subunit RPC2 |
| mstrg.11293 | 0.001 | | 0.323333333 | | 8.336878436 | - |
| bmsk0006418 | 0.64 | | 0.083333333 | | -2.941106311 | uncharacterized protein C05D11.9 [Amyelois transitella] |
| bmsk0011885 | 5.41 | | 1.846666667 | | -1.550705118 | rhophilin-2-B-like isoform X3 [Plutella xylostella] |
| bmsk0009270 | 30.51333333 | | 9.26 | | -1.720355693 | elongator complex protein 5 |
| bmsk0013572 | 2.473333333 | | 0.28 | | -3.142957954 | - |
| bmsk0013171 | 12.75 | | 5.17 | | -1.302261061 | XP 004921733.1 UDP-N-acetylglucosamine--peptide N-acetylglucosaminyltransferase 110 kDa subunit |
| bmsk0006053 | 2.74 | | 0.563333333 | | -2.282115147 | uncharacterized protein LOC101741044 isoform X1 |
| bmsk0002113 | 1.703333333 | | 0.053333333 | | -4.997179481 | T-box transcription factor TBX20 isoform X2 |
| bmsk0012615 | 0.001 | | 0.126666667 | | 6.984893108 | cuticular protein RR-2 motif 92 precursor |
| bmsk0008683 | 7.866666667 | | 2.093333333 | | -1.909950395 | D replication complex GINS protein SLD5 |
| bmsk0005952 | 4.27 | | 1.436666667 | | -1.571510701 | zinc finger protein 62 homolog [Acyrthosiphon pisum] |
| bmsk0013662 | 2.23 | | 0.113333333 | | -4.298399559 | uncharacterized protein LOC101743235 isoform X3 |
| bmsk0011499 | 8.16 | | 2.783333333 | | -1.551755455 | 5-oxoprolinase [Amyelois transitella] |
| bmsk0006622 | 55.19666667 | | 22.55 | | -1.291453712 | SUMO-activating enzyme subunit 2 |
| bmsk0005123 | 7.62 | | 2.3 | | -1.728157137 | BRISC and BRCA1-A complex member 1 isoform X2 |
| bmsk0002306 | 5.53 | | 1.453333333 | | -1.927913846 | - |
| bmsk0014038 | 4.33 | | 1.226666667 | | -1.819623759 | zinc finger protein 37-like isoform X3 |
| bmsk0008318 | 0.286666667 | | 1.72 | | 2.584962501 | Kinesin motor domain |
| bmsk0002056 | 9.66 | | 3.15 | | -1.61667136 | uncharacterized protein LOC101744334 |
| bmsk0010022 | 6.526666667 | | 1.576666667 | | -2.049468676 | lysophospholipid acyltransferase 7-like [Amyelois transitella] |
| bmsk0002049 | 1.976666667 | | 4.67 | | 1.240352946 | - |
| bmsk0003334 | 6.293333333 | | 1.086666667 | | -2.533914895 | Homeodomain |
| bmsk0008094 | 0.073333333 | | 0.236666667 | | 1.690315501 | glucose dehydrogenase [FAD, quinone]-like [Papilio xuthus] |
| bmsk0000873 | 0.066666667 | | 0.223333333 | | 1.744161096 | angiotensin-converting enzyme-like [Amyelois transitella] |
| bmsk0006905 | 30.47 | | 11.26 | | -1.436182673 | engulfment and cell motility protein 1 [Papilio xuthus] |
| bmsk0007838 | 0.246666667 | | 0.001 | | -7.94641896 | - |
| bmsk0012131 | 7.96 | | 2.116666667 | | -1.91097434 | G patch domain and KOW motifs-containing protein [Plutella xylostella] |
| bmsk0012186 | 12.19666667 | | 2.403333333 | | -2.343378251 | XP 021205966.1 integrator complex subunit 3 homolog |
| bmsk0010333 | 5.766666667 | | 1.373333333 | | -2.070055795 | deoxynucleoside kinase-like isoform X1 [Papilio polytes] |
| bmsk0008095 | 0.046666667 | | 0.143333333 | | 1.618909833 | uncharacterized protein LOC105842505 |
| bmsk0003898 | 0.886666667 | | 1.913333333 | | 1.109624491 | uncharacterized protein LOC106131682 [Amyelois transitella] |
| bmsk0013258 | 10.13 | | 3.263333333 | | -1.63421591 | inhibitor of apoptosis protein isoform X1 |
| bmsk0005809 | 2.993333333 | | 0.896666667 | | -1.739109272 | D polymerase eta isoform X1 |
| bmsk0013351 | 5.213333333 | | 1.19 | | -2.131244533 | uncharacterized protein LOC107067961 isoform X2 [Polistes dominula] |
| bmsk0008956 | 0.143333333 | | 0.543333333 | | 1.9224634 | aspartate aminotransferase, cytoplasmic |
| bmsk0007392 | 0.133333333 | | 0.013333333 | | -3.321928095 | Exocyst complex component 7 [Papilio machaon] |
| bmsk0003328 | 0.726666667 | | 0.001 | | -9.505149919 | Hox cluster protein Shx15 |
| mstrg.19359 | 0.163333333 | | 0.563333333 | | 1.786169592 | Yokozuna [Bombyx mori] |
| bmsk0000281 | 1.916666667 | | 0.143333333 | | -3.741153391 | Uncharacterized protein OBRU01 00585 [Operophtera brumata] |
| bmsk0007525 | 0.086666667 | | 0.001 | | -6.437405312 | probable phosphorylase b kinase regulatory subunit alpha isoform X2 |
| bmsk0012067 | 0.001 | | 0.333333333 | | 8.380821784 | cuticular protein RR-2 motif 77 precursor |
| bmsk0004497 | 27.19 | | 8.95 | | -1.603116564 | histone R hairpin-binding protein |
| bmsk0002427 | 44.15666667 | | 16.23333333 | | -1.443671997 | nucleoporin Nup43 [Amyelois transitella] |
| bmsk0002454 | 7.89 | | 2.346666667 | | -1.749412372 | cyclin E |
| bmsk0000112 | 1.736666667 | | 0.496666667 | | -1.805971042 | bromodomain-containing protein DDB G0280777-like |
| bmsk0015360 | 0.143333333 | | 0.001 | | -7.163230349 | XP 012546957.1 negative elongation factor A isoform X2 |
| mstrg.21657 | 20.91 | | 6.736666667 | | -1.634086241 | PREDICTED: nuclear fragile X mental retardation-interacting protein 1 isoform X1 [Bombyx mori] |
| bmsk0012822 | 0.003333333 | | 0.093333333 | | 4.807354922 | XP 021204235.1 zonadhesin |
| bmsk0014432 | 38.58 | | 15.90333333 | | -1.278523958 | U4/U6.U5 small nuclear ribonucleoprotein 27 kDa protein |
| bmsk0006502 | 0.001 | | 0.276666667 | | 8.112005026 | tR -splicing endonuclease subunit Sen34 [Papilio xuthus] |
| bmsk0006829 | 12.69666667 | | 4.763333333 | | -1.414406371 | uncharacterized protein LOC101737213 |
| bmsk0010222 | 23.74 | | 7.85 | | -1.596555376 | non-histone protein 10 [Papilio polytes] |
| bmsk0012016 | 48.89666667 | | 14.62333333 | | -1.741463913 | hnRNPA/B-like 28 isoform X2 |
| bmsk0013230 | 12.39666667 | | 4.04 | | -1.617525049 | translation initiation factor 2 gamma subunit |
| bmsk0013102 | 0.036666667 | | 0.323333333 | | 3.140481224 | aminoacylase |
| bmsk0002399 | 0.11 | | 0.423333333 | | 1.944290567 | Caveolin |
| bmsk0013261 | 4.056666667 | | 1.156666667 | | -1.8103216 | uncharacterized protein LOC106137584 [Amyelois transitella] |
| bmsk0004818 | 0.233333333 | | 1.596666667 | | 2.774598829 | fatty-acid amide hydrolase 2-A-like isoform X3 |
| bmsk0005819 | 14.95333333 | | 5.33 | | -1.488259682 | mediator of R polymerase II transcription subunit 19 |
| bmsk0003326 | 0.336666667 | | 0.001 | | -8.395177077 | uncharacterized protein LOC101742071 |
| bmsk0011610 | 9.17 | | 2.533333333 | | -1.855884816 | Uncharacterized protein OBRU01 00296 [Operophtera brumata] |
| bmsk0013830 | 19.43 | | 7.23 | | -1.426218349 | XP 004923744.1 m7GpppX diphosphatase |
| bmsk0013831 | 19.43 | | 7.23 | | -1.426218349 | XP 004923745.1 uncharacterized protein LOC101739995 |
| bmsk0010045 | 0.786666667 | | 0.176666667 | | -2.154722595 | uncharacterized protein LOC105841590 isoform X1 |
| bmsk0000210 | 36.43 | | 12.86666667 | | -1.501488648 | Ubiquitin conjugating enzyme 4 [Operophtera brumata] |
| bmsk0000592 | 0.001 | | 0.616666667 | | 9.268347055 | - |
| bmsk0012612 | 0.001 | | 0.276666667 | | 8.112005026 | cuticular protein RR-2 motif 96 precursor |
| bmsk0015941 | 11.70333333 | | 4.18 | | -1.485344648 | XP 004923460.1 nuclear pore glycoprotein p62 |
| bmsk0013208 | 42.13666667 | | 15.80333333 | | -1.414847298 | histone deacetylase complex subunit SAP18 [Papilio machaon] |
| bmsk0003157 | 0.001 | | 0.29 | | 8.17990909 | UPF0565 protein C2orf69 homolog |
| bmsk0008872 | 11.57 | | 3.553333333 | | -1.703143927 | uncharacterized protein LOC101740766 |
| mstrg.20636 | 12.64333333 | | 4.826666667 | | -1.389277769 | PREDICTED: ATP-dependent RNA helicase DHX36 [Bombyx mori] |
| bmsk0009172 | 0.316666667 | | 1.603333333 | | 2.340037475 | Uncharacterized protein OBRU01 06884 [Operophtera brumata] |
| bmsk0004480 | 0.3 | | 3.243333333 | | 3.434442898 | - |
| bmsk0011125 | 0.001 | | 0.053333333 | | 5.736965594 | thrombospondin type-1 domain-containing protein 4-like precursor |
| mstrg.7241 | 10.76333333 | | 4.11 | | -1.388914641 | PREDICTED: YTH domain-containing family protein 3 isoform X2 [Bombyx mori] |
| bmsk0000679 | 0.073333333 | | 0.386666667 | | 2.398549376 | - |
| bmsk0012604 | 0.393333333 | | 0.81 | | 1.042169454 | putative cuticle protein |
| bmsk0001877 | 1.17 | | 0.38 | | -1.622437206 | golgin subfamily A member 1 isoform X2 |
| bmsk0013578 | 8.986666667 | | 3.183333333 | | -1.497247858 | XP 012549767.1 R -binding protein 26 isoform X1 |
| bmsk0009419 | 0.523333333 | | 0.001 | | -9.031586343 | Optomotor-blind protein [Papilio machaon] |
| bmsk0007492 | 0.29 | | 0.006666667 | | -5.442943496 | uncharacterized protein LOC101742063 isoform X3 |
| bmsk0014486 | 18.77333333 | | 6.473333333 | | -1.536104133 | XP 004925700.1 ADP-ribosylation factor-like protein 2 |
| bmsk0006348 | 15.32 | | 5.66 | | -1.436542339 | Regulation of nuclear pre-mR domain-containing protein 1B [Operophtera brumata] |
| bmsk0002062 | 0.106666667 | | 0.47 | | 2.139551352 | MORN repeat |
| bmsk0003461 | 0.086666667 | | 0.49 | | 2.499232627 | suppressor of lurcher protein 1 |
| bmsk0003381 | 0.433333333 | | 0.001 | | -8.759333407 | uncharacterized protein LOC101744810 isoform X2 |
| bmsk0010930 | 0.153333333 | | 0.376666667 | | 1.296617006 | uncharacterized protein LOC101737484 |
| bmsk0007734 | 2.69 | | 0.44 | | -2.612030744 | uncharacterized protein LOC101743404 isoform X1 |
| bmsk0001106 | 156.19 | | 56.93 | | -1.456041084 | protein charybde-like [Papilio polytes] |
| bmsk0005803 | 28.44333333 | | 11.61666667 | | -1.291894392 | ATP-dependent R helicase DHX8 [Papilio xuthus] |
| bmsk0003878 | 3.18 | | 0.65 | | -2.290515142 | uncharacterized protein LOC101745257 isoform X1 |
| bmsk0000376 | 1.233333333 | | 0.001 | | -10.26834705 | THAP domain-containing protein 9, partial [Camponotus floridanus] |
| bmsk0014710 | 9.913333333 | | 2.446666667 | | -2.018552679 | uncharacterized protein LOC106126398 isoform X3 [Papilio xuthus] |
| bmsk0007101 | 1.583333333 | | 0.096666667 | | -4.033802708 | Uncharacterized protein OBRU01 02243 [Operophtera brumata] |
| bmsk0009423 | 3.103333333 | | 0.34 | | -3.190212016 | FancJ-like protein |
| bmsk0012951 | 81.23333333 | | 30.63666667 | | -1.406812508 | proliferating cell nuclear antigen isoform X1 |
| bmsk0011270 | 3.586666667 | | 0.193333333 | | -4.213481367 | XP 021206608.1 protein MLP1 isoform X3 |
| bmsk0011417 | 6.23 | | 1.806666667 | | -1.785901812 | XP 004926953.3 NF-X1-type zinc finger protein NFXL1 |
| bmsk0010539 | 0.68 | | 0.116666667 | | -2.543142325 | XP 012547539.2 uncharacterized protein LOC101739032 |
| bmsk0004816 | 80.77333333 | | 32.67333333 | | -1.305765431 | uncharacterized protein LOC106130280 [Amyelois transitella] |
| bmsk0005953 | 0.273333333 | | 0.023333333 | | -3.550197083 | zinc finger protein 271-like isoform X2 |
| bmsk0007647 | 0.73 | | 0.07 | | -3.382469637 | ferritin heavy chain-like |
| bmsk0002358 | 0.026666667 | | 0.2 | | 2.906890596 | Arrestin (or S-antigen), N-terminal domain |
| bmsk0006923 | 4.22 | | 0.826666667 | | -2.351865379 | - |
| bmsk0014651 | 0.123333333 | | 0.806666667 | | 2.709409872 | uncharacterized protein LOC101741141 isoform X1 |
| bmsk0003891 | 0.286666667 | | 0.001 | | -8.163230349 | uncharacterized protein LOC101746017 isoform X3 |
| bmsk0003949 | 0.026666667 | | 1.123333333 | | 5.396604781 | tight junction protein ZO-1 isoform X13 |
| bmsk0013491 | 8.99 | | 3.67 | | -1.292541053 | XP 021206718.1 tyrosine-protein phosphatase non-receptor type 21 |
| bmsk0001650 | 0.2 | | 0.001 | | -7.64385619 | laminin subunit alpha-2 |
| bmsk0000067 | 0.003333333 | | 0.036666667 | | 3.459431619 | titin homolog |
| bmsk0011674 | 1.7 | | 0.046666667 | | -5.186998515 | uncharacterized protein LOC101744317 isoform X2 |
| bmsk0005073 | 2.246666667 | | 0.353333333 | | -2.668684327 | uncharacterized protein LOC101738420 isoform X1 |
| bmsk0004610 | 0.436666667 | | 0.001 | | -8.770388596 | Ubiquitin carboxyl-terminal hydrolase 5 [Papilio xuthus] |
| bmsk0008706 | 0.196666667 | | 0.453333333 | | 1.204819792 | aquaporin |
| mstrg.18231 | 11.56333333 | | 3.533333333 | | -1.710455576 | PREDICTED: phosphatidylinositol N-acetylglucosaminyltransferase subunit H isoform X2 [Bombyx mori] |
| bmsk0002250 | 1.413333333 | | 0.243333333 | | -2.538095896 | intraflagellar transport protein 80 homolog isoform X1 [Amyelois transitella] |
| bmsk0003224 | 0.076666667 | | 0.506666667 | | 2.724365557 | uncharacterized protein LOC101741188 |
| bmsk0005378 | 7.986666667 | | 1.146666667 | | -2.800147438 | - |
| bmsk0000213 | 0.001 | | 0.39 | | 8.607330314 | peptidoglycan-recognition protein 1-like isoform X2 [Papilio machaon] |
| mstrg.2380 | 0.996666667 | | 0.07 | | -3.831684251 | PREDICTED: zinc finger protein 16-like isoform X2 [Bombyx mori] |
| bmsk0001505 | 0.001 | | 0.116666667 | | 6.866248611 | G patch domain and ankyrin repeat-containing protein 1 homolog [Papilio machaon] |
| bmsk0000692 | 6.79 | | 2.096666667 | | -1.695314058 | G-protein-signaling modulator 2 isoform X1 [Amyelois transitella] |
| bmsk0011118 | 7.71 | | 1.923333333 | | -2.003122042 | ras-related protein Rab-43 [Amyelois transitella] |
| mstrg.21039 | 0.173333333 | | 1.006666667 | | 2.537965021 | PREDICTED: NACHT domain- and WD repeat-containing protein 1 [Papilio xuthus] |
| bmsk0005942 | 8.196666667 | | 1.546666667 | | -2.405875024 | uncharacterized protein LOC101740704, partial |
| bmsk0011352 | 10.41666667 | | 3.666666667 | | -1.506352666 | XP 004924242.1 R 3'-terminal phosphate cyclase |
| bmsk0011960 | 5.526666667 | | 1.956666667 | | -1.498011598 | hypothetical protein KGM 08900 [Danaus plexippus] |
| bmsk0005217 | 20.12 | | 7.88 | | -1.35236277 | dynactin subunit 4 [Papilio machaon] |
| bmsk0012910 | 0.001 | | 0.263333333 | | 8.040746342 | XP 012544597.1 L-dopachrome tautomerase yellow-f-like |
| bmsk0009399 | 0.001 | | 0.4 | | 8.64385619 | apyrase-like [Amyelois transitella] |
| bmsk0007718 | 0.01 | | 0.246666667 | | 4.624490865 | uncharacterized protein LOC101735921 |
| bmsk0005124 | 28.95 | | 11.30333333 | | -1.356815065 | RWD domain-containing protein 1 isoform X2 |
| bmsk0002627 | 0.386666667 | | 0.876666667 | | 1.180937994 | sodium/hydrogen exchanger 7, 9, partial [Manduca sexta] |
| bmsk0007862 | 0.4 | | 0.06 | | -2.736965594 | phosphatidylinositol N-acetylglucosaminyltransferase subunit A [Papilio polytes] |
| bmsk0001271 | 0.001 | | 0.116666667 | | 6.866248611 | uncharacterized protein LOC105842655 |
| bmsk0006804 | 0.006666667 | | 0.1 | | 3.906890596 | protein toll-like |
| bmsk0006033 | 6.683333333 | | 2.076666667 | | -1.686298168 | WD repeat domain-containing protein 83 |
| bmsk0000623 | 26.06666667 | | 10.09 | | -1.369279932 | CCR4-NOT transcription complex subunit 1 isoform X1 |
| bmsk0012339 | 16.06666667 | | 5.08 | | -1.661170244 | XP 004925056.1 conserved oligomeric Golgi complex subunit 5 |
| bmsk0005456 | 0.001 | | 0.12 | | 6.906890596 | uncharacterized protein LOC105842218 |
| bmsk0011137 | 1.506666667 | | 0.506666667 | | -1.572251449 | transmembrane protein adipocyte-associated 1 homolog isoform X2 |
| bmsk0007988 | 0.1 | | 0.001 | | -6.64385619 | NEDD8 ultimate buster 1 isoform X2 |
| bmsk0012334 | 5.653333333 | | 1.866666667 | | -1.598637438 | XP 004925050.1 protein FAM172A |
| bmsk0014716 | 0.001 | | 0.143333333 | | 7.163230349 | 4-coumarate--CoA ligase 1-like |
| bmsk0007789 | 0.45 | | 0.046666667 | | -3.269460675 | histone deacetylase complex subunit SAP30 homolog |
| bmsk0011792 | 0.013333333 | | 0.073333333 | | 2.459431619 | ADI47117.1 antennal esterase |
| bmsk0010139 | 5.373333333 | | 0.09 | | -5.899748526 | serine/threonine-protein kinase ATR |
| bmsk0011826 | 0.36 | | 1.016666667 | | 1.49777793 | - |
| bmsk0003151 | 0.003333333 | | 0.59 | | 7.46760555 | carbonic anhydrase 2-like |
| bmsk0007811 | 9.603333333 | | 3.59 | | -1.419551411 | tudor domain-containing protein 3-like |
| mstrg.25460 | 11.77333333 | | 3.816666667 | | -1.625137745 | MyD88 [Spodoptera frugiperda] |
| bmsk0007380 | 0.55 | | 0.016666667 | | -5.044394119 | helicase POLQ-like isoform X1 |
| mstrg.18568 | 13.67333333 | | 3.8 | | -1.847293667 | cytochrome P450, partial [Bombyx mori] |
| bmsk0014251 | 0.116666667 | | 1.333333333 | | 3.514573173 | KWMTBOMO14905 |
| bmsk0009537 | 0.15 | | 0.34 | | 1.180572246 | - |
| bmsk0006761 | 14.59 | | 4.943333333 | | -1.561423786 | uncharacterized protein LOC101743313 |
| bmsk0002544 | 7.843333333 | | 0.15 | | -5.708432509 | nucleoporin NUP188 homolog [Amyelois transitella] |
| bmsk0011257 | 21.35666667 | | 8.46 | | -1.335956921 | 3-hydroxy-3-methylglutaryl-CoA synthase |
| bmsk0008119 | 4.213333333 | | 0.273333333 | | -3.946228744 | Cathepsin propeptide inhibitor domain (I29) |
| bmsk0005910 | 4.223333333 | | 1.093333333 | | -1.949648805 | R -binding protein 48 |
| bmsk0003435 | 17.81666667 | | 5.7 | | -1.644193623 | pre-mR -splicing factor 18 isoform X1 |
| bmsk0013322 | 0.01 | | 0.256666667 | | 4.68182404 | XP 012551313.1 juvenile hormone binding protein ce-0330 isoform X2 |
| bmsk0008849 | 13.79333333 | | 4.763333333 | | -1.533927729 | ribonuclease H1 |
| bmsk0004921 | 11.69666667 | | 3.92 | | -1.577171888 | zinc finger protein 830 [Papilio polytes] |
| bmsk0014450 | 0.09 | | 0.503333333 | | 2.483517237 | eIF2B-beta protein |
| bmsk0004777 | 0.066666667 | | 0.176666667 | | 1.40599236 | katanin p60 ATPase-containing subunit A-like 1 |
| bmsk0015036 | 8.626666667 | | 2.076666667 | | -2.054533549 | CREB-regulated transcription coactivator 1 |
| bmsk0000425 | 0.363333333 | | 0.023333333 | | -3.960829403 | polypeptide N-acetylgalactosaminyltransferase 11 |
| bmsk0001066 | 0.13 | | 0.001 | | -7.022367813 | uncharacterized protein LOC101738867 |
| bmsk0014244 | 0.001 | | 0.06 | | 5.906890596 | XP 004923181.2 lysosome-associated membrane glycoprotein 1 |
| bmsk0001191 | 1.46 | | 0.27 | | -2.434937057 | zinc finger protein 615 isoform X4 |
| bmsk0004513 | 26.62333333 | | 8.996666667 | | -1.565228737 | THO complex subunit 5 homolog |
| bmsk0001112 | 3.266666667 | | 1.076666667 | | -1.601247584 | U5 small nuclear ribonucleoprotein 40 kDa protein |
| bmsk0008487 | 7.75 | | 2.596666667 | | -1.577535483 | U4/U6.U5 tri-snRNP-associated protein 2 |
| bmsk0014211 | 1.573333333 | | 0.046666667 | | -5.075288127 | XP 004923155.1 ADP-ribosylation factor-like protein 13B |
| bmsk0010007 | 0.001 | | 0.066666667 | | 6.058893689 | putative inorganic phosphate cotransporter isoform X1 [Amyelois transitella] |
| bmsk0004367 | 0.37 | | 0.001 | | -8.531381461 | general transcription factor IIH subunit 2 [Papilio polytes] |
| bmsk0003001 | 35.55666667 | | 13.29 | | -1.419778977 | tripeptidyl-peptidase 2 isoform X2 |
| bmsk0006416 | 25.71666667 | | 9.98 | | -1.365591935 | zinc finger CCCH domain-containing protein 18 isoform X3 |
| bmsk0015808 | 0.056666667 | | 0.296666667 | | 2.38827059 | - |
| mstrg.14038 | 0.696666667 | | 0.066666667 | | -3.385431037 | Retrovirus-related Pol polyprotein from transposon 412-like Protein [Tribolium castaneum] |
| bmsk0007614 | 0.203333333 | | 0.023333333 | | -3.123382416 | centrin |
| bmsk0014640 | 19.73 | | 6.133333333 | | -1.685647691 | hypothetical protein KGM 00713 [Danaus plexippus] |
| bmsk0011756 | 1.523333333 | | 0.12 | | -3.666125354 | hypothetical protein ALC57 15604 [Trachymyrmex cornetzi] |
| bmsk0015457 | 0.413333333 | | 0.001 | | -8.691161905 | potassium voltage-gated channel protein Shal isoform X3 [Amyelois transitella] |
| bmsk0007465 | 6.106666667 | | 2.06 | | -1.56774076 | protein FAM50 homolog |
| bmsk0008350 | 2.996666667 | | 0.026666667 | | -6.812177306 | transcription factor E74, partial |
| bmsk0003568 | 0.236666667 | | 0.74 | | 1.644668747 | - |
| bmsk0007769 | 1.506666667 | | 0.346666667 | | -2.119739244 | transmembrane protein 184C [Papilio xuthus] |
| bmsk0007694 | 4.25 | | 1.266666667 | | -1.746425923 | eye-specific diacylglycerol kinase isoform X3 |
| bmsk0008111 | 0.01 | | 0.153333333 | | 3.938599455 | transient-receptor-potential-like protein [Amyelois transitella] |
| bmsk0009434 | 18 | | 6.336666667 | | -1.506200875 | RAN binding protein |
| bmsk0002572 | 3.263333333 | | 0.876666667 | | -1.89624606 | dual specificity protein phosphatase 3 isoform X3 |
| bmsk0003101 | 2.75 | | 0.5 | | -2.459431619 | scavenger receptor class B member 1-like isoform X1 |
| bmsk0012506 | 1.27 | | 0.213333333 | | -2.573647187 | XP 021208673.1 ATP-binding cassette sub-family D member 3, partial |
| bmsk0004186 | 0.06 | | 0.133333333 | | 1.152003093 | lipase 1-like |
| bmsk0008564 | 0.136666667 | | 0.32 | | 1.227410496 | uncharacterized protein LOC101745341 |
| bmsk0010643 | 2.236666667 | | 0.603333333 | | -1.890323069 | leucine-rich repeat-containing protein 58-like [Amyelois transitella] |
| bmsk0012519 | 0.253333333 | | 0.01 | | -4.662965013 | putative sugar transporter protein 5 |
| mstrg.8014 | 25.21 | | 8.65 | | -1.54322408 | hypothetical protein g.14677 [Pectinophora gossypiella] |
| mstrg.5310 | 18.78 | | 7.073333333 | | -1.408734907 | PREDICTED: nuclear pore complex protein Nup50 [Bombyx mori] |
| bmsk0011679 | 0.473333333 | | 0.063333333 | | -2.901819606 | phosphoinositide 3-kinase regulatory subunit 4 [Papilio polytes] |
| bmsk0000591 | 0.001 | | 0.336666667 | | 8.395177077 | sodium-dependent nutrient amino acid transporter 1-like |
| mstrg.10979 | 0.25 | | 0.516666667 | | 1.047305715 | PREDICTED: uncharacterized protein LOC105842596 [Bombyx mori] |
| bmsk0007679 | 0.001 | | 0.183333333 | | 7.518325308 | - |
| bmsk0013809 | 2.473333333 | | 0.653333333 | | -1.920565533 | XP 012543783.1 heterogeneous nuclear ribonucleoprotein L isoform X1 |
| mstrg.26759 | 3.536666667 | | 1.31 | | -1.432823439 | PREDICTED: DNA polymerase zeta catalytic subunit isoform X1 [Bombyx mori] |
| bmsk0012909 | 0.001 | | 0.54 | | 9.076815597 | XP 012552611.2 L-dopachrome tautomerase yellow-f-like, partial |
| bmsk0005619 | 0.22 | | 0.001 | | -7.781359714 | cationic peptide CP8 precursor [Bombyx mandarina] |
| bmsk0011691 | 0.46 | | 0.013333333 | | -5.108524457 | XP 021203087.1 anillin-like |
| mstrg.14416 | 0.19 | | 0.386666667 | | 1.025090981 | PREDICTED: cuticular protein RR-1 motif 33 isoform X1 [Bombyx mori] |
| bmsk0012774 | 2.286666667 | | 0.763333333 | | -1.582860978 | Rapamycin-insensitive companion of mTOR, domain 5 |
| mstrg.22680 | 36.91333333 | | 14.54666667 | | -1.34345342 | PREDICTED: snurportin-1 [Bombyx mori] |
| bmsk0011177 | 1.656666667 | | 0.293333333 | | -2.497670423 | vasa-like |
| bmsk0015983 | 0.001 | | 0.163333333 | | 7.351675438 | uncharacterized protein LOC101736452 isoform X2 |
| bmsk0015653 | 0.183333333 | | 0.001 | | -7.518325308 | zinc finger protein 90-like isoform X2 |
| bmsk0006122 | 0.036666667 | | 0.223333333 | | 2.606657572 | - |
| bmsk0010142 | 5.706666667 | | 0.886666667 | | -2.686184551 | U6 snR -associated Sm-like protein LSm5 |
| bmsk0004092 | 1.99 | | 0.526666667 | | -1.917806373 | uncharacterized protein LOC110385281 |
| bmsk0001600 | 0.001 | | 0.103333333 | | 6.691161905 | serpin-17 |
| bmsk0005032 | 0.001 | | 0.373333333 | | 8.544320516 | - |
| bmsk0012079 | 2.153333333 | | 0.47 | | -2.195839002 | class E vacuolar protein-sorting machinery protein hse1-like isoform X2 |
| mstrg.5388 | 11.11333333 | | 3.566666667 | | -1.639643308 | PREDICTED: gastrula zinc finger protein XlCGF17.1-like [Bombyx mori] |
| bmsk0008537 | 25.01 | | 7.756666667 | | -1.688996347 | zyxin |
| bmsk0006984 | 9.36 | | 3.62 | | -1.370518833 | ATP-binding cassette sub-family G member 4 |
| bmsk0008184 | 22.39 | | 7.996666667 | | -1.485383871 | cleavage and polyadenylation specificity factor subunit CG7185 isoform X3 |
| bmsk0009703 | 1.07 | | 0.043333333 | | -4.625989769 | forkhead box protein K1 |
| bmsk0009191 | 0.003333333 | | 0.04 | | 3.584962501 | serine proteinase stubble-like [Amyelois transitella] |
| bmsk0006670 | 0.01 | | 0.563333333 | | 5.815916936 | collagen alpha-1(XVIII) chain-like |
| bmsk0009051 | 2.386666667 | | 0.273333333 | | -3.126263773 | GATA-beta4, partial |
| bmsk0011531 | 27.72 | | 11.18 | | -1.310007069 | zinc finger protein 585A-like [Amyelois transitella] |
| bmsk0010954 | 0.001 | | 0.43 | | 8.74819285 | AEL88625.1 amino acid transporter-like protein |
| bmsk0007958 | 0.033333333 | | 0.183333333 | | 2.459431619 | carboxypeptidase B-like precursor |
| bmsk0002901 | 2.223333333 | | 0.556666667 | | -1.997838659 | FAST kinase domain-containing protein 3, mitochondrial |
| bmsk0013379 | 373.9533333 | | 146.17 | | -1.355211001 | ubiquitin-like protein SMT3 |
| bmsk0007771 | 1.116666667 | | 0.3 | | -1.896164189 | thymidylate synthase isoform 1 |
| bmsk0003366 | 7.67 | | 2.636666667 | | -1.540511384 | programmed cell death protein 2-like [Amyelois transitella] |
| bmsk0012767 | 5.853333333 | | 1.7 | | -1.783723693 | rab-like protein 3 isoform X1 [Amyelois transitella] |
| bmsk0006607 | 0.13 | | 0.613333333 | | 2.238159737 | alpha-esterase 13 precursor |
| bmsk0004743 | 0.243333333 | | 2.156666667 | | 3.147797343 | tyrosine kinase receptor Cad96Ca isoform X2 [Amyelois transitella] |
| bmsk0005650 | 3.703333333 | | 1.173333333 | | -1.658211483 | adenylate kinase 9 isoform X2 [Amyelois transitella] |
| bmsk0013159 | 6.83 | | 2.58 | | -1.404514513 | XP 004921725.1 SET and MYND domain-containing protein 5 |
| bmsk0013114 | 0.68 | | 0.023333333 | | -4.86507042 | XP 004925513.1 uncharacterized protein LOC101746333 |
| bmsk0013383 | 5.076666667 | | 1.213333333 | | -2.064905586 | protein SON isoform X1 |
| bmsk0011441 | 0.001 | | 0.14 | | 7.129283017 | organic cation transporter 1-like |
| bmsk0000492 | 0.086666667 | | 0.26 | | 1.584962501 | phosphogluconate dehydrogenase, partial |
| bmsk0005900 | 0.226666667 | | 0.033333333 | | -2.765534746 | BTB/POZ domain-containing protein 7-like |
| bmsk0013841 | 0.001 | | 0.063333333 | | 5.984893108 | XP 021202034.1 monoacylglycerol lipase ABHD12-like |
| bmsk0007867 | 0.206666667 | | 0.001 | | -7.691161905 | uncharacterized protein LOC101737689 |
| bmsk0010949 | 5.316666667 | | 0.886666667 | | -2.584058273 | origin recognition complex subunit 1 |
| bmsk0014179 | 0.28 | | 0.01 | | -4.807354922 | XP 012552875.1 cuticular protein RR-2 motif 132 isoform X1 |
| bmsk0007950 | 1.316666667 | | 0.293333333 | | -2.166277224 | uncharacterized protein LOC106719359 [Papilio machaon] |
| bmsk0000188 | 7.713333333 | | 2.97 | | -1.376891528 | syntaxin-7 [Amyelois transitella] |
| bmsk0001241 | 13.47 | | 5.49 | | -1.294871796 | tail-anchored protein insertion receptor WRB-like [Papilio polytes] |
| mstrg.25168 | 4.96 | | 1.326666667 | | -1.902534191 | PREDICTED: importin subunit alpha-2 isoform X1 [Bombyx mori] |
| bmsk0012232 | 0.153333333 | | 0.393333333 | | 1.359081093 | - |
| bmsk0005150 | 8.043333333 | | 2.83 | | -1.506991457 | hypothetical protein KGM 14799 [Danaus plexippus] |
| bmsk0010327 | 0.05 | | 0.383333333 | | 2.938599455 | - |
| bmsk0016057 | 0.193333333 | | 0.001 | | -7.594946589 | XP 004924362.1 uncharacterized protein LOC101743968 |
| bmsk0001985 | 1.003333333 | | 0.001 | | -9.970585271 | dipeptidase 1-like |
| bmsk0009902 | 255.0266667 | | 108.0233333 | | -1.239305137 | ACT4 BOMMO RecName: Full=Actin, cytoplasmic A4; Flags: Precursor |
| bmsk0009471 | 6.323333333 | | 2.006666667 | | -1.655884287 | cell cycle checkpoint control protein RAD9A [Amyelois transitella] |
| bmsk0009347 | 0.001 | | 0.443333333 | | 8.79224803 | - |
| bmsk0009499 | 0.05 | | 0.193333333 | | 1.9510904 | Golgi S P receptor complex member 1 |
| bmsk0013123 | 0.03 | | 0.096666667 | | 1.688055994 | XP 004921706.2 uncharacterized protein LOC101745641 |
| bmsk0010978 | 50.10333333 | | 19.28666667 | | -1.377302765 | Transmembrane protein 214 [Papilio xuthus] |
| bmsk0010151 | 1.713333333 | | 0.546666667 | | -1.648072545 | - |
| bmsk0011479 | 13.45333333 | | 4.303333333 | | -1.644437174 | PB1 domain |
| bmsk0015824 | 0.213333333 | | 0.001 | | -7.736965594 | XP 021208537.1 protein Wnt-7b isoform X2 |
| bmsk0008157 | 0.001 | | 0.3 | | 8.22881869 | U8-agatoxin-Ao1a-like |
| bmsk0010797 | 0.836666667 | | 0.043333333 | | -4.271103836 | connectin-like |
| bmsk0001520 | 0.243333333 | | 14.78666667 | | 5.925219091 | mitochondrial 2-oxoglutarate/malate carrier protein isoform X2 |
| bmsk0015088 | 10.47333333 | | 3.813333333 | | -1.457596129 | XP 004933015.1 upstream activation factor subunit spp27 |
| bmsk0011276 | 0.043333333 | | 0.126666667 | | 1.547487795 | XP 021206667.1 ninjurin-2 isoform X3 |
| bmsk0003560 | 1.87 | | 0.3 | | -2.640003864 | TELO2-interacting protein 1 homolog |
| bmsk0008838 | 2.566666667 | | 0.496666667 | | -2.369546115 | heparan-sulfate 6-O-sulfotransferase 2 |
| bmsk0013400 | 12.57666667 | | 4.93 | | -1.351090048 | uncharacterized protein LOC101738318 |
| bmsk0001137 | 0.853333333 | | 0.103333333 | | -3.04580369 | uncharacterized protein LOC105380908 [Plutella xylostella] |
| bmsk0009824 | 0.79 | | 0.001 | | -9.625708843 | uncharacterized protein LOC105841610 isoform X1 |
| bmsk0013393 | 0.296666667 | | 0.006666667 | | -5.475733431 | XP 004932766.1 catalase |
| bmsk0008719 | 0.001 | | 0.746666667 | | 9.544320516 | probable multidrug resistance-associated protein lethal(2)03659 |
| bmsk0013056 | 10.19 | | 3.743333333 | | -1.444758624 | formin-binding protein 4-like |
| bmsk0009389 | 0.056666667 | | 0.65 | | 3.519867472 | uncharacterized protein LOC105842048 |
| bmsk0000555 | 2.34 | | 0.36 | | -2.700439718 | glucuronyltransferase |
| bmsk0014471 | 2.97 | | 0.766666667 | | -1.953791571 | - |
| bmsk0004994 | 0.001 | | 2.55 | | 11.31628153 | Thioredoxin domain-containing protein [Papilio xuthus] |
| bmsk0005966 | 22.66333333 | | 8.74 | | -1.374654884 | myoneurin-like |
| bmsk0010289 | 1.726666667 | | 0.413333333 | | -2.062611977 | XP 021207808.1 protein tweety isoform X2 |
| bmsk0014986 | 17.84333333 | | 6.32 | | -1.497388688 | HIV Tat-specific factor 1 [Amyelois transitella] |
| bmsk0013848 | 5.176666667 | | 1.823333333 | | -1.505445092 | serine/threonine-protein kinase Genghis Khan |
| bmsk0002474 | 32.88 | | 13.24333333 | | -1.311944006 | solute carrier organic anion transporter family member 2A1-like isoform X1 |
| bmsk0000095 | 0.71 | | 0.186666667 | | -1.927354698 | sex-linked proline rich protein, partial |
| bmsk0000119 | 11.18 | | 4.206666667 | | -1.410170779 | uncharacterized protein LOC105392398 [Plutella xylostella] |
| bmsk0010014 | 0.001 | | 0.086666667 | | 6.437405312 | ommochrome-binding protein |
| bmsk0004383 | 1.816666667 | | 0.523333333 | | -1.795491671 | LDLR chaperone boca |
| mstrg.18240 | 3.186666667 | | 0.946666667 | | -1.751119688 | PREDICTED: DNA replication licensing factor Mcm5-like [Plutella xylostella] |
| bmsk0008411 | 47.94666667 | | 18.72333333 | | -1.356593218 | D -directed R polymerase II subunit RPB9 |
| bmsk0001153 | 4.186666667 | | 1.2 | | -1.802767653 | F-box/LRR-repeat protein 15 |
| bmsk0005608 | 0.673333333 | | 0.116666667 | | -2.528928466 | preimplantation protein |
| bmsk0002749 | 0.001 | | 0.11 | | 6.781359714 | serine proteinase stubble-like [Amyelois transitella] |
| bmsk0005440 | 0.03 | | 0.33 | | 3.459431619 | uncharacterized protein LOC101740020 |
| bmsk0009584 | 0.526666667 | | 0.093333333 | | -2.496425826 | cell cycle checkpoint protein RAD1 [Amyelois transitella] |
| bmsk0005630 | 0.253333333 | | 0.001 | | -7.984893108 | protein phosphatase methylesterase 1 isoform X4 |
| bmsk0004556 | 0.066666667 | | 0.366666667 | | 2.459431619 | nuclear protein 1 |
| bmsk0009546 | 0.303333333 | | 0.056666667 | | -2.420331799 | - |
| bmsk0000339 | 0.106666667 | | 0.001 | | -6.736965594 | uncharacterized protein LOC101743995 |
| bmsk0012269 | 1.08 | | 0.06 | | -4.169925001 | XP 021206731.1 uncharacterized protein LOC105842286 |
| bmsk0014325 | 3.656666667 | | 1.083333333 | | -1.755051902 | - |
| bmsk0013429 | 0.001 | | 0.04 | | 5.321928095 | uncharacterized protein LOC106136069 isoform X2 [Amyelois transitella] |
| bmsk0008974 | 1.336666667 | | 0.07 | | -4.255141004 | putative cAMP-dependent protein kinase inhibitor beta [Danaus plexippus] |
| bmsk0004113 | 0.001 | | 0.06 | | 5.906890596 | Chymotrypsinogen B [Operophtera brumata] |
| bmsk0007335 | 0.11 | | 0.49 | | 2.155278225 | D -binding protein D-ETS-6 isoform X1 |
| bmsk0011103 | 20.36333333 | | 7.853333333 | | -1.374596701 | negative elongation factor B [Amyelois transitella] |
| bmsk0015384 | 0.616666667 | | 6.87 | | 3.477747329 | uncharacterized protein LOC105390186 [Plutella xylostella] |
| bmsk0010872 | 0.006666667 | | 0.136666667 | | 4.357552005 | XP 004933976.1 pupal cuticle protein 27 |
| bmsk0006018 | 0.346666667 | | 0.023333333 | | -3.893084796 | zinc finger protein 16-like isoform X2 |
| bmsk0006343 | 1.316666667 | | 0.143333333 | | -3.199444088 | - |
| bmsk0009796 | 0.293333333 | | 0.053333333 | | -2.459431619 | ATP-dependent D helicase Q1-like [Amyelois transitella] |
| bmsk0007785 | 1.57 | | 0.016666667 | | -6.557655155 | - |
| bmsk0014255 | 21.82333333 | | 7.263333333 | | -1.587167782 | XP 004923124.1 uncharacterized protein LOC101737900 |
| bmsk0014221 | 3.7 | | 0.906666667 | | -2.02888112 | ribosomal protein S6 kinase alpha-4-like isoform X3 [Papilio machaon] |
| bmsk0013696 | 0.001 | | 0.083333333 | | 6.380821784 | XP 021203537.1 fatty acyl-CoA reductase wat-like isoform X2 |
| bmsk0012747 | 0.18 | | 0.001 | | -7.491853096 | KWMTBOMO13160 |
| bmsk0002630 | 3.903333333 | | 1.15 | | -1.763072809 | metallo-beta-lactamase domain-containing protein 1 |
| bmsk0000633 | 0.456666667 | | 0.003333333 | | -7.098032083 | uncharacterized protein LOC105842554 |
| bmsk0005993 | 1.53 | | 0.463333333 | | -1.723409271 | zinc finger protein 624-like isoform X1 [Amyelois transitella] |
| bmsk0005978 | 0.246666667 | | 0.001 | | -7.94641896 | uncharacterized protein LOC106136821 [Amyelois transitella] |
| bmsk0006489 | 2.076666667 | | 0.586666667 | | -1.823656734 | lysophospholipase |
| bmsk0011813 | 0.426666667 | | 0.013333333 | | -5 | AAU12844.1 vitellogenin receptor, partial |
| bmsk0006912 | 0.033333333 | | 0.173333333 | | 2.378511623 | endoplasmic reticulum lectin 1 isoform X1 [Amyelois transitella] |
| bmsk0004288 | 0.033333333 | | 0.386666667 | | 3.5360529 | - |
| bmsk0002135 | 0.076666667 | | 0.001 | | -6.26052755 | hydrocephalus-inducing protein homolog |
| bmsk0011966 | 0.156666667 | | 0.43 | | 1.456638404 | - |
| bmsk0008259 | 0.083333333 | | 0.001 | | -6.380821784 | translation initiation factor 1a |
| bmsk0015716 | 0.233333333 | | 0.001 | | -7.866248611 | E1-E2 ATPase |
| bmsk0002573 | 10.53666667 | | 3.023333333 | | -1.801206579 | microspherule protein 1 [Amyelois transitella] |
| bmsk0013846 | 0.001 | | 0.16 | | 7.321928095 | orthodenticle |
| bmsk0003917 | 0.22 | | 0.573333333 | | 1.381870635 | putative transcription factor capicua |
| bmsk0002426 | 0.096666667 | | 0.01 | | -3.273018494 | UPF0553 protein C9orf64 homolog [Papilio machaon] |
| bmsk0014332 | 0.06 | | 0.433333333 | | 2.852442812 | AAD10243.1 cytochrome oxidase subunit I, partial (mitochondrion) |
| bmsk0001859 | 32.84666667 | | 13.09666667 | | -1.326547304 | cyclin-Y-like protein 1 [Amyelois transitella] |
| bmsk0013647 | 0.001 | | 1.333333333 | | 10.38082178 | nitrilase homolog 1 isoform X2 |
| bmsk0009574 | 0.001 | | 0.276666667 | | 8.112005026 | CRAL/TRIO domain |
| bmsk0004509 | 4.093333333 | | 0.183333333 | | -4.480735132 | - |
| bmsk0006662 | 0.001 | | 0.783333333 | | 9.613482541 | E3 ubiquitin-protein ligase MARCH6 |
| bmsk0003985 | 0.723333333 | | 2.303333333 | | 1.670990668 | Glutathione S-transferase, N-terminal domain |
| bmsk0014328 | 2.046666667 | | 0.76 | | -1.429204831 | - |
| mstrg.983 | 16.30666667 | | 5.653333333 | | -1.528288234 | PREDICTED: E3 ubiquitin-protein ligase UHRF1-like isoform X1 [Bombyx mori] |
| bmsk0014709 | 17.25333333 | | 6.266666667 | | -1.461104955 | - |
| bmsk0015545 | 4.226666667 | | 0.176666667 | | -4.580418576 | HMG (high mobility group) box |
| bmsk0011593 | 17.48 | | 6.906666667 | | -1.339643683 | tetratricopeptide repeat protein 4 [Papilio machaon] |
| bmsk0015736 | 0.433333333 | | 0.013333333 | | -5.022367813 | uncharacterized protein LOC105842017 |
| bmsk0004656 | 1.743333333 | | 0.346666667 | | -2.330227418 | uncharacterized protein LOC105843021 |
| bmsk0002415 | 0.001 | | 0.126666667 | | 6.984893108 | uncharacterized protein LOC101745524 isoform X1 |
| bmsk0012933 | 6.32 | | 0.07 | | -6.496425826 | kinesin-like protein KIF18A isoform X1 |
| bmsk0015522 | 8.07 | | 2.846666667 | | -1.503295104 | hypothetical protein RR46 01267 [Papilio xuthus] |
| bmsk0000782 | 0.46 | | 0.001 | | -8.845490051 | uncharacterized protein LOC101744603 |
| bmsk0010812 | 3.386666667 | | 0.706666667 | | -2.260764232 | insulin-like peptide receptor [Papilio polytes] |
| bmsk0000109 | 0.356666667 | | 0.001 | | -8.478432581 | abnormal chemosensory jump 6, isoform D |
| bmsk0014111 | 15.43666667 | | 5.783333333 | | -1.416388094 | cleavage stimulation factor subunit 1 [Papilio machaon] |
| bmsk0012898 | 0.196666667 | | 0.001 | | -7.619608644 | uncharacterized protein LOC101739117 |
| bmsk0011517 | 0.3 | | 0.001 | | -8.22881869 | low molecular 30 kDa lipoprotein PBMHPC-23 precursor |
| bmsk0005285 | 7.06 | | 2.61 | | -1.435618377 | sesquipedalian-1 [Papilio machaon] |
| bmsk0015864 | 0.996666667 | | 0.19 | | -2.39111166 | lysozyme-like |
| bmsk0006609 | 7.906666667 | | 2.946666667 | | -1.423985735 | glucose-fructose oxidoreductase domain-containing protein 1 [Amyelois transitella] |
| mstrg.11908 | 87.12 | | 35.69 | | -1.287484053 | RNA binding motif protein Y14 [Bombyx mori] |
| bmsk0010890 | 0.636666667 | | 0.043333333 | | -3.87698911 | XP 004926205.1 transmembrane protein 209 |
| bmsk0001586 | 1.433333333 | | 0.28 | | -2.355875427 | pericentrin |
| bmsk0011302 | 0.123333333 | | 0.016666667 | | -2.887525271 | uncharacterized protein LOC106137262 [Amyelois transitella] |
| bmsk0000307 | 1.023333333 | | 0.25 | | -2.033276155 | uncharacterized protein LOC101738134 isoform X1 |
| bmsk0006207 | 5.293333333 | | 1.97 | | -1.425980877 | uncharacterized protein C7orf26 homolog |
| bmsk0004585 | 12.21333333 | | 4.3 | | -1.506048438 | aryl-hydrocarbon-interacting protein-like 1 |
| bmsk0004996 | 0.42 | | 0.066666667 | | -2.655351829 | poly(rC)-binding protein 3 isoform X1 |
| bmsk0014519 | 0.013333333 | | 0.07 | | 2.392317423 | AMP deaminase 2-like [Amyelois transitella] |
| bmsk0008753 | 30.10666667 | | 12.02333333 | | -1.324246063 | nuclear cap-binding protein subunit 2 |
| bmsk0000023 | 0.013333333 | | 0.16 | | 3.584962501 | uncharacterized protein LOC110386361 |
| bmsk0007385 | 0.03 | | 0.001 | | -4.906890596 | uncharacterized protein LOC105841658 isoform X1 |
| bmsk0007396 | 0.01 | | 0.15 | | 3.906890596 | organic cation transporter protein-like isoform X3 |
| bmsk0013489 | 4.096666667 | | 1.586666667 | | -1.368451437 | WD repeat-containing protein 6 [Amyelois transitella] |
| bmsk0001880 | 0.65 | | 2.256666667 | | 1.79568171 | protein muscleblind isoform X6 |
| bmsk0008572 | 0.001 | | 0.166666667 | | 7.380821784 | uncharacterized protein LOC106719573 [Papilio machaon] |
| bmsk0000294 | 7.616666667 | | 2.58 | | -1.561788694 | uncharacterized protein LOC106126268 [Papilio xuthus] |
| bmsk0009778 | 1.013333333 | | 3.29 | | 1.698978761 | Glycosyl hydrolases family 18 |
| bmsk0003449 | 20.44333333 | | 7.096666667 | | -1.526417001 | putative peptidyl-tR hydrolase PTRHD1 |
| bmsk0003393 | 12.66333333 | | 2.753333333 | | -2.201406025 | Leucine rich repeat |
| bmsk0008543 | 81.06666667 | | 33.69 | | -1.266788394 | uncharacterized protein LOC105841250 |
| bmsk0009892 | 0.19 | | 0.446666667 | | 1.233199176 | Baculovirus FP protein |
| bmsk0004034 | 27.61 | | 10.4 | | -1.40860736 | uncharacterized protein LOC106134428 isoform X2 [Amyelois transitella] |
| bmsk0009343 | 0.133333333 | | 0.586666667 | | 2.137503524 | - |
| bmsk0011612 | 12.23333333 | | 4.776666667 | | -1.356741453 | zinc finger and BTB domain-containing protein 24-like |
| bmsk0010097 | 0.286666667 | | 0.001 | | -8.163230349 | uncharacterized protein LOC101744784 |
| bmsk0013188 | 0.456666667 | | 0.046666667 | | -3.290677161 | lysine-specific histone demethylase 1A |
| bmsk0000084 | 4.343333333 | | 1.653333333 | | -1.393425058 | quinone oxidoreductase isoform X1 |
| bmsk0009493 | 0.001 | | 0.186666667 | | 7.544320516 | zinc finger protein 84 isoform X1 |
| bmsk0008840 | 4.25 | | 1.113333333 | | -1.932577239 | ribonuclease H2 subunit B [Papilio xuthus] |
| bmsk0006314 | 0.61 | | 2.083333333 | | 1.772012541 | uncharacterized protein LOC106121342 [Papilio xuthus] |
| bmsk0001757 | 0.023333333 | | 0.296666667 | | 3.668378509 | - |
| bmsk0012773 | 14.24666667 | | 5.86 | | -1.281651838 | rapamycin-insensitive companion of mTOR |
| bmsk0003900 | 0.436666667 | | 0.001 | | -8.770388596 | - |
| bmsk0006442 | 2.023333333 | | 0.586666667 | | -1.786121088 | F-box/LRR-repeat protein 16 |
| bmsk0014931 | 2.58 | | 0.69 | | -1.902702799 | XP 021202506.1 homeobox protein prospero isoform X1 |
| bmsk0014638 | 12.59333333 | | 4.843333333 | | -1.378587999 | AET05794.1 ubiquitin-conjugating enzyme E2 J1-like protein |
| bmsk0005932 | 20.78 | | 7.696666667 | | -1.432889982 | S-phase kinase-associated protein 2 |
| bmsk0003220 | 0.001 | | 0.106666667 | | 6.736965594 | cytochrome b5-like |
| bmsk0004576 | 24.38 | | 9.883333333 | | -1.302628522 | target of rapamycin complex subunit lst8 isoform X1 |
| bmsk0005654 | 0.066666667 | | 0.001 | | -6.058893689 | DEP domain-containing protein 5 |
| bmsk0014933 | 3.303333333 | | 1.053333333 | | -1.648960499 | BAH83641.1 prospero, partial |
| bmsk0011525 | 0.833333333 | | 0.016666667 | | -5.64385619 | ABC-type uncharacterized transport system |
| bmsk0011012 | 2.803333333 | | 0.001 | | -11.45292758 | polynucleotide 5'-hydroxyl-kinase NOL9 [Amyelois transitella] |
| bmsk0004452 | 27.63666667 | | 10.89 | | -1.343579665 | trafficking protein particle complex subunit 13-like [Amyelois transitella] |
| bmsk0000421 | 0.11 | | 0.001 | | -6.781359714 | uncharacterized protein LOC101736416 isoform X1 |
| bmsk0014746 | 32.98333333 | | 13.98333333 | | -1.238028897 | pre-mR -processing factor 39 isoform X1 [Amyelois transitella] |
| bmsk0006868 | 0.001 | | 0.14 | | 7.129283017 | putative fatty acyl-CoA reductase CG5065 isoform X2 |
| bmsk0003231 | 1.87 | | 0.506666667 | | -1.883929447 | DET1- and DDB1-associated protein 1 [Amyelois transitella] |
| bmsk0008919 | 13.17666667 | | 4.753333333 | | -1.470973973 | mR cap guanine-N7 methyltransferase [Papilio machaon] |
| bmsk0005097 | 0.263333333 | | 0.593333333 | | 1.171952683 | - |
| bmsk0008662 | 0.07 | | 0.226666667 | | 1.695145418 | potassium voltage-gated channel subfamily H member 6-like isoform X5 |
| bmsk0014752 | 0.713333333 | | 0.106666667 | | -2.741466986 | - |
| bmsk0010308 | 0.001 | | 0.066666667 | | 6.058893689 | major royal jelly protein 1-like |
| bmsk0007797 | 2.653333333 | | 0.713333333 | | -1.895157634 | - |
| bmsk0002479 | 0.046666667 | | 0.383333333 | | 3.038135129 | uncharacterized protein LOC101745841 |
| mstrg.5081 | 17.75333333 | | 6.193333333 | | -1.519301927 | PREDICTED: FAST kinase domain-containing protein 5 [Bombyx mori] |
| bmsk0004963 | 0.38 | | 0.006666667 | | -5.832890014 | opioid-binding protein/cell adhesion molecule homolog |
| bmsk0007098 | 0.026666667 | | 0.233333333 | | 3.129283017 | cuticular protein glycine-rich 15 precursor |
| bmsk0007132 | 25.01 | | 9.55 | | -1.388932419 | TATA-box-binding protein |
| bmsk0014364 | 0.026666667 | | 0.07 | | 1.392317423 | uncharacterized protein LOC101737485 isoform X2 |
| bmsk0008798 | 0.026666667 | | 0.183333333 | | 2.781359714 | Sulfatase |
| bmsk0008068 | 0.233333333 | | 0.001 | | -7.866248611 | paired box protein Pax-6-like |
| bmsk0001549 | 0.613333333 | | 0.15 | | -2.03170886 | N6-adenosine-methyltransferase subunit METTL14 [Amyelois transitella] |
| bmsk0015901 | 0.143333333 | | 0.023333333 | | -2.618909833 | - |
| bmsk0011070 | 7.163333333 | | 2.45 | | -1.547849328 | - |
| bmsk0014353 | 0.493333333 | | 0.001 | | -8.94641896 | XP 004926504.2 homeobox protein Hox-B5a |
| bmsk0013099 | 0.993333333 | | 0.133333333 | | -2.897240426 | XP 004925483.1 probable cysteine--tR ligase, mitochondrial |
| bmsk0007542 | 3.15 | | 0.143333333 | | -4.457905764 | neurotactin isoform X1 |
| bmsk0002542 | 0.28 | | 0.001 | | -8.129283017 | Prolyl oligopeptidase, N-terminal beta-propeller domain |
| mstrg.19860 | 6.506666667 | | 2.393333333 | | -1.442897304 | juvenile hormone esterase binding protein [Bombyx mori] |
| bmsk0001428 | 16.27 | | 5.823333333 | | -1.482297143 | LSM Sm-like protein family member |
| bmsk0014466 | 14.49666667 | | 4.916666667 | | -1.559968754 | XP 021202958.1 transmembrane emp24 domain-containing protein 5 isoform X2 |
| bmsk0004499 | 0.15 | | 0.001 | | -7.22881869 | methyltransferase-like protein 16 [Papilio xuthus] |
| bmsk0006627 | 1.036666667 | | 0.283333333 | | -1.871379834 | neuropeptide receptor A10 |
| bmsk0004177 | 0.51 | | 0.04 | | -3.672425342 | metabotropic glutamate receptor 2-like [Papilio xuthus] |
| bmsk0007190 | 0.05 | | 0.236666667 | | 2.242856524 | cuticular protein RR-2 motif 139 precursor |
| bmsk0002836 | 0.001 | | 0.793333333 | | 9.631783357 | hypothetical protein RR46 11993 [Papilio xuthus] |
| bmsk0013797 | 1.27 | | 0.13 | | -3.288244969 | XP 021202005.1 uncharacterized protein LOC101744639 isoform X1 |
| bmsk0002972 | 0.003333333 | | 0.533333333 | | 7.321928095 | atlastin, partial |
| bmsk0007121 | 13.17333333 | | 4.72 | | -1.480761682 | transcription initiation factor TFIID subunit 7-like |
| bmsk0003064 | 0.263333333 | | 0.001 | | -8.040746342 | transmembrane protein 205 |
| bmsk0009240 | 0.013333333 | | 0.16 | | 3.584962501 | uncharacterized protein LOC101744375 isoform X2 |
| bmsk0014936 | 0.416666667 | | 0.026666667 | | -3.965784285 | - |
| bmsk0002763 | 5.746666667 | | 1.893333333 | | -1.60179694 | methyltransferase-like 26 |
| bmsk0015801 | 0.001 | | 0.066666667 | | 6.058893689 | gag-polypeptide of LTR copia-type |
| bmsk0004044 | 1.03 | | 0.163333333 | | -2.656753184 | uncharacterized protein LOC105841580 |
| bmsk0013590 | 0.001 | | 0.49 | | 8.936637939 | esterase FE4-like isoform X1 |
| bmsk0010086 | 3.946666667 | | 1.093333333 | | -1.851901361 | DWNN domain |
| bmsk0010341 | 0.306666667 | | 0.001 | | -8.26052755 | Zinc knuckle |
| bmsk0015962 | 1.083333333 | | 0.263333333 | | -2.04051516 | XP 004923468.1 armadillo repeat-containing protein 6 homolog |
| bmsk0003045 | 0.663333333 | | 0.046666667 | | -3.829269698 | Neurotransmitter-gated ion-channel transmembrane region |
| bmsk0008721 | 0.456666667 | | 0.123333333 | | -1.888578717 | beta-1,3-galactosyltransferase 6 isoform X2 |
| bmsk0001057 | 0.276666667 | | 0.026666667 | | -3.375039431 | uncharacterized protein LOC101736094 isoform X1 |
| bmsk0012136 | 0.006666667 | | 0.06 | | 3.169925001 | - |
| bmsk0007266 | 0.001 | | 0.313333333 | | 8.291554446 | - |
| bmsk0009346 | 0.236666667 | | 0.001 | | -7.886712714 | - |
| bmsk0005552 | 0.046666667 | | 0.266666667 | | 2.514573173 | sodium- and chloride-dependent GABA transporter ine |
| bmsk0002872 | 16.05333333 | | 5.976666667 | | -1.425459904 | 6-pyruvoyltetrahydropterin synthase |
| bmsk0003062 | 0.473333333 | | 0.103333333 | | -2.195550809 | kinesin-like protein a isoform X2 |
| bmsk0011115 | 2.046666667 | | 0.346666667 | | -2.561655127 | carbohydrate sulfotransferase 11 isoform X2 |
| bmsk0003095 | 0.026666667 | | 0.18 | | 2.754887502 | uncharacterized protein LOC101743935 isoform X1 |
| bmsk0009600 | 7.55 | | 2.836666667 | | -1.412280013 | CCR4-NOT transcription complex subunit 10-like [Amyelois transitella] |
| bmsk0007445 | 0.253333333 | | 0.013333333 | | -4.247927513 | TRAF6 |
| bmsk0015710 | 0.903333333 | | 0.206666667 | | -2.127952731 | tigger transposable element-derived protein 4-like [Papilio machaon] |
| bmsk0002164 | 0.053333333 | | 0.376666667 | | 2.820178962 | whirlin |
| bmsk0012818 | 0.03 | | 0.15 | | 2.321928095 | XP 021204231.1 serine protease inhibitor 28 isoform X2 |
| bmsk0007581 | 8.003333333 | | 2.99 | | -1.420455513 | DEAD-box helicase Dbp80 |
| bmsk0003331 | 0.39 | | 0.013333333 | | -4.87036472 | transcription factor deformed |
| bmsk0008338 | 2.646666667 | | 0.966666667 | | -1.453086107 | - |
| bmsk0006019 | 0.006666667 | | 0.146666667 | | 4.459431619 | uncharacterized protein LOC101741752 isoform X5 |
| bmsk0002884 | 0.65 | | 0.001 | | -9.344295908 | probable lysine-specific demethylase ELF6 [Papilio xuthus] |
| bmsk0013941 | 0.583333333 | | 0.001 | | -9.188176706 | uncharacterized protein LOC105387121 [Plutella xylostella] |
| bmsk0001832 | 0.39 | | 0.016666667 | | -4.548436625 | - |
| bmsk0001073 | 0.423333333 | | 0.023333333 | | -4.181329765 | Zinc finger D binding protein [Operophtera brumata] |
| bmsk0003811 | 9.28 | | 3.37 | | -1.461376214 | golgin subfamily A member 5 [Amyelois transitella] |
| bmsk0000358 | 0.013333333 | | 0.21 | | 3.977279923 | tyrosine-protein kinase receptor TYRO3-like [Papilio polytes] |
| bmsk0002390 | 10.58333333 | | 3.806666667 | | -1.475193941 | Transcription factor 2B [Operophtera brumata] |
| bmsk0003712 | 0.253333333 | | 0.713333333 | | 1.493539473 | phosphatidylinositol-binding clathrin assembly protein LAP isoform X2 |
| bmsk0006922 | 0.001 | | 0.856666667 | | 9.742590143 | nose resistant to fluoxetine protein 6-like |
| bmsk0009613 | 0.006666667 | | 0.083333333 | | 3.64385619 | gustatory receptor 9 |
| bmsk0007446 | 0.343333333 | | 0.003333333 | | -6.686500527 | uncharacterized protein LOC106134578 [Amyelois transitella] |
| bmsk0005325 | 1.906666667 | | 0.276666667 | | -2.784831905 | uncharacterized protein LOC101741169 isoform X5 |
| bmsk0002045 | 0.026666667 | | 0.273333333 | | 3.357552005 | - |
| bmsk0000061 | 0.493333333 | | 1.02 | | 1.047934477 | - |
| mstrg.22258 | 8.513333333 | | 3.296666667 | | -1.368716099 | PREDICTED: uncharacterized protein LOC101747074 isoform X2 [Bombyx mori] |
| bmsk0014312 | 0.001 | | 0.173333333 | | 7.437405312 | XP 012546088.1 uncharacterized protein LOC101743782 |
| bmsk0001752 | 2.026666667 | | 0.336666667 | | -2.589716031 | uncharacterized protein LOC105841817 |
| bmsk0014239 | 0.146666667 | | 0.003333333 | | -5.459431619 | - |
| bmsk0014205 | 0.066666667 | | 0.001 | | -6.058893689 | - |
| bmsk0006022 | 0.593333333 | | 0.023333333 | | -4.668378509 | - |
| bmsk0002913 | 0.083333333 | | 1.066666667 | | 3.678071905 | monocarboxylate transporter 1 isoform X1 |
| bmsk0011290 | 23.71 | | 10.06666667 | | -1.235909614 | tumor susceptibility gene 101 protein [Amyelois transitella] |
| bmsk0009362 | 1.423333333 | | 0.393333333 | | -1.85544921 | nardilysin-like |
| bmsk0008803 | 0.073333333 | | 0.001 | | -6.196397213 | uncharacterized protein DDB G0284459-like isoform X1 |
| bmsk0013533 | 68.31 | | 27.88333333 | | -1.292693753 | enhancer of rudimentary homolog [Plutella xylostella] |
| bmsk0011710 | 8.683333333 | | 3.5 | | -1.310894045 | XP 004933640.1 G patch domain-containing protein 1 homolog |
| bmsk0000125 | 0.496666667 | | 0.001 | | -8.956134115 | apterous A isoform X2 |
| bmsk0003908 | 0.001 | | 0.04 | | 5.321928095 | hemocyte protease-1 |
| bmsk0012761 | 0.023333333 | | 0.12 | | 2.362570079 | probable cation-transporting ATPase 13A3 |
| bmsk0004800 | 0.31 | | 0.063333333 | | -2.291231298 | tigger transposable element-derived protein 4-like [Papilio machaon] |
| bmsk0014447 | 2.86 | | 0.243333333 | | -3.555009279 | Ankyrin repeats (many copies) |
| bmsk0000853 | 15.18333333 | | 5.45 | | -1.478160418 | protein kinase c inhibitor isoform X1 |
| bmsk0013507 | 0.003333333 | | 0.126666667 | | 5.247927513 | uncharacterized protein LOC106116608 isoform X2 [Papilio xuthus] |
| bmsk0011907 | 0.51 | | 0.04 | | -3.672425342 | protein CBFA2T1 |
| bmsk0009940 | 1.11 | | 0.153333333 | | -2.855816411 | craniofacial development protein 2-like |
| bmsk0000652 | 0.203333333 | | 0.82 | | 2.011777168 | Domain of unknown function (DUF4780) |
| bmsk0000654 | 0.203333333 | | 0.82 | | 2.011777168 | Domain of unknown function (DUF4780) |
| bmsk0000315 | 0.523333333 | | 0.15 | | -1.802767653 | - |
| bmsk0004518 | 11.79 | | 4.143333333 | | -1.508699923 | PRKR-interacting protein 1 homolog |
| bmsk0010313 | 0.001 | | 0.03 | | 4.906890596 | XP 004926673.1 inactive pancreatic lipase-related protein 1 |
| bmsk0006208 | 5.686666667 | | 1.996666667 | | -1.509989739 | cleavage and polyadenylation specific factor 4 |
| bmsk0004102 | 0.143333333 | | 0.513333333 | | 1.840521786 | adipokinetic prohormone precursor type II |
| bmsk0011363 | 0.213333333 | | 0.001 | | -7.736965594 | - |
| bmsk0010760 | 0.076666667 | | 0.253333333 | | 1.724365557 | XP 021204965.1 uncharacterized protein LOC101736278 isoform X1 |
| bmsk0008800 | 0.186666667 | | 0.001 | | -7.544320516 | - |
| bmsk0000495 | 0.001 | | 0.126666667 | | 6.984893108 | uncharacterized protein LOC101742079 isoform X2 |
| bmsk0012623 | 0.001 | | 0.056666667 | | 5.824428435 | cuticular protein RR-2 motif 86 precursor |
| bmsk0001023 | 0.001 | | 0.643333333 | | 9.329422631 | Cysteine dioxygenase type I |
| bmsk0010566 | 1.156666667 | | 0.376666667 | | -1.61861289 | REST corepressor isoform X3 [Papilio xuthus] |
| bmsk0005185 | 0.026666667 | | 0.156666667 | | 2.554588852 | - |
| bmsk0011211 | 3.27 | | 1.133333333 | | -1.52871839 | X-ray repair cross-complementing protein 5 [Operophtera brumata] |
| bmsk0005492 | 5.326666667 | | 1.543333333 | | -1.78718331 | - |
| bmsk0007499 | 0.603333333 | | 0.001 | | -9.236811481 | transcriptional adapter 1-like [Amyelois transitella] |
| bmsk0010207 | 13.07666667 | | 4.876666667 | | -1.423027566 | carboxyl terminus of heat shock cognate70 interacting protein |
| bmsk0011138 | 0.743333333 | | 0.26 | | -1.515497681 | serine/threonine-protein kinase PRP4 homolog |
| bmsk0011771 | 1.376666667 | | 0.173333333 | | -2.989558253 | uncharacterized protein LOC105841875 isoform X1 |
| bmsk0007533 | 0.173333333 | | 0.01 | | -4.115477217 | filamin-A-interacting protein 1-like [Amyelois transitella] |
| bmsk0000296 | 0.25 | | 0.763333333 | | 1.610385098 | proton-coupled folate transporter isoform X2 |
| mstrg.10077 | 0.086666667 | | 0.276666667 | | 1.674599713 | PREDICTED: odorant-binding protein 4 isoform X1 [Bombyx mori] |
| bmsk0003979 | 0.006666667 | | 0.393333333 | | 5.882643049 | UDP-glucuronosyltransferase 2B1 |
| **Differentially expressed genes of silkworm between 5-day embryos and 10-day larvae during *N. bombycis* infection** | | | | | | |
| Id | I-E5_mean | | I-L10_mean | | log2(fc) | Description |
| bmsk0013768 | 5.146666667 | | 42.83666667 | | 3.057135969 | Tetraspanin [Operophtera brumata] |
| bmsk0013118 | 23.78 | | 52.89333333 | | 1.153337182 | XP 021201818.1 axoneme-associated protein mst101(2) isoform X1 |
| bmsk0015665 | 5.863333333 | | 35.34666667 | | 2.591781187 | KWMTBOMO16085 |
| bmsk0002578 | 43.25 | | 125.9366667 | | 1.541926349 | hypothetical protein OBRU01 00840 [Operophtera brumata] |
| bmsk0012200 | 6.666666667 | | 13.84 | | 1.053806444 | Ubiquitin family |
| bmsk0005753 | 72.46666667 | | 173.11 | | 1.256299627 | X-box-binding protein 1 |
| bmsk0015666 | 72.85666667 | | 554.9933333 | | 2.929337546 | protein lethal(2)essential for life-like [Amyelois transitella] |
| bmsk0014513 | 9.28 | | 18.76666667 | | 1.015975711 | XP 004925716.1 atlastin |
| bmsk0007849 | 0.703333333 | | 11.96666667 | | 4.08866894 | uncharacterized protein LOC106131201 [Amyelois transitella] |
| bmsk0005202 | 0.063333333 | | 21.61333333 | | 8.414740862 | putative cuticle protein |
| bmsk0015991 | 4.263333333 | | 13.08333333 | | 1.61767639 | serpin 5 [Bombyx mandarina] |
| bmsk0015992 | 4.263333333 | | 13.08333333 | | 1.61767639 | - |
| bmsk0014899 | 0.64 | | 4.99 | | 2.962896005 | papilin |
| bmsk0004872 | 45.46666667 | | 133.9866667 | | 1.559208298 | uncharacterized protein LOC101735549 isoform X3 |
| bmsk0001286 | 1.556666667 | | 8.456666667 | | 2.441629064 | protein spaetzle 3 |
| bmsk0002517 | 584.24 | | 1606.68 | | 1.459449577 | 32 kDa apolipoprotein precursor |
| mstrg.4923 | 1.906666667 | | 4.953333333 | | 1.377347064 | PREDICTED: lipopolysaccharide-induced tumor necrosis factor-alpha factor homolog [Bombyx mori] |
| bmsk0009266 | 0.966666667 | | 5.083333333 | | 2.394684437 | uncharacterized protein LOC101742743 |
| mstrg.10278 | 3.536666667 | | 8.69 | | 1.296965927 | PREDICTED: fasciclin-2-like [Bombyx mori] |
| bmsk0004805 | 57.49666667 | | 119.4233333 | | 1.054534519 | 5-aminolevulinate synthase, nonspecific, mitochondrial |
| bmsk0001933 | 67.13333333 | | 288.98 | | 2.105868466 | uncharacterized protein LOC106143014 [Amyelois transitella] |
| bmsk0003664 | 2.36 | | 25.89333333 | | 3.455721935 | uncharacterized protein LOC105842978 |
| bmsk0003071 | 49.28333333 | | 249.4166667 | | 2.33938613 | protein lethal(2)essential for life-like [Amyelois transitella] |
| bmsk0013357 | 18.46 | | 57.25666667 | | 1.63304113 | kazal-type proteinase inhibitor precursor |
| bmsk0003658 | 1.7 | | 18.78333333 | | 3.465846458 | uncharacterized protein LOC105842978 |
| bmsk0002005 | 1.816666667 | | 5.79 | | 1.672269619 | tensin isoform X4 |
| bmsk0015670 | 37.87666667 | | 317.2066667 | | 3.066041815 | heat shock protein 70 |
| bmsk0004553 | 0.826666667 | | 2.096666667 | | 1.342719896 | gamma-glutamyltranspeptidase 1 isoform X2 |
| mstrg.4922 | 1.556666667 | | 3.5 | | 1.168894873 | olfactory receptor [Bombyx mori] |
| bmsk0007085 | 2.376666667 | | 5.456666667 | | 1.19908034 | Alcohol dehydrogenase transcription factor Myb/SANT-like |
| bmsk0007086 | 2.376666667 | | 5.456666667 | | 1.19908034 | uridine phosphorylase 1 isoform X1 |
| bmsk0010120 | 114.4866667 | | 234.0033333 | | 1.031349492 | cathepsin B precursor |
| bmsk0012604 | 0.393333333 | | 22.10333333 | | 5.81236769 | putative cuticle protein |
| bmsk0008490 | 15.62666667 | | 113.0133333 | | 2.854411018 | gasp precursor |
| bmsk0013426 | 17.11 | | 35.61 | | 1.057442676 | 60S ribosomal protein L38 |
| bmsk0001934 | 0.556666667 | | 2.79 | | 2.32537952 | very low-density lipoprotein receptor isoform 1 precursor |
| bmsk0005576 | 15.28333333 | | 38.59666667 | | 1.336517024 | beta-galactosidase |
| bmsk0013066 | 0.033333333 | | 124.4333333 | | 11.86611979 | ecdysteroid regulated protein [Danaus plexippus] |
| mstrg.22442 | 34.13333333 | | 71.30666667 | | 1.062855655 | Rab-related protein [Bombyx mori] |
| bmsk0011093 | 73.97 | | 164.7633333 | | 1.155383038 | peroxisomal multifunctional enzyme type 2-like [Papilio xuthus] |
| bmsk0002545 | 37.55333333 | | 83.82 | | 1.158353551 | adenylate kinase 2 |
| bmsk0012298 | 569.58 | | 1193.256667 | | 1.066934003 | ribosomal protein L36 |
| bmsk0006521 | 0.083333333 | | 70.15 | | 9.717333699 | - |
| mstrg.4881 | 5.796666667 | | 18.05666667 | | 1.639236158 | PREDICTED: trimethyllysine dioxygenase, mitochondrial [Bombyx mori] |
| bmsk0007743 | 0.33 | | 4.953333333 | | 3.90786178 | yellow-y isoform X1 |
| bmsk0011897 | 61.72 | | 260.4366667 | | 2.077122612 | beta-tubulin |
| bmsk0001719 | 3.603333333 | | 11.82 | | 1.713826013 | Krueppel-like factor 10 |
| bmsk0005038 | 7.91 | | 16.35333333 | | 1.047835133 | phosphoserine aminotransferase |
| bmsk0015671 | 149.78 | | 1051.153333 | | 2.811056233 | heat shock protein 70 |
| bmsk0015756 | 7.393333333 | | 20.84333333 | | 1.495289152 | XP 004933525.1 growth arrest and D damage-inducible protein GADD45 alpha |
| bmsk0001550 | 0.663333333 | | 7.403333333 | | 3.480369057 | inducible metalloproteinase inhibitor protein-like |
| bmsk0006394 | 0.783333333 | | 2.163333333 | | 1.465557721 | BTB/POZ domain-containing protein 6-B |
| mstrg.11341 | 4.346666667 | | 13.60666667 | | 1.646332313 | PREDICTED: chemosensory protein 9 isoform X1 [Bombyx mori] |
| bmsk0000624 | 51.25666667 | | 108.2066667 | | 1.077977822 | uncharacterized protein LOC100302108 isoform X1 |
| bmsk0001822 | 44.79 | | 154.0866667 | | 1.782493458 | uricase |
| bmsk0005264 | 0.103333333 | | 12.71666667 | | 6.943271031 | cuticular protein glycine-rich 14 precursor |
| bmsk0005808 | 1315.186667 | | 2687.386667 | | 1.030936336 | ribosomal protein L10 |
| bmsk0002054 | 1.613333333 | | 3.236666667 | | 1.004464248 | probable nuclear hormone receptor HR38 |
| bmsk0011378 | 27.35333333 | | 3.333333333 | | -3.036679154 | putative odorant-degrading enzyme [Antheraea polyphemus] |
| bmsk0002666 | 36.22333333 | | 3.23 | | -3.487313242 | uncharacterized protein LOC101740936 |
| bmsk0006495 | 4.79 | | 57.21333333 | | 3.578253839 | uncharacterized protein LOC106109800 [Papilio polytes] |
| bmsk0009407 | 7.903333333 | | 25.58666667 | | 1.694859049 | fatty acid-binding protein, muscle-like [Plutella xylostella] |
| bmsk0014641 | 33.12333333 | | 67.74666667 | | 1.032302099 | electron-transfer-flavoprotein beta polypeptide |
| bmsk0006121 | 3.07 | | 7.41 | | 1.271234887 | probable R -binding protein orb2 isoform X2 |
| bmsk0015669 | 5.426666667 | | 34.19333333 | | 2.655576872 | heat shock protein 70 |
| bmsk0001285 | 2.08 | | 4.616666667 | | 1.150268042 | Spatzle 3 [Operophtera brumata] |
| bmsk0000230 | 0.54 | | 2.063333333 | | 1.933945596 | cyclic nucleotide-gated cation channel subunit A |
| bmsk0005627 | 0.49 | | 2.383333333 | | 2.282127087 | potassium voltage-gated channel subfamily H member 8 isoform X1 |
| bmsk0001925 | 89.5 | | 206.74 | | 1.207857961 | transport protein Sec61 beta subunit |
| bmsk0009124 | 12.09 | | 36.04333333 | | 1.575918195 | CoA binding domain |
| bmsk0013693 | 1.56 | | 7.523333333 | | 2.269825984 | fatty-acyl CoA reductase 2 [Ostrinia nubilalis] |
| bmsk0006832 | 1.556666667 | | 4.053333333 | | 1.380648774 | scavenger receptor class B member 4 |
| bmsk0013661 | 1.396666667 | | 7.186666667 | | 2.363335029 | scavenger receptor class B member 1-like [Amyelois transitella] |
| bmsk0009253 | 1.99 | | 6.166666667 | | 1.631722434 | uncharacterized protein LOC106129912 [Amyelois transitella] |
| bmsk0005455 | 0.86 | | 3.553333333 | | 2.046764467 | putative fatty acyl-CoA reductase CG5065 |
| bmsk0013856 | 0.936666667 | | 11.95666667 | | 3.67413571 | transcription factor SPT20 homolog [Papilio polytes] |
| bmsk0003379 | 14.91666667 | | 31.69333333 | | 1.087254221 | transmembrane emp24 domain-containing protein 3 |
| bmsk0013565 | 15.03333333 | | 30.79333333 | | 1.034453113 | sorting and assembly machinery component 50 homolog [Amyelois transitella] |
| mstrg.22714 | 15.59333333 | | 1.15 | | -3.761223595 | Myosin-J heavy chain [Operophtera brumata] |
| mstrg.5704 | 6.906666667 | | 15.78666667 | | 1.192645078 | PREDICTED: apoptosis-inducing factor 1, mitochondrial-like [Bombyx mori] |
| bmsk0008916 | 884.9366667 | | 1775.49 | | 1.004571122 | ribosomal protein P1 |
| bmsk0012452 | 9.98 | | 24.18 | | 1.276702524 | ADG85767.1 triose-phosphate transporter-like protein |
| bmsk0003025 | 2.1 | | 6.756666667 | | 1.685922355 | uncharacterized protein LOC101741240 isoform X2 |
| bmsk0009128 | 0.85 | | 2.343333333 | | 1.463027442 | uncharacterized protein LOC101739604 isoform X1 |
| bmsk0002838 | 0.626666667 | | 5.223333333 | | 3.059200613 | proline-rich receptor-like protein kinase PERK12 |
| bmsk0009580 | 3.183333333 | | 6.846666667 | | 1.104863543 | uncharacterized protein LOC105842714 |
| bmsk0008453 | 1381.936667 | | 2787.486667 | | 1.012273405 | ribosomal protein L7A |
| bmsk0013403 | 43.45 | | 94.13333333 | | 1.115349506 | transferrin precursor |
| bmsk0001318 | 14.58 | | 1.66 | | -3.134735573 | uncharacterized protein LOC101741830 isoform X1 |
| bmsk0015996 | 5.19 | | 11.03333333 | | 1.088062273 | elongation factor Tu-like [Amyelois transitella] |
| bmsk0003797 | 0.026666667 | | 6.9 | | 8.015415052 | uncharacterized protein LOC106707703 isoform X1 [Papilio machaon] |
| bmsk0005638 | 5.303333333 | | 25.50333333 | | 2.265714487 | cytochrome P450 |
| bmsk0014921 | 24.9 | | 54.93666667 | | 1.141623634 | bifunctional glutamate/proline--tR ligase isoform X1 |
| bmsk0008977 | 567.1133333 | | 1240.276667 | | 1.128952995 | ribosomal protein P0 |
| bmsk0001149 | 186.9866667 | | 418.85 | | 1.163498273 | 60 kDa heat shock protein, mitochondrial |
| bmsk0006278 | 78.34333333 | | 203.7466667 | | 1.378894039 | signal sequence receptor precursor |
| bmsk0011539 | 8.536666667 | | 26.34333333 | | 1.625693155 | hemolymph protein isoform X1 |
| bmsk0009475 | 22.81666667 | | 59.40333333 | | 1.380455847 | PRP2 BOMMO RecName: Full=Phenoloxidase subunit 2; AltName: Full=PO 2; AltName: Full=Tyrosinase 2; Flags: Precursor |
| bmsk0013576 | 0.846666667 | | 2.026666667 | | 1.259242827 | acetyl-CoA carboxylase isoform X1 |
| bmsk0002269 | 0.14 | | 8.21 | | 5.87388349 | cuticular protein RR-2 motif 60 precursor |
| bmsk0002387 | 92.88666667 | | 251.4266667 | | 1.436594246 | 19.5 kDa heat shock protein |
| bmsk0009509 | 26.12333333 | | 59.84666667 | | 1.195931898 | asparagine--tR ligase, cytoplasmic [Papilio xuthus] |
| bmsk0000504 | 0.706666667 | | 1.856666667 | | 1.393613063 | fibrinogen alpha chain [Papilio xuthus] |
| bmsk0012894 | 12.12 | | 1.833333333 | | -2.724848676 | XP 004924762.1 protein Spindly |
| bmsk0011954 | 3.823333333 | | 12.75 | | 1.737594356 | fibrillin-1-like |
| bmsk0012568 | 0.023333333 | | 2.21 | | 6.565510138 | putative uncharacterized protein DDB G0282133 [Amyelois transitella] |
| bmsk0003853 | 0.006666667 | | 65.14 | | 13.25429051 | cuticular protein RR-2 motif 70 precursor |
| bmsk0012603 | 0.046666667 | | 6.956666667 | | 7.219859963 | cuticular protein RR-2 motif 105 precursor |
| bmsk0009932 | 6.96 | | 17.56 | | 1.335133634 | XP 012545428.1 globin 1 isoform X1 |
| bmsk0009503 | 33.21333333 | | 3.953333333 | | -3.070621012 | cell division cycle 2 |
| bmsk0012503 | 168.23 | | 357.06 | | 1.085731523 | ABF85696.1 transport protein Sec61 alpha subunit |
| bmsk0003987 | 0.693333333 | | 2.07 | | 1.57800974 | receptor type guanylyl cyclase |
| bmsk0010182 | 0.713333333 | | 4.38 | | 2.618282574 | cuticular protein RR-1 motif 7 precursor |
| bmsk0008990 | 10.81333333 | | 0.001 | | -13.4005237 | glypican-4 |
| bmsk0000346 | 0.001 | | 2.643333333 | | 11.36814265 | abnormal cell migration protein 10 isoform X2 |
| bmsk0001804 | 78.22666667 | | 165.08 | | 1.077432947 | prohibitin protein WPH isoform X1 |
| bmsk0002698 | 0.793333333 | | 1.633333333 | | 1.041820176 | potassium voltage-gated channel subfamily KQT member 1 isoform X1 |
| mstrg.16035 | 0.94 | | 4.38 | | 2.220198208 | PREDICTED: fatty acyl-CoA reductase 1-like [Bombyx mori] |
| mstrg.5283 | 5.28 | | 22.49 | | 2.090673826 | olfactory receptor [Bombyx mori] |
| bmsk0003627 | 0.513333333 | | 4.3 | | 3.06636881 | attacin-like |
| mstrg.10782 | 40.67 | | 4.62 | | -3.138000234 | PREDICTED: cyclin-dependent kinases regulatory subunit [Bombyx mori] |
| bmsk0007098 | 0.026666667 | | 13.58 | | 8.992230265 | cuticular protein glycine-rich 15 precursor |
| bmsk0012067 | 0.001 | | 20.72666667 | | 14.3392005 | cuticular protein RR-2 motif 77 precursor |
| bmsk0011681 | 29.18666667 | | 0.001 | | -14.83302183 | ovary C/EBPg transcription factor |
| bmsk0013199 | 2.143333333 | | 5.033333333 | | 1.231657907 | organic cation transporter [Danaus plexippus] |
| bmsk0009171 | 1.586666667 | | 3.253333333 | | 1.035919574 | uncharacterized protein LOC101743410 |
| bmsk0006873 | 0.583333333 | | 2.413333333 | | 2.048634775 | glutamate transporter isoform X1 |
| bmsk0013761 | 0.2 | | 0.846666667 | | 2.081794091 | - |
| bmsk0008350 | 2.996666667 | | 0.033333333 | | -6.490249211 | transcription factor E74, partial |
| bmsk0009033 | 19.35 | | 2.563333333 | | -2.916240564 | zinc finger protein NOS-O isoform X1 |
| mstrg.18592 | 43.77333333 | | 4.24 | | -3.367916079 | Zinc finger protein 41 [Pararge aegeria] |
| bmsk0014503 | 4.58 | | 9.256666667 | | 1.015145173 | - |
| bmsk0001301 | 0.7 | | 3.9 | | 2.478047297 | cGMP-dependent protein kinase, isozyme 2 forms cD4/T1/T3A/T3B [Papilio xuthus] |
| bmsk0012613 | 0.001 | | 12.08666667 | | 13.5611288 | FAA00598.1 TPA: putative cuticle protein |
| bmsk0002728 | 3.946666667 | | 11.54 | | 1.547936644 | uncharacterized protein LOC101746304 |
| mstrg.667 | 8.966666667 | | 30.20666667 | | 1.752223318 | olfactory receptor [Bombyx mori] |
| bmsk0012062 | 0.02 | | 21.18333333 | | 10.04871391 | cuticular protein RR-2 motif 75 precursor |
| bmsk0012179 | 8.26 | | 0.001 | | -13.01192607 | XP 004929658.1 G kinase-anchoring protein 1 |
| bmsk0010532 | 22.79666667 | | 4.073333333 | | -2.484541104 | uncharacterized protein LOC106719879 [Papilio machaon] |
| bmsk0012743 | 0.07 | | 14.14 | | 7.658211483 | FAA00533.1 TPA: putative cuticle protein, partial |
| bmsk0004519 | 0.001 | | 1.226666667 | | 10.26052755 | cytochrome P450, family 315, subfamily a, polypeptide 1 isoform X1 |
| bmsk0008344 | 2.416666667 | | 5.063333333 | | 1.06706897 | - |
| bmsk0006406 | 25.94333333 | | 3.09 | | -3.069685113 | CDT1 |
| bmsk0014461 | 5.976666667 | | 0.913333333 | | -2.71012769 | XP 004925106.1 UPF0669 protein C6orf120 homolog isoform X2 |
| bmsk0002267 | 0.023333333 | | 4.573333333 | | 7.614709844 | cuticular protein RR-2 motif 62 precursor |
| bmsk0000608 | 5.78 | | 13.83666667 | | 1.259355033 | basement membrane-specific heparan sulfate proteoglycan core protein-like isoform X3 |
| bmsk0013390 | 0.053333333 | | 10.11 | | 7.566529783 | odorant binding protein LOC100307012 precursor |
| bmsk0001980 | 0.001 | | 1.103333333 | | 10.107653 | collagen alpha-2(IV) chain |
| mstrg.441 | 32.94666667 | | 6.463333333 | | -2.349782207 | PREDICTED: putative serine protease K12H4.7 [Bombyx mori] |
| bmsk0012064 | 0.001 | | 11.02 | | 13.4278366 | cuticular protein RR-2 motif 138 precursor |
| mstrg.26220 | 10.31666667 | | 46.29 | | 2.165723654 | reverse transcriptase-like protein [Bombyx mori] |
| bmsk0012608 | 0.016666667 | | 11.76666667 | | 9.463524373 | putative cuticle protein |
| bmsk0015873 | 7.313333333 | | 0.86 | | -3.088120555 | - |
| bmsk0007267 | 4.146666667 | | 0.001 | | -12.01773636 | uncharacterized protein LOC101739362 |
| bmsk0002497 | 8.543333333 | | 62.35333333 | | 2.867595709 | ice nucleation protein-like [Amyelois transitella] |
| bmsk0002046 | 0.336666667 | | 1.413333333 | | 2.069708972 | chitin synthase A isoform X3 |
| bmsk0011745 | 9.436666667 | | 19.57666667 | | 1.052785889 | - |
| bmsk0012611 | 0.036666667 | | 8.266666667 | | 7.816692787 | cuticular protein RR-2 motif 97 precursor |
| bmsk0010872 | 0.006666667 | | 12.30333333 | | 10.84979602 | XP 004933976.1 pupal cuticle protein 27 |
| bmsk0004663 | 1.016666667 | | 2.17 | | 1.093848301 | mucin-5AC |
| bmsk0005390 | 50.98 | | 103.7966667 | | 1.025756835 | putative aldehyde dehydrogenase family 7 member A1 homolog [Amyelois transitella] |
| bmsk0002243 | 0.083333333 | | 0.966666667 | | 3.5360529 | glycerol-3-phosphate dehydrogenase |
| bmsk0006537 | 0.103333333 | | 0.62 | | 2.584962501 | cuticular protein hypothetical 12 precursor |
| bmsk0007470 | 24.81333333 | | 2.596666667 | | -3.256382822 | adenosine deaminase-like protein |
| bmsk0012609 | 0.001 | | 5.633333333 | | 12.45977313 | XP 004934088.1 extensin-1-like, partial |
| bmsk0000809 | 11.72333333 | | 1.65 | | -2.828844905 | Helicase conserved C-terminal domain |
| bmsk0004507 | 1.173333333 | | 4.513333333 | | 1.943580405 | septin-2 [Amyelois transitella] |
| bmsk0012066 | 0.001 | | 5.253333333 | | 12.35901741 | cuticular protein RR-2 motif 76 precursor |
| bmsk0009085 | 5.576666667 | | 12.92333333 | | 1.21250329 | pro-phenol oxidase [Bombyx mandarina] |
| bmsk0006315 | 0.84 | | 4.243333333 | | 2.33673678 | balbiani ring protein 3-like [Amyelois transitella] |
| bmsk0008201 | 0.41 | | 3.793333333 | | 3.209770337 | - |
| bmsk0010262 | 3.976666667 | | 0.001 | | -11.95734392 | ABD36119.1 G10 protein |
| bmsk0003698 | 0.37 | | 1.003333333 | | 1.43920381 | transient receptor potential channel pyrexia, partial |
| bmsk0015668 | 331.1733333 | | 787.1533333 | | 1.249058183 | heat shock protein 70 A1 |
| mstrg.23206 | 12.00666667 | | 2.336666667 | | -2.361311832 | PREDICTED: exportin-5-like [Plutella xylostella] |
| bmsk0005216 | 20.01333333 | | 3.02 | | -2.728341022 | protoporphyrinogen oxidase |
| bmsk0015116 | 12.49333333 | | 2.34 | | -2.416578017 | XP 004933026.1 zinc finger protein on ecdysone puffs isoform X1 |
| bmsk0012605 | 0.02 | | 2.296666667 | | 6.843397672 | putative cuticle protein |
| bmsk0011477 | 5.756666667 | | 14.60666667 | | 1.343321401 | tubulointerstitial nephritis antigen precursor |
| bmsk0013438 | 19.44 | | 2.543333333 | | -2.934235757 | XP 004930689.1 PWWP domain-containing protein 2A isoform X1 |
| bmsk0005846 | 7.826666667 | | 1.013333333 | | -2.94928918 | regulator of telomere elongation helicase 1 homolog isoform X2 |
| bmsk0007418 | 0.14 | | 1.416666667 | | 3.339001608 | elongation of very long chain fatty acids protein 4 |
| bmsk0004571 | 1109.69 | | 270.2333333 | | -2.037879158 | ABF51255.1 mobility group protein 1B |
| bmsk0008641 | 0.446666667 | | 1.56 | | 1.804275529 | matrix metalloproteinase 1 isoform X2 |
| bmsk0015807 | 3.003333333 | | 8.44 | | 1.490678394 | XP 004933542.1 la-related protein 6 |
| bmsk0004276 | 0.7 | | 2.7 | | 1.94753258 | 4-hydroxyphenylpyruvate dioxygenase |
| bmsk0005668 | 4.783333333 | | 0.001 | | -12.22380062 | - |
| bmsk0010871 | 0.02 | | 4.123333333 | | 7.687667284 | cuticular protein RR-1 motif 13 precursor |
| bmsk0011925 | 0.033333333 | | 0.496666667 | | 3.897240426 | uncharacterized protein LOC106102902 [Papilio polytes] |
| bmsk0007978 | 2.336666667 | | 5.03 | | 1.106106457 | tR =m(4)X modification enzyme TRM13 homolog isoform X2 |
| bmsk0006825 | 0.136666667 | | 4.09 | | 4.903367529 | cuticular protein hypothetical 28 precursor |
| bmsk0010063 | 0.776666667 | | 4.146666667 | | 2.416584625 | yellow-e precursor |
| bmsk0012063 | 0.001 | | 5.713333333 | | 12.48011699 | cuticular protein RR-2 motif 138 precursor |
| bmsk0005005 | 0.606666667 | | 1.983333333 | | 1.708951218 | uncharacterized protein LOC101743820 |
| bmsk0013321 | 0.72 | | 4.173333333 | | 2.535131345 | XP 004932669.1 juvenile hormone binding protein ce-0330 isoform X3 |
| bmsk0014415 | 0.001 | | 0.863333333 | | 9.753773882 | putative uncharacterized protein DDB G0282133 [Amyelois transitella] |
| bmsk0011680 | 2.14 | | 0.001 | | -11.06339508 | Centrosome-associated C terminus |
| bmsk0002004 | 0.876666667 | | 2.346666667 | | 1.420512629 | v-src sarcoma (Schmidt-Ruppin A-2) viral oncogene homolog isoform X2 |
| bmsk0005316 | 1.33 | | 3.456666667 | | 1.377955243 | - |
| bmsk0001519 | 4.07 | | 0.53 | | -2.94096453 | melanoma-associated antigen G1-like [Papilio machaon] |
| bmsk0014822 | 0.546666667 | | 2.003333333 | | 1.873669176 | XP 012545358.1 nephrin isoform X1 |
| bmsk0006908 | 25.69666667 | | 4.343333333 | | -2.564706644 | protein KBP homolog |
| bmsk0010789 | 3.36 | | 0.001 | | -11.71424552 | XP 004926830.1 RUN domain-containing protein 1 |
| bmsk0007164 | 15.19666667 | | 3.573333333 | | -2.088412503 | synaptojanin-1 |
| bmsk0011953 | 0.81 | | 2.853333333 | | 1.816654483 | XP 012545245.1 fibrillin-1, partial |
| bmsk0006023 | 0.001 | | 1.19 | | 10.21674586 | sugar transporter ERD6-like 6 |
| bmsk0015120 | 0.001 | | 2.163333333 | | 11.07904026 | facilitated trehalose transporter Tret1-like |
| bmsk0007708 | 0.48 | | 1.006666667 | | 1.068479738 | dynamin-1-like protein |
| bmsk0003114 | 0.31 | | 1.84 | | 2.569365646 | uncharacterized protein LOC101742785 |
| bmsk0001086 | 0.613333333 | | 1.653333333 | | 1.430634354 | Ig-like and fibronectin type-III domain-containing protein 1 isoform X1 |
| bmsk0010507 | 2.513333333 | | 6.936666667 | | 1.464640536 | helix-loop-helix protein delilah-like [Amyelois transitella] |
| bmsk0001235 | 0.56 | | 0.001 | | -9.129283017 | kinesin-like protein unc-104 isoform X4 |
| bmsk0007422 | 1.486666667 | | 4.07 | | 1.452947585 | proton-coupled amino acid transporter 4-like |
| bmsk0007077 | 0.393333333 | | 1.07 | | 1.443786438 | phospholipase A1 member A-like |
| bmsk0011490 | 4.063333333 | | 8.143333333 | | 1.002955738 | - |
| bmsk0015755 | 0.076666667 | | 0.84 | | 3.453717967 | glucose dehydrogenase [FAD, quinone]-like [Papilio polytes] |
| bmsk0010933 | 0.153333333 | | 0.696666667 | | 2.183797176 | - |
| bmsk0005260 | 1.87 | | 0.001 | | -10.86882255 | zinc finger protein 433-like |
| bmsk0014875 | 3.486666667 | | 0.001 | | -11.76763273 | synaptotagmin-16 |
| bmsk0010223 | 20.41666667 | | 3.986666667 | | -2.356492455 | uncharacterized protein LOC101735977 isoform X1 |
| bmsk0007542 | 3.15 | | 0.193333333 | | -4.026189524 | neurotactin isoform X1 |
| bmsk0001105 | 0.896666667 | | 2.07 | | 1.206987096 | uncharacterized protein LOC101746935 isoform X1 |
| bmsk0004351 | 22.75333333 | | 4.636666667 | | -2.294917995 | C-5 cytosine-specific D methylase |
| bmsk0006789 | 0.206666667 | | 1.003333333 | | 2.279423366 | transmembrane protease serine 2-like |
| bmsk0012374 | 0.001 | | 0.146666667 | | 7.196397213 | XP 004925070.1 leucine-rich repeat-containing protein AAC1 isoform X1 |
| bmsk0004968 | 0.87 | | 2.063333333 | | 1.245889603 | zinc finger and BTB domain-containing protein 17 isoform X3 |
| bmsk0002836 | 0.001 | | 0.72 | | 9.491853096 | hypothetical protein RR46 11993 [Papilio xuthus] |
| bmsk0005976 | 0.001 | | 3.733333333 | | 11.86624861 | gelsolin |
| mstrg.16320 | 0.346666667 | | 25.4 | | 6.195135564 | PREDICTED: ommochrome-binding protein-like [Papilio xuthus] |
| bmsk0014724 | 11.59666667 | | 2.14 | | -2.438027476 | XP 004924536.1 D -directed R polymerase III subunit RPC6 |
| bmsk0004455 | 42.47666667 | | 10.29666667 | | -2.044493186 | AP-2 complex subunit alpha |
| bmsk0015205 | 17.31333333 | | 4.083333333 | | -2.084064265 | Phosphoglucomutase/phosphomannomutase, C-terminal domain |
| bmsk0012523 | 0.001 | | 1.403333333 | | 10.45464202 | cuticular protein RR-1 motif 47 isoform X1 |
| bmsk0001813 | 17.32666667 | | 3.51 | | -2.303451197 | D primase large subunit |
| bmsk0005644 | 4.54 | | 0.84 | | -2.434231065 | D polymerase delta catalytic subunit |
| bmsk0005645 | 4.54 | | 0.84 | | -2.434231065 | - |
| mstrg.14416 | 0.19 | | 2.183333333 | | 3.522461082 | PREDICTED: cuticular protein RR-1 motif 33 isoform X1 [Bombyx mori] |
| bmsk0007714 | 0.18 | | 0.593333333 | | 1.720845929 | cAMP-dependent protein kinase catalytic subunit-like |
| bmsk0009420 | 2.156666667 | | 0.21 | | -3.360341978 | optomotor-blind protein isoform X4 |
| bmsk0012465 | 5.243333333 | | 0.43 | | -3.6080757 | nucleoporin NUP53 [Amyelois transitella] |
| bmsk0011158 | 0.323333333 | | 0.92 | | 1.508611615 | chemosensory protein 10 isoform X1 |
| bmsk0005258 | 0.05 | | 0.47 | | 3.232660757 | ankyrin repeat domain-containing protein 29 isoform X3 |
| bmsk0007989 | 0.001 | | 0.796666667 | | 9.637832402 | TRAF3 |
| bmsk0000298 | 1.993333333 | | 0.001 | | -10.96096727 | RUS1 family protein C16orf58 homolog isoform X1 |
| bmsk0012709 | 0.113333333 | | 0.6 | | 2.404390255 | XP 004931901.1 uncharacterized protein LOC101746542 |
| bmsk0007522 | 6.17 | | 1.02 | | -2.596701337 | Adenylate and Guanylate cyclase catalytic domain |
| bmsk0006458 | 0.001 | | 21.06333333 | | 14.36244614 | phosphoglycolate phosphatase-like [Plutella xylostella] |
| bmsk0010930 | 0.153333333 | | 0.84 | | 2.453717967 | uncharacterized protein LOC101737484 |
| bmsk0013372 | 0.506666667 | | 1.326666667 | | 1.388697107 | hypothetical protein RR48 13994 [Papilio machaon] |
| bmsk0008110 | 0.223333333 | | 0.51 | | 1.191298652 | transient receptor potential protein isoform X1 |
| mstrg.10043 | 0.036666667 | | 1.72 | | 5.551795637 | - |
| bmsk0005217 | 20.12 | | 5.15 | | -1.965985968 | dynactin subunit 4 [Papilio machaon] |
| bmsk0002675 | 1.906666667 | | 3.816666667 | | 1.001260546 | phospholipid scramblase 2 isoform X1 [Papilio xuthus] |
| bmsk0009481 | 1.453333333 | | 0.001 | | -10.50514992 | CDP-diacylglycerol--inositol 3-phosphatidyltransferase |
| bmsk0014673 | 0.32 | | 1.156666667 | | 1.853829352 | XP 012544466.1 uncharacterized protein LOC105841398 isoform X2 |
| bmsk0004019 | 0.006666667 | | 2.68 | | 8.651051691 | hornerin-like |
| bmsk0010184 | 0.026666667 | | 0.656666667 | | 4.622051819 | cuticular protein RR-1 motif 9 precursor |
| bmsk0010660 | 0.026666667 | | 1.916666667 | | 6.167418146 | XP 004928296.1 brachyurin |
| bmsk0012785 | 0.23 | | 0.606666667 | | 1.399270183 | hypothetical protein KGM 01235 [Danaus plexippus] |
| bmsk0002756 | 0.17 | | 0.423333333 | | 1.316259345 | cilia- and flagella-associated protein 57 isoform X2 [Amyelois transitella] |
| mstrg.23050 | 0.576666667 | | 1.326666667 | | 1.201996393 | PREDICTED: protein rolling stone-like [Bombyx mori] |
| bmsk0009297 | 0.276666667 | | 0.683333333 | | 1.304440668 | vanin-like protein 2 isoform X1 |
| mstrg.1677 | 25.64666667 | | 5.176666667 | | -2.308676 | PREDICTED: DNA primase small subunit [Bombyx mori] |
| bmsk0002474 | 32.88 | | 8.39 | | -1.970467583 | solute carrier organic anion transporter family member 2A1-like isoform X1 |
| mstrg.12765 | 3.423333333 | | 0.116666667 | | -4.874937449 | PREDICTED: uncharacterized protein LOC101745265 [Bombyx mori] |
| bmsk0010406 | 0.001 | | 3.653333333 | | 11.83499768 | cuticular protein glycine-rich 27 precursor |
| bmsk0013647 | 0.001 | | 1.15 | | 10.16741815 | nitrilase homolog 1 isoform X2 |
| bmsk0004184 | 0.016666667 | | 0.74 | | 5.472487771 | cationic amino acid transporter 2 [Papilio machaon] |
| bmsk0004341 | 17.88666667 | | 3.516666667 | | -2.346604056 | transmembrane protein 19 isoform X2 |
| bmsk0003933 | 0.001 | | 1.596666667 | | 10.64084744 | - |
| bmsk0005091 | 0.263333333 | | 0.79 | | 1.584962501 | proton-associated sugar transporter A-like [Papilio polytes] |
| bmsk0010928 | 0.166666667 | | 0.633333333 | | 1.925999419 | nose resistant to fluoxetine protein 6-like [Amyelois transitella] |
| bmsk0012757 | 14.80666667 | | 3.166666667 | | -2.225209974 | probable tR N6-adenosine threonylcarbamoyltransferase, mitochondrial, partial [Papilio machaon] |
| bmsk0001712 | 3.053333333 | | 0.563333333 | | -2.438324352 | heparin sulfate O-sulfotransferase [Amyelois transitella] |
| bmsk0012623 | 0.001 | | 0.563333333 | | 9.13784503 | cuticular protein RR-2 motif 86 precursor |
| bmsk0010529 | 0.173333333 | | 1.246666667 | | 2.846454742 | UDP-glycosyltransferase UGT50A1 precursor |
| bmsk0000075 | 14.67666667 | | 1.816666667 | | -3.014158709 | Bmptp-Z and Bmap-A fusion protein gamma |
| bmsk0006342 | 6.35 | | 0.186666667 | | -5.08822036 | supervillin isoform X3 |
| bmsk0001472 | 4.06 | | 0.6 | | -2.758445322 | BLOC-1-related complex subunit 6 isoform X1 |
| bmsk0015683 | 0.053333333 | | 0.2 | | 1.906890596 | facilitated trehalose transporter Tret1-like |
| bmsk0012131 | 7.96 | | 1.416666667 | | -2.49026809 | G patch domain and KOW motifs-containing protein [Plutella xylostella] |
| bmsk0007900 | 1.143333333 | | 3.41 | | 1.576525664 | MOB kinase activator-like 2 isoform X3 [Amyelois transitella] |
| bmsk0010980 | 16.93666667 | | 4.753333333 | | -1.833138482 | peptide-N(4)-(N-acetyl-beta-glucosaminyl)asparagine amidase |
| bmsk0004945 | 8.74 | | 1.886666667 | | -2.211793727 | protein timeless homolog isoform X1 |
| bmsk0008279 | 0.001 | | 0.456666667 | | 8.834997677 | uncharacterized protein LOC101738072 isoform X3 |
| bmsk0012615 | 0.001 | | 0.42 | | 8.714245518 | cuticular protein RR-2 motif 92 precursor |
| bmsk0013466 | 21.2 | | 4.106666667 | | -2.368024509 | XP 004930707.1 THAP domain-containing protein 1 |
| bmsk0010420 | 12.18 | | 2.05 | | -2.570818318 | gastrula zinc finger protein XlCGF57.1-like [Amyelois transitella] |
| bmsk0007109 | 0.001 | | 2.05 | | 11.00140819 | putative eggshell protein [Papilio polytes] |
| bmsk0010250 | 0.001 | | 1.443333333 | | 10.49518881 | - |
| bmsk0008612 | 4.76 | | 1.036666667 | | -2.199009494 | E3 ubiquitin-protein ligase LRSAM1 |
| bmsk0014124 | 10.89 | | 1.723333333 | | -2.659730269 | XP 021203775.1 uncharacterized protein LOC101736046 isoform X1 |
| bmsk0007643 | 28.67666667 | | 4.846666667 | | -2.564812569 | anaphase promoting complex subunit 10 |
| bmsk0008759 | 3.97 | | 0.63 | | -2.655715274 | Wd-repeat protein [Operophtera brumata] |
| bmsk0013101 | 0.001 | | 0.513333333 | | 9.003752135 | aminoacylase |
| bmsk0013662 | 2.23 | | 0.016666667 | | -7.063934306 | uncharacterized protein LOC101743235 isoform X3 |
| bmsk0010857 | 0.37 | | 0.896666667 | | 1.277046496 | - |
| bmsk0003122 | 52.76 | | 15.28333333 | | -1.787485331 | programmed cell death 6-interacting protein |
| bmsk0000510 | 0.073333333 | | 0.273333333 | | 1.898120386 | autophagy-related protein 9A |
| bmsk0012631 | 0.193333333 | | 0.796666667 | | 2.042885813 | cuticular protein RR-2 motif 79 precursor |
| bmsk0004410 | 10.58666667 | | 2.28 | | -2.215142682 | replication protein A1 |
| bmsk0010352 | 9.653333333 | | 1.766666667 | | -2.449997338 | uncharacterized protein LOC101745082 isoform X2 |
| bmsk0009257 | 3.186666667 | | 6.443333333 | | 1.015759114 | fructose-bisphosphate aldolase isoform X1 |
| bmsk0006352 | 0.793333333 | | 0.001 | | -9.631783357 | C-type lectin domain family 4 member F |
| bmsk0006030 | 1.186666667 | | 0.001 | | -10.21269903 | gastrula zinc finger protein xFG20-1-like |
| bmsk0005172 | 0.06 | | 2.386666667 | | 5.313890776 | uncharacterized protein LOC101736774 isoform X2 |
| bmsk0002541 | 1.98 | | 0.036666667 | | -5.754887502 | PAS domain-containing serine/threonine-protein kinase [Amyelois transitella] |
| bmsk0010659 | 0.763333333 | | 0.001 | | -9.576169382 | Semaphorin-2A [Papilio xuthus] |
| bmsk0001367 | 1.34 | | 6.326666667 | | 2.239212586 | protein split ends |
| bmsk0001505 | 0.001 | | 0.476666667 | | 8.896836931 | G patch domain and ankyrin repeat-containing protein 1 homolog [Papilio machaon] |
| bmsk0010548 | 0.696666667 | | 3.76 | | 2.43219222 | Uncharacterized protein OBRU01 02397 [Operophtera brumata] |
| mstrg.25461 | 51.77666667 | | 11.62 | | -2.15569202 | geminin [Bombyx mori] |
| bmsk0011936 | 0.886666667 | | 1.81 | | 1.029525952 | XP 004927327.1 DH dehydrogenase [ubiquinone] 1 beta subcomplex subunit 3 |
| bmsk0013823 | 2.273333333 | | 4.573333333 | | 1.008436837 | - |
| bmsk0001383 | 56.82 | | 9.613333333 | | -2.563290168 | transcriptional adaptor 3 |
| bmsk0004245 | 11.1 | | 2.723333333 | | -2.027114194 | calpain-7 isoform X1 |
| bmsk0001859 | 32.84666667 | | 7.986666667 | | -2.040081564 | cyclin-Y-like protein 1 [Amyelois transitella] |
| bmsk0008644 | 4.643333333 | | 0.683333333 | | -2.764499443 | retinal homeobox protein Rx3 |
| bmsk0004493 | 3.77 | | 0.566666667 | | -2.733992278 | condensin complex subunit 1 |
| mstrg.1644 | 21.51 | | 5.05 | | -2.090652232 | PREDICTED: methylosome protein 50-like [Bombyx mori] |
| bmsk0008754 | 3.2 | | 0.24 | | -3.736965594 | uncharacterized protein LOC101746139 |
| bmsk0007442 | 5.38 | | 1.04 | | -2.371022644 | GDP-fucose transporter 1 |
| bmsk0004465 | 12.3 | | 1.126666667 | | -3.448525665 | protein suppressor of sable isoform X2 |
| bmsk0008094 | 0.073333333 | | 0.63 | | 3.102810806 | glucose dehydrogenase [FAD, quinone]-like [Papilio xuthus] |
| bmsk0010841 | 0.306666667 | | 0.001 | | -8.26052755 | XP 004927006.1 muskelin isoform X1 |
| bmsk0000650 | 0.503333333 | | 1.386666667 | | 1.462034979 | Domain of unknown function (DUF4780) |
| bmsk0004183 | 0.136666667 | | 0.3 | | 1.134301092 | cationic amino acid transporter 3 |
| bmsk0006647 | 40.57 | | 11.49333333 | | -1.819616029 | mediator of R polymerase II transcription subunit 10 [Papilio polytes] |
| bmsk0004020 | 0.006666667 | | 0.88 | | 7.044394119 | hornerin-like |
| bmsk0011756 | 1.523333333 | | 0.001 | | -10.57301595 | hypothetical protein ALC57 15604 [Trachymyrmex cornetzi] |
| bmsk0008719 | 0.001 | | 0.113333333 | | 6.824428435 | probable multidrug resistance-associated protein lethal(2)03659 |
| mstrg.12764 | 3.33 | | 0.156666667 | | -4.409752016 | PREDICTED: uncharacterized protein LOC101736344 [Bombyx mori] |
| bmsk0007094 | 12.26333333 | | 2.16 | | -2.505247958 | coiled-coil domain-containing protein 97 [Amyelois transitella] |
| bmsk0000367 | 0.001 | | 1.423333333 | | 10.47505785 | uncharacterized protein LOC105842168 |
| bmsk0010613 | 0.35 | | 0.001 | | -8.451211112 | XP 004928406.1 inositol-tetrakisphosphate 1-kinase |
| bmsk0004701 | 7.613333333 | | 1.57 | | -2.277763686 | uncharacterized protein LOC101739530 isoform X1 |
| bmsk0006037 | 19.47333333 | | 4.613333333 | | -2.077618415 | uncharacterized protein LOC106134389 [Amyelois transitella] |
| bmsk0007950 | 1.316666667 | | 0.013333333 | | -6.625708843 | uncharacterized protein LOC106719359 [Papilio machaon] |
| bmsk0004873 | 0.326666667 | | 0.866666667 | | 1.407657969 | - |
| bmsk0013319 | 20.06333333 | | 4.486666667 | | -2.160845407 | Replication factor C 38kD subunit [Operophtera brumata] |
| bmsk0004885 | 0.14 | | 0.92 | | 2.716207034 | uncharacterized protein LOC106143247 [Amyelois transitella] |
| bmsk0008073 | 0.326666667 | | 1.8 | | 2.462105753 | stromal interaction molecule 1 precursor |
| bmsk0011903 | 8.083333333 | | 1.436666667 | | -2.492224973 | ubiquitin-conjugating enzyme E2 T-like [Papilio polytes] |
| mstrg.24646 | 22.39666667 | | 5.106666667 | | -2.132830233 | PREDICTED: F-box/WD repeat-containing protein 4-like isoform X1 [Bombyx mori] |
| bmsk0011276 | 0.043333333 | | 0.363333333 | | 3.067744607 | XP 021206667.1 ninjurin-2 isoform X3 |
| bmsk0011958 | 3.24 | | 6.946666667 | | 1.100327059 | XP 004925448.1 uncharacterized protein LOC101736566 |
| bmsk0009369 | 0.946666667 | | 0.076666667 | | -3.626185163 | vacuolar protein sorting 37B |
| bmsk0010222 | 23.74 | | 5.683333333 | | -2.062510696 | non-histone protein 10 [Papilio polytes] |
| bmsk0012637 | 22.48333333 | | 5.373333333 | | -2.064966699 | sulfatase-modifying factor 1 |
| bmsk0006069 | 2.82 | | 7.4 | | 1.391830108 | heat shock protein 68-like |
| bmsk0007855 | 15.13 | | 2.726666667 | | -2.47220174 | nitric oxide synthase-interacting protein homolog |
| bmsk0000897 | 11.86 | | 2.776666667 | | -2.09467811 | - |
| bmsk0006987 | 15.91 | | 3.346666667 | | -2.249137067 | transmembrane protein 62-like |
| bmsk0013154 | 28.6 | | 7.28 | | -1.974004791 | XP 004921720.1 THO complex subunit 7 homolog isoform X1 |
| bmsk0008260 | 1.176666667 | | 0.076666667 | | -3.939962417 | speckle-type POZ protein-like [Plutella xylostella] |
| bmsk0013617 | 0.056666667 | | 0.643333333 | | 3.504994196 | uncharacterized protein LOC101741500 |
| bmsk0012664 | 0.173333333 | | 0.48 | | 1.469485283 | - |
| bmsk0012591 | 0.001 | | 1.42 | | 10.47167521 | cuticular protein RR-2 motif 114 precursor |
| bmsk0008457 | 8.963333333 | | 2.27 | | -1.981343052 | histone-lysine N-methyltransferase ASHR1 |
| bmsk0008808 | 0.436666667 | | 0.963333333 | | 1.141502681 | oxygen resistance gene 1 isoform X6 |
| bmsk0006617 | 33.95333333 | | 5.873333333 | | -2.531301793 | uncharacterized protein LOC100286771 isoform X1 |
| bmsk0006639 | 1.283333333 | | 0.001 | | -10.32568023 | trimethylguanosine synthase |
| bmsk0010252 | 6.113333333 | | 12.75333333 | | 1.060843235 | Aconitase family (aconitate hydratase) |
| bmsk0000936 | 0.001 | | 0.23 | | 7.845490051 | ced-6 protein isoform X1 |
| bmsk0006650 | 4.48 | | 0.68 | | -2.719892081 | mitochondrial thiamine pyrophosphate carrier |
| bmsk0012149 | 0.376666667 | | 1.036666667 | | 1.460591808 | hypothetical protein KGM 11843 [Danaus plexippus] |
| mstrg.15041 | 12.96666667 | | 3.056666667 | | -2.084776516 | PREDICTED: membrane-bound transcription factor site-2 protease [Bombyx mori] |
| bmsk0004922 | 27.60666667 | | 5.65 | | -2.288693929 | dnaJ (Hsp40) homolog 10 isoform X1 |
| bmsk0000505 | 13.16 | | 2.98 | | -2.142775253 | ubiquitin carboxyl-terminal hydrolase isozyme L5 isoform X2 |
| mstrg.25264 | 4.336666667 | | 0.85 | | -2.35105181 | reverse transcriptase [Lasius niger] |
| mstrg.25220 | 18.89666667 | | 3.563333333 | | -2.406832416 | PREDICTED: phosphatidylinositol-glycan biosynthesis class X protein isoform X1 [Bombyx mori] |
| bmsk0010697 | 5.356666667 | | 0.62 | | -3.110995403 | dopamine N-acetyltransferase-like |
| bmsk0003424 | 8.023333333 | | 1.406666667 | | -2.511921238 | non-structural maintenance of chromosomes element 1 homolog [Amyelois transitella] |
| bmsk0012930 | 5.83 | | 0.59 | | -3.304709024 | XP 012544618.1 uncharacterized protein LOC101737053 |
| bmsk0001735 | 1.013333333 | | 0.046666667 | | -4.440572591 | protein asteroid |
| bmsk0000271 | 0.001 | | 0.123333333 | | 6.94641896 | serine/threonine-protein kinase OSR1-like [Amyelois transitella] |
| bmsk0013485 | 0.24 | | 0.001 | | -7.906890596 | XP 021206611.1 ovarian tumor protein isoform X1 |
| bmsk0012596 | 0.001 | | 0.716666667 | | 9.485158444 | cuticular protein RR-2 motif 109 precursor |
| bmsk0001384 | 2.36 | | 0.2 | | -3.560714954 | zinc finger protein 426-like isoform X2 |
| bmsk0016013 | 0.123333333 | | 0.616666667 | | 2.321928095 | UDP-glucoronosyl and UDP-glucosyl transferase |
| bmsk0010509 | 18.06 | | 3.42 | | -2.400729663 | XP 004928169.1 inhibitor of nuclear factor kappa-B kinase subunit beta |
| bmsk0014256 | 3.666666667 | | 0.026666667 | | -7.103287808 | XP 021209087.1 uncharacterized protein LOC101744973 isoform X2 |
| bmsk0003353 | 0.23 | | 0.546666667 | | 1.249027548 | uncharacterized protein LOC101741926 |
| bmsk0004187 | 0.063333333 | | 0.306666667 | | 2.275634443 | Low affinity cationic amino acid transporter 2 [Papilio xuthus] |
| bmsk0008711 | 8.43 | | 1.566666667 | | -2.427834375 | decapping nuclease DXO homolog isoform X1 |
| bmsk0012625 | 0.053333333 | | 6.83 | | 7.000704269 | cuticular protein RR-2 motif 84 precursor |
| bmsk0000879 | 0.033333333 | | 0.6 | | 4.169925001 | - |
| bmsk0015040 | 0.04 | | 0.836666667 | | 4.386581053 | lipase member H-B-like isoform X2 |
| bmsk0013518 | 13.10333333 | | 2.44 | | -2.42498081 | importin subunit alpha-5-like isoform X2 |
| bmsk0004164 | 1.08 | | 2.643333333 | | 1.291327053 | putative inorganic phosphate cotransporter |
| bmsk0006735 | 31.02333333 | | 8.526666667 | | -1.863299943 | serine/arginine repetitive matrix protein 5 isoform X1 |
| bmsk0007531 | 18.42666667 | | 3.373333333 | | -2.449548326 | coiled-coil domain-containing protein 94 [Amyelois transitella] |
| bmsk0008511 | 2.01 | | 0.11 | | -4.191620073 | GTPase-activating Rap/Ran-GAP domain-like protein 3 isoform X3 |
| bmsk0003195 | 0.13 | | 0.63 | | 2.276840205 | - |
| bmsk0012517 | 15.39 | | 2.963333333 | | -2.376700408 | (6-4) photolyase [Operophtera brumata] |
| bmsk0011937 | 26.19666667 | | 6.63 | | -1.982302476 | XP 004927331.1 SOSS complex subunit B homolog |
| bmsk0012329 | 1.886666667 | | 0.093333333 | | -4.337303321 | XP 021202917.1 myoneurin isoform X2 |
| bmsk0005056 | 7.303333333 | | 1.256666667 | | -2.538953056 | protein PET100 homolog, mitochondrial |
| bmsk0000849 | 2.836666667 | | 0.663333333 | | -2.096390701 | uncharacterized protein LOC101742758 isoform X1 |
| mstrg.19412 | 0.053333333 | | 0.33 | | 2.62935662 | olfactory receptor [Bombyx mori] |
| bmsk0003026 | 13.8 | | 3.126666667 | | -2.14197094 | chromatin accessibility complex protein 1 |
| bmsk0015988 | 25.72333333 | | 5.636666667 | | -2.190163446 | XP 012544259.1 MIP18 family protein CG30152 |
| bmsk0008035 | 0.193333333 | | 0.436666667 | | 1.175442006 | roundabout homolog 3 |
| bmsk0006528 | 0.001 | | 0.12 | | 6.906890596 | cuticular protein hypothetical 22 precursor |
| bmsk0010842 | 0.213333333 | | 0.69 | | 1.693486957 | myosin-7B isoform X2 [Papilio xuthus] |
| mstrg.14474 | 23.63 | | 4.143333333 | | -2.511755834 | PREDICTED: zinc finger protein 729-like isoform X2 [Bombyx mori] |
| mstrg.6851 | 14.63 | | 3.013333333 | | -2.279497592 | PREDICTED: NADPH:adrenodoxin oxidoreductase, mitochondrial [Bombyx mori] |
| bmsk0001733 | 7.506666667 | | 1.133333333 | | -2.727600176 | uncharacterized protein LOC106143196 [Amyelois transitella] |
| bmsk0007170 | 2.12 | | 0.023333333 | | -6.505528033 | D repair endonuclease XPF [Amyelois transitella] |
| bmsk0005302 | 0.03 | | 0.266666667 | | 3.152003093 | cytochrome P450 6B2-like |
| bmsk0005383 | 3.336666667 | | 0.07 | | -5.574908836 | Transposable element P transposase [Papilio machaon] |
| bmsk0008070 | 20.20333333 | | 2.806666667 | | -2.847663704 | cytoskeleton-associated protein 5 |
| bmsk0008783 | 29.57333333 | | 7.986666667 | | -1.888631457 | transcription factor-like protein |
| bmsk0008512 | 28.32 | | 6.846666667 | | -2.048347584 | ZZ-type zinc finger-containing protein 3 |
| bmsk0000131 | 0.116666667 | | 0.293333333 | | 1.330148602 | solute carrier family 22 member 13-like [Papilio xuthus] |
| bmsk0009618 | 1.456666667 | | 0.01 | | -7.186526969 | protein lethal(2)k10201-like [Plutella xylostella] |
| bmsk0008872 | 11.57 | | 2.716666667 | | -2.090479401 | uncharacterized protein LOC101740766 |
| bmsk0003047 | 15.03 | | 3.746666667 | | -2.004165474 | mediator of R polymerase II transcription subunit 27 |
| bmsk0005338 | 22.89666667 | | 4.43 | | -2.36975898 | uncharacterized protein LOC101743276 |
| bmsk0005514 | 28.47 | | 4.17 | | -2.771323204 | Vacuolar protein-sorting protein BRO1 [Operophtera brumata] |
| bmsk0012650 | 0.136666667 | | 0.323333333 | | 1.242360838 | XP 012550801.1 uncharacterized protein LOC101742417 |
| bmsk0004569 | 7.276666667 | | 0.873333333 | | -3.058673413 | uncharacterized protein LOC101738502 |
| bmsk0008398 | 0.266666667 | | 0.943333333 | | 1.822730148 | protein vav isoform X1 |
| bmsk0001298 | 0.603333333 | | 0.001 | | -9.236811481 | THAP domain |
| mstrg.23780 | 15.44666667 | | 3.45 | | -2.162627276 | PREDICTED: TAF5-like RNA polymerase II p300/CBP-associated factor-associated factor 65 kDa subunit 5L [Amyelois transitella] |
| bmsk0011878 | 6.683333333 | | 1.293333333 | | -2.369473679 | ADD10141.1 origin recognition complex subunit 5 |
| bmsk0009343 | 0.133333333 | | 1.926666667 | | 3.852997588 | - |
| bmsk0013329 | 0.053333333 | | 0.366666667 | | 2.781359714 | XP 004932671.1 protein takeout |
| bmsk0006525 | 0.02 | | 1.346666667 | | 6.073248982 | pupal cuticle protein C1B |
| bmsk0012998 | 2.903333333 | | 0.001 | | -11.5034945 | histone H2AX-like protein |
| bmsk0009286 | 0.13 | | 0.356666667 | | 1.456064768 | glucose dehydrogenase [FAD, quinone] |
| bmsk0008397 | 3.176666667 | | 0.546666667 | | -2.538780399 | KWMTBOMO08896 |
| bmsk0003616 | 0.29 | | 2.96 | | 3.351472371 | uncharacterized protein LOC105389986 [Plutella xylostella] |
| bmsk0007769 | 1.506666667 | | 0.203333333 | | -2.889441625 | transmembrane protein 184C [Papilio xuthus] |
| bmsk0004532 | 0.001 | | 0.596666667 | | 9.220781371 | R recognition motif. (a.k.a. RRM, RBD, or RNP domain) |
| bmsk0012592 | 0.001 | | 0.903333333 | | 9.819114636 | cuticular protein RR-2 motif 113 precursor |
| bmsk0008722 | 7.736666667 | | 2.006666667 | | -1.946911131 | UDP-glucuronic acid decarboxylase 1 |
| bmsk0015085 | 9.98 | | 2.17 | | -2.201344773 | XP 012551699.1 biorientation of chromosomes in cell division protein 1-like 1 isoform X1 |
| bmsk0011349 | 1.09 | | 0.17 | | -2.680721484 | - |
| bmsk0012893 | 3.403333333 | | 0.423333333 | | -3.007082464 | exocyst complex component 4 |
| bmsk0006838 | 7.596666667 | | 0.903333333 | | -3.072036168 | sprT-like domain-containing protein Spartan [Papilio machaon] |
| bmsk0011561 | 2.843333333 | | 0.5 | | -2.507583241 | gastrula zinc finger protein XlCGF26.1-like isoform X2 [Amyelois transitella] |
| bmsk0002729 | 15.14 | | 2.826666667 | | -2.421191536 | uncharacterized protein LOC101746063 isoform X3 |
| bmsk0015109 | 7.026666667 | | 1.276666667 | | -2.46045857 | Zinc-finger of R -polymerase I-specific TFIIB, Rrn7 |
| bmsk0007661 | 33.09 | | 7.293333333 | | -2.181745054 | zinc transporter 9 [Amyelois transitella] |
| bmsk0001508 | 0.016666667 | | 0.146666667 | | 3.137503524 | sarcoplasmic calcium-binding protein 1 |
| bmsk0012201 | 25.28 | | 5.203333333 | | -2.280488427 | cyclin dependent kinase 7 |
| bmsk0002113 | 1.703333333 | | 0.001 | | -10.73414508 | T-box transcription factor TBX20 isoform X2 |
| bmsk0014255 | 21.82333333 | | 5.416666667 | | -2.010394261 | XP 004923124.1 uncharacterized protein LOC101737900 |
| bmsk0012651 | 1.213333333 | | 0.13 | | -3.222392421 | uncharacterized protein LOC106132394 isoform X2 [Amyelois transitella] |
| bmsk0013141 | 12.97333333 | | 2.913333333 | | -2.154806525 | delta-1-pyrroline-5-carboxylate synthase isoform X1 [Papilio polytes] |
| bmsk0010152 | 0.6 | | 0.001 | | -9.22881869 | spermatogenesis-associated protein 20-like isoform X2 |
| bmsk0010849 | 0.033333333 | | 0.403333333 | | 3.596935142 | XP 012546508.1 nose resistant to fluoxetine protein 6 |
| bmsk0003494 | 11.26666667 | | 2.64 | | -2.093450911 | vacuolar protein sorting-associated protein 72 homolog [Amyelois transitella] |
| bmsk0004702 | 9.74 | | 2.393333333 | | -2.024900429 | protein-S-isoprenylcysteine O-methyltransferase |
| bmsk0008388 | 18.02666667 | | 4.963333333 | | -1.860751398 | nucleolar complex protein 4 homolog B isoform X2 |
| bmsk0005684 | 14.70333333 | | 3.033333333 | | -2.27716731 | ABD36106.1 E2F transcription factor 4-like protein |
| bmsk0010272 | 3.523333333 | | 0.456666667 | | -2.947727578 | endonuclease III-like protein 1 isoform X3 |
| bmsk0015083 | 5.98 | | 1.1 | | -2.442641961 | myb-like protein X isoform X2 |
| bmsk0003435 | 17.81666667 | | 4.08 | | -2.12658639 | pre-mR -splicing factor 18 isoform X1 |
| bmsk0007334 | 7.67 | | 0.8 | | -3.261154673 | trafficking protein particle complex subunit 4 [Plutella xylostella] |
| bmsk0014925 | 1.46 | | 0.001 | | -10.51175265 | potassium channel subfamily K member 9 [Amyelois transitella] |
| bmsk0003147 | 0.42 | | 0.001 | | -8.714245518 | organic cation/carnitine transporter 7-like |
| bmsk0002595 | 0.053333333 | | 0.31 | | 2.539158811 | sodium-coupled monocarboxylate transporter 1-like [Amyelois transitella] |
| bmsk0010646 | 4.49 | | 0.903333333 | | -2.313385094 | AFC01229.1 DnaJ-15 |
| bmsk0006917 | 0.786666667 | | 0.001 | | -9.619608644 | sodium-dependent nutrient amino acid transporter 1-like isoform X1 [Amyelois transitella] |
| bmsk0009564 | 0.786666667 | | 1.943333333 | | 1.304709024 | ribosomal protein P2 |
| bmsk0007691 | 0.256666667 | | 0.001 | | -8.003752135 | uncharacterized protein LOC101739818 isoform X1 |
| mstrg.16224 | 2.176666667 | | 0.283333333 | | -2.941548245 | zinc finger protein NANOS-P [Bombyx mori] |
| bmsk0013533 | 68.31 | | 19.36666667 | | -1.818521129 | enhancer of rudimentary homolog [Plutella xylostella] |
| bmsk0000079 | 0.853333333 | | 0.116666667 | | -2.870716983 | protein Jumonji isoform X1 |
| bmsk0005031 | 0.33 | | 0.001 | | -8.366322214 | zinc finger MIZ domain-containing protein 1 |
| bmsk0009942 | 9.483333333 | | 1.74 | | -2.446306941 | uncharacterized protein LOC101740899 |
| bmsk0008849 | 13.79333333 | | 3.036666667 | | -2.183410686 | ribonuclease H1 |
| bmsk0004518 | 11.79 | | 2.62 | | -2.169925001 | PRKR-interacting protein 1 homolog |
| bmsk0002762 | 6.706666667 | | 1.24 | | -2.435255779 | CD2 antigen cytoplasmic tail-binding protein 2 homolog |
| mstrg.10138 | 3.946666667 | | 1.07 | | -1.883023879 | PREDICTED: alkylated DNA repair protein alkB homolog 1 isoform X1 [Bombyx mori] |
| bmsk0008463 | 0.001 | | 0.613333333 | | 9.26052755 | phosrestin-2 |
| bmsk0003568 | 0.236666667 | | 1.076666667 | | 2.185643235 | - |
| bmsk0014422 | 9.283333333 | | 1.576666667 | | -2.557765239 | protein O-fucosyltransferase 2 precursor |
| bmsk0007789 | 0.45 | | 0.013333333 | | -5.076815597 | histone deacetylase complex subunit SAP30 homolog |
| bmsk0005382 | 19.80333333 | | 3.256666667 | | -2.604275321 | peroxisomal targeting signal 1 receptor [Amyelois transitella] |
| bmsk0003628 | 3.22 | | 0.001 | | -11.65284497 | collagen alpha chain CG42342 |
| bmsk0007126 | 23.87666667 | | 6.296666667 | | -1.92294124 | ribonucleoside-diphosphate reductase large subunit isoform X1 [Amyelois transitella] |
| bmsk0008422 | 0.001 | | 0.046666667 | | 5.544320516 | amidophosphoribosyltransferase-like isoform X1 |
| bmsk0014400 | 17.00333333 | | 4.75 | | -1.839818181 | RSM22-like protein, mitochondrial [Operophtera brumata] |
| bmsk0011195 | 0.07 | | 1.173333333 | | 4.067114196 | XP 004922398.2 uncharacterized protein LOC101735305 |
| bmsk0015117 | 0.283333333 | | 0.001 | | -8.14635653 | erythroid differentiation-related factor 1 |
| bmsk0000541 | 0.33 | | 0.001 | | -8.366322214 | ubiquitin-conjugating enzyme E2 G2 |
| bmsk0007811 | 9.603333333 | | 1.873333333 | | -2.357927625 | tudor domain-containing protein 3-like |
| bmsk0000801 | 0.03 | | 1.17 | | 5.285402219 | chorion class CA protein ERA.2-like isoform X1 |
| bmsk0014449 | 0.996666667 | | 0.016666667 | | -5.902073579 | uncharacterized protein LOC105841476 |
| bmsk0005226 | 0.093333333 | | 0.333333333 | | 1.836501268 | cytoplasmic dynein 1 intermediate chain isoform X9 |
| bmsk0003843 | 2.41 | | 0.256666667 | | -3.231065296 | uncharacterized protein LOC101742851 isoform X3 |
| bmsk0014450 | 0.09 | | 0.866666667 | | 3.267480311 | eIF2B-beta protein |
| bmsk0005626 | 0.24 | | 0.926666667 | | 1.949016071 | - |
| bmsk0010519 | 17.6 | | 2.856666667 | | -2.62317082 | uncharacterized protein LOC106711973 isoform X1 [Papilio machaon] |
| bmsk0010645 | 7.006666667 | | 1.67 | | -2.068880161 | XP 004928288.1 28S ribosomal protein S2, mitochondrial |
| bmsk0014004 | 0.001 | | 0.35 | | 8.451211112 | XP 021204006.1 hemolin isoform X1 |
| bmsk0002097 | 7.37 | | 1.106666667 | | -2.735443878 | OTU domain-containing protein 6B |
| bmsk0002427 | 44.15666667 | | 12.26666667 | | -1.847888003 | nucleoporin Nup43 [Amyelois transitella] |
| bmsk0006436 | 16.52 | | 4.283333333 | | -1.947407828 | protein disulfide-isomerase TMX3 [Amyelois transitella] |
| bmsk0001391 | 2.533333333 | | 0.326666667 | | -2.955145764 | chitinase domain-containing protein 1 [Amyelois transitella] |
| mstrg.25588 | 4.953333333 | | 0.55 | | -3.170896186 | olfactory receptor [Bombyx mori] |
| bmsk0005159 | 11.29 | | 3.246666667 | | -1.798014309 | probable ubiquitin carboxyl-terminal hydrolase creB |
| bmsk0009444 | 0.001 | | 0.333333333 | | 8.380821784 | Phospholipase A2 |
| bmsk0010403 | 0.476666667 | | 0.97 | | 1.025004006 | ABF51376.1 eukaryotic translation initiation factor 3 subunit 5 |
| mstrg.983 | 16.30666667 | | 3.456666667 | | -2.23800851 | PREDICTED: E3 ubiquitin-protein ligase UHRF1-like isoform X1 [Bombyx mori] |
| bmsk0002438 | 22.42333333 | | 5.776666667 | | -1.956691604 | diptheria toxin resistance protein |
| mstrg.8172 | 10.31333333 | | 2.4 | | -2.103404385 | hypothetical protein g.7559 [Pectinophora gossypiella] |
| bmsk0000679 | 0.073333333 | | 0.563333333 | | 2.941447818 | - |
| mstrg.26541 | 13.10666667 | | 2.813333333 | | -2.219948418 | molybdenum cofactor sulfurase [Bombyx mori] |
| bmsk0005490 | 0.93 | | 2.463333333 | | 1.405309242 | - |
| bmsk0000275 | 13.97666667 | | 3.54 | | -1.981199064 | geminin isoform X2 [Amyelois transitella] |
| bmsk0007584 | 3.29 | | 0.363333333 | | -3.17872195 | Protein of unknown function (DUF812) |
| bmsk0000555 | 2.34 | | 0.24 | | -3.285402219 | glucuronyltransferase |
| bmsk0006648 | 16.69666667 | | 3.463333333 | | -2.269326957 | uncharacterized protein LOC101747195 |
| mstrg.16159 | 0.663333333 | | 1.856666667 | | 1.484908897 | olfactory receptor [Bombyx mori] |
| bmsk0007288 | 25.55333333 | | 6.736666667 | | -1.923404676 | protein abrupt isoform X1 |
| bmsk0003723 | 0.266666667 | | 0.743333333 | | 1.478971805 | leucokinin precursor |
| bmsk0002061 | 37.44666667 | | 12.49666667 | | -1.583293977 | major facilitator superfamily domain-containing protein 1-like isoform X1 [Amyelois transitella] |
| mstrg.10044 | 0.01 | | 0.31 | | 4.95419631 | - |
| bmsk0010890 | 0.636666667 | | 0.001 | | -9.314394422 | XP 004926205.1 transmembrane protein 209 |
| bmsk0008455 | 0.26 | | 0.001 | | -8.022367813 | fizzy-related protein homolog [Papilio machaon] |
| bmsk0009819 | 7.09 | | 1.28 | | -2.469641817 | leucine-rich repeat protein soc-2 homolog [Amyelois transitella] |
| bmsk0005693 | 0.363333333 | | 0.001 | | -8.505149919 | transcription initiation factor TFIID subunit 2 [Papilio xuthus] |
| bmsk0004580 | 6.66 | | 1.746666667 | | -1.930917866 | cold shock domain-containing protein CG9705 [Amyelois transitella] |
| mstrg.10599 | 0.94 | | 2.68 | | 1.511500339 | cuticular protein RR-1 motif 52 [Bombyx mori] |
| bmsk0013386 | 11.31333333 | | 2.016666667 | | -2.487979517 | UPF0414 transmembrane protein-like protein [Operophtera brumata] |
| bmsk0008018 | 1.496666667 | | 5.846666667 | | 1.965861398 | - |
| bmsk0010401 | 1.803333333 | | 0.023333333 | | -6.272129862 | uncharacterized protein LOC105386400 [Plutella xylostella] |
| bmsk0008689 | 21.50666667 | | 3.81 | | -2.496921035 | transcription termination factor 5, mitochondrial-like |
| bmsk0013351 | 5.213333333 | | 0.903333333 | | -2.528875756 | uncharacterized protein LOC107067961 isoform X2 [Polistes dominula] |
| bmsk0004103 | 12.54666667 | | 3.106666667 | | -2.013864768 | cyclin-H [Amyelois transitella] |
| bmsk0014312 | 0.001 | | 0.166666667 | | 7.380821784 | XP 012546088.1 uncharacterized protein LOC101743782 |
| bmsk0014816 | 2.206666667 | | 0.001 | | -11.107653 | XP 021202427.1 uncharacterized protein LOC101737526 |
| bmsk0005369 | 9.443333333 | | 2.226666667 | | -2.084410594 | death related ced-3/Nedd2-like protein |
| bmsk0007339 | 0.5 | | 1.203333333 | | 1.267036336 | alpha carbonic anhydrase 8 |
| bmsk0001727 | 0.15 | | 0.001 | | -7.22881869 | maestro heat-like repeat-containing protein family member 1 |
| mstrg.19359 | 0.163333333 | | 0.8 | | 2.292180751 | Yokozuna [Bombyx mori] |
| mstrg.19137 | 1.376666667 | | 0.073333333 | | -4.230566353 | PREDICTED: uncharacterized protein LOC106142350 [Amyelois transitella] |
| bmsk0012594 | 0.001 | | 0.33 | | 8.366322214 | cuticular protein RR-2 motif 111 precursor |
| bmsk0005420 | 34.53333333 | | 9.436666667 | | -1.871640349 | D -directed R polymerase II subunit RPB11 |
| bmsk0015762 | 0.853333333 | | 0.03 | | -4.830074999 | tektin-4-like |
| bmsk0012334 | 5.653333333 | | 1.46 | | -1.953133395 | XP 004925050.1 protein FAM172A |
| bmsk0003861 | 10.73 | | 2.596666667 | | -2.046917343 | E3 ubiquitin-protein ligase Arkadia |
| bmsk0006061 | 0.44 | | 0.001 | | -8.781359714 | Peptidase M1 N-terminal domain |
| bmsk0003205 | 0.293333333 | | 0.001 | | -8.196397213 | uncharacterized protein LOC106136756 [Amyelois transitella] |
| bmsk0009465 | 17.61 | | 3.843333333 | | -2.195964897 | uncharacterized protein LOC101743179 |
| bmsk0005578 | 27.48333333 | | 8.003333333 | | -1.77988409 | uncharacterized protein LOC106131814 [Amyelois transitella] |
| bmsk0002350 | 0.31 | | 0.713333333 | | 1.202308175 | uncharacterized protein LOC101741863 |
| bmsk0002647 | 0.083333333 | | 0.283333333 | | 1.765534746 | myosin-2 heavy chain |
| bmsk0001836 | 0.14 | | 2.783333333 | | 4.313314965 | - |
| bmsk0015477 | 0.003333333 | | 0.46 | | 7.108524457 | talin-2-like |
| bmsk0004921 | 11.69666667 | | 2.94 | | -1.992209387 | zinc finger protein 830 [Papilio polytes] |
| bmsk0005123 | 7.62 | | 1.776666667 | | -2.100617965 | BRISC and BRCA1-A complex member 1 isoform X2 |
| bmsk0010889 | 20.62666667 | | 4.55 | | -2.180572246 | ABF51274.1 AAA family ATPase |
| bmsk0014098 | 0.001 | | 0.26 | | 8.022367813 | acyl-CoA Delta(11) desaturase-like |
| bmsk0007544 | 0.47 | | 1.406666667 | | 1.581547836 | - |
| bmsk0007295 | 9.933333333 | | 1.816666667 | | -2.450984196 | uncharacterized protein CG5902 isoform X1 |
| bmsk0007019 | 15.47666667 | | 3.21 | | -2.269449578 | calcium/calmodulin-dependent protein kinase type 1 |
| bmsk0005806 | 5.976666667 | | 1.346666667 | | -2.14994829 | fibroin-modulator-binding protein-1 |
| bmsk0006202 | 20.82333333 | | 6.726666667 | | -1.630237355 | TATA element modulatory factor isoform X1 |
| bmsk0004556 | 0.066666667 | | 0.49 | | 2.87774425 | nuclear protein 1 |
| bmsk0012806 | 1.276666667 | | 0.246666667 | | -2.371747216 | calcium uptake protein 1, mitochondrial-like isoform X2 [Papilio polytes] |
| bmsk0009117 | 1.976666667 | | 0.016666667 | | -6.8899602 | Domain of unknown function (DUF1899) |
| bmsk0005898 | 11.46333333 | | 3.27 | | -1.809664074 | RING finger and WD repeat domain-containing protein 3 [Operophtera brumata] |
| bmsk0012222 | 256.3633333 | | 72.29333333 | | -1.826255416 | uncharacterized protein LOC103519968 isoform X3 [Diaphorina citri] |
| bmsk0008285 | 0.001 | | 0.273333333 | | 8.094517599 | rho GTPase-activating protein 6 |
| bmsk0009619 | 0.116666667 | | 0.001 | | -6.866248611 | uncharacterized protein LOC101739529 |
| bmsk0004452 | 27.63666667 | | 8.526666667 | | -1.696529855 | trafficking protein particle complex subunit 13-like [Amyelois transitella] |
| bmsk0002449 | 2.436666667 | | 0.38 | | -2.680837582 | arrestin domain-containing protein 4 |
| bmsk0010949 | 5.316666667 | | 0.883333333 | | -2.589492159 | origin recognition complex subunit 1 |
| bmsk0006398 | 0.343333333 | | 0.703333333 | | 1.034598662 | breast cancer anti-estrogen resistance protein 1 |
| mstrg.8014 | 25.21 | | 5.51 | | -2.193871894 | hypothetical protein g.14677 [Pectinophora gossypiella] |
| bmsk0007247 | 8.95 | | 1.77 | | -2.338138322 | R -binding protein 7 |
| bmsk0011685 | 10.27333333 | | 2.396666667 | | -2.099803186 | XP 004925346.1 sister chromatid cohesion protein DCC1 isoform X1 |
| bmsk0001258 | 25.76666667 | | 6.176666667 | | -2.060605533 | SET and MYND domain-containing protein 4 [Amyelois transitella] |
| bmsk0009425 | 3.046666667 | | 0.426666667 | | -2.836050355 | zinc finger protein 677-like |
| bmsk0002572 | 3.263333333 | | 0.553333333 | | -2.560125618 | dual specificity protein phosphatase 3 isoform X3 |
| bmsk0000266 | 1.52 | | 0.013333333 | | -6.832890014 | myosin-9-like isoform X4 |
| bmsk0009384 | 9.776666667 | | 1.666666667 | | -2.552377071 | transcription initiation protein SPT3 homolog [Amyelois transitella] |
| bmsk0010052 | 0.286666667 | | 0.001 | | -8.163230349 | double-time protein |
| bmsk0011577 | 1.286666667 | | 0.206666667 | | -2.638260727 | sister chromatid cohesion protein PDS5 homolog B-B isoform X1 |
| bmsk0015804 | 10.21333333 | | 2.223333333 | | -2.199657631 | XP 021208540.1 TIMELESS-interacting protein |
| bmsk0013628 | 1.563333333 | | 0.286666667 | | -2.447179358 | DH dehydrogenase [ubiquinone] 1 alpha subcomplex assembly factor 5 |
| bmsk0000707 | 0.25 | | 1.04 | | 2.056583528 | - |
| bmsk0003052 | 3.253333333 | | 0.466666667 | | -2.801454321 | scm-like with four MBT domains protein 2 isoform X1 |
| bmsk0014118 | 9.913333333 | | 3.146666667 | | -1.655545883 | gastrula zinc finger protein XlCGF49.1-like |
| bmsk0011159 | 0.083333333 | | 0.19 | | 1.189033824 | chemosensory protein 11 isoform X1 |
| bmsk0010172 | 0.216666667 | | 0.001 | | -7.759333407 | D topoisomerase 3-alpha |
| bmsk0014709 | 17.25333333 | | 4.18 | | -2.045300269 | - |
| bmsk0014271 | 37.62333333 | | 9.08 | | -2.050863471 | vacuolar protein sorting-associated protein 26B-like [Amyelois transitella] |
| bmsk0006475 | 16.50333333 | | 3.696666667 | | -2.158460584 | tether containing UBX domain for GLUT4 |
| bmsk0000188 | 7.713333333 | | 2.19 | | -1.816423589 | syntaxin-7 [Amyelois transitella] |
| bmsk0014280 | 24.89 | | 8.086666667 | | -1.621949181 | uncharacterized protein LOC101744111 |
| bmsk0012375 | 0.036666667 | | 0.833333333 | | 4.506352666 | - |
| bmsk0003311 | 22.22 | | 5.096666667 | | -2.124232911 | leucine-rich repeat protein soc-2 homolog [Papilio xuthus] |
| bmsk0011286 | 16.92666667 | | 4.8 | | -1.818191583 | XP 004922454.1 WD repeat-containing protein 91 isoform X1 |
| bmsk0005238 | 0.1 | | 0.496666667 | | 2.312277925 | - |
| bmsk0012543 | 5.75 | | 0.553333333 | | -3.377341215 | XP 021207491.1 lysine-specific demethylase 8 |
| bmsk0009861 | 29.40333333 | | 7.496666667 | | -1.971658556 | putative mitogen-activated protein kinase (MAPKK) [Heliconius melpomene] |
| bmsk0007136 | 0.001 | | 0.733333333 | | 9.518325308 | histidine-rich glycoprotein [Athalia rosae] |
| bmsk0000600 | 0.713333333 | | 1.553333333 | | 1.122719158 | - |
| bmsk0000628 | 0.713333333 | | 1.553333333 | | 1.122719158 | - |
| bmsk0005218 | 0.001 | | 0.306666667 | | 8.26052755 | phospholipase B1, membrane-associated-like |
| mstrg.13773 | 12.17666667 | | 2.346666667 | | -2.37543442 | PREDICTED: D-amino-acid oxidase [Bombyx mori] |
| bmsk0004251 | 3.016666667 | | 0.736666667 | | -2.033871423 | conventional protein kinase C |
| mstrg.1471 | 32.44 | | 8.983333333 | | -1.852451047 | PREDICTED: LOW QUALITY PROTEIN: mitochondrial Rho GTPase-like [Bombyx mori] |
| bmsk0012933 | 6.32 | | 0.21 | | -4.911463325 | kinesin-like protein KIF18A isoform X1 |
| bmsk0008609 | 0.396666667 | | 1.29 | | 1.701371993 | Amino acid permease |
| bmsk0015088 | 10.47333333 | | 2.966666667 | | -1.819805939 | XP 004933015.1 upstream activation factor subunit spp27 |
| bmsk0009859 | 44.02666667 | | 11.93666667 | | -1.882977602 | cytokine receptor |
| bmsk0014495 | 0.086666667 | | 8.813333333 | | 6.668066743 | XP 021203363.1 carboxyl/cholinesterase 6 isoform X1 |
| bmsk0012352 | 0.02 | | 0.276666667 | | 3.790076931 | uncharacterized family 31 glucosidase KIAA1161-like [Papilio polytes] |
| bmsk0006918 | 0.001 | | 0.643333333 | | 9.329422631 | histidine-rich glycoprotein [Athalia rosae] |
| bmsk0003892 | 10.50333333 | | 2.343333333 | | -2.16421316 | mediator of R polymerase II transcription subunit 29 |
| mstrg.23401 | 4.28 | | 0.953333333 | | -2.16655815 | PREDICTED: ankyrin-3-like [Amyelois transitella] |
| bmsk0003101 | 2.75 | | 0.466666667 | | -2.558967292 | scavenger receptor class B member 1-like isoform X1 |
| bmsk0013237 | 27.36666667 | | 5.46 | | -2.325446865 | nuclear receptor-binding factor 2-like [Papilio polytes] |
| bmsk0014939 | 5.66 | | 0.533333333 | | -3.407692649 | Rad51 homolog |
| bmsk0011137 | 1.506666667 | | 0.37 | | -2.025763096 | transmembrane protein adipocyte-associated 1 homolog isoform X2 |
| bmsk0010046 | 13.18666667 | | 3.506666667 | | -1.910907721 | nudix hydrolase 8-like [Amyelois transitella] |
| bmsk0008683 | 7.866666667 | | 1.633333333 | | -2.267933205 | D replication complex GINS protein SLD5 |
| bmsk0002854 | 0.17 | | 0.001 | | -7.409390936 | lamin-C |
| bmsk0002140 | 1.966666667 | | 0.256666667 | | -2.937784604 | pre-mR -splicing factor CWC25 homolog isoform X1 [Amyelois transitella] |
| bmsk0012706 | 0.001 | | 0.186666667 | | 7.544320516 | elastase-1 |
| mstrg.13596 | 6.923333333 | | 1.696666667 | | -2.028763655 | PREDICTED: protein prenyltransferase alpha subunit repeat-containing protein 1 [Bombyx mori] |
| bmsk0011547 | 0.223333333 | | 0.663333333 | | 1.57053543 | 30K protein 20, partial |
| bmsk0009424 | 3.213333333 | | 0.623333333 | | -2.365994876 | FancJ-like protein |
| bmsk0014233 | 6.256666667 | | 1.486666667 | | -2.073313035 | XP 012552957.1 zinc finger protein 718 isoform X2 |
| bmsk0014778 | 4.29 | | 0.62 | | -2.790637527 | intron-binding protein aquarius [Amyelois transitella] |
| bmsk0014027 | 0.573333333 | | 0.013333333 | | -5.426264755 | XP 012546288.1 zinc finger protein 136 isoform X4 |
| mstrg.6998 | 22.32333333 | | 6.116666667 | | -1.867734905 | PREDICTED: COMM domain-containing protein 2-like isoform X3 [Bombyx mori] |
| bmsk0015497 | 0.096666667 | | 0.523333333 | | 2.436639754 | XP 021201890.1 uncharacterized protein LOC101737983 isoform X7 |
| bmsk0008361 | 9.366666667 | | 2.536666667 | | -1.884601772 | bromodomain-containing protein DDB G0280777-like [Amyelois transitella] |
| bmsk0010851 | 2.563333333 | | 0.46 | | -2.478315331 | XP 004926993.1 CCR4-NOT transcription complex subunit 11 |
| bmsk0000626 | 0.063333333 | | 0.846666667 | | 3.740757173 | uncharacterized protein LOC101736996 isoform X2 |
| bmsk0013283 | 12.87333333 | | 2.236666667 | | -2.524963493 | ras-related GTP-binding protein 4b |
| bmsk0007282 | 0.001 | | 0.166666667 | | 7.380821784 | - |
| bmsk0003811 | 9.28 | | 2.123333333 | | -2.127793934 | golgin subfamily A member 5 [Amyelois transitella] |
| bmsk0005804 | 0.923333333 | | 0.133333333 | | -2.791814071 | mediator of R polymerase II transcription subunit 9 [Amyelois transitella] |
| bmsk0001265 | 0.04 | | 0.253333333 | | 2.662965013 | PHD-finger |
| bmsk0005299 | 8.916666667 | | 1.993333333 | | -2.161321502 | tumor protein p63-regulated gene 1-like protein isoform X1 |
| bmsk0012590 | 0.001 | | 0.696666667 | | 9.444324726 | cuticular protein RR-2 motif 115 precursor |
| bmsk0009445 | 0.013333333 | | 0.306666667 | | 4.523561956 | phospholipase A2-like |
| bmsk0003441 | 0.283333333 | | 0.001 | | -8.14635653 | angiotensin-converting enzyme isoform X1 |
| bmsk0003150 | 0.001 | | 0.82 | | 9.6794801 | Eukaryotic-type carbonic anhydrase |
| bmsk0009839 | 21.76 | | 6.68 | | -1.703758549 | dual specificity mitogen-activated protein kinase kinase 6 |
| bmsk0010166 | 0.001 | | 0.5 | | 8.965784285 | zinc finger protein 583-like isoform X1 |
| bmsk0001929 | 24.00666667 | | 7.25 | | -1.727382199 | integrator complex subunit 2 [Amyelois transitella] |
| bmsk0005669 | 10.22333333 | | 2.943333333 | | -1.796342824 | uncharacterized protein LOC106139923 [Amyelois transitella] |
| bmsk0014114 | 13.93666667 | | 3.43 | | -2.022605061 | protein phosphatase 1 regulatory subunit 3B-B [Amyelois transitella] |
| mstrg.16241 | 16.49333333 | | 3.586666667 | | -2.201167422 | Zinc finger protein 26, partial [Pararge aegeria] |
| bmsk0011290 | 23.71 | | 6.973333333 | | -1.765575312 | tumor susceptibility gene 101 protein [Amyelois transitella] |
| mstrg.597 | 11.50333333 | | 2.846666667 | | -2.014706499 | PREDICTED: probable palmitoyltransferase ZDHHC16 [Bombyx mori] |
| bmsk0010675 | 0.696666667 | | 0.103333333 | | -2.753162822 | serine/threonine-protein phosphatase 4 regulatory subunit 2 [Amyelois transitella] |
| bmsk0011097 | 0.18 | | 0.001 | | -7.491853096 | farnesyl diphosphate synthase 3 isoform X1 |
| bmsk0014145 | 1.05 | | 0.156666667 | | -2.744619167 | - |
| bmsk0014487 | 44.90666667 | | 10.97666667 | | -2.032489626 | pinin [Amyelois transitella] |
| bmsk0014716 | 0.001 | | 0.26 | | 8.022367813 | 4-coumarate--CoA ligase 1-like |
| bmsk0010960 | 14.91666667 | | 3.396666667 | | -2.134733631 | putative Lancl1 protein [Danaus plexippus] |
| bmsk0013230 | 12.39666667 | | 2.846666667 | | -2.122606773 | translation initiation factor 2 gamma subunit |
| bmsk0011970 | 0.001 | | 0.32 | | 8.321928095 | XP 004925452.2 uncharacterized protein LOC101737101 isoform X1 |
| bmsk0001023 | 0.001 | | 0.82 | | 9.6794801 | Cysteine dioxygenase type I |
| bmsk0004647 | 25.33666667 | | 7.793333333 | | -1.700914304 | cyclin dependent kinase 12 isoform X1 |
| mstrg.19860 | 6.506666667 | | 1.783333333 | | -1.867342256 | juvenile hormone esterase binding protein [Bombyx mori] |
| bmsk0014704 | 1.45 | | 0.14 | | -3.372554168 | XP 004924552.1 TRAF3-interacting protein 1 |
| bmsk0008668 | 0.04 | | 0.11 | | 1.459431619 | uncharacterized protein LOC105842023 |
| bmsk0001460 | 11.84 | | 3.356666667 | | -1.818567898 | ubiA prenyltransferase domain-containing protein 1 homolog |
| bmsk0003917 | 0.22 | | 0.606666667 | | 1.463400521 | putative transcription factor capicua |
| mstrg.10249 | 3.086666667 | | 0.68 | | -2.182443041 | PREDICTED: proline-rich protein 11-like [Amyelois transitella] |
| bmsk0010596 | 3.99 | | 0.89 | | -2.164511505 | uncharacterized protein LOC101744659 isoform X1 |
| bmsk0005470 | 1.32 | | 0.14 | | -3.237039197 | BTB/POZ domain-containing adapter for CUL3-mediated RhoA degradation protein 3 |
| mstrg.22680 | 36.91333333 | | 11.68 | | -1.660101747 | PREDICTED: snurportin-1 [Bombyx mori] |
| bmsk0009447 | 0.001 | | 0.286666667 | | 8.163230349 | phospholipase A2-like |
| bmsk0002525 | 8.706666667 | | 2.573333333 | | -1.758482144 | peptidylglycine alpha-hydroxylating monooxygenase isoform X1 [Amyelois transitella] |
| bmsk0011306 | 0.001 | | 0.216666667 | | 7.759333407 | putative phosphatidate phosphatase [Papilio xuthus] |
| bmsk0015682 | 2.733333333 | | 0.723333333 | | -1.917928867 | glycosyltransferase isoform X1 |
| bmsk0005362 | 13.22 | | 4.5 | | -1.55472527 | zinc finger CCHC domain-containing protein 8 homolog |
| bmsk0011921 | 9.07 | | 2.72 | | -1.737495899 | Protein SDE2-like protein [Operophtera brumata] |
| bmsk0008780 | 10.1 | | 2.426666667 | | -2.057307438 | cyclin-C |
| bmsk0003099 | 0.013333333 | | 0.1 | | 2.906890596 | scavenger receptor class B member 1-like isoform X1 |
| bmsk0006166 | 11.52 | | 3.46 | | -1.735296774 | Motile sperm domain-containing protein [Operophtera brumata] |
| bmsk0015499 | 0.06 | | 0.863333333 | | 3.846883286 | microtubule-associated serine/threonine-protein kinase 3 isoform X3 |
| bmsk0006816 | 5.373333333 | | 0.716666667 | | -2.906443179 | peroxiredoxin |
| bmsk0015955 | 0.07 | | 0.436666667 | | 2.641105579 | - |
| bmsk0006293 | 0.013333333 | | 0.456666667 | | 5.098032083 | elongation of very long chain fatty acids protein 7-like isoform X2 |
| bmsk0011417 | 6.23 | | 1.49 | | -2.063919833 | XP 004926953.3 NF-X1-type zinc finger protein NFXL1 |
| bmsk0008656 | 1.563333333 | | 0.27 | | -2.53359411 | collagen alpha-2(VIII) chain |
| bmsk0015754 | 3.186666667 | | 0.636666667 | | -2.32343798 | hypothetical protein KGM 05116 [Danaus plexippus] |
| bmsk0012253 | 4.386666667 | | 0.166666667 | | -4.718087584 | uncharacterized protein LOC106129070 [Amyelois transitella] |
| bmsk0008191 | 0.233333333 | | 0.001 | | -7.866248611 | myosin light chain kinase, smooth muscle-like [Amyelois transitella] |
| bmsk0005910 | 4.223333333 | | 0.59 | | -2.839595259 | R -binding protein 48 |
| bmsk0005604 | 0.09 | | 0.213333333 | | 1.245112498 | tyrosine decarboxylase [Amyelois transitella] |
| bmsk0015926 | 0.001 | | 0.17 | | 7.409390936 | uncharacterized protein LOC106110594 [Papilio polytes] |
| bmsk0000002 | 0.001 | | 0.226666667 | | 7.824428435 | anion exchange protein 3 isoform X1 |
| bmsk0003566 | 0.001 | | 0.073333333 | | 6.196397213 | rootletin |
| bmsk0014116 | 0.036666667 | | 0.286666667 | | 2.966833136 | - |
| bmsk0014701 | 0.19 | | 0.41 | | 1.109624491 | XP 021202580.1 endochitinase A isoform X1 |
| bmsk0011010 | 7.026666667 | | 2.253333333 | | -1.640779715 | PX domain-containing protein kinase-like protein |
| bmsk0007122 | 11.41 | | 2.226666667 | | -2.357341285 | rab3 GTPase-activating protein regulatory subunit |
| bmsk0014796 | 0.403333333 | | 1.233333333 | | 1.612518223 | transient receptor potential channel pyrexia |
| bmsk0007763 | 16.57 | | 4.853333333 | | -1.771525748 | ER degradation-enhancing alpha-mannosidase-like protein 2 isoform X1 |
| bmsk0010597 | 13.87333333 | | 3.716666667 | | -1.900233255 | XP 021205066.1 protein SMG9 |
| bmsk0012661 | 52.58 | | 13.83333333 | | -1.926365306 | XP 021207427.1 ca2 /calmodulin-dependent protein kinase II isoform X1 |
| bmsk0010333 | 5.766666667 | | 1.376666667 | | -2.066558351 | deoxynucleoside kinase-like isoform X1 [Papilio polytes] |
| bmsk0005809 | 2.993333333 | | 0.703333333 | | -2.089472446 | D polymerase eta isoform X1 |
| bmsk0007893 | 1.943333333 | | 0.023333333 | | -6.379997151 | - |
| bmsk0007692 | 14.91 | | 3.783333333 | | -1.978550461 | CRAL-TRIO domain-containing protein C3H8.02 [Papilio xuthus] |
| bmsk0007099 | 12.23 | | 2.716666667 | | -2.17051494 | uncharacterized protein LOC101739358 isoform X1 |
| bmsk0007771 | 1.116666667 | | 0.22 | | -2.343623166 | thymidylate synthase isoform 1 |
| bmsk0010002 | 0.083333333 | | 1.096666667 | | 3.718087584 | inactive rhomboid protein 1, partial |
| bmsk0011610 | 9.17 | | 2.246666667 | | -2.029135643 | Uncharacterized protein OBRU01 00296 [Operophtera brumata] |
| mstrg.25168 | 4.96 | | 1.043333333 | | -2.249139964 | PREDICTED: importin subunit alpha-2 isoform X1 [Bombyx mori] |
| bmsk0003711 | 17.07666667 | | 4.82 | | -1.82492134 | ANTH domain |
| mstrg.9809 | 5.736666667 | | 1.413333333 | | -2.021110927 | PREDICTED: uncharacterized protein LOC103307937 [Acyrthosiphon pisum] |
| bmsk0015467 | 15.59333333 | | 4.216666667 | | -1.886754477 | XP 004926638.1 UPF0428 protein CXorf56 homolog |
| bmsk0013797 | 1.27 | | 0.096666667 | | -3.715666192 | XP 021202005.1 uncharacterized protein LOC101744639 isoform X1 |
| bmsk0001677 | 1.603333333 | | 0.001 | | -10.64685868 | uncharacterized protein LOC110385729 |
| bmsk0008348 | 15.39666667 | | 4.65 | | -1.727315424 | nudC domain-containing protein 1 [Amyelois transitella] |
| bmsk0014665 | 16.71666667 | | 5.263333333 | | -1.66723853 | XP 012553133.1 DDB1- and CUL4-associated factor 5 isoform X1 |
| bmsk0011445 | 0.001 | | 0.736666667 | | 9.524868154 | Lepidopteran low molecular weight (30 kD) lipoprotein |
| bmsk0015851 | 9.666666667 | | 2.873333333 | | -1.750293126 | juvenile hormone epoxide hydrolase-like protein 3 isoform X1 |
| bmsk0013168 | 14.02666667 | | 4.583333333 | | -1.613703086 | Retrovirus-related Pol polyprotein from transposon TNT 1-94 [Papilio machaon] |
| bmsk0007139 | 8.25 | | 2.29 | | -1.849046521 | putative ATP-dependent R helicase me31b |
| bmsk0010009 | 0.001 | | 0.25 | | 7.965784285 | uncharacterized protein LOC106119184 [Papilio xuthus] |
| bmsk0004839 | 0.59 | | 0.08 | | -2.882643049 | uncharacterized protein LOC101746412 isoform X2 |
| bmsk0000923 | 3.57 | | 0.583333333 | | -2.613531653 | hepatoma-derived growth factor-related protein 3 isoform X2 |
| bmsk0007259 | 0.001 | | 0.043333333 | | 5.437405312 | retinol dehydrogenase 12-like [Anolis carolinensis] |
| bmsk0008332 | 2.943333333 | | 0.556666667 | | -2.402565335 | protein OPI10 homolog [Amyelois transitella] |
| bmsk0011487 | 4.183333333 | | 0.136666667 | | -4.935919644 | microtubule-actin cross-linking factor 1, isoforms 1/2/3/5 [Amyelois transitella] |
| bmsk0005037 | 0.76 | | 0.036666667 | | -4.373458396 | coronin-2B-like isoform X1 |
| bmsk0007102 | 75.57 | | 20.77666667 | | -1.862849411 | CRM1 [Spodoptera frugiperda] |
| bmsk0001732 | 5.553333333 | | 1.253333333 | | -2.147583834 | TBC1 domain family member 24 isoform X1 |
| bmsk0013481 | 0.163333333 | | 0.973333333 | | 2.575114715 | GRAM domain-containing protein 1B-like isoform X1 |
| bmsk0011163 | 211.6766667 | | 55.28333333 | | -1.936945737 | ABH88200.1 chemosensory protein 7 |
| bmsk0014486 | 18.77333333 | | 5.6 | | -1.745186101 | XP 004925700.1 ADP-ribosylation factor-like protein 2 |
| bmsk0001485 | 0.03 | | 0.456666667 | | 3.928107082 | trypsin-1-like |
| bmsk0014211 | 1.573333333 | | 0.04 | | -5.297680549 | XP 004923155.1 ADP-ribosylation factor-like protein 13B |
| bmsk0012269 | 1.08 | | 0.001 | | -10.0768156 | XP 021206731.1 uncharacterized protein LOC105842286 |
| bmsk0004648 | 0.116666667 | | 0.28 | | 1.263034406 | myosin-IB isoform X3 |
| bmsk0014247 | 0.116666667 | | 0.553333333 | | 2.245756414 | ABW86946.1 sex peptide receptor |
| bmsk0002630 | 3.903333333 | | 0.543333333 | | -2.844797206 | metallo-beta-lactamase domain-containing protein 1 |
| bmsk0000408 | 0.001 | | 0.21 | | 7.714245518 | dendritic arbor reduction protein 1 |
| bmsk0012588 | 0.001 | | 0.546666667 | | 9.094517599 | Insect cuticle protein |
| bmsk0013279 | 4.763333333 | | 1.23 | | -1.953313195 | protein FRA10AC1 homolog isoform X2 [Amyelois transitella] |
| bmsk0009471 | 6.323333333 | | 0.803333333 | | -2.976614627 | cell cycle checkpoint control protein RAD9A [Amyelois transitella] |
| bmsk0006329 | 13.58666667 | | 4.003333333 | | -1.7629179 | maternal protein tudor-like isoform X3 [Amyelois transitella] |
| bmsk0008790 | 0.126666667 | | 0.293333333 | | 1.211504105 | uncharacterized protein LOC101735403 |
| bmsk0000961 | 1.496666667 | | 0.24 | | -2.640646633 | uncharacterized protein LOC100902684 [Metaseiulus occidentalis] |
| bmsk0002833 | 0.456666667 | | 0.036666667 | | -3.638600464 | uncharacterized protein LOC101740833 |
| bmsk0007854 | 0.153333333 | | 0.001 | | -7.26052755 | uncharacterized protein LOC106105015 [Papilio polytes] |
| bmsk0012723 | 1.87 | | 0.043333333 | | -5.431417242 | - |
| bmsk0012614 | 0.01 | | 0.283333333 | | 4.824428435 | cuticle protein 7-like [Plutella xylostella] |
| bmsk0009270 | 30.51333333 | | 8.746666667 | | -1.802634572 | elongator complex protein 5 |
| bmsk0000112 | 1.736666667 | | 0.466666667 | | -1.895856545 | bromodomain-containing protein DDB G0280777-like |
| bmsk0005024 | 0.943333333 | | 0.13 | | -2.859256024 | uncharacterized protein LOC101735733 |
| bmsk0001867 | 1.063333333 | | 0.04 | | -4.732450113 | transcription factor glial cells missing 2-like |
| bmsk0009364 | 11.70333333 | | 0.04 | | -8.19270378 | centromere protein J [Papilio polytes] |
| bmsk0001579 | 3.956666667 | | 0.416666667 | | -3.247319935 | blastopia polyprotein [Lasius niger] |
| bmsk0008919 | 13.17666667 | | 3.633333333 | | -1.85861982 | mR cap guanine-N7 methyltransferase [Papilio machaon] |
| bmsk0000752 | 0.016666667 | | 0.096666667 | | 2.5360529 | androgen-dependent TFPI-regulating protein-like isoform X2 |
| bmsk0006449 | 1.336666667 | | 0.12 | | -3.477533425 | wiskott-Aldrich syndrome protein family member 2 |
| bmsk0002872 | 16.05333333 | | 3.643333333 | | -2.139541991 | 6-pyruvoyltetrahydropterin synthase |
| bmsk0014441 | 1.176666667 | | 0.106666667 | | -3.463524373 | uncharacterized protein LOC106138591 [Amyelois transitella] |
| bmsk0006427 | 3.23 | | 0.553333333 | | -2.545313424 | N-alpha-acetyltransferase 60 isoform X2 |
| mstrg.9119 | 16.09666667 | | 4.14 | | -1.95905929 | DNA polymerase accessory subunit [Bombyx mori] |
| bmsk0002355 | 0.001 | | 0.256666667 | | 8.003752135 | - |
| bmsk0005996 | 1.166666667 | | 0.273333333 | | -2.093659107 | calmodulin-like protein 4 [Papilio xuthus] |
| bmsk0009344 | 0.113333333 | | 0.71 | | 2.647246779 | glucose dehydrogenase [FAD, quinone] isoform X1 |
| bmsk0006627 | 1.036666667 | | 0.1 | | -3.373880175 | neuropeptide receptor A10 |
| bmsk0009172 | 0.316666667 | | 1.483333333 | | 2.227805918 | Uncharacterized protein OBRU01 06884 [Operophtera brumata] |
| bmsk0002693 | 19.76666667 | | 6.116666667 | | -1.692252042 | splicing factor 45 |
| mstrg.9990 | 0.283333333 | | 0.983333333 | | 1.795180208 | olfactory receptor [Bombyx mori] |
| bmsk0004743 | 0.243333333 | | 1.216666667 | | 2.321928095 | tyrosine kinase receptor Cad96Ca isoform X2 [Amyelois transitella] |
| bmsk0005899 | 0.67 | | 0.08 | | -3.06608919 | - |
| bmsk0001482 | 0.056666667 | | 0.243333333 | | 2.102361718 | neuropeptide receptor A34 |
| mstrg.6810 | 21.84333333 | | 6.563333333 | | -1.73469242 | ubiquinone biosynthesis protein COQ4 homolog, mitochondrial [Bombyx mori] |
| mstrg.11071 | 11.99 | | 2.6 | | -2.20524813 | CG8818, partial [Drosophila busckii] |
| bmsk0007860 | 2.446666667 | | 0.483333333 | | -2.339727163 | growth arrest and D damage-inducible proteins-interacting protein 1 isoform X2 |
| bmsk0012632 | 11.24 | | 2.26 | | -2.314247358 | gonadal protein gdl [Papilio polytes] |
| bmsk0005942 | 8.196666667 | | 1.06 | | -2.950973064 | uncharacterized protein LOC101740704, partial |
| bmsk0006424 | 13.09333333 | | 2.813333333 | | -2.218480026 | aprataxin-like protein |
| bmsk0008672 | 71.63333333 | | 22.14333333 | | -1.693758663 | uncharacterized protein CG16817-like |
| mstrg.9536 | 44.42 | | 14.06 | | -1.659612798 | BRCA1-associated ATM activator 1, partial [Pararge aegeria] |
| bmsk0008577 | 1.656666667 | | 0.413333333 | | -2.002905731 | myotubularin-related protein 2 |
| bmsk0003712 | 0.253333333 | | 1.163333333 | | 2.199155713 | phosphatidylinositol-binding clathrin assembly protein LAP isoform X2 |
| bmsk0004501 | 134.6866667 | | 44.64333333 | | -1.593090382 | ALY |
| bmsk0009131 | 0.426666667 | | 0.033333333 | | -3.678071905 | uncharacterized protein LOC105842042 |
| bmsk0009997 | 46.04 | | 14.77333333 | | -1.639892453 | DRK |
| bmsk0007297 | 0.07 | | 0.001 | | -6.129283017 | remodeling and spacing factor 1-like |
| bmsk0006175 | 0.123333333 | | 0.001 | | -6.94641896 | extensin-2-like |
| bmsk0006228 | 0.001 | | 0.363333333 | | 8.505149919 | bombyxin C-1 precursor |
| bmsk0006093 | 1.106666667 | | 0.001 | | -10.11200503 | - |
| bmsk0015290 | 0.01 | | 0.28 | | 4.807354922 | antennal binding protein X precursor |
| bmsk0002439 | 3.106666667 | | 0.69 | | -2.170699187 | coiled-coil domain-containing protein 124 |
| bmsk0001191 | 1.46 | | 0.183333333 | | -2.993427346 | zinc finger protein 615 isoform X4 |
| bmsk0003350 | 0.813333333 | | 0.02 | | -5.345774837 | protein tramtrack, beta isoform |
| bmsk0000584 | 13.09333333 | | 3.813333333 | | -1.779707878 | uncharacterized protein LOC101736297 |
| bmsk0014408 | 0.126666667 | | 0.001 | | -6.984893108 | probable tyrosyl-D phosphodiesterase [Amyelois transitella] |
| bmsk0007838 | 0.246666667 | | 0.001 | | -7.94641896 | - |
| bmsk0006212 | 0.26 | | 0.001 | | -8.022367813 | uncharacterized protein C18orf8 |
| bmsk0013173 | 1.64 | | 0.07 | | -4.550197083 | protein inturned isoform X2 |
| bmsk0010508 | 0.08 | | 0.206666667 | | 1.36923381 | Uncharacterized protein OBRU01 14771 [Operophtera brumata] |
| bmsk0007318 | 0.773333333 | | 0.06 | | -3.688055994 | hypothetical protein TcasGA2 TC001839 [Tribolium castaneum] |
| bmsk0011050 | 21.13 | | 6.463333333 | | -1.708942465 | putative ribosome-binding factor A, mitochondrial isoform X2 |
| bmsk0005847 | 0.96 | | 0.15 | | -2.678071905 | - |
| bmsk0015842 | 2.21 | | 0.48 | | -2.202940059 | uncharacterized protein LOC101735769 |
| bmsk0004186 | 0.06 | | 0.13 | | 1.115477217 | lipase 1-like |
| bmsk0014142 | 1.11 | | 0.183333333 | | -2.598018654 | - |
| bmsk0006799 | 2.273333333 | | 0.18 | | -3.658740427 | - |
| bmsk0013974 | 2.273333333 | | 0.18 | | -3.658740427 | Uncharacterized protein OBRU01 10407 [Operophtera brumata] |
| bmsk0006642 | 0.736666667 | | 0.04 | | -4.202940059 | protein FAM195A-like [Plutella xylostella] |
| bmsk0012339 | 16.06666667 | | 2.456666667 | | -2.709296622 | XP 004925056.1 conserved oligomeric Golgi complex subunit 5 |
| bmsk0015761 | 0.803333333 | | 0.046666667 | | -4.105534414 | testis specific tektin |
| bmsk0004444 | 16.43 | | 4.563333333 | | -1.848172534 | cytochrome P450 CYP354A3 [Helicoverpa armigera] |
| bmsk0003949 | 0.026666667 | | 0.953333333 | | 5.159871337 | tight junction protein ZO-1 isoform X13 |
| bmsk0003328 | 0.726666667 | | 0.001 | | -9.505149919 | Hox cluster protein Shx15 |
| bmsk0006912 | 0.033333333 | | 0.163333333 | | 2.292781749 | endoplasmic reticulum lectin 1 isoform X1 [Amyelois transitella] |
| bmsk0010045 | 0.786666667 | | 0.146666667 | | -2.423211431 | uncharacterized protein LOC105841590 isoform X1 |
| bmsk0004891 | 0.783333333 | | 0.001 | | -9.613482541 | - |
| bmsk0002443 | 0.56 | | 0.02 | | -4.807354922 | unconventional prefoldin RPB5 interactor [Amyelois transitella] |
| bmsk0008839 | 3.833333333 | | 0.506666667 | | -2.919490632 | hypothetical protein M513 07274 [Trichuris suis] |
| bmsk0008148 | 0.816666667 | | 0.086666667 | | -3.236198221 | Uncharacterized protein OBRU01 08256 [Operophtera brumata] |
| bmsk0002213 | 3.52 | | 0.72 | | -2.289506617 | rab3 GTPase-activating protein catalytic subunit [Papilio polytes] |
| bmsk0003962 | 0.001 | | 0.096666667 | | 6.594946589 | UDP-glucoronosyl and UDP-glucosyl transferase |
| bmsk0007859 | 1.06 | | 0.14 | | -2.920565533 | Protein Cep78-like [Papilio machaon] |
| bmsk0011813 | 0.426666667 | | 0.001 | | -8.736965594 | AAU12844.1 vitellogenin receptor, partial |
| mstrg.22526 | 44.35666667 | | 12.94333333 | | -1.776941747 | PREDICTED: RNA-binding protein 12-like isoform X3 [Papilio polytes] |
| bmsk0013749 | 0.79 | | 0.15 | | -2.396890153 | sid-1-related gene1 precursor |
| bmsk0002791 | 1.51 | | 0.23 | | -2.714842783 | MSL2 protein |
| bmsk0011400 | 6.17 | | 1.303333333 | | -2.243064383 | XP 004927389.1 uroporphyrinogen-III synthase |
| bmsk0004497 | 27.19 | | 7.03 | | -1.951479557 | histone R hairpin-binding protein |
| bmsk0007142 | 3.58 | | 0.596666667 | | -2.584962501 | uncharacterized protein LOC101743024 isoform X2 |
| bmsk0008318 | 0.286666667 | | 1.21 | | 2.077560983 | Kinesin motor domain |
| bmsk0007132 | 25.01 | | 8.133333333 | | -1.62058641 | TATA-box-binding protein |
| bmsk0014440 | 7.77 | | 2.063333333 | | -1.91293769 | segment polarity protein dishevelled, partial |
| bmsk0003951 | 0.03 | | 0.36 | | 3.584962501 | calcium-binding protein P, partial |
| bmsk0012916 | 0.223333333 | | 0.646666667 | | 1.533823652 | elongation of very long chain fatty acids protein 7-like [Amyelois transitella] |
| bmsk0010047 | 1.233333333 | | 0.18 | | -2.776493958 | nudix hydrolase 8 isoform X3 |
| bmsk0015384 | 0.616666667 | | 9.626666667 | | 3.964473566 | uncharacterized protein LOC105390186 [Plutella xylostella] |
| bmsk0006817 | 0.03 | | 0.146666667 | | 2.289506617 | uncharacterized protein LOC101742066 |
| bmsk0011998 | 0.176666667 | | 0.78 | | 2.142444265 | myotubularin-related protein 10-A |
| bmsk0003326 | 0.336666667 | | 0.001 | | -8.395177077 | uncharacterized protein LOC101742071 |
| bmsk0001083 | 2.09 | | 0.25 | | -3.063502942 | uncharacterized protein LOC106713191 [Papilio machaon] |
| bmsk0003376 | 17.89333333 | | 4.866666667 | | -1.878416302 | transcription elongation factor B polypeptide 3 [Papilio xuthus] |
| bmsk0011872 | 27.97 | | 8.74 | | -1.678175069 | XP 004926025.1 digestive cysteine proteinase 1 isoform X2 |
| bmsk0010832 | 0.396666667 | | 0.056666667 | | -2.807354922 | BAG family molecular chaperone regulator 2-like isoform X1 |
| bmsk0009353 | 0.001 | | 0.23 | | 7.845490051 | vacuolar protein sorting-associated protein 33A-like |
| bmsk0013392 | 0.11 | | 0.583333333 | | 2.406816992 | diuretic hormone 31 precursor |
| bmsk0008955 | 0.286666667 | | 0.001 | | -8.163230349 | periodic tryptophan protein 1 homolog [Amyelois transitella] |
| bmsk0011705 | 2.53 | | 0.383333333 | | -2.722466024 | - |
| bmsk0003878 | 3.18 | | 0.683333333 | | -2.218365357 | uncharacterized protein LOC101745257 isoform X1 |
| bmsk0011526 | 0.623333333 | | 0.056666667 | | -3.459431619 | XP 021207167.1 intraflagellar transport protein 52 homolog isoform X2 |
| bmsk0011263 | 1.48 | | 0.143333333 | | -3.368151112 | Tc5 transposase D -binding domain |
| bmsk0003001 | 35.55666667 | | 10.62666667 | | -1.742430953 | tripeptidyl-peptidase 2 isoform X2 |
| mstrg.14615 | 44.89 | | 15.58333333 | | -1.526390232 | PREDICTED: poly [ADP-ribose] polymerase [Bombyx mori] |
| bmsk0013696 | 0.001 | | 0.076666667 | | 6.26052755 | XP 021203537.1 fatty acyl-CoA reductase wat-like isoform X2 |
| bmsk0013507 | 0.003333333 | | 0.223333333 | | 6.06608919 | uncharacterized protein LOC106116608 isoform X2 [Papilio xuthus] |
| bmsk0005656 | 1.153333333 | | 0.196666667 | | -2.551985178 | uncharacterized protein LOC105842971 |
| bmsk0003403 | 32.31333333 | | 9.183333333 | | -1.815039764 | circumsporozoite protein |
| mstrg.1693 | 29.94333333 | | 8.303333333 | | -1.850472312 | PREDICTED: LOW QUALITY PROTEIN: protein kintoun [Bombyx mori] |
| bmsk0011790 | 9.42 | | 2.703333333 | | -1.800987646 | XP 004927286.1 NF-kappa-B inhibitor-interacting Ras-like protein isoform X1 |
| bmsk0010506 | 54.88666667 | | 17.60666667 | | -1.640333925 | XP 004928165.1 ADP-dependent glucokinase |
| bmsk0010931 | 1.79 | | 0.43 | | -2.057551023 | uncharacterized protein LOC101747117 |
| bmsk0010571 | 0.001 | | 0.41 | | 8.6794801 | XP 004928219.1 uncharacterized protein LOC101741814 |
| bmsk0000428 | 0.583333333 | | 0.036666667 | | -3.991779493 | discoidin domain-containing receptor 2-like |
| bmsk0007933 | 0.83 | | 0.036666667 | | -4.500570313 | Plasmodium histidine-rich protein (HRPII/III) |
| bmsk0010387 | 0.001 | | 0.193333333 | | 7.594946589 | - |
| bmsk0009449 | 0.001 | | 0.29 | | 8.17990909 | Phospholipase A2 |
| bmsk0007508 | 0.756666667 | | 0.07 | | -3.434231065 | protein ALP1-like |
| bmsk0007121 | 13.17333333 | | 3.47 | | -1.924612878 | transcription initiation factor TFIID subunit 7-like |
| bmsk0013603 | 0.006666667 | | 0.22 | | 5.044394119 | XP 012549859.1 fatty acyl-CoA reductase wat |
| bmsk0003697 | 0.006666667 | | 0.06 | | 3.169925001 | protein unc-79 homolog isoform X4 |
| bmsk0003902 | 0.136666667 | | 0.41 | | 1.584962501 | uncharacterized protein LOC101746867 isoform X1 |
| bmsk0014110 | 2.09 | | 0.2 | | -3.385431037 | - |
| bmsk0015312 | 1.9 | | 0.21 | | -3.177538186 | - |
| bmsk0005267 | 23.79 | | 7.213333333 | | -1.721617273 | probable D replication complex GINS protein PSF2 [Amyelois transitella] |
| bmsk0011388 | 26.60333333 | | 8.11 | | -1.713833204 | XP 004934317.1 SAP30-binding protein |
| bmsk0011585 | 0.04 | | 0.22 | | 2.459431619 | - |
| bmsk0007074 | 0.001 | | 0.08 | | 6.321928095 | cadherin-related tumor suppressor-like, partial |
| bmsk0011411 | 0.001 | | 0.153333333 | | 7.26052755 | trypsin-like isoform X1 |
| bmsk0014343 | 0.001 | | 0.703333333 | | 9.458064783 | glycerophosphodiester phosphodiesterase 1-like isoform X1 |
| bmsk0001240 | 0.826666667 | | 0.12 | | -2.784271309 | solute carrier family 25 member 36-A [Amyelois transitella] |
| bmsk0003065 | 0.096666667 | | 0.43 | | 2.15324626 | Domain of unknown function (DUF4149) |
| bmsk0006314 | 0.61 | | 2.903333333 | | 2.25082907 | uncharacterized protein LOC106121342 [Papilio xuthus] |
| bmsk0005124 | 28.95 | | 9.823333333 | | -1.559278788 | RWD domain-containing protein 1 isoform X2 |
| bmsk0014603 | 0.12 | | 0.343333333 | | 1.516575526 | XP 004923380.3 uncharacterized protein LOC101745601 |
| bmsk0013393 | 0.296666667 | | 0.003333333 | | -6.475733431 | XP 004932766.1 catalase |
| bmsk0008585 | 5.203333333 | | 0.45 | | -3.531439225 | polycomb protein Pcl isoform X3 |
| bmsk0014218 | 10.69 | | 3.086666667 | | -1.792140255 | XP 004923153.1 D polymerase epsilon subunit 3 |
| bmsk0013009 | 1.06 | | 0.056666667 | | -4.225420114 | - |
| bmsk0011650 | 0.001 | | 0.253333333 | | 7.984893108 | - |
| bmsk0003842 | 0.58 | | 0.05 | | -3.5360529 | - |
| bmsk0011634 | 18.11 | | 5.11 | | -1.82539135 | uncharacterized protein LOC101734835 isoform X1 [Xenopus tropicalis] |
| bmsk0004093 | 27.34666667 | | 7.556666667 | | -1.855543101 | uncharacterized protein LOC106135326 [Amyelois transitella] |
| bmsk0013773 | 0.09 | | 0.983333333 | | 3.449683642 | XP 004926062.1 uncharacterized protein LOC101742885, partial |
| bmsk0008125 | 0.076666667 | | 0.446666667 | | 2.542527234 | UHRF1-binding protein 1-like |
| bmsk0011728 | 1.883333333 | | 0.083333333 | | -4.498250868 | - |
| bmsk0000376 | 1.233333333 | | 0.001 | | -10.26834705 | THAP domain-containing protein 9, partial [Camponotus floridanus] |
| bmsk0014519 | 0.013333333 | | 0.073333333 | | 2.459431619 | AMP deaminase 2-like [Amyelois transitella] |
| bmsk0009261 | 8.723333333 | | 1.91 | | -2.19130688 | zinc finger HIT domain-containing protein 2 [Papilio xuthus] |
| bmsk0003297 | 0.373333333 | | 0.01 | | -5.222392421 | tetraspanin-5 |
| bmsk0004587 | 15.69 | | 5.566666667 | | -1.49495975 | transcription elongation factor SPT6 |
| bmsk0010415 | 1.926666667 | | 0.243333333 | | -2.985101124 | XP 021203506.1 zinc finger protein 77 isoform X2 |
| bmsk0009908 | 3.793333333 | | 0.436666667 | | -3.118861841 | FANCI solenoid 1 |
| bmsk0012616 | 0.026666667 | | 0.893333333 | | 5.06608919 | cuticular protein RR-2 motif 91 precursor |
| bmsk0005492 | 5.326666667 | | 0.95 | | -2.487233584 | - |
| bmsk0006922 | 0.001 | | 0.333333333 | | 8.380821784 | nose resistant to fluoxetine protein 6-like |
| bmsk0010794 | 10.45666667 | | 3.36 | | -1.63788989 | acyl-CoA=lysophosphatidylglycerol acyltransferase 1-like |
| bmsk0012516 | 2.803333333 | | 0.7 | | -2.001716473 | XP 012550828.1 ATP-binding cassette sub-family G member 8 |
| bmsk0008225 | 0.503333333 | | 0.026666667 | | -4.238404739 | piggyBac transposable element-derived protein 4-like |
| bmsk0004951 | 1.15 | | 0.08 | | -3.845490051 | - |
| bmsk0011104 | 15.24666667 | | 4.353333333 | | -1.808301469 | XP 004933405.1 CWF19-like protein 1 |
| bmsk0001902 | 2.916666667 | | 0.28 | | -3.380821784 | U2 small nuclear ribonucleoprotein auxiliary factor 35 kDa subunit-related protein 2 isoform X1 |
| bmsk0002685 | 0.13 | | 0.296666667 | | 1.190331212 | Facilitated trehalose transporter Tret1; Short=BmTRET1 |
| bmsk0012337 | 2.163333333 | | 0.493333333 | | -2.132621302 | probable tR (guanine(26)-N(2))-dimethyltransferase isoform X1 |
| bmsk0011904 | 0.6 | | 0.063333333 | | -3.243925583 | kxDL motif-containing protein CG10681 [Amyelois transitella] |
| mstrg.9957 | 17.03333333 | | 5.313333333 | | -1.680671662 | Retrovirus-related Pol polyprotein from transposon TNT 1-94 [Papilio machaon] |
| bmsk0013784 | 12.58 | | 3.413333333 | | -1.881878708 | thyroid receptor-interacting protein 11-like |
| bmsk0011598 | 25.29333333 | | 7.71 | | -1.713954413 | XP 021207351.1 BSD domain-containing protein 1-B |
| bmsk0005358 | 0.001 | | 0.096666667 | | 6.594946589 | dopamine transporter |
| bmsk0001341 | 0.476666667 | | 1.89 | | 1.987333588 | cytochrome b5 reductase 4 isoform X1 [Amyelois transitella] |
| bmsk0003470 | 1.793333333 | | 0.213333333 | | -3.071462363 | - |
| bmsk0009546 | 0.303333333 | | 0.04 | | -2.922832139 | - |
| bmsk0006358 | 0.363333333 | | 0.016666667 | | -4.44625623 | enoyl-CoA delta isomerase 1, mitochondrial |
| bmsk0007113 | 0.01 | | 0.61 | | 5.930737338 | histidine-rich glycoprotein-like [Amyelois transitella] |
| bmsk0009232 | 9.613333333 | | 2.963333333 | | -1.69781584 | TBC1 domain family member 10B isoform X2 |
| bmsk0003844 | 31.35333333 | | 10.13333333 | | -1.629510009 | SAP domain-containing ribonucleoprotein |
| bmsk0008584 | 0.993333333 | | 0.196666667 | | -2.336525471 | high-affinity copper uptake protein 1 |
| bmsk0002015 | 2.24 | | 0.05 | | -5.485426827 | diacylglycerol O-acyltransferase 1 isoform X1 |
| bmsk0004092 | 1.99 | | 0.47 | | -2.082035769 | uncharacterized protein LOC110385281 |
| bmsk0009023 | 0.01 | | 0.096666667 | | 3.273018494 | probable cytochrome P450 9f2 |
| bmsk0009668 | 0.563333333 | | 0.001 | | -9.13784503 | - |
| bmsk0002304 | 2.966666667 | | 0.586666667 | | -2.338229907 | cell division control protein 48 isoform X1 |
| bmsk0009778 | 1.013333333 | | 4.13 | | 2.027032959 | Glycosyl hydrolases family 18 |
| bmsk0001650 | 0.2 | | 0.001 | | -7.64385619 | laminin subunit alpha-2 |
| bmsk0008268 | 0.01 | | 0.12 | | 3.584962501 | uncharacterized protein LOC101737570 isoform X1 |
| bmsk0005587 | 0.916666667 | | 0.023333333 | | -5.295932886 | sorting nexin-29-like isoform X2 |
| bmsk0015463 | 46.46333333 | | 14.59666667 | | -1.670453709 | carbonyl reductase [ DPH] 1-like [Papilio machaon] |
| bmsk0008721 | 0.456666667 | | 0.1 | | -2.191141487 | beta-1,3-galactosyltransferase 6 isoform X2 |
| bmsk0006369 | 0.443333333 | | 0.053333333 | | -3.055282436 | fas-binding factor 1 homolog |
| bmsk0007617 | 0.84 | | 0.05 | | -4.070389328 | uroporphyrinogen decarboxylase [Papilio machaon] |
| bmsk0015751 | 0.013333333 | | 0.12 | | 3.169925001 | uncharacterized protein LOC106138474 [Amyelois transitella] |
| bmsk0002195 | 0.22 | | 0.483333333 | | 1.135514971 | ras-related and estrogen-regulated growth inhibitor-like protein [Papilio machaon] |
| bmsk0012745 | 0.001 | | 0.086666667 | | 6.437405312 | cuticular protein RR-1 motif 27 precursor |
| bmsk0015907 | 1.916666667 | | 0.49 | | -1.967745801 | XP 021209292.1 multiple C2 and transmembrane domain-containing protein 1 isoform X4 |
| bmsk0007321 | 7.62 | | 1.853333333 | | -2.039668615 | D replication complex GINS protein PSF1-like [Plutella xylostella] |
| mstrg.10625 | 4.77 | | 1.23 | | -1.955330951 | PREDICTED: alpha-2-macroglobulin-like [Bombyx mori] |
| bmsk0005608 | 0.673333333 | | 0.08 | | -3.073248982 | preimplantation protein |
| bmsk0005512 | 0.001 | | 0.173333333 | | 7.437405312 | MICAL-like protein 1 isoform X1 |
| bmsk0003151 | 0.003333333 | | 0.543333333 | | 7.348728154 | carbonic anhydrase 2-like |
| bmsk0002399 | 0.11 | | 0.29 | | 1.398549376 | Caveolin |
| bmsk0004477 | 0.643333333 | | 0.02 | | -5.007494537 | NK-tumor recognition protein |
| bmsk0015036 | 8.626666667 | | 1.63 | | -2.403931247 | CREB-regulated transcription coactivator 1 |
| bmsk0007988 | 0.1 | | 0.001 | | -6.64385619 | NEDD8 ultimate buster 1 isoform X2 |
| bmsk0005002 | 0.29 | | 0.04 | | -2.857980995 | centrosomal protein of 290 kDa-like |
| bmsk0007240 | 7.78 | | 1.623333333 | | -2.260810884 | von Willebrand factor A domain-containing protein DDB G0286969-like isoform X1 [Amyelois transitella] |
| bmsk0007146 | 0.076666667 | | 0.166666667 | | 1.120294234 | - |
| bmsk0002452 | 44.59 | | 11.79666667 | | -1.918340939 | cAMP-regulated phosphoprotein 19 [Amyelois transitella] |
| bmsk0006395 | 17.17333333 | | 4.373333333 | | -1.973364873 | uncharacterized protein LOC106134980 [Amyelois transitella] |
| bmsk0007862 | 0.4 | | 0.056666667 | | -2.819427754 | phosphatidylinositol N-acetylglucosaminyltransferase subunit A [Papilio polytes] |
| bmsk0014467 | 0.001 | | 0.116666667 | | 6.866248611 | XP 021202954.1 uncharacterized zinc finger protein CG2678 |
| bmsk0006900 | 4.55 | | 0.706666667 | | -2.686764781 | WEB family protein At4g27595, chloroplastic isoform X1 |
| bmsk0004933 | 1.593333333 | | 0.096666667 | | -4.042885813 | - |
| bmsk0010518 | 5.73 | | 1.213333333 | | -2.239559189 | XP 021205019.1 uncharacterized protein LOC101735968 isoform X3 |
| bmsk0011196 | 2.173333333 | | 0.28 | | -2.956410731 | - |
| bmsk0005090 | 10.04 | | 0.08 | | -6.971543554 | synaptic vesicle glycoprotein 2B isoform X2 |
| bmsk0010774 | 0.06 | | 0.223333333 | | 1.896164189 | tetraspanin E118 isoform X1 |
| bmsk0008195 | 0.676666667 | | 0.046666667 | | -3.857980995 | intraflagellar transport protein 57 homolog [Amyelois transitella] |
| bmsk0010142 | 5.706666667 | | 0.366666667 | | -3.960107273 | U6 snR -associated Sm-like protein LSm5 |
| bmsk0012896 | 0.001 | | 0.14 | | 7.129283017 | cuticular protein RR-3 motif 148 precursor |
| bmsk0012213 | 27.21666667 | | 8.586666667 | | -1.664320292 | BAF62110.1 phosphomevalonate kinase |
| mstrg.22788 | 8.036666667 | | 2.036666667 | | -1.980387366 | PREDICTED: cytoplasmic tRNA 2-thiolation protein 2 [Bombyx mori] |
| bmsk0011452 | 3.76 | | 0.79 | | -2.250808104 | XP 021204634.1 facilitated trehalose transporter Tret1-2 homolog |
| bmsk0015068 | 15.34666667 | | 4.876666667 | | -1.653958064 | XP 004933009.1 TBC1 domain family member 7 |
| bmsk0000443 | 3.1 | | 0.476666667 | | -2.701215569 | period, partial |
| mstrg.14068 | 2.353333333 | | 0.29 | | -3.020580877 | PREDICTED: 5'-3' exoribonuclease 1 isoform X2 [Bombyx mori] |
| bmsk0001976 | 0.093333333 | | 0.001 | | -6.544320516 | - |
| bmsk0005662 | 1.096666667 | | 0.156666667 | | -2.807354922 | protein rogdi |
| bmsk0012953 | 28.7 | | 9.666666667 | | -1.569960337 | Uncharacterized protein OBRU01 07690 [Operophtera brumata] |
| bmsk0001259 | 2.87 | | 0.583333333 | | -2.298658316 | - |
| bmsk0004044 | 1.03 | | 0.11 | | -3.227068909 | uncharacterized protein LOC105841580 |
| bmsk0002073 | 24.69 | | 6.4 | | -1.947783026 | conserved oligomeric Golgi complex subunit 6 [Amyelois transitella] |
| bmsk0010313 | 0.001 | | 0.043333333 | | 5.437405312 | XP 004926673.1 inactive pancreatic lipase-related protein 1 |
| bmsk0005456 | 0.001 | | 0.083333333 | | 6.380821784 | uncharacterized protein LOC105842218 |
| bmsk0002220 | 0.36 | | 0.001 | | -8.491853096 | uncharacterized protein LOC101743717 |
| bmsk0004859 | 13.99 | | 4.81 | | -1.540287163 | protein AAR2 homolog [Amyelois transitella] |
| bmsk0009550 | 0.566666667 | | 0.001 | | -9.14635653 | - |
| bmsk0000087 | 8.566666667 | | 2.66 | | -1.687307708 | biogenesis of lysosome-related organelles complex 1 subunit 2 isoform X2 |
| mstrg.5081 | 17.75333333 | | 4.77 | | -1.896028756 | PREDICTED: FAST kinase domain-containing protein 5 [Bombyx mori] |
| bmsk0005326 | 34.34666667 | | 11.27333333 | | -1.607255934 | uncharacterized protein LOC106138168 [Amyelois transitella] |
| bmsk0013191 | 0.05 | | 0.25 | | 2.321928095 | partner of bursicon precursor |
| bmsk0001752 | 2.026666667 | | 0.186666667 | | -3.440572591 | uncharacterized protein LOC105841817 |
| bmsk0011516 | 0.016666667 | | 0.07 | | 2.070389328 | AFC87802.1 30K protein 4 |
| bmsk0014232 | 4.51 | | 0.72 | | -2.647058622 | XP 004923145.1 poly(A) polymerase type 3 |
| bmsk0013213 | 3.163333333 | | 0.57 | | -2.472411762 | - |
| bmsk0013115 | 16.31 | | 1.823333333 | | -3.161106545 | XP 021203221.1 methionine synthase reductase isoform X1 |
| mstrg.17774 | 6.233333333 | | 1.88 | | -1.729271202 | PREDICTED: zinc finger protein 711-like [Halyomorpha halys] |
| bmsk0009892 | 0.19 | | 0.396666667 | | 1.061927749 | Baculovirus FP protein |
| bmsk0010954 | 0.001 | | 0.493333333 | | 8.94641896 | AEL88625.1 amino acid transporter-like protein |
| bmsk0007380 | 0.55 | | 0.086666667 | | -2.665882496 | helicase POLQ-like isoform X1 |
| bmsk0013150 | 182.4633333 | | 55.49 | | -1.71730687 | XP 004921718.1 porimin |
| bmsk0003788 | 0.03 | | 0.68 | | 4.502500341 | uncharacterized protein LOC101740250 isoform X2 |
| bmsk0008756 | 0.08 | | 0.32 | | 2 | ras-like GTP-binding protein RhoL [Papilio xuthus] |
| bmsk0001978 | 1.103333333 | | 0.001 | | -10.107653 | WD repeat-containing protein 35-like |
| bmsk0004237 | 0.156666667 | | 0.001 | | -7.291554446 | calmodulin-binding transcription activator 2 |
| bmsk0010967 | 0.28 | | 1.006666667 | | 1.846087317 | hypothetical protein KGM 11096 [Danaus plexippus] |
| bmsk0012031 | 0.523333333 | | 0.01 | | -5.709658248 | uncharacterized protein LOC101745712 |
| bmsk0013545 | 6.906666667 | | 0.066666667 | | -6.694880193 | - |
| mstrg.4132 | 25.4 | | 9.03 | | -1.492030604 | PREDICTED: galectin-9-like isoform X1 [Bombyx mori] |
| bmsk0002903 | 0.07 | | 0.263333333 | | 1.911463325 | solute carrier family 12 member 4 isoform X1 |
| bmsk0005336 | 7.54 | | 1.44 | | -2.388495712 | SEC14-like protein 2 isoform X1 |
| bmsk0002082 | 10.43 | | 2.923333333 | | -1.835052911 | neuroguidin [Papilio polytes] |
| bmsk0008262 | 6.736666667 | | 2 | | -1.752034916 | ras-related protein Rab-30 [Papilio polytes] |
| bmsk0015457 | 0.413333333 | | 0.001 | | -8.691161905 | potassium voltage-gated channel protein Shal isoform X3 [Amyelois transitella] |
| bmsk0006066 | 20.89333333 | | 5.633333333 | | -1.890981933 | histone chaperone asf1 [Papilio polytes] |
| bmsk0013590 | 0.001 | | 0.11 | | 6.781359714 | esterase FE4-like isoform X1 |
| bmsk0011714 | 0.743333333 | | 0.096666667 | | -2.942918905 | mediator of R polymerase II transcription subunit 25-like isoform X4 |
[truncated: 29,051 more chars]
